# Supplementary material for: Expanding the terpene biosynthetic code with non-canonical 16 carbon atom building blocks
Source: Nat Commun. 2022 Sep 3;13:5188. doi: 10.1038/s41467-022-32921-w (PMC9440906; doi:10.1038/s41467-022-32921-w)
Supplement: Supplementary file 1 — Supplementary Information [file 41467_2022_32921_MOESM1_ESM.pdf]

Expanding the terpene biosynthetic code with non-canonical 16 carbon atom building blocks

Ignea *et al*.

Table of contents

Supplementary Tables 1
Supplementary Figures 16
Supplementary Notes 151
Supplementary references 155

List of Supplementary Tables

Supplementary Table 1. Summary of C_{16} compounds produced in yeast, including elucidated or proposed structures, proposed names and numbers in the text and figures... 1
Supplementary Table 2. Product profile of selected *Sp*SodMT variants.. 3
Supplementary Table 3. List of *Sp*SodMT double variants engineered in this study.......................... 4
Supplementary Table 4. Product profile of *Sp*SodMT variants analyzed using in vitro assays with SAM and FPP.. 5
Supplementary Table 5. List of terpene synthases and cytochrome P450s used in this study. 6
Supplementary Table 6. Production of C_{16} terpenes by *Ha*KS... 7
Supplementary Table 7. Production of main C16 olefins by Cyc2. 8
Supplementary Table 8. Production of oxidized C_{16} terpenes by *Pt*AO.. 9
Supplementary Table 9. ^{1}H NMR data (δ in ppm, J in Hz) of compounds **1a**, **1b**, ***epi*-1b**, **1c**, **1d** and **13OH-1b** in $CDCl_3$. 10
Supplementary Table 10. ^{1}H NMR data (δ in ppm, J in Hz) of compounds **2a**, **2b**, **2c**, **2d**, **3a**, **3b**, **3c** and **9OH-3b** in $CDCl_3$.. 11
Supplementary Table 11. ^{1}H NMR data (δ in ppm, J in Hz) of compounds **4a**, **4b**, **4c**, **5a**, **5b**, ***epi*-5b** and **6a** in $CDCl_3$. 12
Supplementary Table 12. ^{1}H NMR data (δ in ppm, J in Hz) of compounds **7a**, **7b**, **7c**, **8b**, ***epi*-8b**, **9b** and **10b** in $CDCl_3$.. 13
Supplementary Table 13. ^{13}C NMR data (δ in ppm) of compounds **1a**, **1b**, **1c**, **1d**, **13OH-1b**, **2a**, **2b**, **2c**, **2d**, **3a**, **3b**, **3c** and **9OH-3b** in $CDCl_3$.. 14
Supplementary Table 14. ^{13}C NMR data (δ in ppm) of compounds **4a**, **4b**, **4c**, **5a**, **5b**, **6a**, **7a**, **7b**, **7c**, **8b**, **9b** and **10b** in $CDCl_3$.. 15

List of Supplementary Figures

Supplementary Fig. 1. Chemical structures of C_{16} compounds isolated in pure form. 16
Supplementary Fig. 2. Overexpression of the yeast oxidoreductase Oye2p increases the levels of compound **1c.** 17
Supplementary Fig. 3. Assessment of cellular growth of AM109 yeast strain producing C_{16} terpenes.. 18
Supplementary Fig. 4. Sterol profile of AM109 yeast cells producing PSPP.................................... 19
Supplementary Fig. 5. Evaluation of *Sp*SodMT enzymatic activity.. 20

Supplementary Fig. 6. Synthesis of new C_{16} building blocks in yeast by *Sp*SodMT variants (continuation of Fig. 4)........ 22
Supplementary Fig. 7. Chromatogram of *Sp*SodMT(F58L) product profile. 23
Supplementary Fig. 8. Heat map representing the total C_{16} terpene production in engineered *Sp*SodMT double variants. 24
Supplementary Fig. 9. Activity assays of selected *Sp*SodMT variants with SAM and FPP as co-substrates. 26
Supplementary Fig. 10. Production of C_{16} compounds by *Sp*SodMT wt and mutants.. 27
Supplementary Fig. 11. Production of C_{16} olefins by Cyc2 in yeast cells expressing *Sp*SodMT(Q57N). 28
Supplementary Fig. 12. HR-APCI-GC-qTOF mass spectra of compounds tentatively assigned as **3d** and **4d**. 29
Supplementary Fig. 13. In vitro activity assays with *Sp*SodMT and Cyc2. 30
Supplementary Fig. 14. In vitro activity assay with *Sp*SodMT and *Ha*KS. 31
Supplementary Fig. 15. HR-APCI-GC-qTOF mass spectra of compounds **11-15**. 32
Supplementary Fig. 16. COSY and important HMBC correlations observed for building blocks **1**–**10** and side chains **a**–**d** constituting the isolated C_{16} compounds. 33
Supplementary Fig. 17. Key NOE correlations used for the assignment of the relative configuration of building blocks **1**–**10**, as exemplified by representative examples. 35
Supplementary Fig. 18. ^{1}H NMR spectrum (in $CDCl_3$) of compound **1a**. 36
Supplementary Fig. 19. LR-EI-MS spectrum of compound **1a**. 36
Supplementary Fig. 20. HR-APCI-MS spectrum of compound **1a**. 37
Supplementary Fig. 21. ^{13}C NMR spectrum (in $CDCl_3$) of compound **1a**. 37
Supplementary Fig. 22. HSQC spectrum (in $CDCl_3$) of compound **1a**. 38
Supplementary Fig. 23. HMBC spectrum (in $CDCl_3$) of compound **1a**. 38
Supplementary Fig. 24. COSY spectrum (in $CDCl_3$) of compound **1a**. 39
Supplementary Fig. 25. ^{1}H NMR spectrum (in $CDCl_3$) of compound **1b**. 40
Supplementary Fig. 26. LR-EI-MS spectrum of compound **1b**. 40
Supplementary Fig. 27. HR-APCI-MS spectrum of compound **1b**. 41
Supplementary Fig. 28. ^{13}C NMR spectrum (in $CDCl_3$) of compound **1b**. 41
Supplementary Fig. 29. HSQC spectrum (in $CDCl_3$) of compound **1b**. 42
Supplementary Fig. 30. HMBC spectrum (in $CDCl_3$) of compound **1b**. 42
Supplementary Fig. 31. COSY spectrum (in $CDCl_3$) of compound **1b**. 43
Supplementary Fig. 32. NOESY spectrum (in $CDCl_3$) of compound **1b**. 43
Supplementary Fig. 33. ^{1}H NMR spectrum (in $CDCl_3$) of compound ***epi*-1b**. 44
Supplementary Fig. 34. LR-EI-MS spectrum of compound ***epi*-1b**. 44
Supplementary Fig. 35. HR-APCI-MS spectrum of compound ***epi*-1b**. 45
Supplementary Fig. 36. ^{1}H NMR spectrum (in $CDCl_3$) of compound **1c**. 46
Supplementary Fig. 37. LR-EI-MS spectrum of compound **1c**. 46
Supplementary Fig. 38. HR-APCI-MS spectrum of compound **1c**. 47
Supplementary Fig. 39. HSQC spectrum (in $CDCl_3$) of compound **1c**. 47
Supplementary Fig. 40. HMBC spectrum (in $CDCl_3$) of compound **1c**. 48
Supplementary Fig. 41. COSY spectrum (in $CDCl_3$) of compound **1c**. 48
Supplementary Fig. 42. ^{1}H NMR spectrum (in $CDCl_3$) of compound **1d**. 49
Supplementary Fig. 43. LR-EI-MS spectrum of compound **1d**. 49
Supplementary Fig. 44. HR-APCI-MS spectrum of compound **1d**. 50
Supplementary Fig. 45. HSQC spectrum (in $CDCl_3$) of compound **1d**. 50
Supplementary Fig. 46. HMBC spectrum (in $CDCl_3$) of compound **1d**. 51
Supplementary Fig. 47. COSY spectrum (in $CDCl_3$) of compound **1d**. 51
Supplementary Fig. 48. NOESY spectrum (in $CDCl_3$) of compound **1d**. 52
Supplementary Fig. 49. ^{1}H NMR spectrum (in $CDCl_3$) of compound **13OH-1b**. 53

Supplementary Fig. 50. LR-EI-MS spectrum of compound **13OH-1b**. 53
Supplementary Fig. 51. HR-APCI-MS spectrum of compound **13OH-1b**.. 54
Supplementary Fig. 52. HSQC spectrum (in $CDCl_3$) of compound **13OH-1b**.................................. 54
Supplementary Fig. 53. HMBC spectrum (in $CDCl_3$) of compound **13OH-1b**................................. 55
Supplementary Fig. 54. COSY spectrum (in $CDCl_3$) of compound **13OH-1b**.................................. 55
Supplementary Fig. 55. 1D NOE difference spectrum (in $CDCl_3$) of compound **13OH-1b** upon irradiation of H_3-11. 56
Supplementary Fig. 56. 1D NOE difference spectrum (in $CDCl_3$) of compound **13OH-1b** upon irradiation of H_3-14. 56
Supplementary Fig. 57. 1D NOE difference spectrum (in $CDCl_3$) of compound **13OH-1b** upon irradiation of H_3-15. 57
Supplementary Fig. 58. ^{1}H NMR spectrum (in $CDCl_3$) of compound **2a**.. 58
Supplementary Fig. 59. LR-EI-MS spectrum of compound **2a**. 58
Supplementary Fig. 60. HR-APCI-MS spectrum of compound **2a**. 59
Supplementary Fig. 61. HSQC spectrum (in $CDCl_3$) of compound **2a**. 59
Supplementary Fig. 62. HMBC spectrum (in $CDCl_3$) of compound **2a**. 60
Supplementary Fig. 63. COSY spectrum (in $CDCl_3$) of compound **2a**. 60
Supplementary Fig. 64. ^{1}H NMR spectrum (in $CDCl_3$) of compound **2b**.. 61
Supplementary Fig. 65. LR-EI-MS spectrum of compound **2b**. 61
Supplementary Fig. 66. HR-APCI-MS spectrum of compound **2b**. 62
Supplementary Fig. 67. ^{13}C NMR spectrum (in $CDCl_3$) of compound **2b**... 62
Supplementary Fig. 68. HSQC spectrum (in $CDCl_3$) of compound **2b**. 63
Supplementary Fig. 69. HMBC spectrum (in $CDCl_3$) of compound **2b**. 63
Supplementary Fig. 70. COSY spectrum (in $CDCl_3$) of compound **2b**. 64
Supplementary Fig. 71. NOESY spectrum (in $CDCl_3$) of compound **2b**.. 64
Supplementary Fig. 72. 1D NOE difference spectrum (in $CDCl_3$) of compound **2b** upon irradiation of H_3-12. 65
Supplementary Fig. 73. 1D NOE difference spectrum (in $CDCl_3$) of compound **2b** upon irradiation of H_3-13. 65
Supplementary Fig. 74. 1D NOE difference spectrum (in $CDCl_3$) of compound **2b** upon irradiation of H_3-14. 66
Supplementary Fig. 75. ^{1}H NMR spectrum (in $CDCl_3$) of compound **2c**.. 67
Supplementary Fig. 76. LR-EI-MS spectrum of compound **2c**. 67
Supplementary Fig. 77. HR-APCI-MS spectrum of compound **2c**.. 68
Supplementary Fig. 78. HSQC spectrum (in $CDCl_3$) of compound **2c**.. 68
Supplementary Fig. 79. HMBC spectrum (in $CDCl_3$) of compound **2c**... 69
Supplementary Fig. 80. COSY spectrum (in $CDCl_3$) of compound **2c**.. 69
Supplementary Fig. 81. ^{1}H NMR spectrum (in $CDCl_3$) of compound **2d** (as a 1:1 mixture with **1d**). 70
Supplementary Fig. 82. HR-APCI-MS spectrum of compound **2d**. 70
Supplementary Fig. 83. ^{13}C NMR spectrum (in $CDCl_3$) of compound **2d** (as a 1:1 mixture with **1d**). 71
Supplementary Fig. 84. HSQC spectrum (in $CDCl_3$) of compound **2d** (as a 1:1 mixture with **1d**).... 71
Supplementary Fig. 85. HMBC spectrum (in $CDCl_3$) of compound **2d** (as a 1:1 mixture with **1d**)... 72
Supplementary Fig. 86. COSY spectrum (in $CDCl_3$) of compound **2d** (as a 1:1 mixture with **1d**).... 72
Supplementary Fig. 87. ^{1}H NMR spectrum (in $CDCl_3$) of compound **3a**.. 73
Supplementary Fig. 88. LR-EI-MS spectrum of compound **3a**. 73
Supplementary Fig. 89. HR-APCI-MS spectrum of compound **3a**. 74
Supplementary Fig. 90. HSQC spectrum (in $CDCl_3$) of compound **3a**. 74
Supplementary Fig. 91. HMBC spectrum (in $CDCl_3$) of compound **3a**. 75
Supplementary Fig. 92. COSY spectrum (in $CDCl_3$) of compound **3a**. 75
Supplementary Fig. 93. NOESY spectrum (in $CDCl_3$) of compound **3a**.. 76

Supplementary Fig. 94. ^{1}H NMR spectrum (in $CDCl_3$) of compound **3b**. 77
Supplementary Fig. 95. LR-EI-MS spectrum of compound **3b**. 77
Supplementary Fig. 96. HR-APCI-MS spectrum of compound **3b**. 78
Supplementary Fig. 97. ^{13}C NMR spectrum (in $CDCl_3$) of compound **3b**. 78
Supplementary Fig. 98. HSQC spectrum (in $CDCl_3$) of compound **3b**. 79
Supplementary Fig. 99. HMBC spectrum (in $CDCl_3$) of compound **3b**. 79
Supplementary Fig. 100. COSY spectrum (in $CDCl_3$) of compound **3b**. 80
Supplementary Fig. 101. NOESY spectrum (in $CDCl_3$) of compound **3b**. 80
Supplementary Fig. 102. ^{1}H NMR spectrum (in $CDCl_3$) of compound **3c**. 81
Supplementary Fig. 103. LR-EI-MS spectrum of compound **3c**. 81
Supplementary Fig. 104. HR-APCI-MS spectrum of compound **3c**. 82
Supplementary Fig. 105. HSQC spectrum (in $CDCl_3$) of compound **3c**. 82
Supplementary Fig. 106. HMBC spectrum (in $CDCl_3$) of compound **3c**. 83
Supplementary Fig. 107. COSY spectrum (in $CDCl_3$) of compound **3c**. 83
Supplementary Fig. 108. ^{1}H NMR spectrum (in $CDCl_3$) of compound **9OH-3b**. 84
Supplementary Fig. 109. LR-EI-MS spectrum of compound **9OH-3b**. 84
Supplementary Fig. 110. HR-APCI-MS spectrum of compound **9OH-3b**. 85
Supplementary Fig. 111. HSQC spectrum (in $CDCl_3$) of compound **9OH-3b**. 85
Supplementary Fig. 112. HMBC spectrum (in $CDCl_3$) of compound **9OH-3b**. 86
Supplementary Fig. 113. COSY spectrum (in $CDCl_3$) of compound **9OH-3b**. 86
Supplementary Fig. 114. 1D NOE difference spectrum (in $CDCl_3$) of compound **9OH-3b** upon irradiation of H-9. 87
Supplementary Fig. 115. 1D NOE difference spectrum (in $CDCl_3$) of compound **9OH-3b** upon irradiation of H_3-12. 87
Supplementary Fig. 116. 1D NOE difference spectrum (in $CDCl_3$) of compound **9OH-3b** upon irradiation of H_3-13. 88
Supplementary Fig. 117. ^{1}H NMR spectrum (in $CDCl_3$) of compound **4a**. 89
Supplementary Fig. 118. LR-EI-MS spectrum of compound **4a**. 89
Supplementary Fig. 119. HR-APCI-MS spectrum of compound **4a**. 90
Supplementary Fig. 120. ^{13}C NMR spectrum (in $CDCl_3$) of compound **4a**. 90
Supplementary Fig. 121. HSQC spectrum (in $CDCl_3$) of compound **4a**. 91
Supplementary Fig. 122. HMBC spectrum (in $CDCl_3$) of compound **4a**. 91
Supplementary Fig. 123. COSY spectrum (in $CDCl_3$) of compound **4a**. 92
Supplementary Fig. 124. NOESY spectrum (in $CDCl_3$) of compound **4a**. 92
Supplementary Fig. 125. 1D NOE difference spectrum (in $CDCl_3$) of compound **4a** upon irradiation of H_3-12. 93
Supplementary Fig. 126. 1D NOE difference spectrum (in $CDCl_3$) of compound **4a** upon irradiation of H_3-13. 93
Supplementary Fig. 127. 1D NOE difference spectrum (in $CDCl_3$) of compound **4a** upon irradiation of H_3-15. 94
Supplementary Fig. 128. ^{1}H NMR spectrum (in $CDCl_3$) of compound **4b**. 95
Supplementary Fig. 129. LR-EI-MS spectrum of compound **4b**. 95
Supplementary Fig. 130. HR-APCI-MS spectrum of compound **4b**. 96
Supplementary Fig. 131. ^{13}C NMR spectrum (in $CDCl_3$) of compound **4b**. 96
Supplementary Fig. 132. HSQC spectrum (in $CDCl_3$) of compound **4b**. 97
Supplementary Fig. 133. HMBC spectrum (in $CDCl_3$) of compound **4b**. 97
Supplementary Fig. 134. COSY spectrum (in $CDCl_3$) of compound **4b**. 98
Supplementary Fig. 135. NOESY spectrum (in $CDCl_3$) of compound **4b**. 98
Supplementary Fig. 136. 1D NOE difference spectrum (in $CDCl_3$) of compound **4b** upon irradiation of H-7. 99

Supplementary Fig. 137. 1D NOE difference spectrum (in $CDCl_3$) of compound **4b** upon irradiation of H_3-12. 99
Supplementary Fig. 138. 1D NOE difference spectrum (in $CDCl_3$) of compound **4b** upon irradiation of H_3-13. 100
Supplementary Fig. 139. 1D NOE difference spectrum (in $CDCl_3$) of compound **4b** upon irradiation of H_3-15. 100
Supplementary Fig. 140. ^{1}H NMR spectrum (in $CDCl_3$) of compound **4c**. 101
Supplementary Fig. 141. LR-EI-MS spectrum of compound **4c**. 101
Supplementary Fig. 142. HR-APCI-MS spectrum of compound **4c**. 102
Supplementary Fig. 143. ^{13}C NMR spectrum (in $CDCl_3$) of compound **4c**. 102
Supplementary Fig. 144. HSQC spectrum (in $CDCl_3$) of compound **4c**. 103
Supplementary Fig. 145. HMBC spectrum (in $CDCl_3$) of compound **4c**. 103
Supplementary Fig. 146. COSY spectrum (in $CDCl_3$) of compound **4c**. 104
Supplementary Fig. 147. ^{1}H NMR spectrum (in $CDCl_3$) of compound **5a**. 105
Supplementary Fig. 148. LR-EI-MS spectrum of compound **5a**. 105
Supplementary Fig. 149. HR-APCI-MS spectrum of compound **5a**. 106
Supplementary Fig. 150. HSQC spectrum (in $CDCl_3$) of compound **5a**. 106
Supplementary Fig. 151. HMBC spectrum (in $CDCl_3$) of compound **5a**. 107
Supplementary Fig. 152. COSY spectrum (in $CDCl_3$) of compound **5a**. 107
Supplementary Fig. 153. NOESY spectrum (in $CDCl_3$) of compound **5a**. 108
Supplementary Fig. 154. ^{1}H NMR spectrum (in $CDCl_3$) of compound **5b**. 109
Supplementary Fig. 155. LR-EI-MS spectrum of compound **5b**. 109
Supplementary Fig. 156. HR-APCI-MS spectrum of compound **5b**. 110
Supplementary Fig. 157. ^{13}C NMR spectrum (in $CDCl_3$) of compound **5b**. 110
Supplementary Fig. 158. HSQC spectrum (in $CDCl_3$) of compound **5b**. 111
Supplementary Fig. 159. HMBC spectrum (in $CDCl_3$) of compound **5b**. 111
Supplementary Fig. 160. COSY spectrum (in $CDCl_3$) of compound **5b**. 112
Supplementary Fig. 161. NOESY spectrum (in $CDCl_3$) of compound **5b**. 112
Supplementary Fig. 162. 1D NOE difference spectrum (in $CDCl_3$) of compound **5b** upon irradiation of H_3-12. 113
Supplementary Fig. 163. 1D NOE difference spectrum (in $CDCl_3$) of compound **5b** upon irradiation of H_3-13. 113
Supplementary Fig. 164. 1D NOE difference spectrum (in $CDCl_3$) of compound **5b** upon irradiation of H_3-14. 114
Supplementary Fig. 165. 1D NOE difference spectrum (in $CDCl_3$) of compound **5b** upon irradiation of H_3-15. 114
Supplementary Fig. 166. ^{1}H NMR spectrum (in $CDCl_3$) of compound ***epi*-5b**. 115
Supplementary Fig. 167. LR-EI-MS spectrum of compound ***epi*-5b**. 115
Supplementary Fig. 168. HR-APCI-MS spectrum of compound ***epi*-5b**. 116
Supplementary Fig. 169. ^{1}H NMR spectrum (in $CDCl_3$) of compound **6a**. 117
Supplementary Fig. 170. LR-EI-MS spectrum of compound **6a**. 117
Supplementary Fig. 171. HR-APCI-MS spectrum of compound **6a**. 118
Supplementary Fig. 172. ^{13}C NMR spectrum (in $CDCl_3$) of compound **6a**. 118
Supplementary Fig. 173. HSQC spectrum (in $CDCl_3$) of compound **6a**. 119
Supplementary Fig. 174. HMBC spectrum (in $CDCl_3$) of compound **6a**. 119
Supplementary Fig. 175. COSY spectrum (in $CDCl_3$) of compound **6a**. 120
Supplementary Fig. 176. NOESY spectrum (in $CDCl_3$) of compound **6a**. 120
Supplementary Fig. 177. 1D NOE difference spectrum (in $CDCl_3$) of compound **6a** upon irradiation of H_3-12. 121
Supplementary Fig. 178. 1D NOE difference spectrum (in $CDCl_3$) of compound **6a** upon irradiation of H_3-13. 121

Supplementary Fig. 179. ^{1}H NMR spectrum (in $CDCl_3$) of compound **7a**. 122
Supplementary Fig. 180. LR-EI-MS spectrum of compound **7a**. 122
Supplementary Fig. 181. HR-APCI-MS spectrum of compound **7a**. 123
Supplementary Fig. 182. ^{13}C NMR spectrum (in $CDCl_3$) of compound **7a**. 123
Supplementary Fig. 183. HSQC spectrum (in $CDCl_3$) of compound **7a**. 124
Supplementary Fig. 184. HMBC spectrum (in $CDCl_3$) of compound **7a**. 124
Supplementary Fig. 185. COSY spectrum (in $CDCl_3$) of compound **7a**. 125
Supplementary Fig. 186. NOESY spectrum (in $CDCl_3$) of compound **7a**. 125
Supplementary Fig. 187. 1D NOE difference spectrum (in $CDCl_3$) of compound **7a** upon irradiation of H_3-12. 126
Supplementary Fig. 188. 1D NOE difference spectrum (in $CDCl_3$) of compound **7a** upon irradiation of H_3-13. 126
Supplementary Fig. 189. 1D NOE difference spectrum (in $CDCl_3$) of compound **7a** upon irradiation of H_3-15. 127
Supplementary Fig. 190. ^{1}H NMR spectrum (in $CDCl_3$) of compound **7b**. 128
Supplementary Fig. 191. LR-EI-MS spectrum of compound **7b**. 128
Supplementary Fig. 192. HR-APCI-MS spectrum of compound **7b**. 129
Supplementary Fig. 193. HSQC spectrum (in $CDCl_3$) of compound **7b**. 129
Supplementary Fig. 194. HMBC spectrum (in $CDCl_3$) of compound **7b**. 130
Supplementary Fig. 195. COSY spectrum (in $CDCl_3$) of compound **7b**. 130
Supplementary Fig. 196. NOESY spectrum (in $CDCl_3$) of compound **7b**. 131
Supplementary Fig. 197. ^{1}H NMR spectrum (in $CDCl_3$) of compound **7c**. 132
Supplementary Fig. 198. LR-EI-MS spectrum of compound **7c**. 132
Supplementary Fig. 199. HR-APCI-MS spectrum of compound **7c**. 133
Supplementary Fig. 200. HSQC spectrum (in $CDCl_3$) of compound **7c**. 133
Supplementary Fig. 201. HMBC spectrum (in $CDCl_3$) of compound **7c**. 134
Supplementary Fig. 202. COSY spectrum (in $CDCl_3$) of compound **7c**. 134
Supplementary Fig. 203. NOESY spectrum (in $CDCl_3$) of compound **7c**. 135
Supplementary Fig. 204. ^{1}H NMR spectrum (in $CDCl_3$) of compound **8b**. 136
Supplementary Fig. 205. LR-EI-MS spectrum of compound **8b**. 136
Supplementary Fig. 206. HR-APCI-MS spectrum of compound **8b**. 137
Supplementary Fig. 207. HSQC spectrum (in $CDCl_3$) of compound **8b**. 137
Supplementary Fig. 208. HMBC spectrum (in $CDCl_3$) of compound **8b**. 138
Supplementary Fig. 209. COSY spectrum (in $CDCl_3$) of compound **8b**. 138
Supplementary Fig. 210. NOESY spectrum (in $CDCl_3$) of compound **8b**. 139
Supplementary Fig. 211. ^{1}H NMR spectrum (in $CDCl_3$) of compound ***epi*-8b**. 140
Supplementary Fig. 212. LR-EI-MS spectrum of compound ***epi*-8b**. 140
Supplementary Fig. 213. HR-APCI-MS spectrum of compound ***epi*-8b**. 141
Supplementary Fig. 214. ^{1}H NMR spectrum (in $CDCl_3$) of compound **9b**. 142
Supplementary Fig. 215. LR-EI-MS spectrum of compound **9b**. 142
Supplementary Fig. 216. HR-APCI-MS spectrum of compound **9b**. 143
Supplementary Fig. 217. ^{13}C NMR spectrum (in $CDCl_3$) of compound **9b**. 143
Supplementary Fig. 218. HSQC spectrum (in $CDCl_3$) of compound **9b**. 144
Supplementary Fig. 219. HMBC spectrum (in $CDCl_3$) of compound **9b**. 144
Supplementary Fig. 220. COSY spectrum (in $CDCl_3$) of compound **9b**. 145
Supplementary Fig. 221. NOESY spectrum (in $CDCl_3$) of compound **9b**. 145
Supplementary Fig. 222. ^{1}H NMR spectrum (in $CDCl_3$) of compound **10b**. 146
Supplementary Fig. 223. LR-EI-MS spectrum of compound **10b**. 146
Supplementary Fig. 224. HR-APCI-MS spectrum of compound **10b**. 147
Supplementary Fig. 225. ^{13}C NMR spectrum (in $CDCl_3$) of compound **10b**. 147
Supplementary Fig. 226. HSQC spectrum (in $CDCl_3$) of compound **10b**. 148

Supplementary Fig. 227. HMBC spectrum (in $CDCl_3$) of compound **10b**. 148
Supplementary Fig. 228. COSY spectrum (in $CDCl_3$) of compound **10b**. 149
Supplementary Fig. 229. NOESY spectrum (in $CDCl_3$) of compound **10b**...................................... 149
Supplementary Fig. 230. 1D NOE difference spectrum (in $CDCl_3$) of compound **10b** upon irradiation of H_3-12. 150

Supplementary Tables

Supplementary Table 1. Summary of C_{16} compounds produced in yeast, including elucidated or proposed structures, proposed names and numbers in the text and figures.

Building block	Core structure	Side chain (R)	Product name	Compound number	Structural analysis
		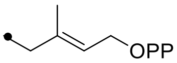	presodorifenyl diphosphate	**1**	Deduced
		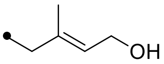	presodorifenol	**1a**	Elucidated
	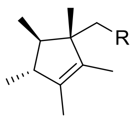	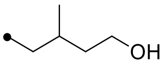	2,3-dihydro-presodorifenol	**1c**	Elucidated
		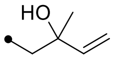	presodorifelool	**1b**	Elucidated
**1 PSPP**		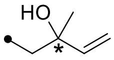	*epi*-presodorifelool	***epi*-1b**	Elucidated
		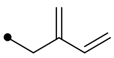	*β*-presodorifene	**1d**	Elucidated
	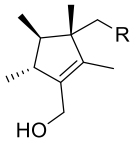	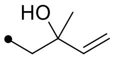	13-hydroxy-presodorifelool	**13OH-1b**	Elucidated
		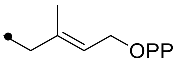	plymuthenyl diphosphate	**2**	Deduced
		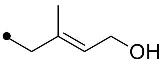	plymuthenol	**2a**	Elucidated
**2 PPP**	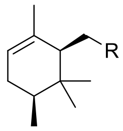	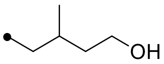	2,3-dihydro-plymuthenol	**2c**	Elucidated
		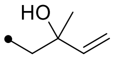	plymuthelool	**2b**	Elucidated
		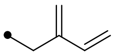	*β*-plymuthene	**2d**	Elucidated
		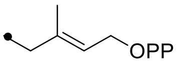	thorvaldsenyl diphosphate	**3**	Deduced
		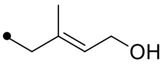	thorvaldsenol	**3a**	Elucidated
	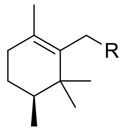	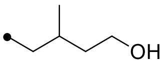	2,3-dihydro-thorvaldsenol	**3c**	Elucidated
**3 TPP**		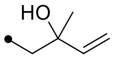	thorvaldselool	**3b**	Elucidated
		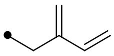	*β*-thorvaldsenene	**3d**	Proposed
	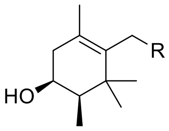	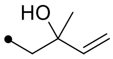	9-hydroxy-thorvaldselool	**9OH-3b**	Elucidated
		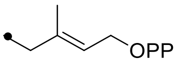	weylandtenyl diphosphate	**4**	Deduced
		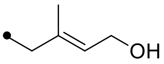	weylandtenol	**4a**	Elucidated
**4 WPP**	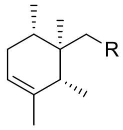	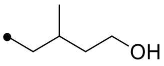	2,3-dihydro-weylandtenol	**4c**	Elucidated
		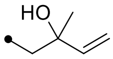	weylandtelool	**4b**	Elucidated
		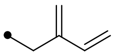	*β*-weylandtenene	**4d**	Proposed

Building block	Core structure	Side chain (R)	Product name	Compound number	Structural analysis
**5 BPP**	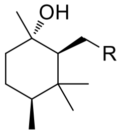	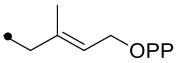	blixenyl diphosphate	**5**	Deduced
		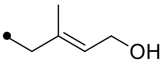	blixenol	**5a**	Elucidated
		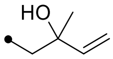	blixelool	**5b**	Elucidated
		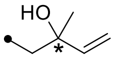	*epi*-blixelool	***epi*-5b**	Elucidated
**6 KPP**	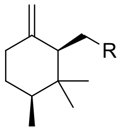	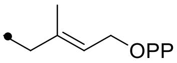	kimlarsenyl diphosphate	**6**	Deduced
		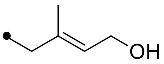	kimlarsenol	**6a**	Elucidated
**7 SPP**	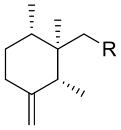	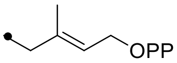	serratinyl diphosphate	**7**	Deduced
		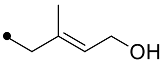	serratinol	**7a**	Elucidated
		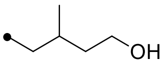	2,3-dihydro-serratinol	**7c**	Elucidated
		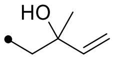	serratilool	**7b**	Elucidated
**8 JPP**	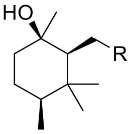	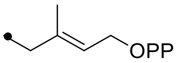	jacobsenyl diphosphate	**8**	Deduced
		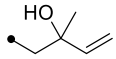	jacobselool	**8b**	Elucidated
		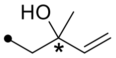	*epi*-jacobselool	***epi*-8b**	Elucidated
**9 HPP**	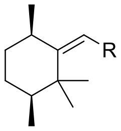	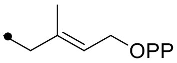	hammershoyl diphosphate	**9**	Deduced
		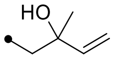	hammersholool	**9b**	Elucidated
**10 APP**	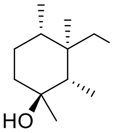	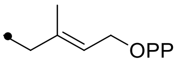	ancheryl diphosphate	**10**	Deduced
		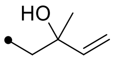	anchelool	**10b**	Elucidated

Supplementary Table 2. Product profile of selected *Sp*SodMT variants. Samples were analyzed in triplicate (n=3 biological replicates) and the mean value of the sum of products corresponding to each C_{16} diphosphate is shown. The C_{16} building block producing the most abundant product in each mutant is shown in bold. Source data provided as a Source Data file.

**Variant**	**Building blocks (%)**										
	**PSPP**	**PPP**	**TPP**	**WPP**	**BPP**	**KPP**	**SPP**	**JPP**	**HPP**	**APP**	**Total**
*Sp*SodMT wt	**100.00**	-	-	-	-	-	-	-	-	-	100
*Sp*SodMT(Q57N)	43.37	**52.95**	-	3.67		-	-	-	-	-	100
*Sp*SodMT(N219S)	**53.80**	46.20	-	-	-	-	-	-	-	-	100
*Sp*SodMT(F58V)	-	**77.07**	22.93	-	-	-	-	-	-	-	100
*Sp*SodMT(F58L)	-	29.59	**46.92**	15.21	6.14	2.14	-	-	-	-	100
*Sp*SodMT(F58M)	**45.97**	28.62	-	16.09	9.31	-	-	-	-	-	100
*Sp*SodMT(V273A)	10.44	2.15	**-**	**80.33**	4.11	2.39	-	-	0.57	-	100
*Sp*SodMT(L302Q)	32.05	3.43	1.82	-	**40.84**	21.87	-	-	-	-	100
*Sp*SodMT(L302S)	**61.29**	6.27	3.69	-	26.90	0.71	1.14	-	-	-	100
*Sp*SodMT(F58M-V273A)	2.43	**39.62**	25.27	19.93	8.77	3.82	-	-	0.16	-	100
*Sp*SodMT(F58M-L302Q)	12.20	13.52	-	14.51	21.89	**23.92**	-	13.97	-	-	100
*Sp*SodMT(F58M-L302S)	15.89	11.43	3.23	10.80	**39.94**	1.04	16.45	0.84	-	0.40	100

Supplementary Table 3. List of *Sp*SodMT double variants engineered in this study.

No	Mutant name
1.	*Sp*SodMT(Q57N-V273A)
2.	*Sp*SodMT(Q57N-L302Q)
3.	*Sp*SodMT(Q57N-L302S)
4.	*Sp*SodMT(Q57N-L302H)
5.	*Sp*SodMT(F58L-V273A)
6.	*Sp*SodMT(F58L-L302Q)
7.	*Sp*SodMT(F58L-L302S)
8.	*Sp*SodMT(F58L-L302H)
9.	*Sp*SodMT(F58M-V273A)
10.	*Sp*SodMT(F58M-L302Q)
11.	*Sp*SodMT(F58M-L302S)
12.	*Sp*SodMT(F58M-L302H)
13.	*Sp*SodMT(V273A-L302Q)
14.	*Sp*SodMT(V273A-L302S)
15.	*Sp*SodMT(V273A-L302H)

Supplementary Table 4. Product profile of *Sp*SodMT variants analyzed using in vitro assays with SAM and FPP. Samples were analyzed in duplicate (n=2 biological replicates) and the mean value of the sum of products corresponding to each C_{16} diphosphate is shown. Source data provided as a Source Data file.

**Variant**	**Building block (%)**									
	**PSPP**	**PPP**	**TPP**	**WPP**	**BPP**	**KPP**	**SPP**	**JPP**	**HPP**	**APP**
*Sp*SodMT	100.0	-	-	-	-	-	-	-	-	-
*Sp*SodMT(Q57N)	50.2	49.6	-	0.2	-	-	-	-	-	-
*Sp*SodMT(F58L)	-	25.8	61.0	13.0	-	0.2	-	-	-	-
*Sp*SodMT(V273A)	18.4	3.7	2.1	75.7	-	-	-	-	0.2	-
*Sp*SodMT(F58M-L302Q)	16.4	9.3	-	33.7	-	40.7	-	-	-	-
*Sp*SodMT(F58M-L302S)	19.7	15.5	-	33.5	9.6	-	20.7	-	-	0.2

Supplementary Table 5. List of terpene synthases and cytochrome P450s used in this study.

Class	Enzyme	Substrate	Organism	GenBank Accession	Source
**Sesqui-terpene synthases**	γ-humulene synthase	FPP	*Abies grandis*	U92267	[1]
	δ-cadinene synthase	FPP	*Salvia pomifera*	OK356797	[2]
	germacrene synthase	FPP	*Salvia fruticosa*	OK356799	[3]
	trans-β-caryophyllene synthase	FPP	*Salvia fruticosa*	OK356796	[4]
	trans-β-caryophyllene/α-humulene synthase	FPP	*Solanum elaeagnifolium*	OK356798	[5]
	*Sf*CinS(N338S/I451A)	GPP, FPP	*Salvia fruticosa*	ABH07677	[3]
	*Sf*CinS(N338C/I451A)	GPP, FPP	*Salvia fruticosa*	ABH07677	[3]
**Diterpene synthases**	abietadiene synthase	(+)-copalyl PP	*Picea abis*	AY779541	[6]
	levopimaradiene synthase	(+)-copalyl PP	*Gingko biloba*	AF331704	[7]
	miltiradiene synthase	(+)-copalyl PP	*Salvia pomifera*	KP119676	[8]
	cis-abienol synthase	8-hydroxy-copalyl-PP	*Nicotiana tabacum*	HE588140	[9]
	sclareol synthase	8-hydroxy-copalyl-PP	*Salvia sclarea*	JN133922	[10]
	terpentetriene synthase	terpentedienyl-PP, (+)-copalyl-PP, halimadienyl-PP, FPP, GGPP	*Kitasatospora griseola*	AB048795	[11]
	kolavelool synthase	kovalenyl PP	*Herpetosiphon aurantiacus*	ABX04786	[12]
**P450s**	CYP71AV1- artemisinic acid synthase	amorpha-4,11-diene	*Artemisia annua*	DQ318192	[13]
	CYP706M1 - valencene oxidase	valencene	*Callitropsis nootkatensis*	JX518290	[14]
	CYP71AV8 - valencene oxidase	valencene	*Cichorium intybus*	HQ166835	[15]
	CYP720B1 - abietadiene oxidase	abietadiene, miltiradiene	*Pinus taeda*	AY779537	[16]
	CYP76AH24 - ferruginol synthase	abietatriene, ferruginol	*Salvia pomifera*	KT157044	[17,18]
	CYP76AK6 - carnosic acid synthase	ferruginol, 11-hydroxy-ferruginol	*Salvia pomifera*	KT157045	[17,18]
	CYP71BE52 - salviol synthase	ferruginol	*Salvia pomifera*	KT157042	[17,18]
	CYP76AK1 - 11,20-hydroxy-ferruginol synthase	11-hydroxy-ferruginol	*Salvia miltiorrhiza*	KR140169	[19]
	CfCYP71D381 - MO oxidase	manoyl oxide	*Coleus forskohlii*	KT382342	[20]

Supplementary Table 6. Production of C_{16} terpenes by *Ha*KS. Production of C_{16} olefins in yeast strains co-expressing *Ha*KS with *Sp*SodMT wild-type or variants. Samples resulting from three different yeast transformations for each combination of enzymes were analyzed independently (n=3) and the mean yield of each compound was calculated. Errors correspond to the mean absolute deviation (MAD) around the mean. Source data provided as a Source Data file.

**Variant**	**Titer (mg/L)**								
	**1b**	**2b**	**3b**	**4b**	**5b**	**7b**	**8b**	**9b**	**10b**
*Sp*SodMT wt	59.30 ±3.84	0.60 ±0.17	-	0.04 ±0.01	-	-	-	-	-
*Sp*SodMT(Q57N)	34.46 ±3.86	33.77 ±4.10	-	0.53 ±0.10	-	-	-	0.21 ±0.03	-
*Sp*SodMT(N219S)	26.76 ±3.30	26.16 ±2.51	-	0.27 ±0.05	-	-	-	-	-
*Sp*SodMT(F58L)	-	8.03 ±1.09	11.25 ±0.80	3.98 ±0.22	1.03 ±0.17	-	-	-	-
*Sp*SodMT(F58M)	23.21 ±3.15	19.51 ±1.94	1.64 ±0.27	5.71 ±0.15	2.07 ±0.22	0.23 ±0.04	0.74 ±0.15	0.56 ±0.08	-
*Sp*SodMT(V273A)	7.13 ±1.84	2.15 ±0.28	0.86 ±0.11	15.00 ±0.91	1.88 ±0.14	-	1.55 ±0.14	2.49 ±0.26	-
*Sp*SodMT(L302Q)	6.87 ±1.87	0.39 ±0.06	0.23 ±0.34	0.08 ±0.002	2.87 ±0.12	-	1.18 ±0.12	-	-
*Sp*SodMT(L302S)	33.00 ±3.86	3.06 ±0.32	0.44 ±0.09	0.76 ±0.07	8.45 ±0.22	0.79 ±0.09	1.55 ±0.14	-	0.09 ±0.01
*Sp*SodMT(F58M-V273A)	0.51 ±0.09	14.09 ±2.24	8.81 ±0.95	4.77 ±0.24	1.94 ±0.29	-	0.59 ±0.08	3.18 ±0.29	-
*Sp*SodMT(F58M-L302Q)	1.66 ±0.22	1.15 ±0.08	-	0.85 ±0.03	1.26 ±0.12	-	4.67 ±0.40	-	-
*Sp*SodMT(F58M-L302S)	14.73 ±1.89	9.80 ±1.05	2.61 ±0.37	1.00 ±0.07	10.62 ±0.84	4.47 ±0.77	1.21 ±0.40	-	0.61 ±0.11

Supplementary Table 7. Production of main C16 olefins by Cyc2. Production of main C_{16} olefins in yeast strains co-expressing Cyc2 with *Sp*SodMT wild-type or variants. Samples resulting from three different yeast transformations for each combination of enzymes were analyzed independently (n=3) and the mean yield of each compound was calculated. Errors correspond to the mean absolute deviation (MAD) around the mean. Source data provided as a Source Data file.

**Variant**	**Titer (mg/L)**	
	**1d**	**2d**
*Sp*SodMT wt	10.27±1.49	-
*Sp*SodMT(Q57N)	11.63±2.66	5.38±0.78
*Sp*SodMT(N219S)	3.77±0.89	1.91±0.20
*Sp*SodMT(F58L)	-	0.59±0.08
*Sp*SodMT(F58M)	1.99±0.36	1.06±0.15
*Sp*SodMT(V273A)	0.67±0.13	0.16±0.04
*Sp*SodMT(L302Q)	0.31±0.11	0.15±0.04
*Sp*SodMT(L302S)	5.93±1.51	0.46±0.04
*Sp*SodMT(F58M-V273A)	0.03±0.008	0.76±0.13
*Sp*SodMT(F58M-L302Q)	0.16±0.03	0.16±0.03
*Sp*SodMT(F58M-L302S)	1.15±0.26	0.05±0.01

Supplementary Table 8. Production of oxidized C_{16} terpenes by *Pt*AO. Yeast strains co-expressing *Sp*SodMT wt or variants were used to analyze formation of oxidized C_{16} compounds by *Pt*AO in the presence of *Ha*KS**.** Samples resulting from three different yeast transformations for each combination of enzymes were analyzed independently (n=3) and the mean yield of each compound is shown. Errors correspond to the mean absolute deviation (MAD) around the mean. Source data provided as a Source Data file.

***Sp*SodMT variant**	**Titer (mg/L)**	
	**13OH-1b**	**9OH-3b**
*Sp*SodMT	35.34±2.11	-
*Sp*SodMT (Q57N)	8.98±0.98	-
*Sp*SodMT (F58L)	-	8.82±1.14
*Sp*SodMT (F58M)	14.16±2.97	2.12±0.34
*Sp*SodMT (V273A)	0.54±0.12	-
*Sp*SodMT (L302S)	20.69±1.76	-
*Sp*SodMT (F58M-V273A)	-	7.62±0.67

Supplementary Table 9. ^{1}H NMR data (δ in ppm, J in Hz) of compounds **1a**, **1b**, ***epi*-1b**, **1c**, **1d** and **13OH-1b** in $CDCl_3$.

Position	1a	1b	*epi*-1b	1c	1d	13OH-1b
1	4.12 d (7.0)	5.180 dd (17.3, 1.2), 5.029 dd (10.8, 1.2)	5.175 dd (17.4, 1.3), 5.027 dd (10.8, 1.3)	3.66 m	5.19 brd (17.5), 5.02 brd (10.7)	5.18 dd (17.4, 1.2), 5.04 dd (10.8, 1.2)
2	5.38 brt (7.0)	5.880 dd (17.3, 10.8)	5.881 dd (17.4, 10.8)	1.59 m, 1.34 m	6.35 dd (17.5, 10.7)	5.87 dd (17.4, 10.8)
3	-	-	-	1.45 m	-	-
4	1.91 m, 1.68 m	1.46 m, 1.22 m	1.46 m, 1.22 m	0.84 m, 1.22 m	2.13 m, 1.89 m	1.48 m, 1.18 m
5	1.35 m	1.24 m	1.24 m	1.25 m, 1.19 m	1.44 m	1.30 m
9	1.95 m	1.95 m	1.95 m	1.94 m	1.96 m	2.24 m
10	1.37 m	1.35 m	1.35 m	1.35 m	1.45 m	1.41 m
11	0.70 s	0.695 s	0.695 s	0.68 s	0.72 s	0.75 s
12	1.46 s	1.438 s	1.446 s	1.44 s	1.48 s	1.55 brs
13	1.50 s	1.491 s	1.491 s	1.50 s	1.52 s	4.17 d (11.8), 4.06 d (11.8)
14	0.96 d (6.8)	0.952 d (6.8)	0.953 d (6.8)	0.95 d (6.8)	0.98 d (6.8)	1.06 d (6.8)
15	0.88 d (7.0)	0.869 d (7.0)	0.865 d (7.0)	0.87 d (6.9)	0.91 d (7.0)	0.89 d (7.0)
16	1.66 brs	1.254 s	1.253 s	0.88 d (6.3)	4.95 brs	1.26 s

Supplementary Table 10. ^{1}H NMR data (δ in ppm, J in Hz) of compounds **2a**, **2b**, **2c**, **2d**, **3a**, **3b**, **3c** and **9OH-3b** in $CDCl_3$.

**Position**	**2a**	**2b**	**2c**	**2d**	**3a**	**3b**	**3c**	**9OH-3b**
1	4.14 d (6.9)	5.20 dd (17.4, 1.0), 5.04 dd (10.8, 1.0)	3.68 m	5.24 brd (17.6), 5.05 brd (10.8)	4.15 d (6.9)	5.21 dd (17.4, 1.2), 5.06 dd (10.8, 1.2)	3.68 m	5.21 dd (17.4, 1.2), 5.07 dd (10.8, 1.2)
2	5.41 brt (6.9)	5.92 dd (17.4, 10.8)	1.65 m, 1.36 m	6.36 dd (17.6, 10.8)	5.43 brt (6.9)	5.94 dd (17.4, 10.8)	1.64 m, 1.39 m	5.93 dd (17.4, 10.8)
3	-	-	1.53 m	-	-	-	1.55 m	-
4	2.22 m, 1.97 m	1.78 m, 1.44 m	1.52 m, 1.15 m	2.44 m, 2.16 m	2.05 m	1.57 m	1.34 m, 1.24 m	1.53 m
5	1.60 m, 1.32 m	1.56 m, 1.22 m	1.52 m, 1.16 m	1.67 m, 1.36 m	2.10 m, 2.06 m	2.00 m	1.97 m	2.04 m, 1.92 m
6	1.59 m	1.54 m	1.53 m	1.65 m	-	-	-	-
8	1.41 m	1.41 m	1.41 m	1.43 m	1.42 m	1.39 m	1.38 m	1.55 m
9	1.84 m, 1.66 m	1.82 m, 1.65 m	1.83 m, 1.67 m	1.85 m, 1.66 m	1.51 m, 1.35 m	1.48 m, 1.32 m	1.49 m, 1.33 m	4.11 ddd (9.6, 6.1, 3.7)
10	5.31 m	5.29 m	5.30 m	5.32 m	1.96 m, 1.85 dt (17.2, 4.6)	1.93 m, 1.83 dt (17.1, 4.7)	1.93 m, 1.82	2.07 m, 1.94 m
12	0.88 s	0.88 s	0.88 s	0.88 s	0.82 s	0.80 s	0.80 s	1.04 s
13	0.62 s	0.62 s	0.62 s	0.62 s	1.00 s	0.98 s	0.97 s	0.99 s
14	0.82 d (6.8)	0.81 d (6.8)	0.82 d (6.8)	0.82 d (6.8)	0.86 d (6.6)	0.85 d (6.6)	0.85 d (6.5)	0.81 d (6.9)
15	1.69 brs	1.66 brs	1.66 brs	1.72 brs	1.59 brs	1.56 brs	1.56 brs	1.57 brs
16	1.69 brs	1.28 s	0.92 d (6.4)	4.98 brs	1.70 brs	1.28 s	0.93 d (6.6)	1.29 s

Supplementary Table 11. ^{1}H NMR data (δ in ppm, J in Hz) of compounds **4a**, **4b**, **4c**, **5a**, **5b**, ***epi*-5b** and **6a** in $CDCl_3$.

Position	4a	4b	4c	5a	5b	*epi*-5b	6a
1	4.12 d (6.9)	5.19 dd (17.4, 1.2), 5.05 dd (10.8, 1.2)	3.66 m	4.14 dd (6.9, 2.3)	5.206 dd (17.4, 1.3), 5.024 dd (10.8, 1.3)	5.224 dd (17.3, 1.4), 5.059 dd (10.7, 1.4)	4.14 d (6.9)
2	5.40 brt (6.9)	5.89 dd (17.4, 10.8)	1.62 m, 1.36 m	5.43 brt (6.9)	5.927 dd (17.4, 10.8)	5.882 dd (17.3, 10.7)	5.39 brt (6.9)
3	-	-	1.46 m	-	-	-	
4	1.90 m, 1.71 m	1.42 m, 1.34 m	1.22 m, 1.02 m	2.14 m, 2.08 m	1.67 m	1.67 m	2.15 m, 1.82 m
5	1.43 m	1.40 m, 1.32 m	1.36 m, 1.22 m	1.58 m, 1.44 m	1.53 m, 1.40 m	1.53 m, 1.40 m	1.66 m, 1.49 m
6	-	-	-	1.07 t (4.0)	1.15 t (4.0)	1.15 t (4.0)	1.62 m
7	2.14 m	2.09 m	2.13 m	-	-	-	-
8	-	-	-	1.24 m	1.23 m	1.23 m	1.38 m
9	5.31 m	5.30 m	5.30 brs	1.44 m, 1.24 m	1.44 m, 1.24 m	1.44 m, 1.24 m	1.54 m, 1.21 m
10	1.84 m, 1.65 m	1.83 m, 1.67 m	1.83 m, 1.65 m	1.73 m, 1.40 m	1.70 m, 1.40 m	1.70 m, 1.40 m	2.26 ddd (12.5, 3.7, 2.9), 1.96 ddd (12.5, 12.5, 4.8)
11	1.64 m	1.59 m	1.62 m	-	-	-	-
12	0.59 s	0.59 s	0.58 s	0.91 s	0.894 s	0.885 s	0.95 s
13	0.88 d (7.3)	0.86 d (7.3)	0.86 d (7.3)	0.65 s	0.636 s	0.621 s	0.52 s
14	1.62 brs	1.60 brs	1.60 brs	0.81 d (6.3)	0.803 d (6.3)	0.801 d (6.3)	0.81 d (6.7)
15	0.81 d (6.4)	0.79 d (6.6)	0.78 d (6.5)	1.10 s	1.122 s	1.114 s	4.82 brs, 4.51 brs
16	1.68 brs	1.27 s	0.90 d (6.6)	1.70 brs	1.273 s	1.245 s	1.67 brs

Supplementary Table 12. ^{1}H NMR data (δ in ppm, J in Hz) of compounds **7a**, **7b**, **7c**, **8b**, ***epi*-8b**, **9b** and **10b** in $CDCl_3$.

Position	7a	7b	7c	8b	*epi*-8b	9b	10b
1	4.13 d (6.8)	5.20 dd (17.4, 1.1), 5.06 dd (10.8, 1.1)	3.68 m	5.216 dd (17.4, 1.2), 5.064 dd (10.8, 1.2)	5.216 dd (17.4, 1.2), 5.066 dd (10.8, 1.2)	5.18 dd (17.3, 1.3), 5.03 dd (10.7, 1.3)	5.18 dd (17.3, 1.0), 5.05 dd (10.8, 1.0)
2	5.41 brt (6.8)	5.91 dd (17.4, 10.8)	1.63 m, 1.37 m	5.931 dd (17.4, 10.8)	5.927 dd (17.4, 10.8)	5.91 dd (17.3, 10.7)	5.87 dd (17.3, 10.8)
3	-	-	1.48 m	-	-	-	-
4	1.93 m	1.45 m	1.27 m, 1.06 m	1.64 m, 1.52 m	1.64 m, 1.52 m	2.39 dd (14.3, 8.8), 2.15 dd (14.3, 6.3)	1.43 m, 1.35 m
5	1.46 m, 1.39 m	1.35 m, 1.26 m	1.30 m	1.45 m, 1.35 m	1.45 m, 1.35 m	5.29 dd (8.8, 6.3)	1.27 m, 1.22 m
6	-	-	-	0.76 m	0.76 m	-	-
7	2.09 q (6.9)	2.04 q (6.9)	2.06 q (6.8)	-	-	-	1.42 m
8	-	-	-	1.16 m	1.16 m	1.24 m	-
9	2.28 ddd (13.0, 4.4, 2.2), 2.03 m	2.26 ddd (12.9, 4.4, 2.2), 2.00 m	2.26 ddd (12.9, 4.5, 2.3), 2.00 m	1.51 m, 1.26 m	1.51 m, 1.26 m	1.51 m, 1.31 m	1.76 m, 1.38 m
10	1.50 m, 1.32 m	1.49 m, 1.29 m	1.49 m, 1.30 m	1.58 m, 1.44 m	1.58 m, 1.44 m	1.50 m	1.42 m, 1.25 m
11	1.65 m	1.61 m	1.64 m	-	-	2.94 m	1.45 m
12	0.52 s	0.52 s	0.51 s	0.840 s	0.848 s	1.03 s	0.65 s
13	0.93 d (6.9)	0.89 d (6.9)	0.90 d (6.8)	0.790 s	0.793 s	0.91 s	0.87 d (7.0)
14	4.73 brs, 4.53 brs	4.72 brs, 4.52 brs	4.71 brs, 4.52 brs	0.836 d (6.8)	0.832 d (6.8)	0.87 d (6.7)	1.10 s
15	0.79 d (6.8)	0.76 d (6.8)	0.76 d (6.8)	1.131 s	1.122 s	1.06 d (7.6)	0.76 d (6.7)
16	1.69 brs	1.28 s	0.91 d (6.4)	1.288 s	1.293 s	1.26 s	1.26 s

Supplementary Table 13. ^{13}C NMR data (δ in ppm) of compounds **1a**, **1b**, **1c**, **1d**, **13OH-1b**, **2a**, **2b**, **2c**, **2d**, **3a**, **3b**, **3c** and **9OH-3b** in $CDCl_3$.

Position	1a	1b	1c†	1d†	13OH-1b†	2a†	2b	2c†	2d†	3a†	3b	3c†	9OH-3b†
1	59.4	111.5	61.1	112.9	111.6	59.4	111.7	61.3	113.2	59.7	111.7	60.8	111.6
2	122.6	145.2	39.8	138.9	144.9	123.2	145.1	39.8	139.0	122.9	145.0	39.4	144.6
3	141.0	73.3	30.3	147.6	72.7	140.7	73.6	30.5	147.2	140.9	73.6	29.9	73.2
4	34.7	37.2	31.6	26.4	36.8	42.1	44.9	40.0	34.1	40.3	42.4	36.8	42.0
5	36.3	31.3	35.1	36.8	30.8	26.9	22.5	25.8	27.6	27.8	23.0	26.2	22.3
6	50.2	49.9	50.1	50.4	50.2	50.7	51.0	51.2	51.0	137.0	136.7	137.3	135.1
7	136.6	136.6	136.9	137.1	142.4	36.1	36.3	36.0	36.2	38.9	38.3	38.2	40.5
8	134.1	134.1	133.6	134.3	137.1	38.5	38.6	38.6	38.9	39.4	39.3	39.0	45.0
9	47.8	47.7	47.5	47.7	44.7	32.2	31.9	31.9	32.2	27.4	27.3	26.8	67.1
10	46.1	45.9	45.9	46.2	45.8	121.9	121.8	121.6	122.1	31.6	31.6	31.0	37.8
11	19.5	19.7	19.3	19.4	19.5	136.2	136.3	136.5	136.5	127.1	126.8	126.0	123.8
12	10.0	10.0	9.9	10.0	9.8	26.2	26.2	26.3	26.4	21.9	21.8	21.0	29.9
13	11.9	11.9	11.9	11.8	57.3	14.4	14.4	14.2	14.6	27.1	27.0	26.5	25.3
14	17.4	17.3	16.9	17.2	17.0	15.8	15.9	16.0	16.2	16.8	16.6	16.2	8.5
15	12.8	12.7	12.7	12.7	12.5	22.4	22.6	22.4	22.9	20.1	19.9	19.5	19.5
16	16.5	27.7	19.3	115.1	27.8	16.3	27.5	19.8	115.9	16.4	27.5	19.2	27.5

† Determined through HMBC correlations.

Supplementary Table 14. ^{13}C NMR data (δ in ppm) of compounds **4a**, **4b**, **4c**, **5a**, **5b**, **6a**, **7a**, **7b**, **7c**, **8b**, **9b** and **10b** in $CDCl_3$.

Position	4a	4b	4c	5a†	5b	6a	7a	7b†	7c†	8b†	9b	10b
1	59.5	111.7	61.3	59.2	111.4	59.5	59.5	112.0	61.3	111.7	111.7	111.8
2	122.8	145.2	39.9	123.0	145.8	123.0	122.9	145.2	39.8	144.8	145.1	145.0
3	140.9	73.4	30.3	141.0	73.7	140.6	140.8	73.6	30.1	73.4	72.6†	73.3
4	32.0	34.9	29.3	43.1	45.2	38.6	32.2	34.7	29.1	46.1	40.2	34.9
5	34.8	29.8	33.4	24.4	20.0	23.3	35.4	30.5	33.8	20.1	115.4	31.6
6	37.3	37.0	37.0	57.9	58.2	53.4	40.4	40.5	40.3	54.9	154.7†	38.7
7	39.1	39.0	39.1	38.9	38.6	39.3	42.1	42.2	41.9	37.9	39.6	46.1
8	136.4	136.4	136.6	42.4	42.5	42.3	151.9	152.1	152.1	42.4	41.7	73.3
9	121.0	121.0	121.0	29.3	29.3	33.5	37.0	37.2	37.1	26.5	26.5	43.1
10	32.4	32.0	32.0	43.3	43.3	37.8	32.2	32.3	32.2	40.8	32.4	28.7
11	33.5	33.4	33.4	74.0	74.8	148.8	36.8	36.8	36.6	73.0	29.9	36.3
12	14.8	14.8	14.9	28.9	29.0	26.7	14.7	14.9	14.8	27.8	16.9	16.6
13	12.0	11.9	12.0	15.6	15.6	14.2	11.1	11.2	11.1	15.5	24.0	7.8
14	22.2	22.2	22.3	15.9	16.1	16.5	106.2	106.3	105.9	16.2	17.1	23.0
15	15.4	15.3	15.4	22.8	23.3	106.4	15.6	15.7	15.6	30.5	21.1	15.6
16	16.5	27.8	19.7	16.4	27.5	16.4	16.5	27.8	19.7	27.4	27.5	27.9

† Determined through HMBC correlations.

Supplementary Figures

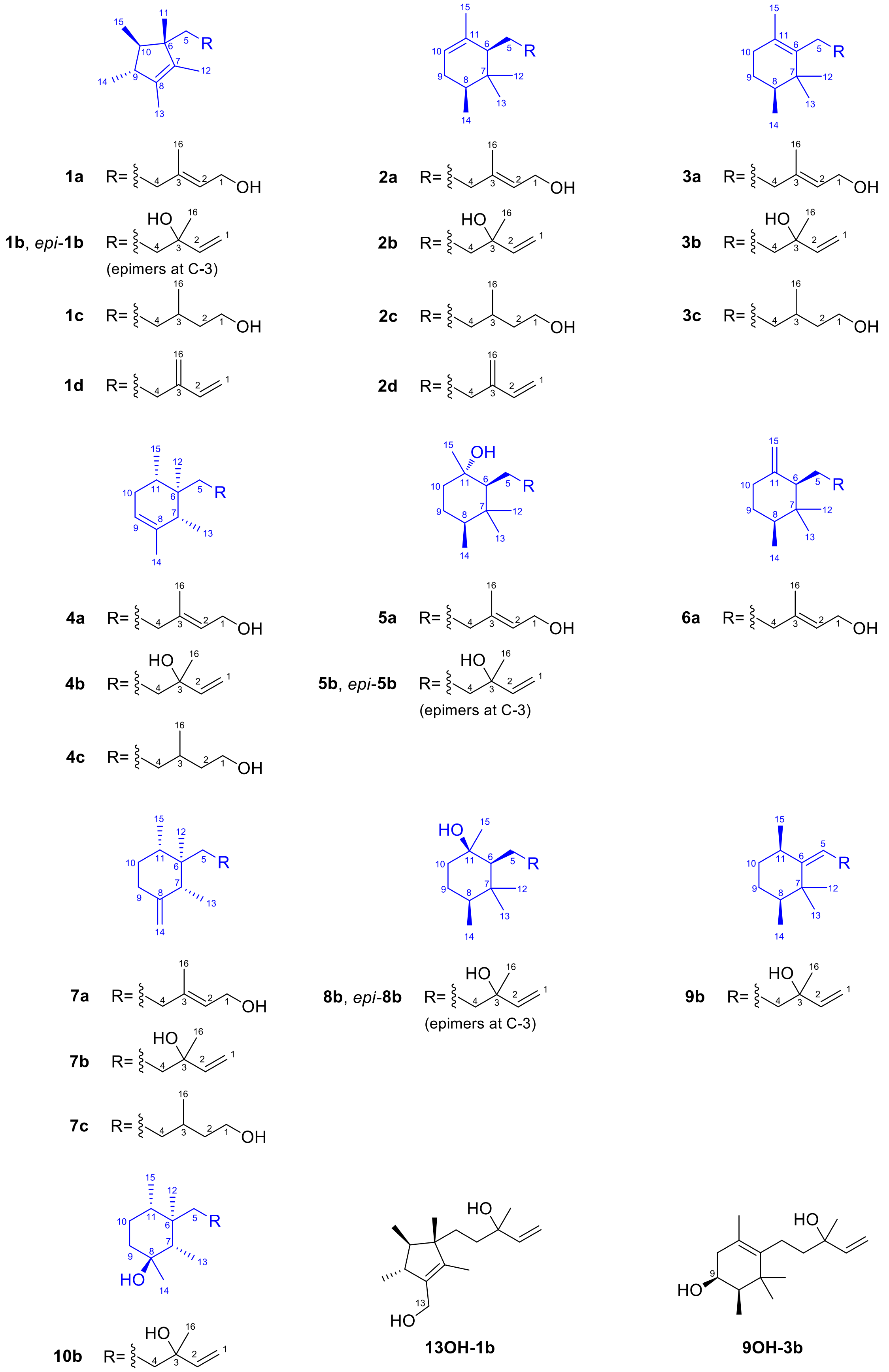

Supplementary Fig. 1. Chemical structures of C_{16} compounds isolated in pure form. The chemical structures of the isolated compounds depict only their relative configuration, as determined on the basis of their NMR data.

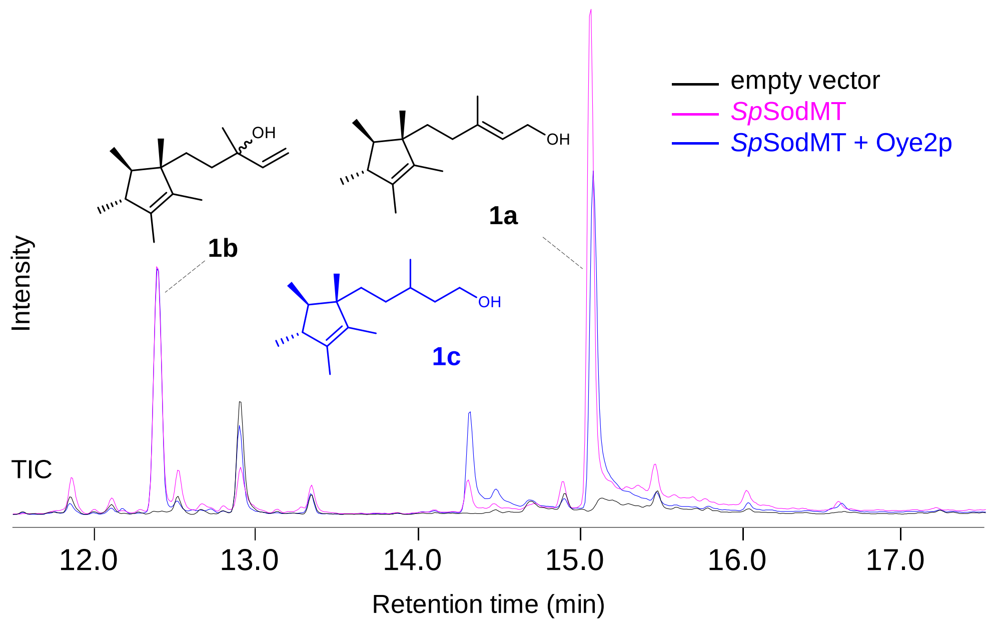

Supplementary Fig. 2. Overexpression of the yeast oxidoreductase Oye2p increases the levels of compound **1c.** Overexpression of Oye2p enzyme in *Sp*SodMT-expressing yeast cells resulted in 2-fold increase in production of compound **1c** without increase in the titers of **1b**. This suggests that Oye2p contributes to the synthesis of **1c** in yeast cells, which is in agreement with its role in the formation of the structurally-related citronellol from geraniol in yeast cells[21,22].

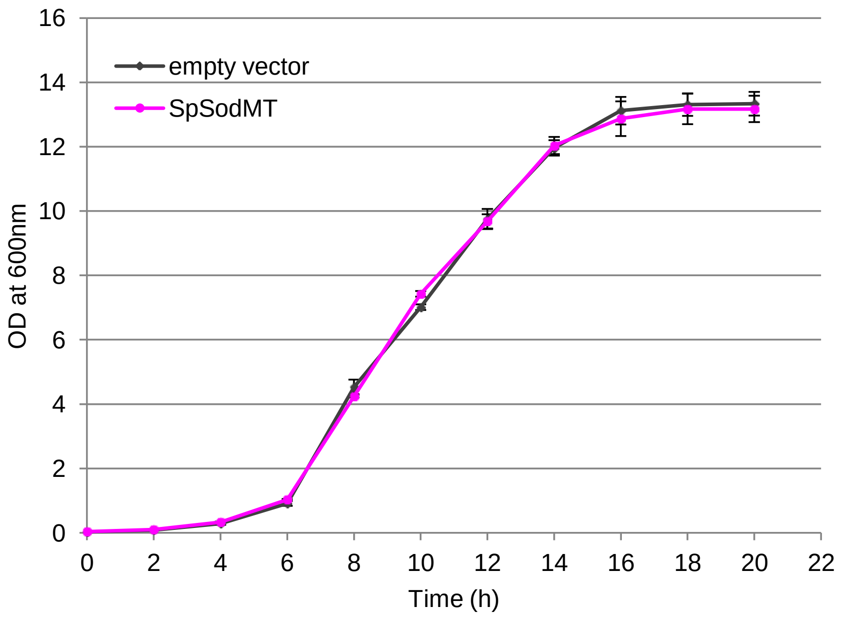

Supplementary Fig. 3. Assessment of cellular growth of AM109 yeast strain producing C_{16} terpenes. To determine the effect of C_{16} terpene production in yeast, we evaluated the growth of AM109 yeast cells expressing *Sp*SodMT (pink) or empty vector (black). We did not observe any significant toxicity of the C_{16} products on yeast growth. Errors correspond to the mean absolute deviation (MAD) around the mean (n = 3 different starting cultures for each condition). Individual data points not shown for clarity but can be found in the associated Source Data file.

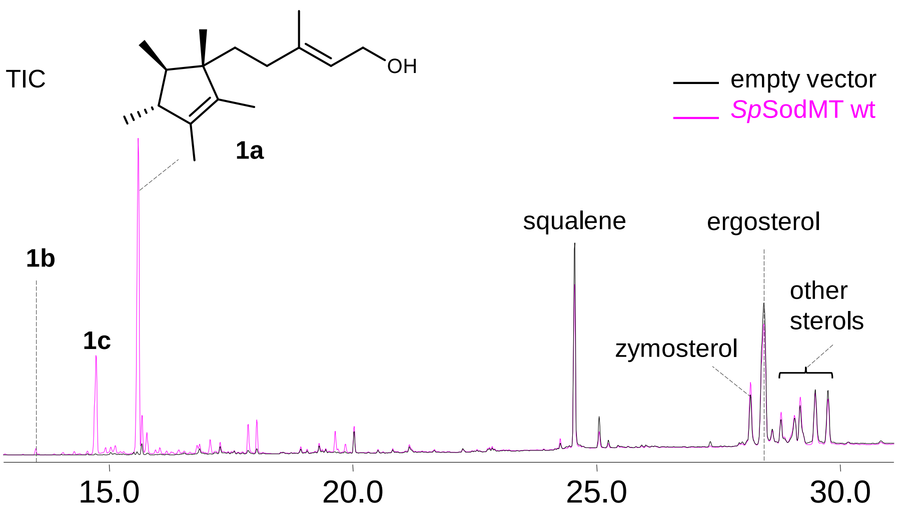

Supplementary Fig. 4. Sterol profile of AM109 yeast cells producing PSPP. Yeast cells expressing *Sp*SodMT (pink) or empty vector (black) were evaluated for their sterol content. The media were removed and the cells were harvested and subjected to non-saponifiable lipid extraction, as described in the materials and methods section. We confirmed that PSPP was entirely channeled into the production PSPP-derived compounds (**1a**, **1b** and **1c**) and not used by endogenous enzymes involved in sterol pathway to produce new squalene or sterol-like molecules.

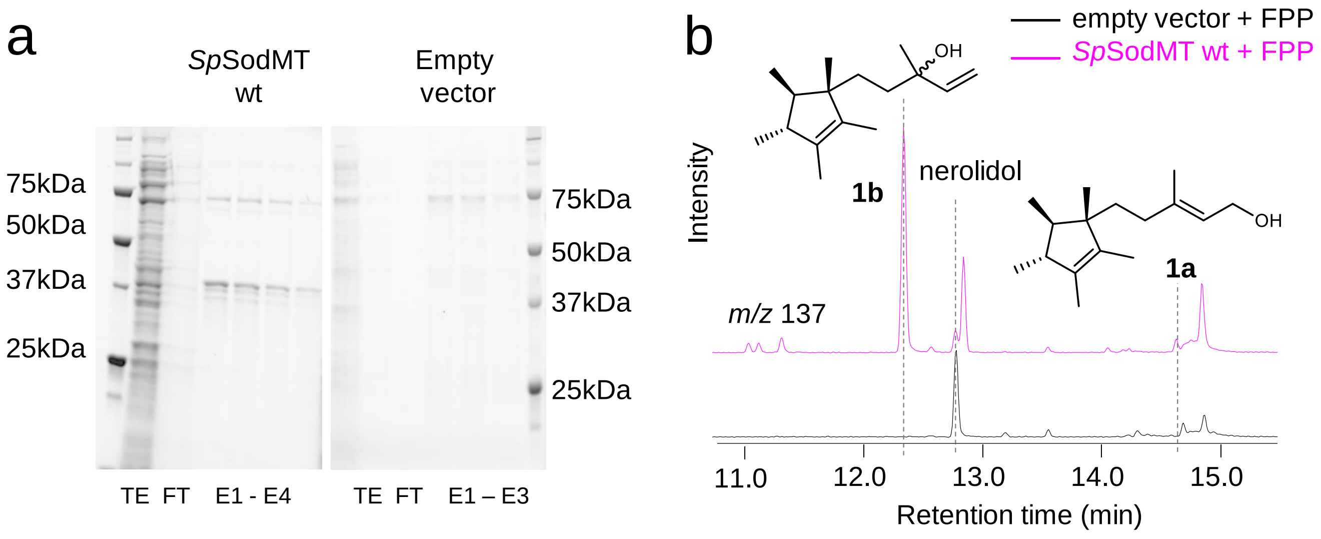

Supplementary Fig. 5. Evaluation of *Sp*SodMT enzymatic activity. **a.** SDS-PAGE of the recombinant *Sp*SodMT protein purified from *E. coli* was obtained and used in the in vitro assays. Protein containing a 6xHis tag has been purified by Ni^{2+} affinity chromatography. Total extract (TE) and three or four elutions (E1-E4 or E1-E3) from the column are shown for *Sp*SodMT and the empty vector sample, respectively. *Sp*SodMT protein purification was performed once. **b.** *Sp*SodMT enzymatic activity in an in vitro enzymatic assay using FPP and SAM as substrates. Acid hydrolysis was applied to detect the conversion products (**1b**) of PSPP synthesized by *Sp*SodMT. Small amount of nerolidol was also formed from conversion of excess of FPP substrate. In the absence of *Sp*SodMT (using elutions from lysates of cells transformed with the empty expression vector) the FPP substrate was entirely hydrolyzed to nerolidol. Characteristic chromatogram of experiment carried out in triplicate (n=3). Source data are provided as a Source Data file.

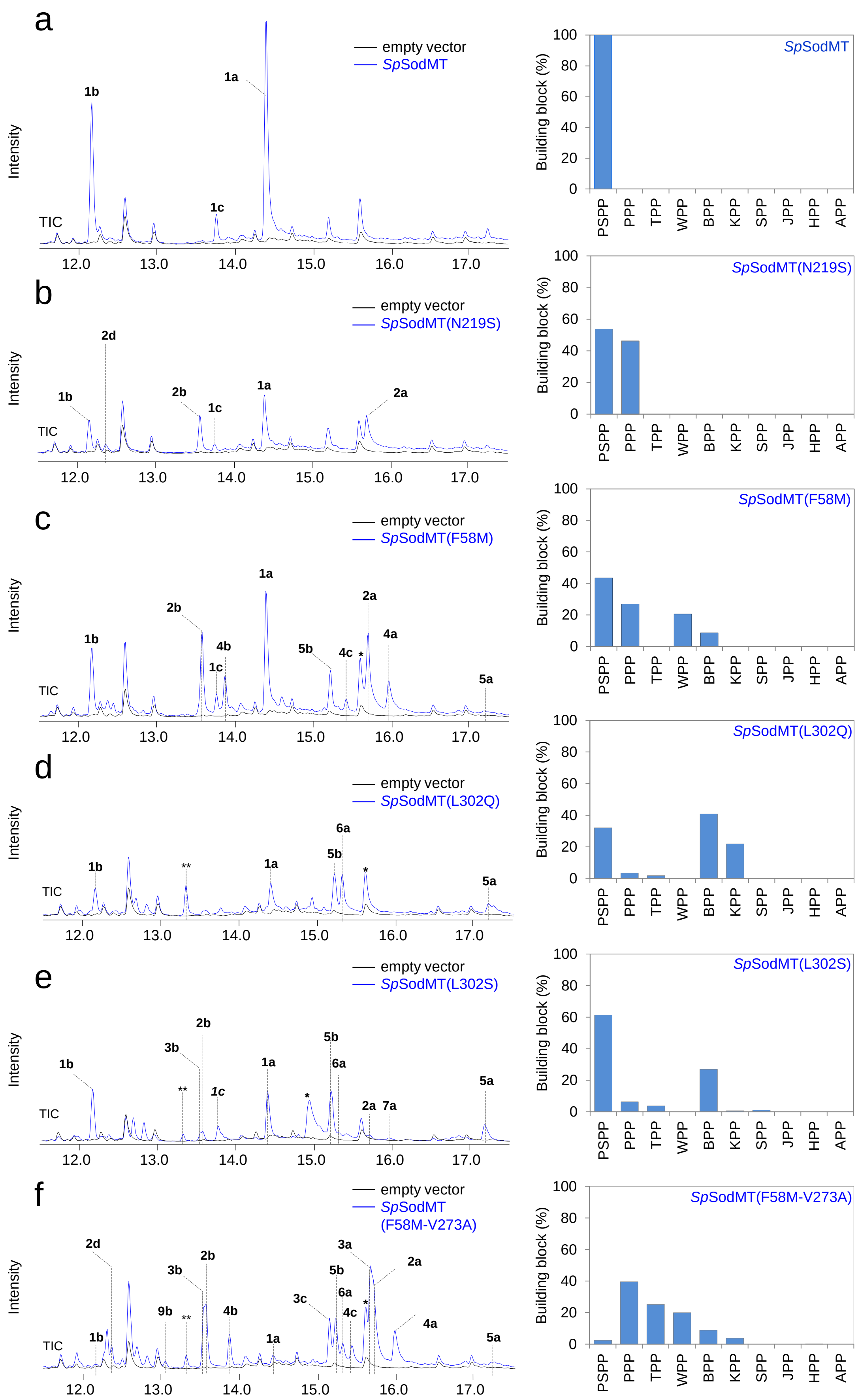

Please see figure legend in the next page

Supplementary Fig. 6. Synthesis of new C_{16} building blocks in yeast by *Sp*SodMT variants (continuation of Fig. 4). The product profile of different *Sp*SodMT variants (pink) using strains transformed with empty vector as control samples (black). Bar chart showing the C_{16} building block specificity of different SpSodMT variants (right). **a.** The profile of *Sp*SodMT wild-type showing the PSPP conversion products **1a**, **1b** and **1c** was used as reference to evaluate production of new C_{16} compounds in AM109 cells expressing different *Sp*SodMT variants. **b.** Chromatogram of *Sp*SodMT(N219S) showing production of compounds derived from PSPP (**1a**, **1b** and **1c**) and PPP (**2a, 2b** and **2d**). **c.** Chromatogram of *Sp*SodMT(F58M) showing a broad product profile. Peaks marked with * correspond to indole-containing compounds from yeast. **d.** Product profile of *Sp*SodMT(L302Q) in yeast showing preferential production of alcohols derived from buildings blocks BPP and KPP, while still producing PSPP-derived products. The peak indicated with "**" corresponds to an uncharacterized compound possibly derived from KPP, as suggested by the observation that it is present only in samples where **6a** is also present. The area of this peak was not included in the calculation of the product profile of *Sp*SodMT(L302Q) or the other *Sp*SodMT mutants. **e.** In cells expressing variant *Sp*SodMT(L302S), the alcohols resulting from conversion of PSPP (**1a**, **1b** and **1c**) still represented the majority of total C_{16} production. **f.** *Sp*SodMT(F58M-V273A) variant showing a broad profile of C_{16} products. Samples were analyzed in triplicate (n=3 biological replicates) and the mean value of the sum of products corresponding to each C_{16} diphosphate is shown. Source data provided as a Source Data file.

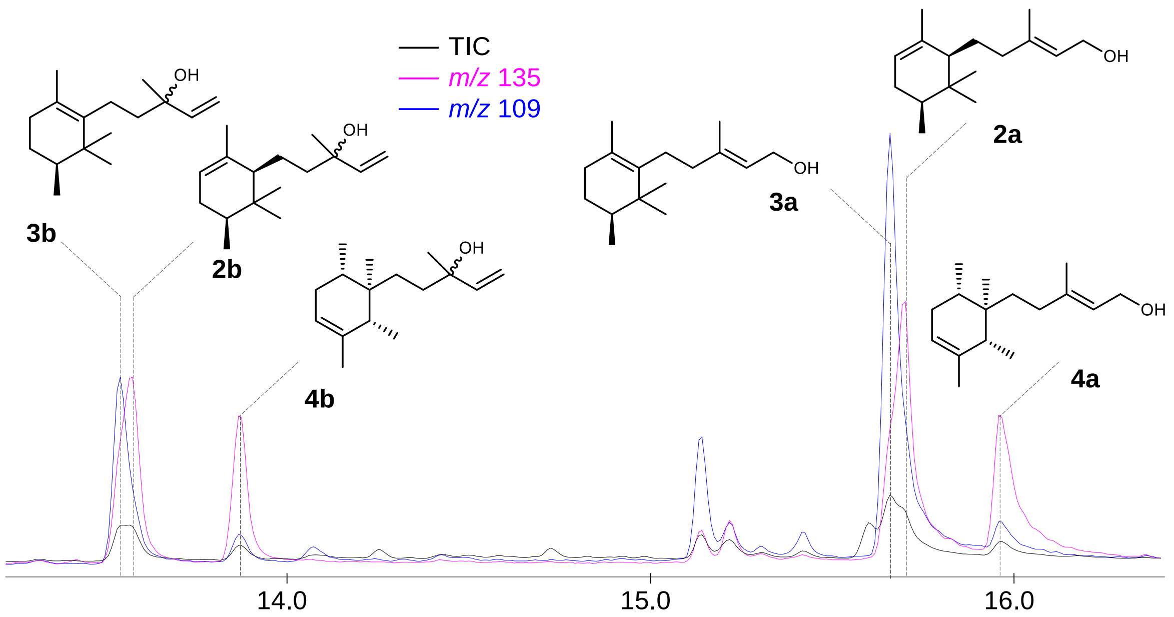

Supplementary Fig. 7. Chromatogram of *Sp*SodMT(F58L) product profile. Detection of PPP-, TPP- and WPP-derived products is shown in total ion count mode (TIC; black line) or by selecting for specific ions, *m/z* 135 (pink) for conversion products of PPP (**2a** and **2b**) and TPP (**3a** and **3b**) or *m/z* 109 (blue) for conversion products of WPP (**4a** and **4b**).

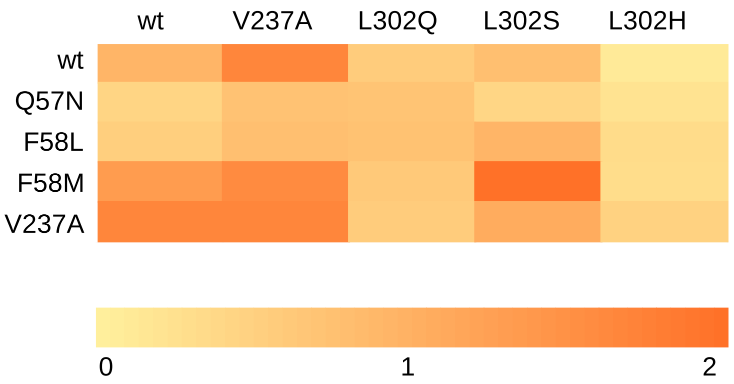

Supplementary Fig. 8. Heat map representing the total C_{16} terpene production in engineered *Sp*SodMT double variants. The highest production was obtained in *Sp*SodMT(F58M-L302S) and *Sp*SodMT(F58M-V273A) variants, while mutants derived from *Sp*SodMT(L302H) the lowest amount of C_{16} compounds. C_{16} production titers for each mutant were normalized to the titer of *Sp*SodMT wild-type. Samples were analyzed in triplicate (n=3 biological replicates) and the mean value of total C_{16} production is shown. Source data provided as a Source Data file.

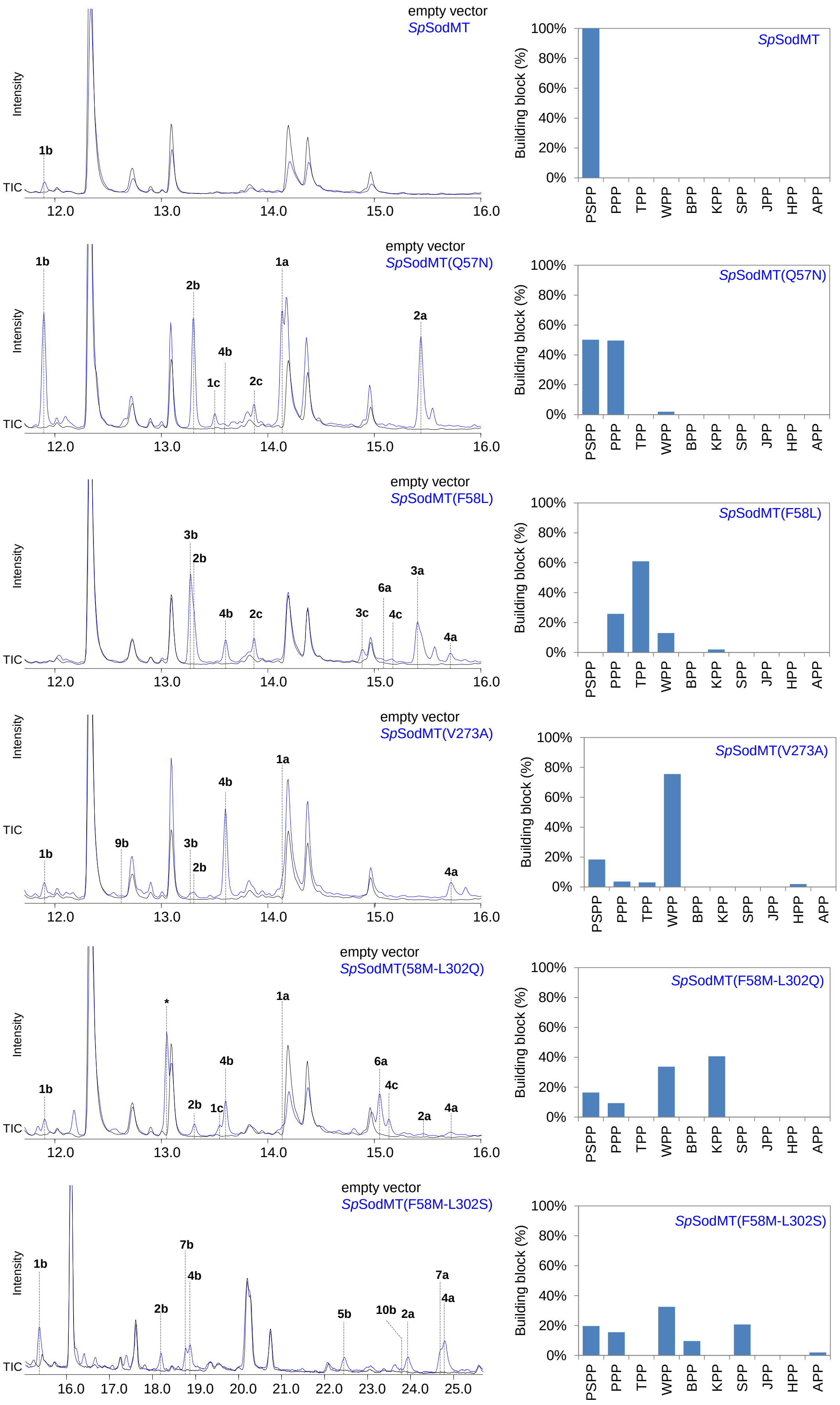

Please see figure legend in the next page

Supplementary Fig. 9. Activity assays of selected *Sp*SodMT variants with SAM and FPP as co-substrates. In vitro activity assays using extracts from yeast cells expressing *Sp*SodMT or selected variants. The assays were incubated at 30 °C for 3 h before termination by acid hydrolysis in combination with hexane extraction. The hexane was analyzed by GC-MS using analysis method 1 for *Sp*SodMT, *Sp*SodMT(Q57N), *Sp*SodMT(F58L), *Sp*SodMT(V273A), and *Sp*SodMT(58M-L302Q). GC-MS analysis method 2 was used for *Sp*SodMT(F58M-L302S). Only trace amounts of C_{16} peaks were present in control assays without FPP or SAM added, confirming that the products observed are not a remnant of the cell extract used. The corresponding bar charts show the percentage of products derived from each of the building blocks in the product profile. The peak areas of a, b, and c-forms of each building block were added together and the % of each building block was calculated. The peak indicated with "*" corresponds to an uncharacterized compound possibly derived from KPP, as suggested by the observation that it is present only in samples where **6a** is also present. The area of this peak was not included in the calculation of the product profile of the *Sp*SodMT mutants. Samples were analyzed in duplicate (n=2 biological replicates). The data shown here are representative of one out of two biological replicates. Source data are provided as a Source Data file.

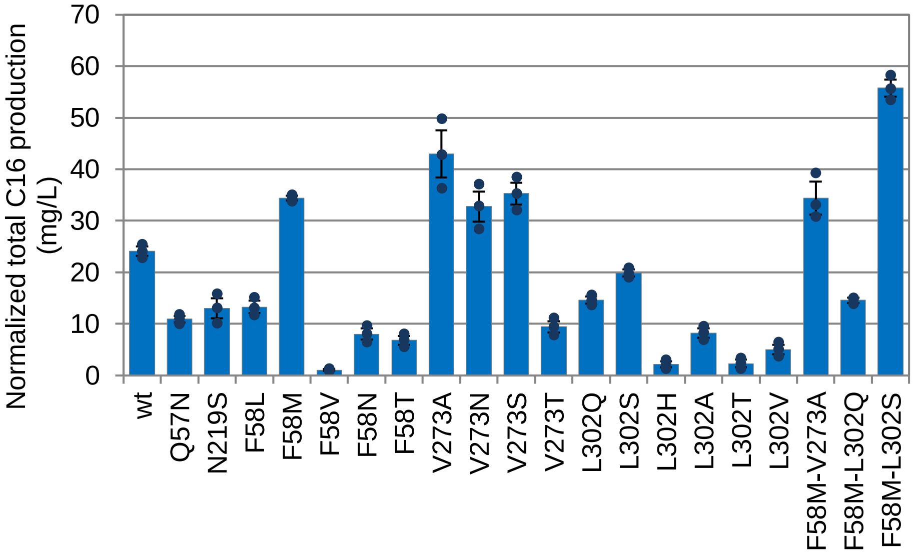

Supplementary Fig. 10. Production of C_{16} compounds by *Sp*SodMT wt and mutants. *Sp*SodMT wild-type and different variants were overexpressed in AM109 yeast cells and analyzed for production of C_{16} compounds. Total C_{16} production titers for each mutant were normalized to the C_{16} production titer of *Sp*SodMT wild type. Samples were analyzed in triplicate (n=3 biological replicates) and the mean value of total C_{16} production is shown. Errors correspond to the mean absolute deviation (MAD) around the mean. Source data provided as a Source Data file.

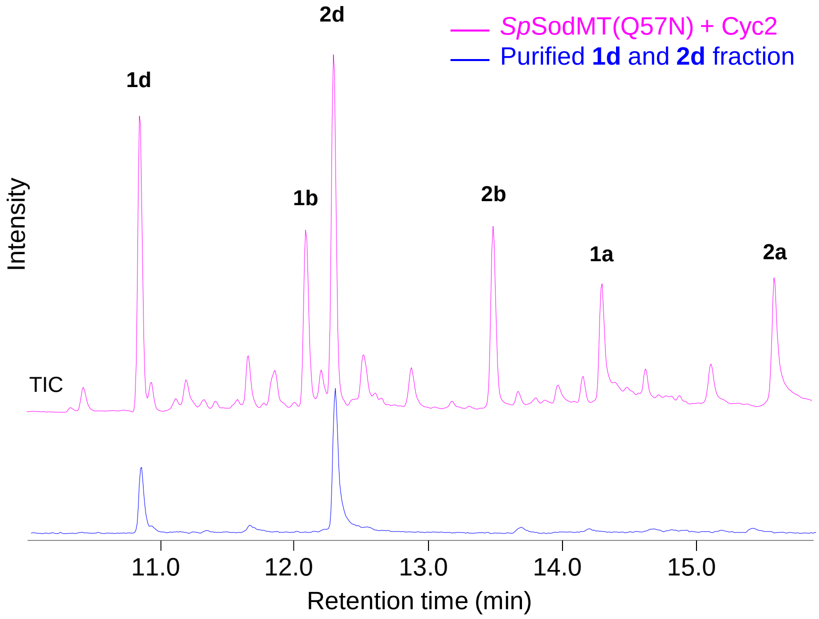

Supplementary Fig. 11. Production of C_{16} olefins by Cyc2 in yeast cells expressing *Sp*SodMT(Q57N). The product profile of yeast cells co-expressing Cyc2 and *Sp*SodMT(Q57N) was dominated by the C_{16} olefins **1d** and **2d**, which were identified using a mixture of the two purified compounds.

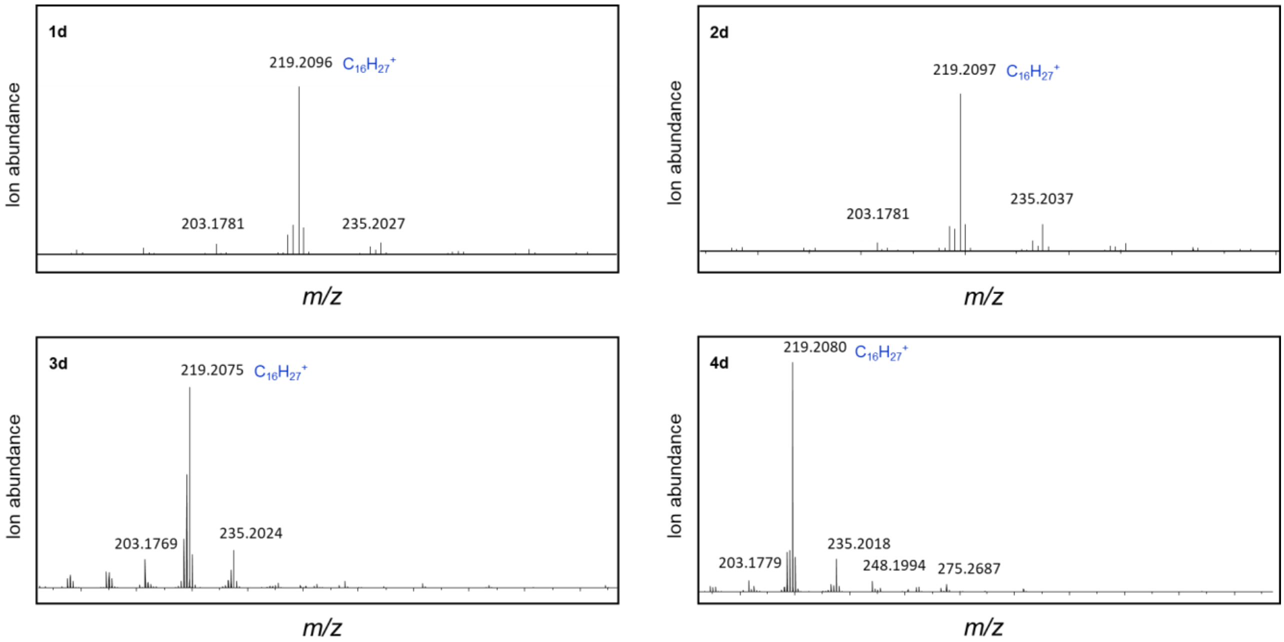

Supplementary Fig. 12. HR-APCI-GC-qTOF mass spectra of compounds **1d** and **2d** and of those tentatively assigned as **3d** and **4d**.

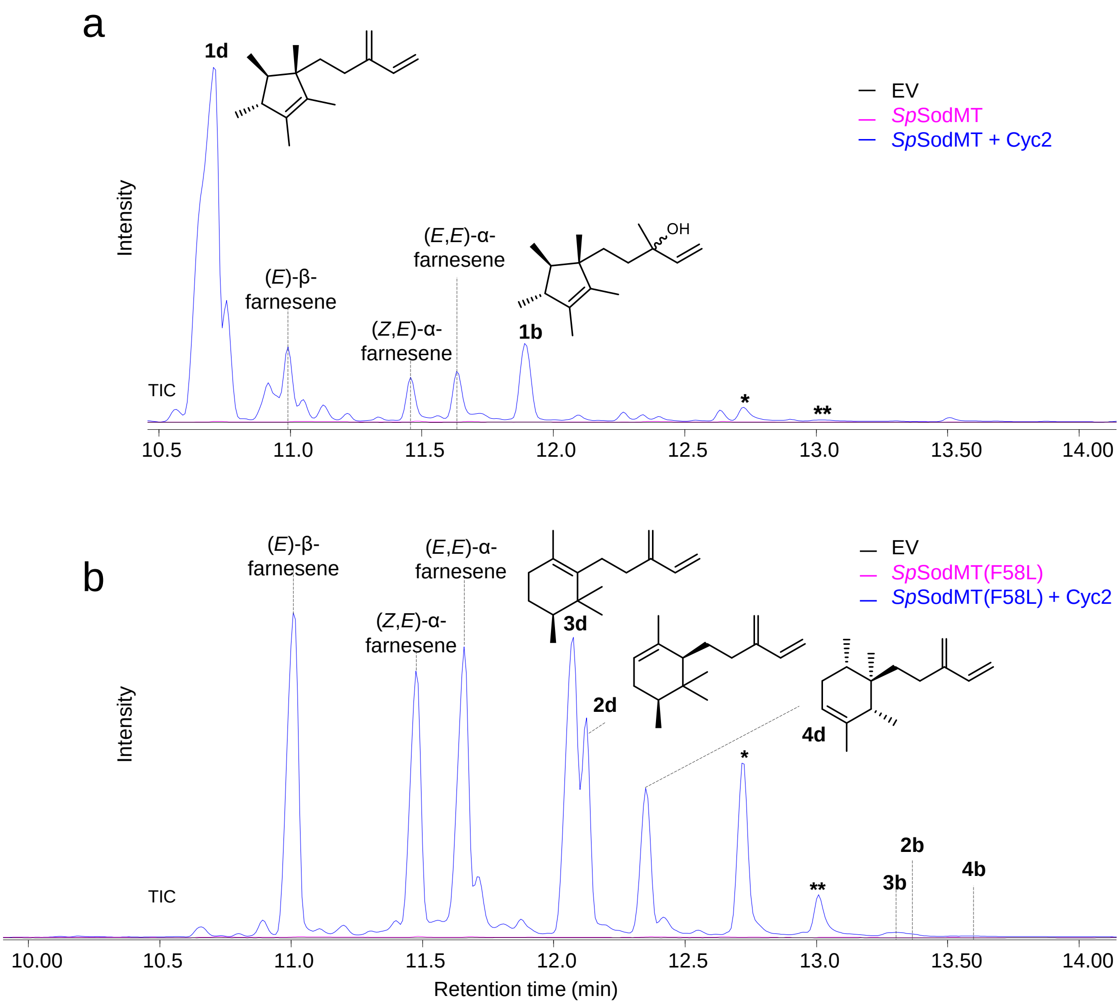

Supplementary Fig. 13. In vitro activity assays with *Sp*SodMT and Cyc2. In vitro assays using extracts from yeast cell co-expressing Cyc2 with **(a)** *Sp*SodMT or **(b)** *Sp*SodMT(F58L). The assays were incubated at 30 °C for 24 h, after which the headspace of the reaction vial was directly sampled by SPME fiber and analyzed by GC-MS analysis method 1 (see "Methods" section). Cyc2 mainly produces β-C_{16} olefins (**1d**, **2d**, **3d**, **4d**) from the C_{16} diphosphates. Cyc2 also converts the FPP added in the assay and makes a mix of farnesene isomers and two uncharacterized C_{15} compounds (denoted by * and **).

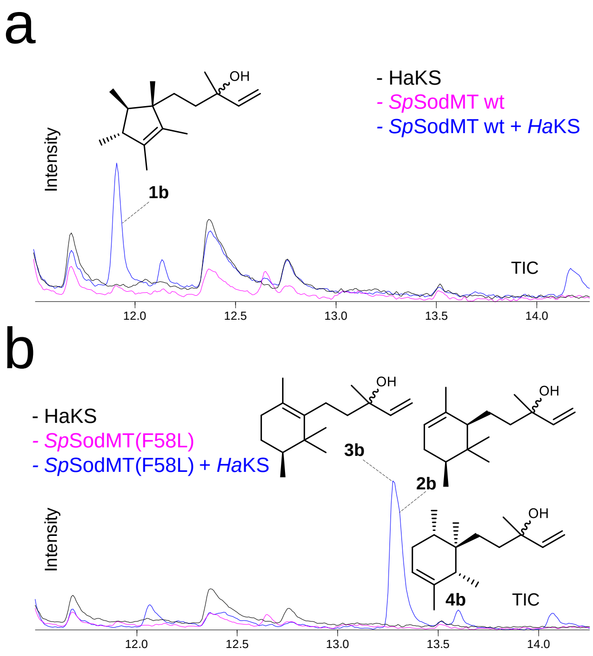

Supplementary Fig. 14. In vitro activity assay with *Sp*SodMT and *Ha*KS. In vitro assays using extracts from yeast cells co-expressing HaKS with **(a)** *Sp*SodMT or **(b)** *Sp*SodMT(F58L). The assays were incubated at 30 °C for 24 h, after which the headspace of the reaction vial was directly sampled by SPME fiber and analyzed by GC-MS analysis method 1 (see “Methods” section).

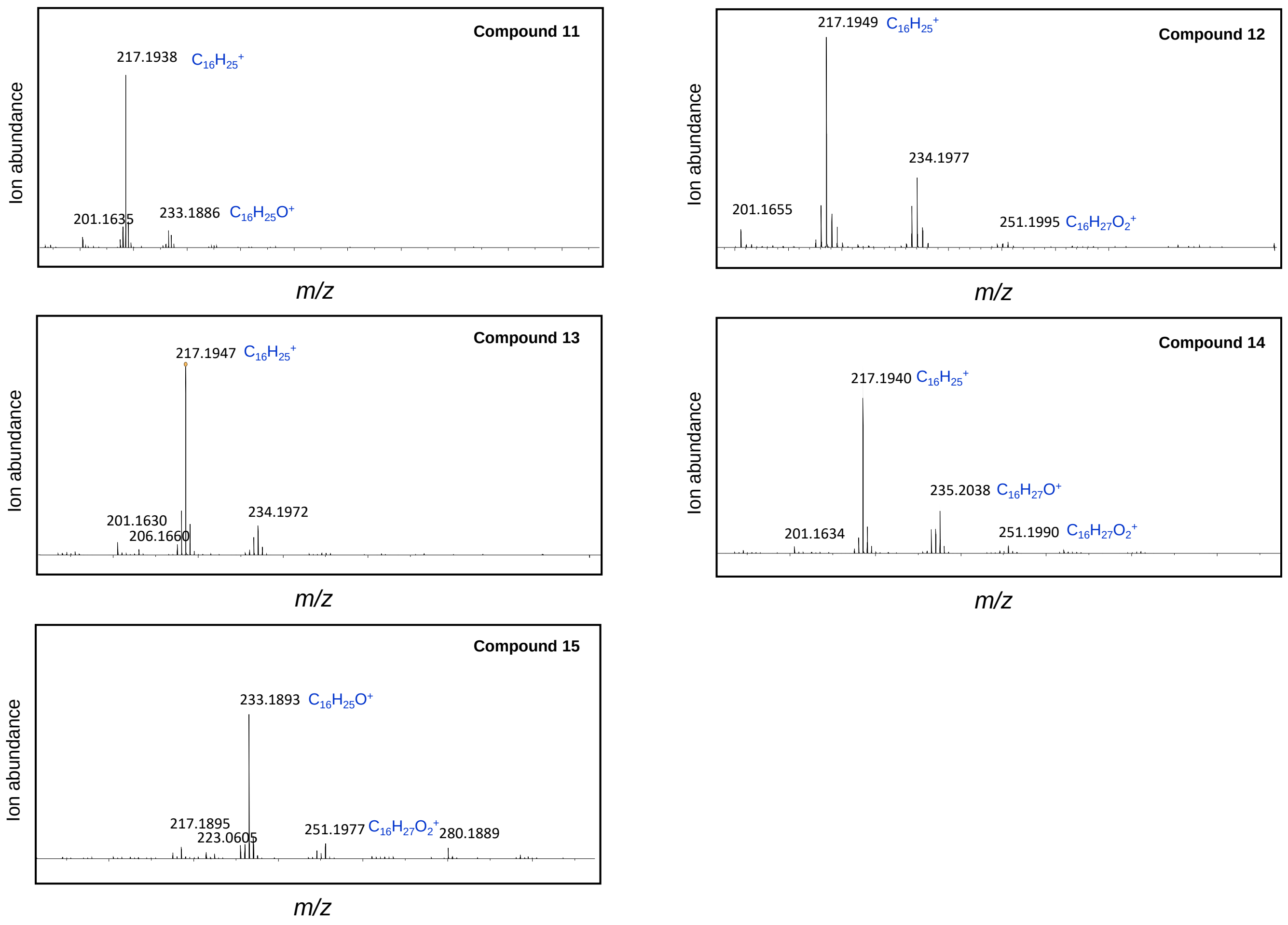

Supplementary Fig. 15. HR-APCI-GC-qTOF mass spectra of compounds **11-15**.

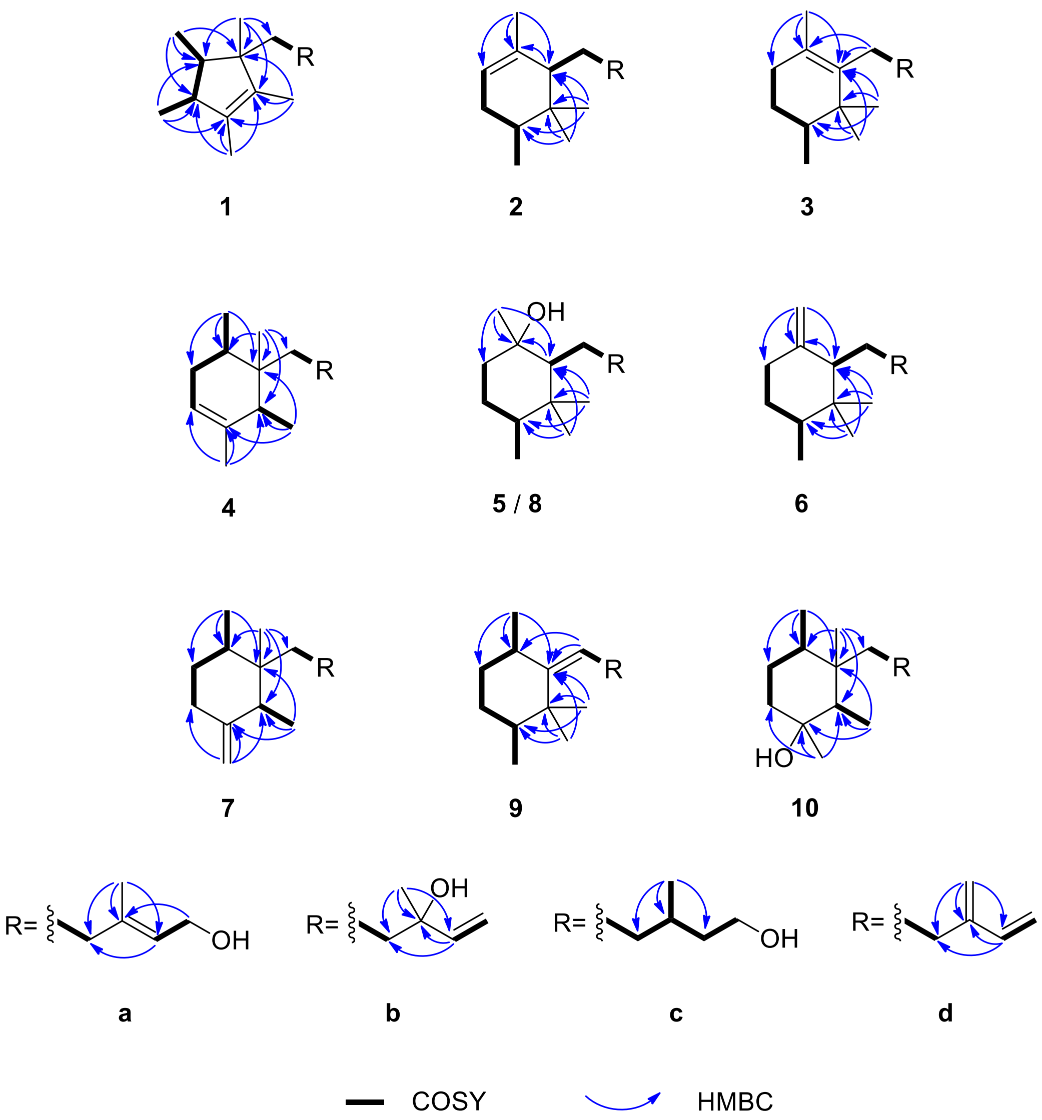

Supplementary Fig. 16. COSY and important HMBC correlations observed for building blocks **1**–**10** and side chains **a**–**d** constituting the isolated C_{16} compounds.

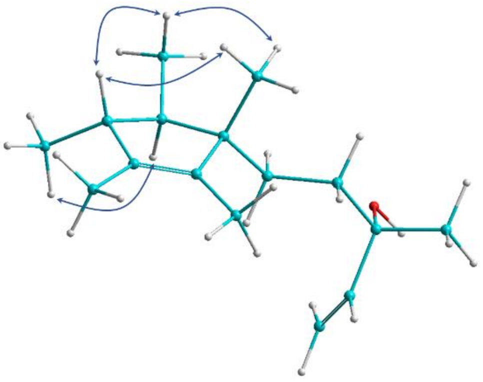

1b

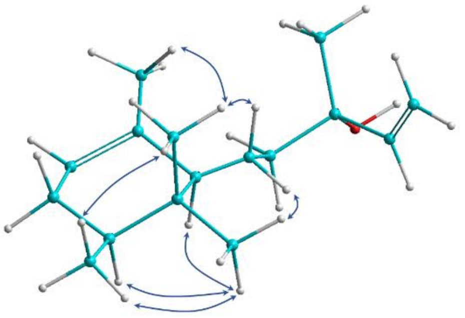

2b

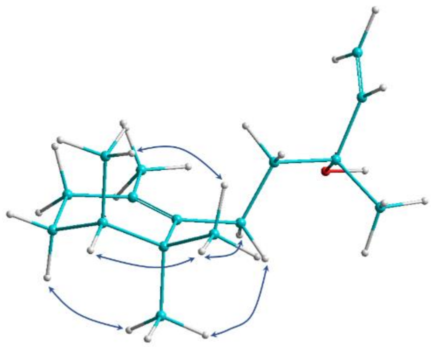

3b

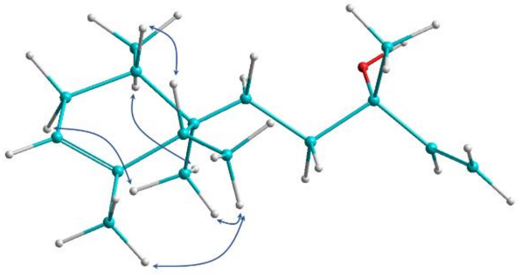

4b

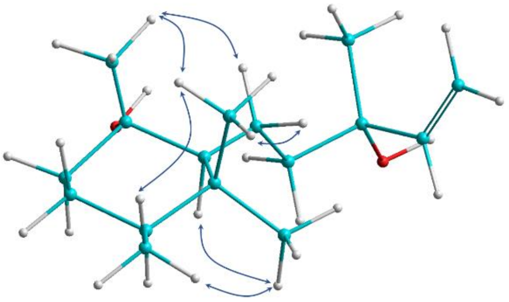

5b

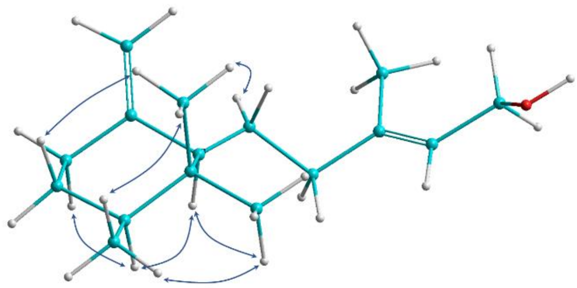

6a

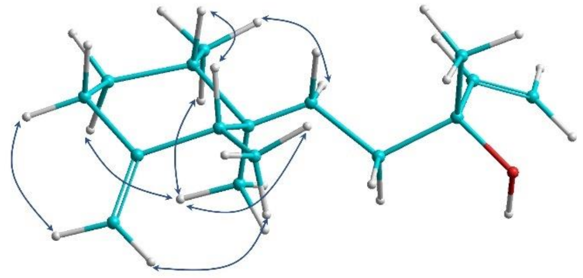

7b

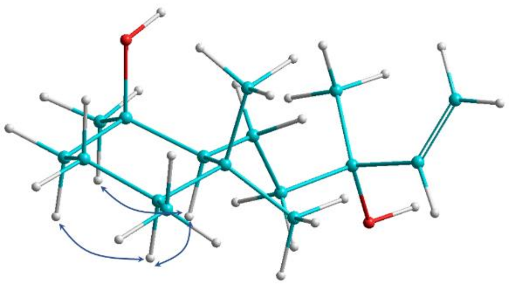

8b

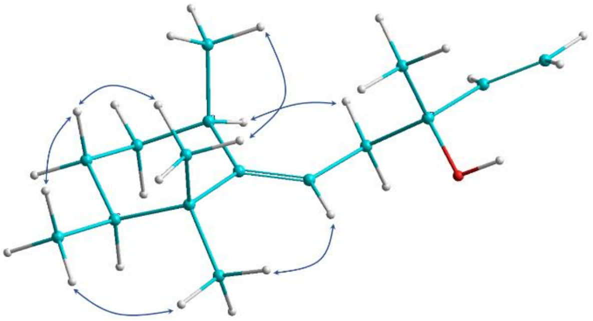

9b

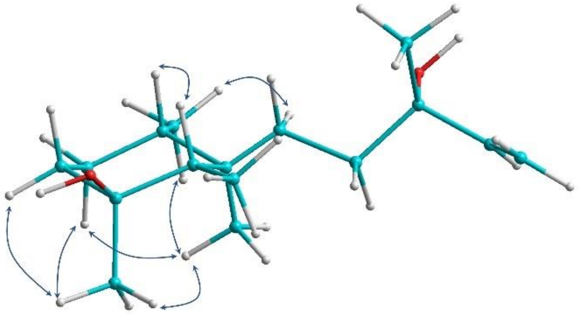

10b

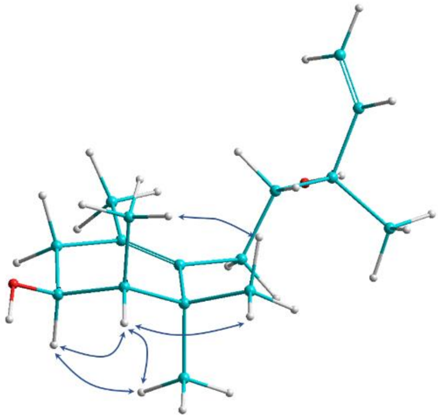

9OH-3b

Supplementary Fig. 17. Key NOE correlations used for the assignment of the relative configuration of building blocks **1**–**10**, as exemplified by representative examples.

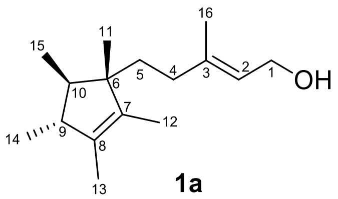

1a

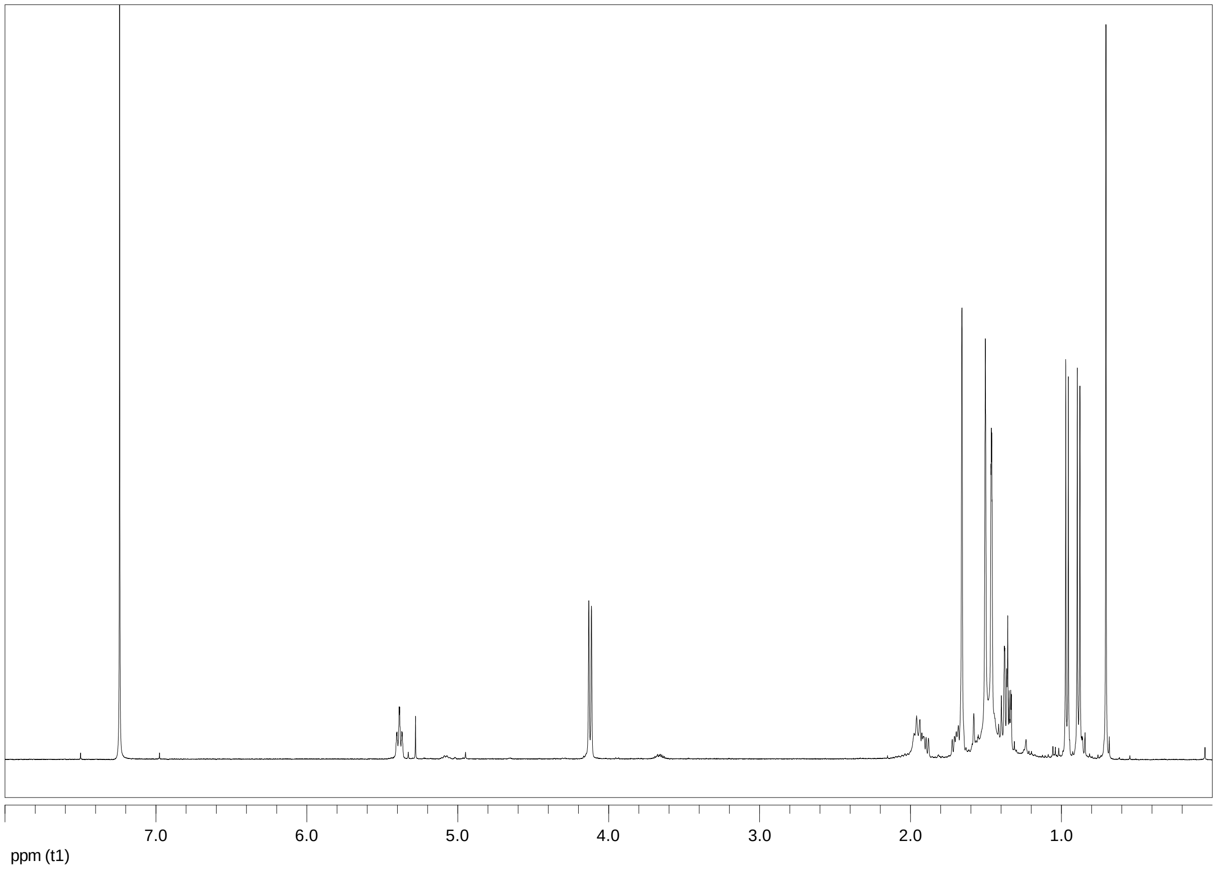

Supplementary Fig. 18. ^{1}H NMR spectrum (in $CDCl_3$) of compound **1a**.

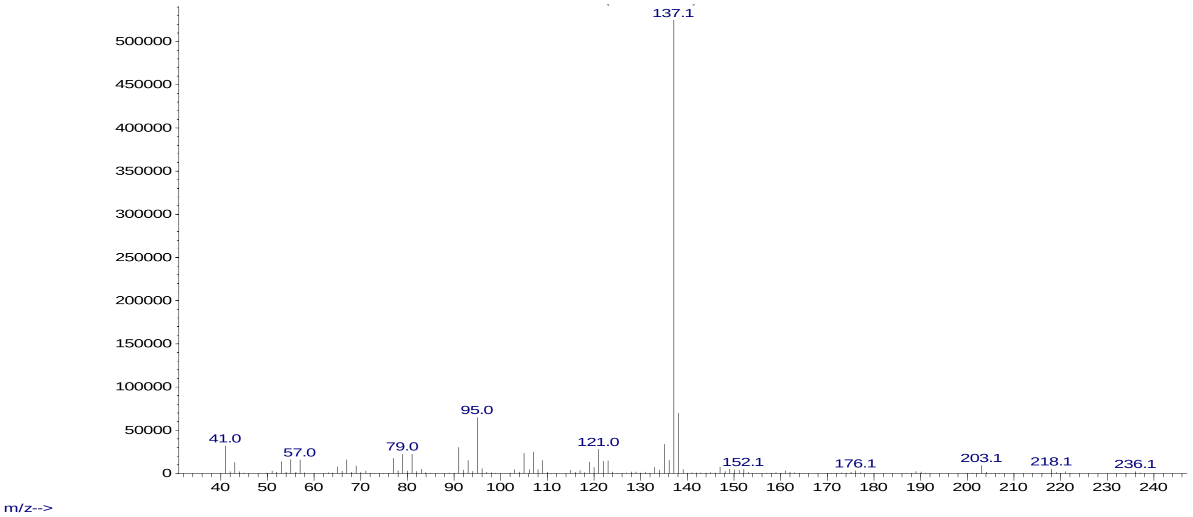

Supplementary Fig. 19. LR-EI-MS spectrum of compound **1a**.

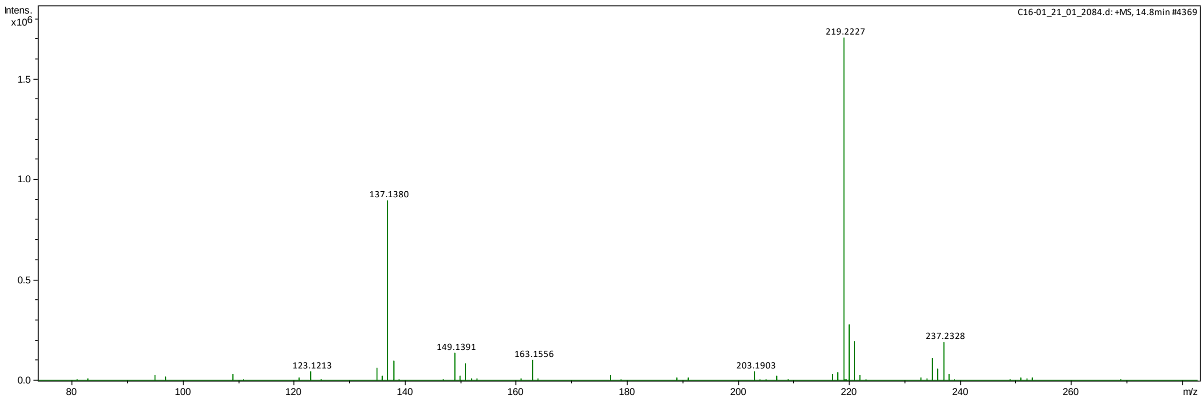

Supplementary Fig. 20. HR-APCI-MS spectrum of compound **1a**.

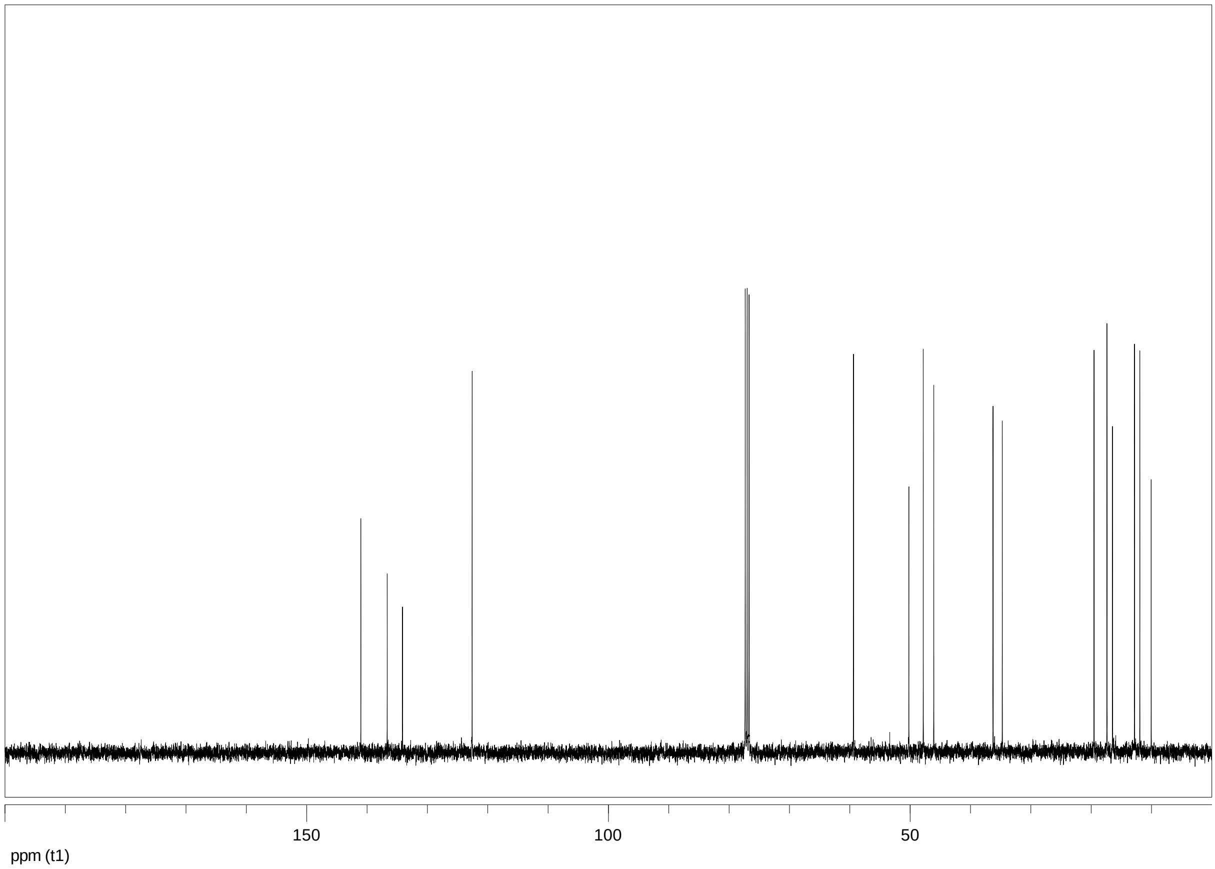

Supplementary Fig. 21. ^{13}C NMR spectrum (in $CDCl_3$) of compound **1a**.

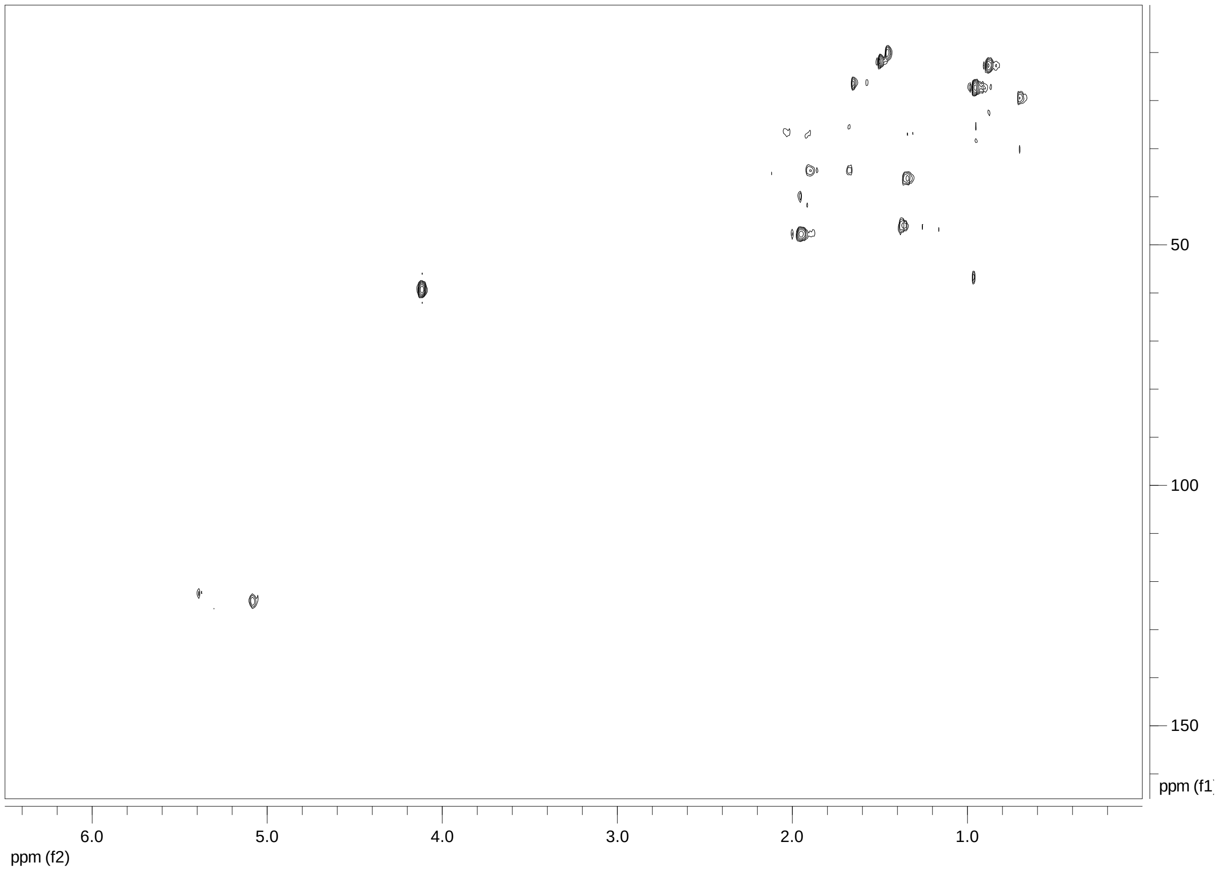

Supplementary Fig. 22. HSQC spectrum (in $CDCl_3$) of compound **1a**.

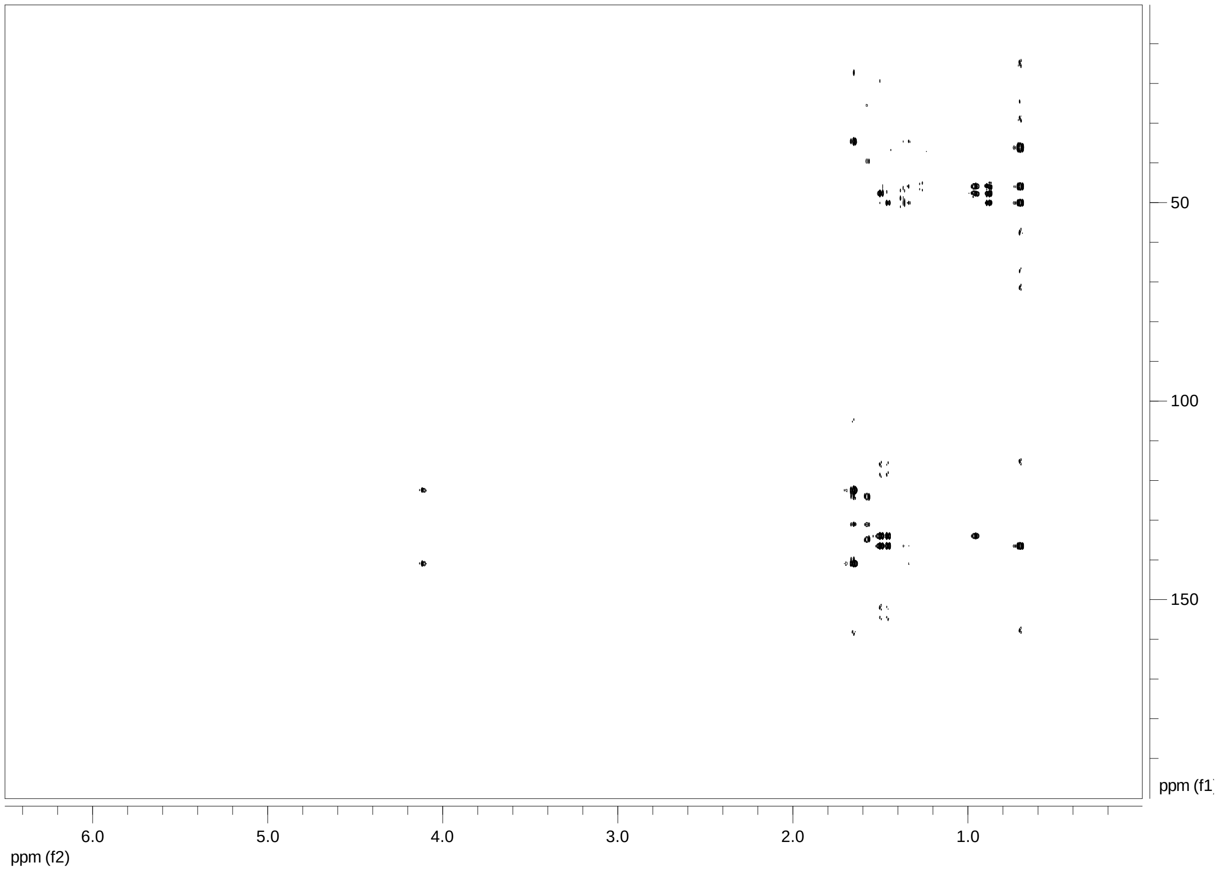

Supplementary Fig. 23. HMBC spectrum (in $CDCl_3$) of compound **1a**.

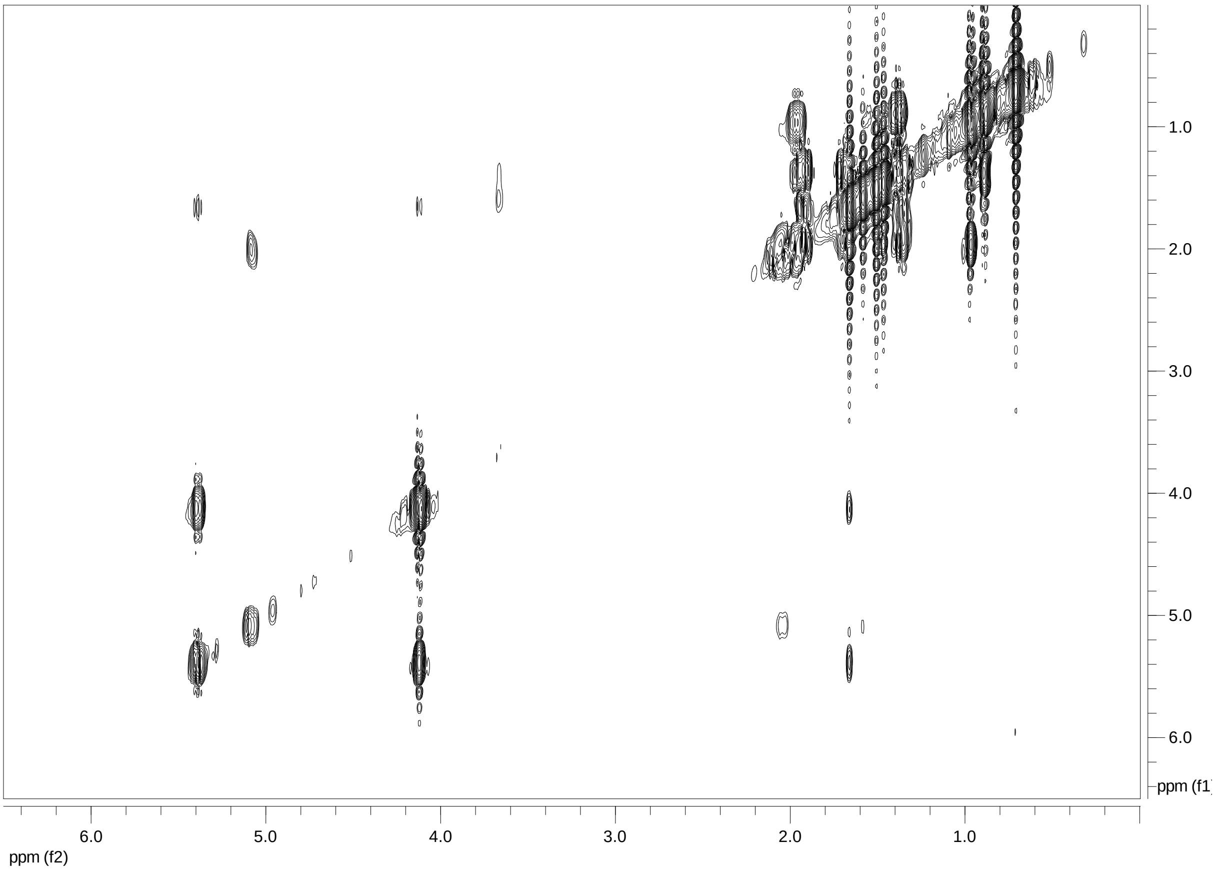

Supplementary Fig. 24. COSY spectrum (in $CDCl_3$) of compound **1a**.

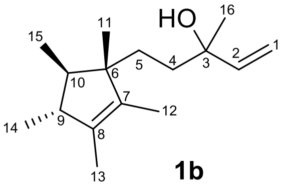

1b

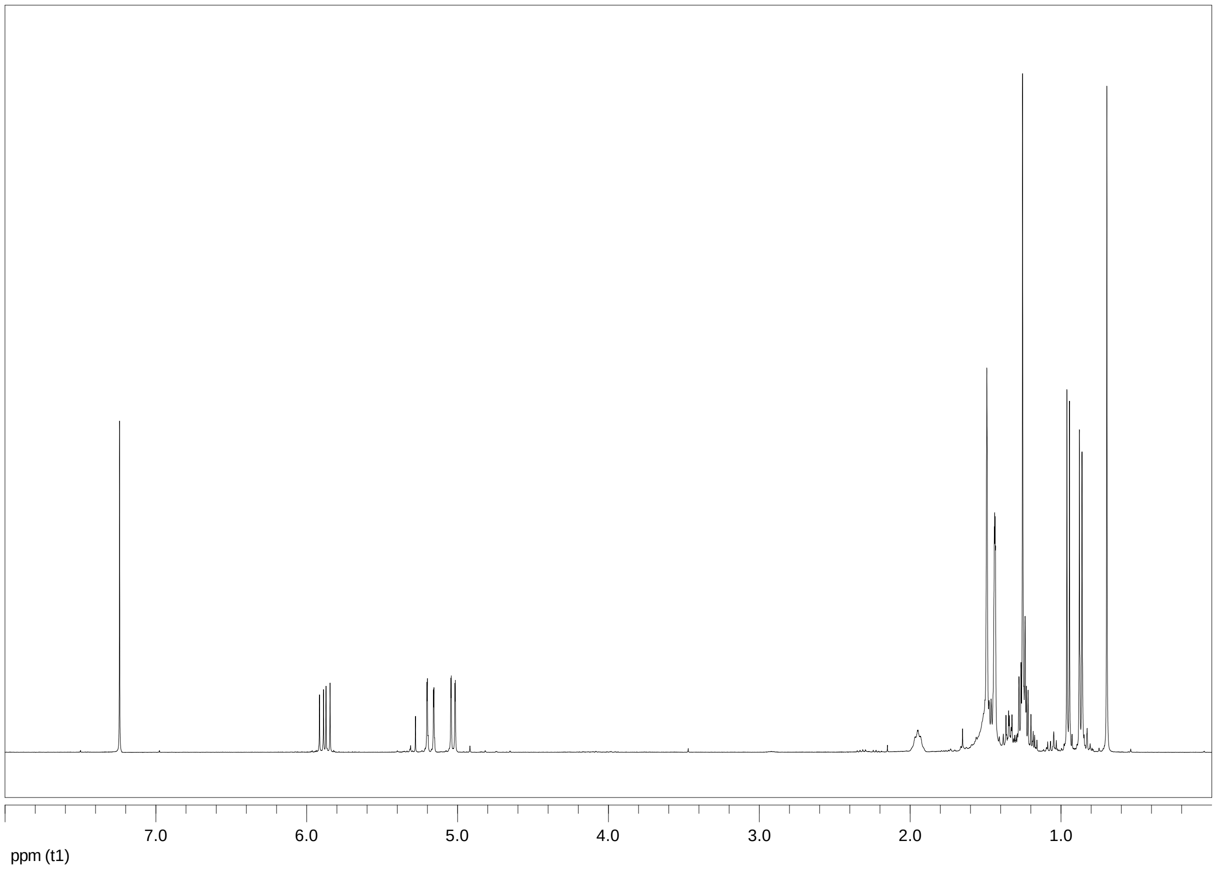

Supplementary Fig. 25. ^{1}H NMR spectrum (in $CDCl_3$) of compound **1b**.

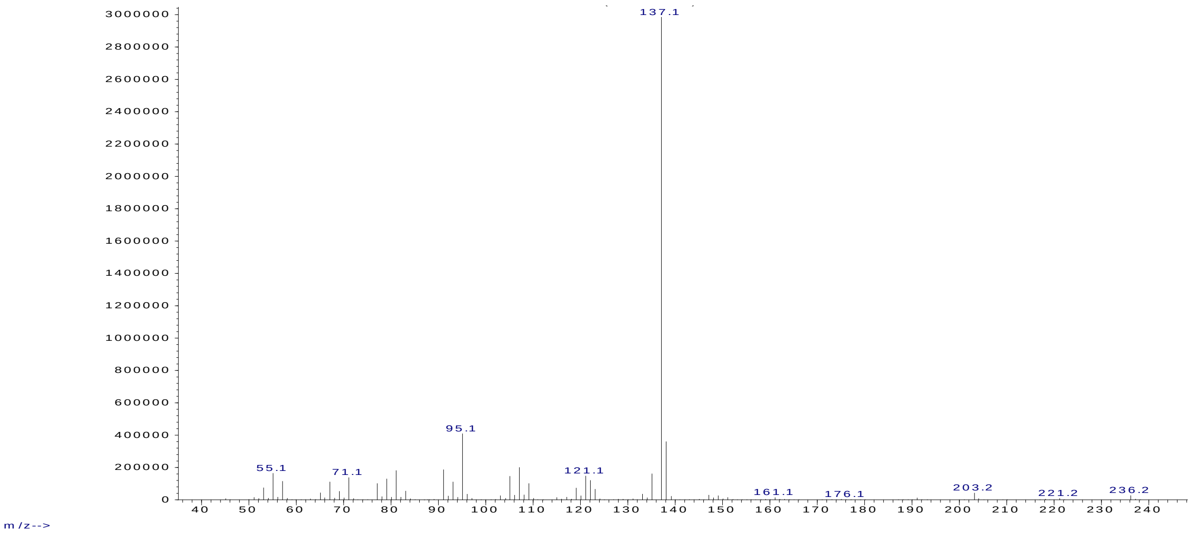

Supplementary Fig. 26. LR-EI-MS spectrum of compound **1b**.

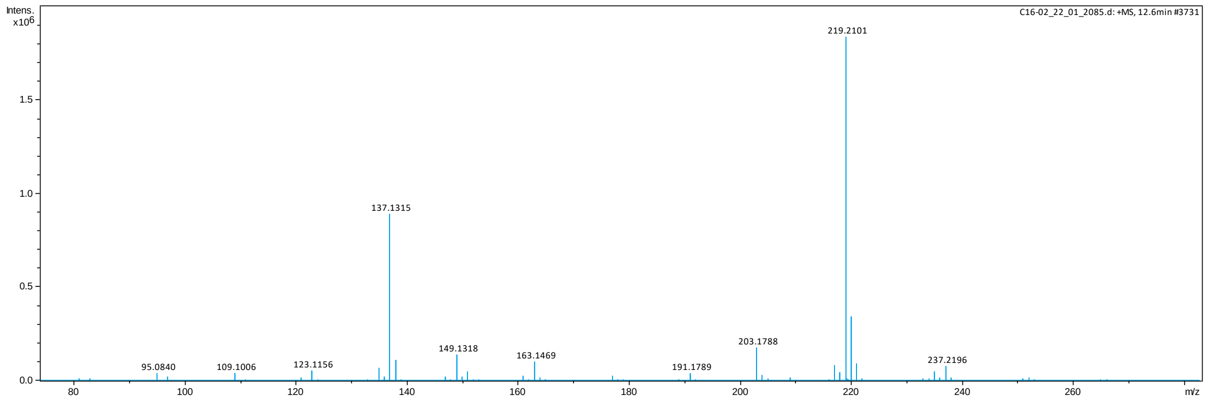

Supplementary Fig. 27. HR-APCI-MS spectrum of compound **1b**.

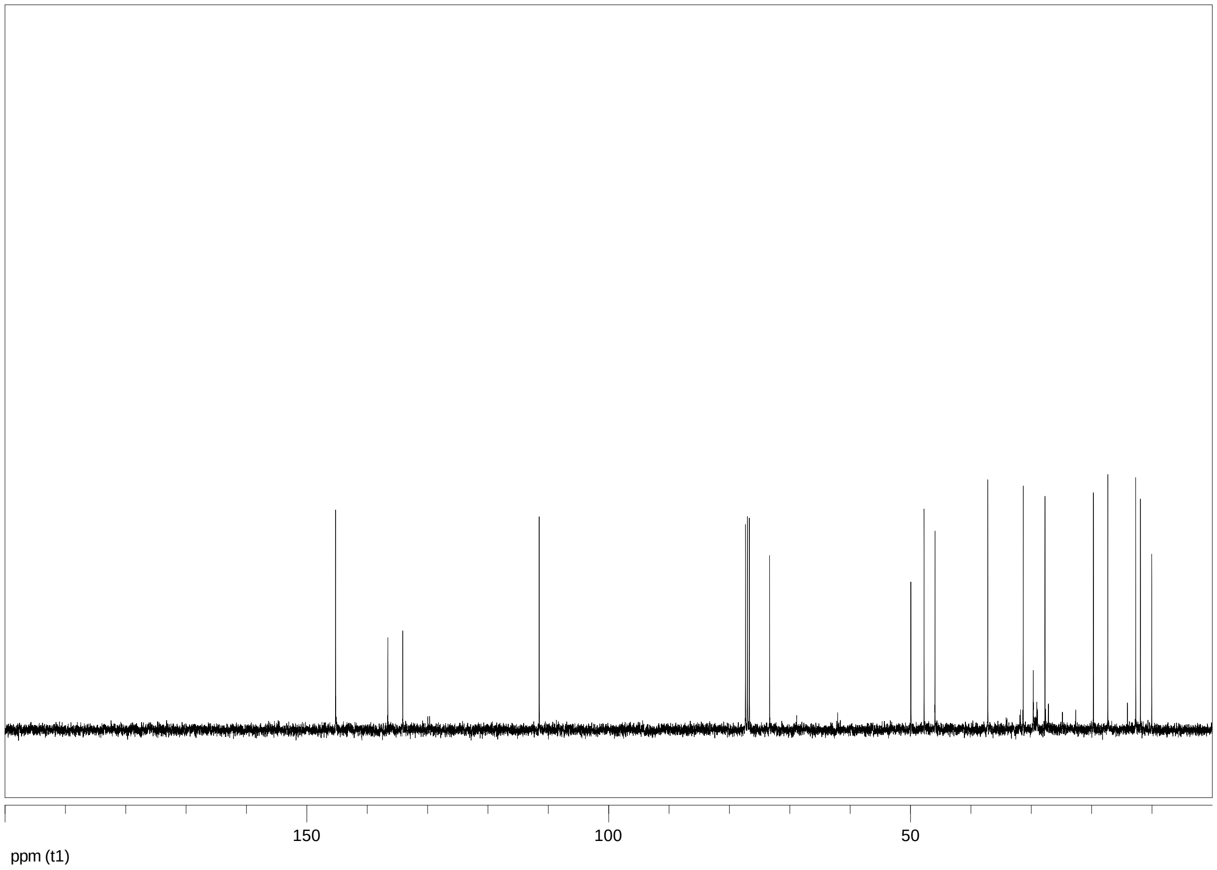

Supplementary Fig. 28. ^{13}C NMR spectrum (in $CDCl_3$) of compound **1b**.

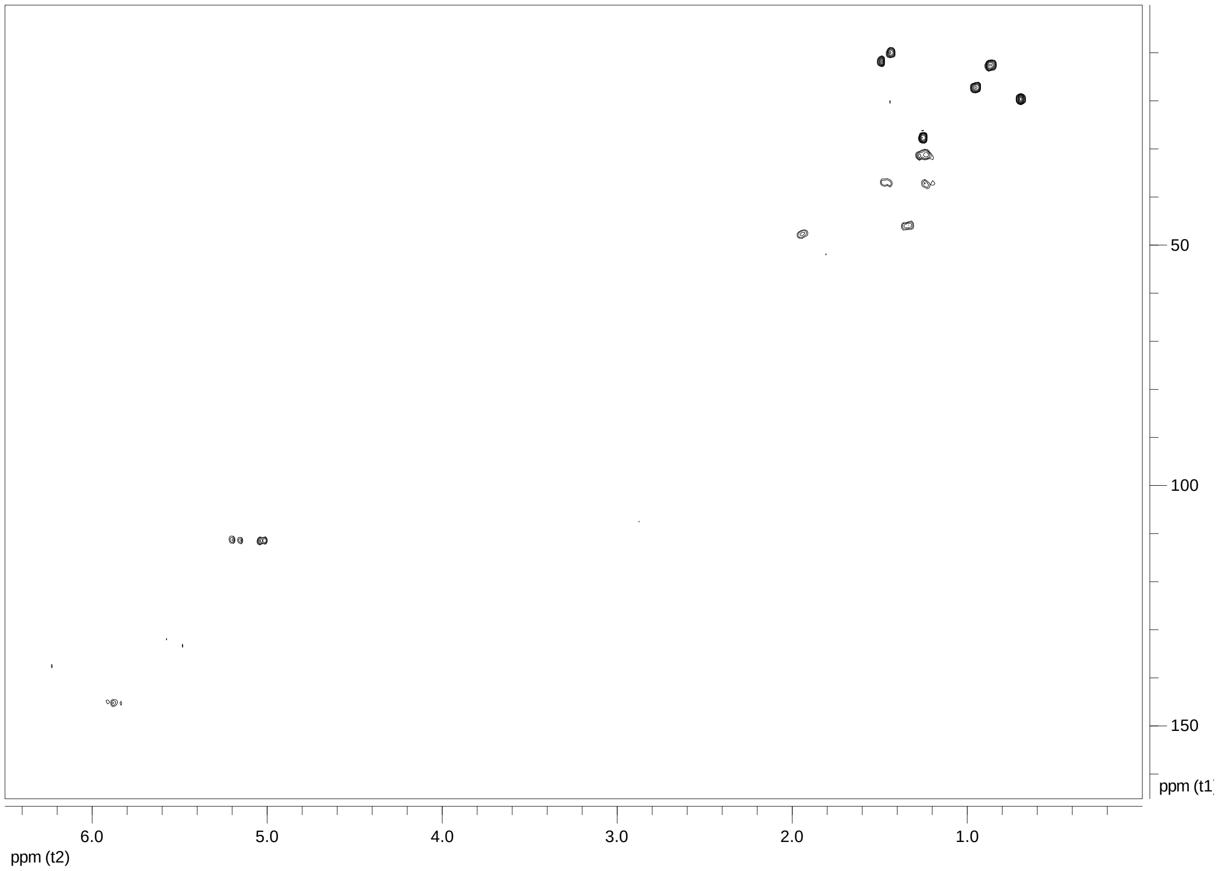

Supplementary Fig. 29. HSQC spectrum (in $CDCl_3$) of compound **1b**.

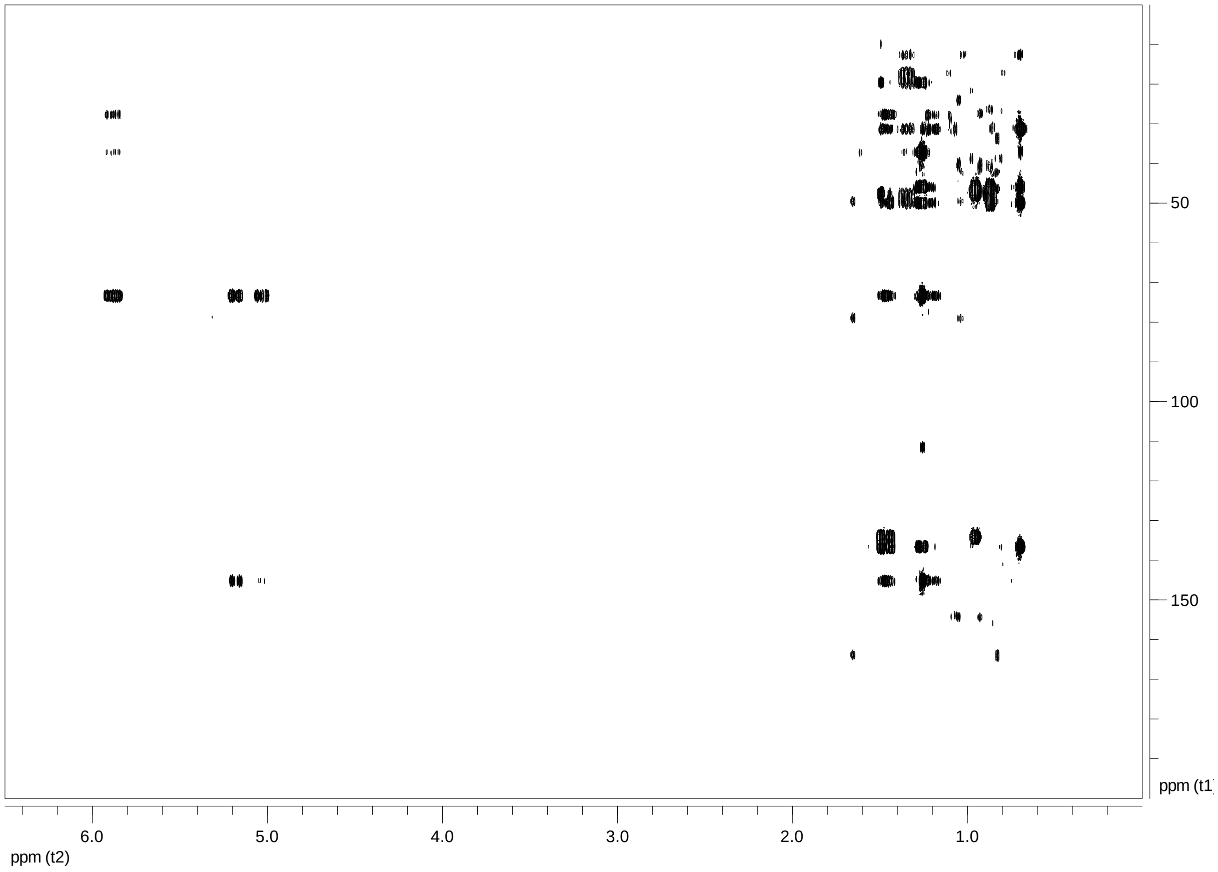

Supplementary Fig. 30. HMBC spectrum (in $CDCl_3$) of compound **1b**.

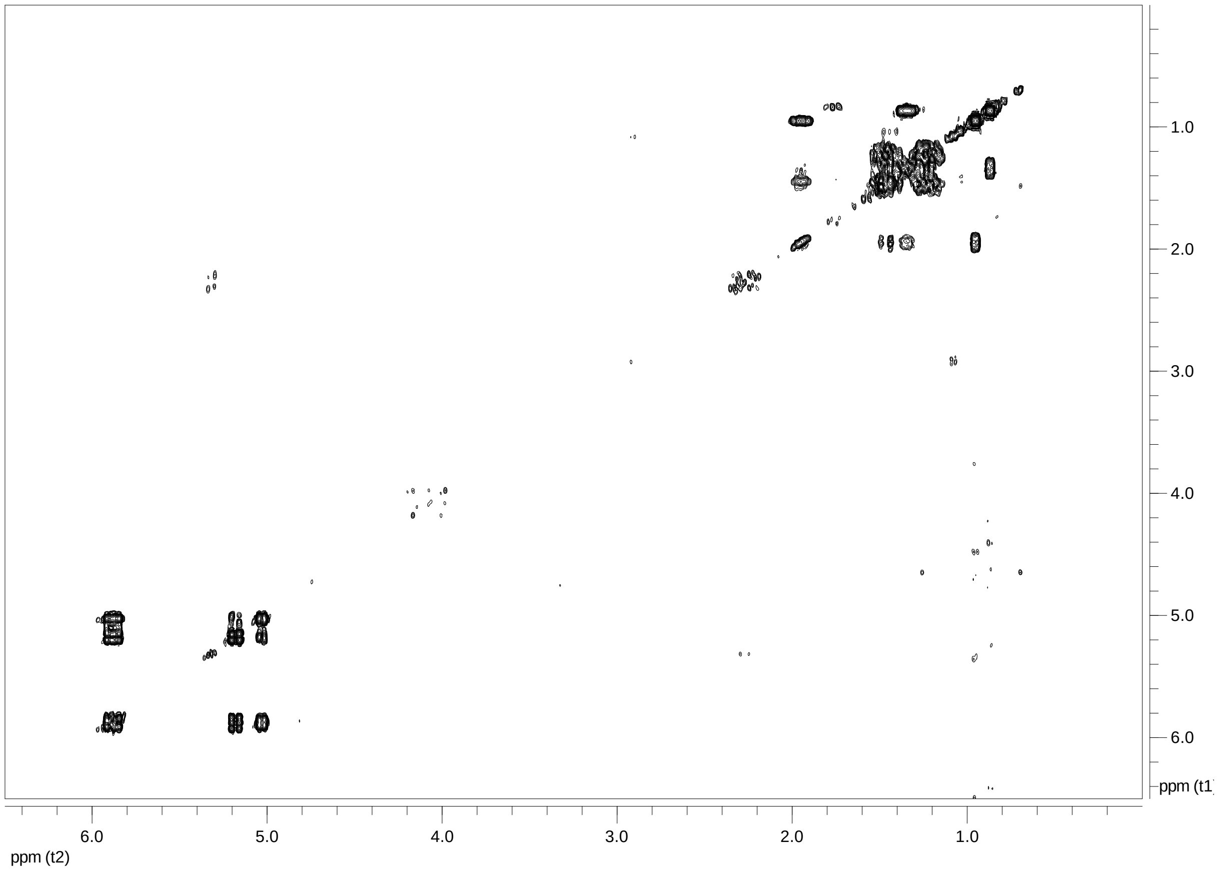

Supplementary Fig. 31. COSY spectrum (in $CDCl_3$) of compound **1b**.

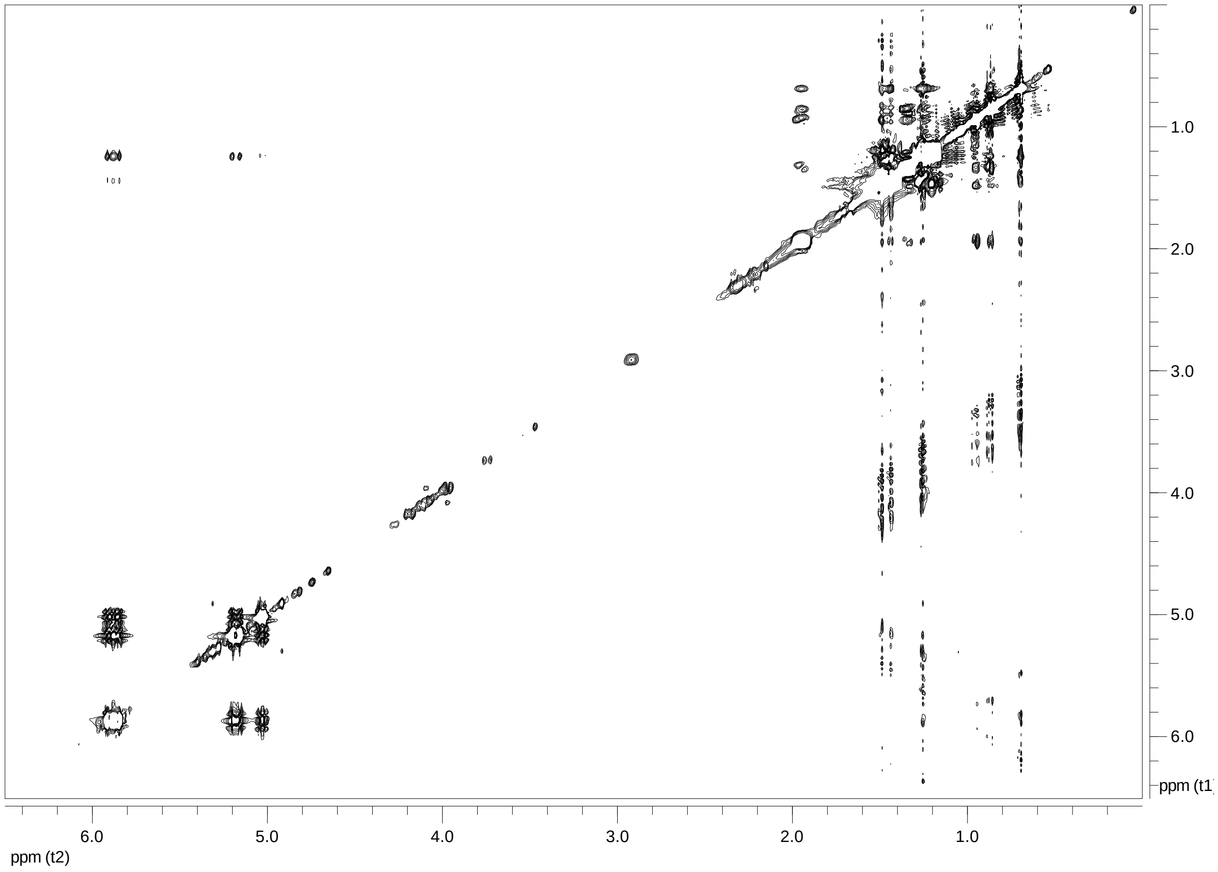

Supplementary Fig. 32. NOESY spectrum (in $CDCl_3$) of compound **1b**.

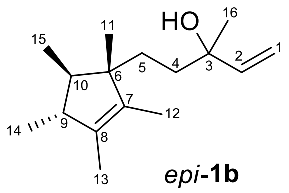

epi-**1b**

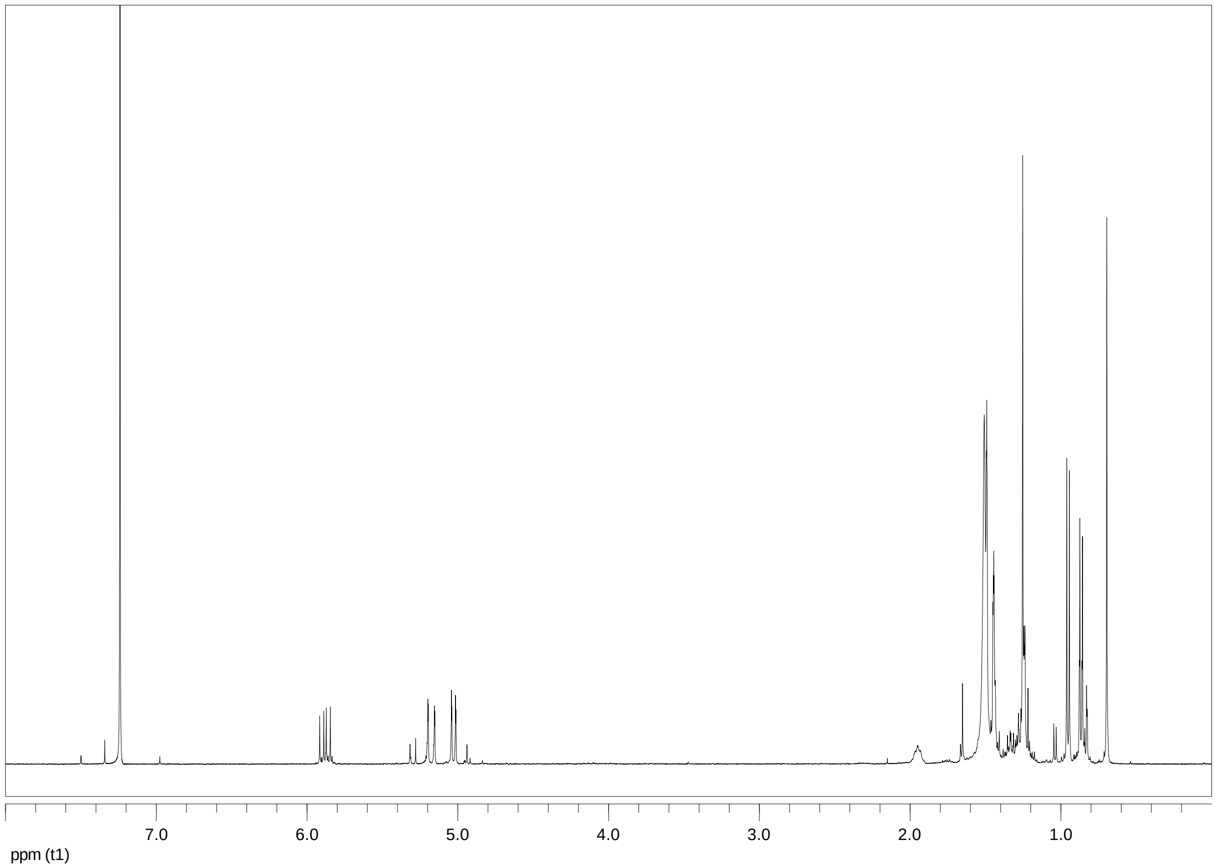

Supplementary Fig. 33. ^{1}H NMR spectrum (in $CDCl_3$) of compound ***epi*-1b**.

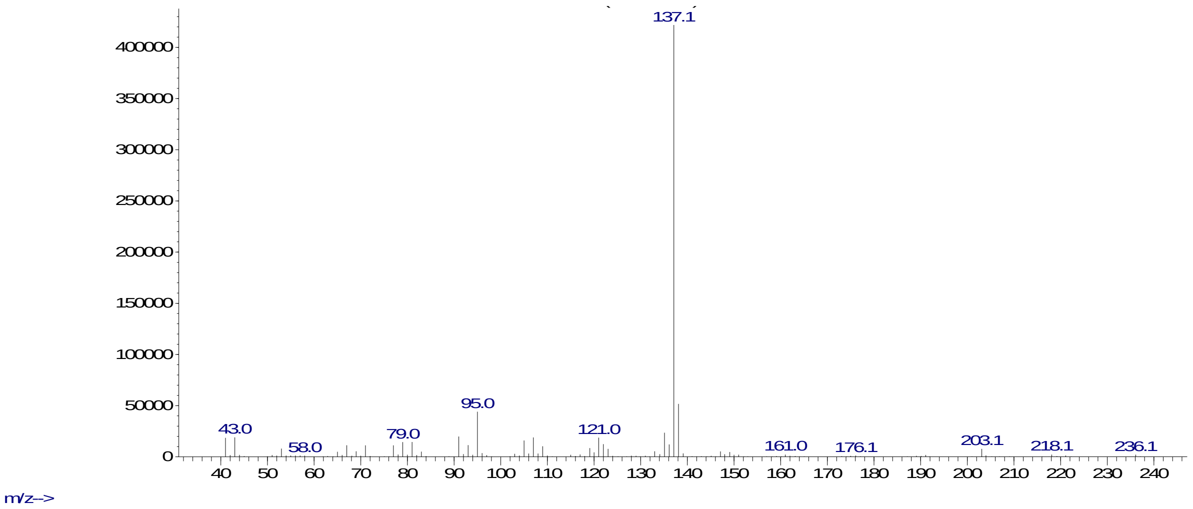

Supplementary Fig. 34. LR-EI-MS spectrum of compound ***epi*-1b**.

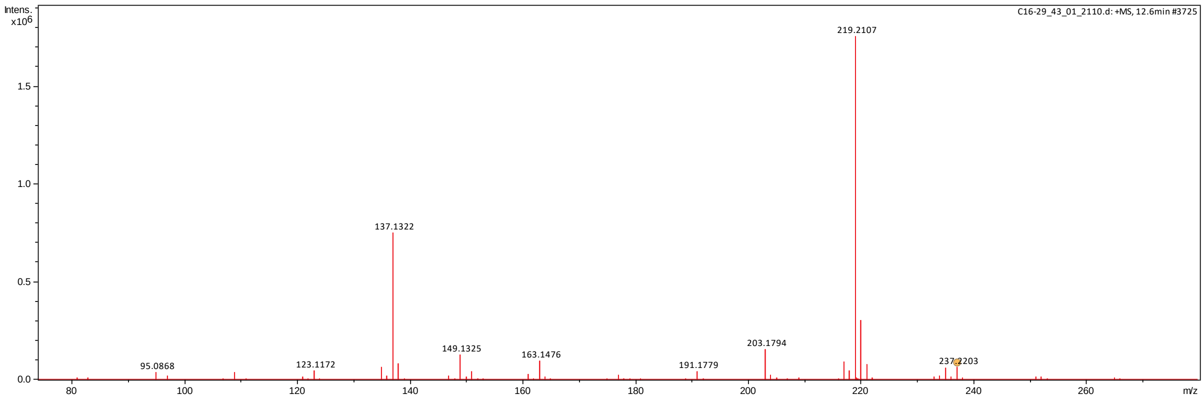

Supplementary Fig. 35. HR-APCI-MS spectrum of compound ***epi*-1b**.

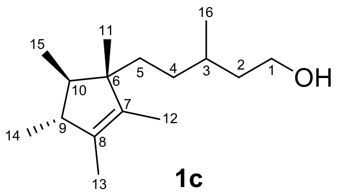

1c

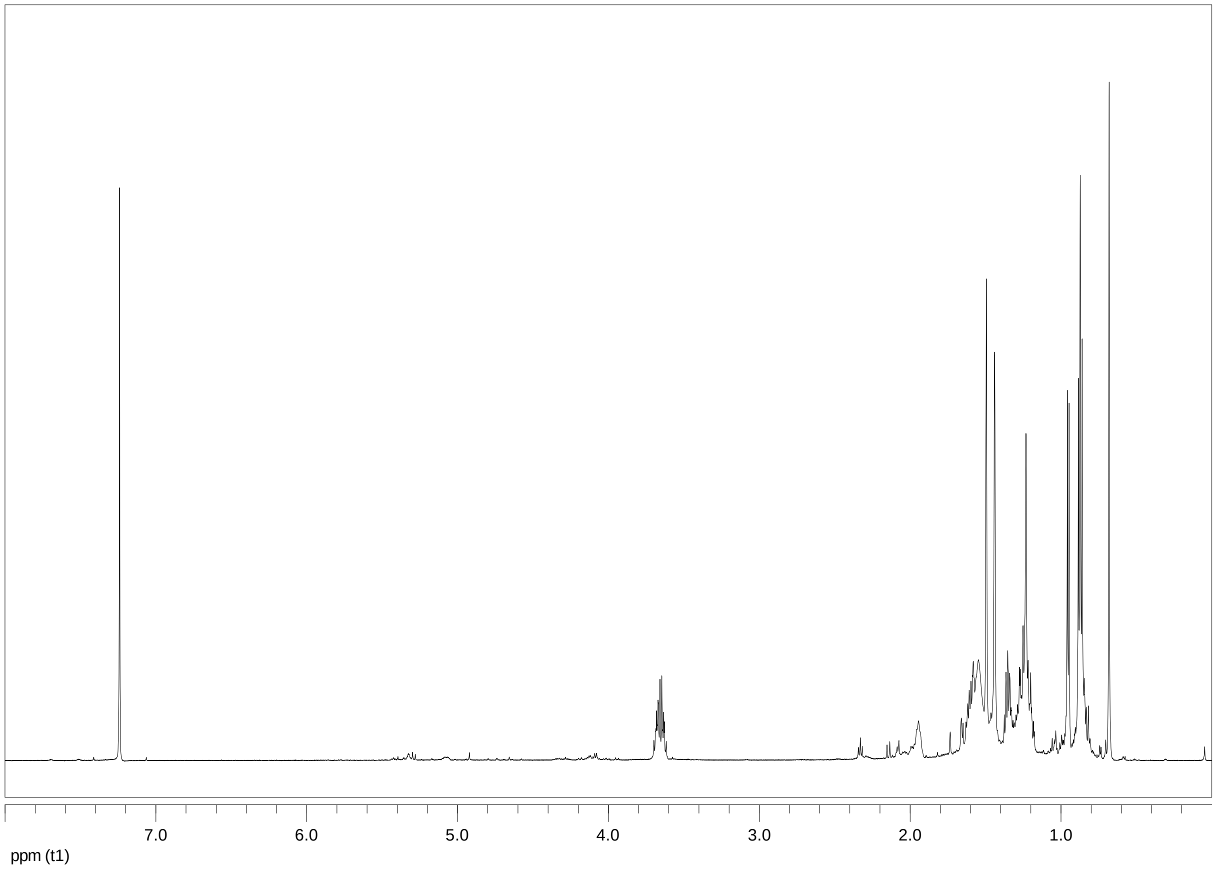

Supplementary Fig. 36. ^{1}H NMR spectrum (in $CDCl_3$) of compound **1c**.

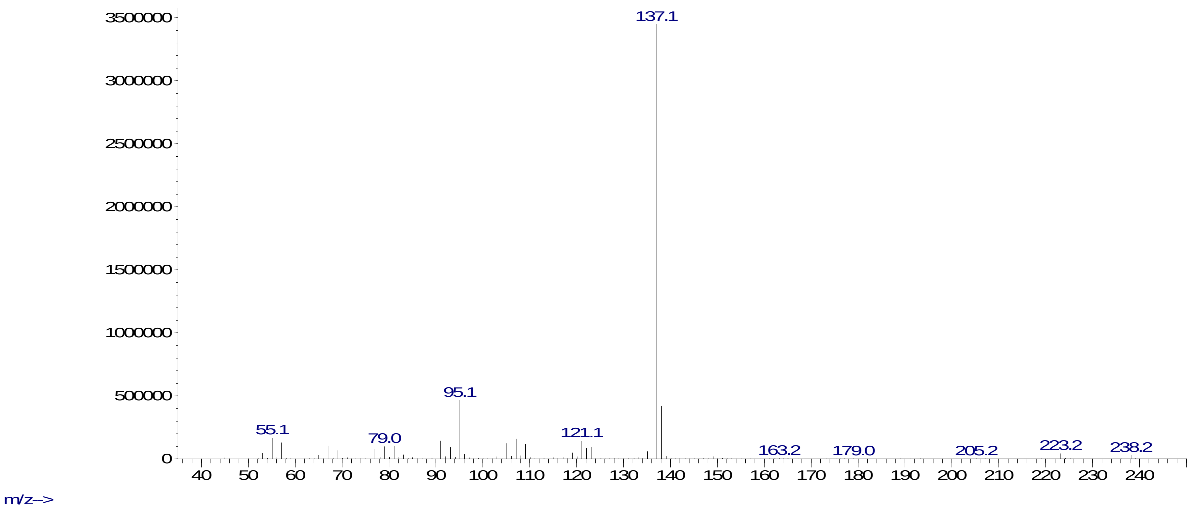

Supplementary Fig. 37. LR-EI-MS spectrum of compound **1c**.

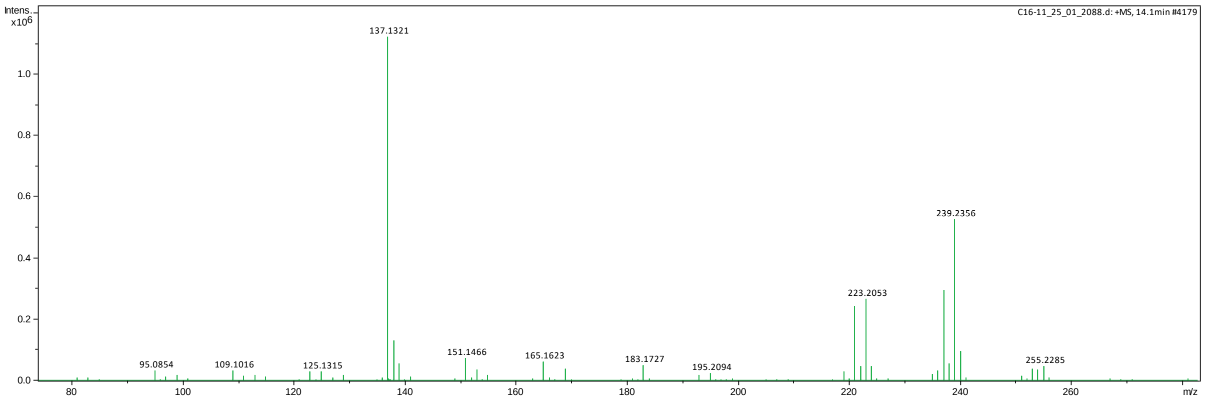

Supplementary Fig. 38. HR-APCI-MS spectrum of compound **1c**.

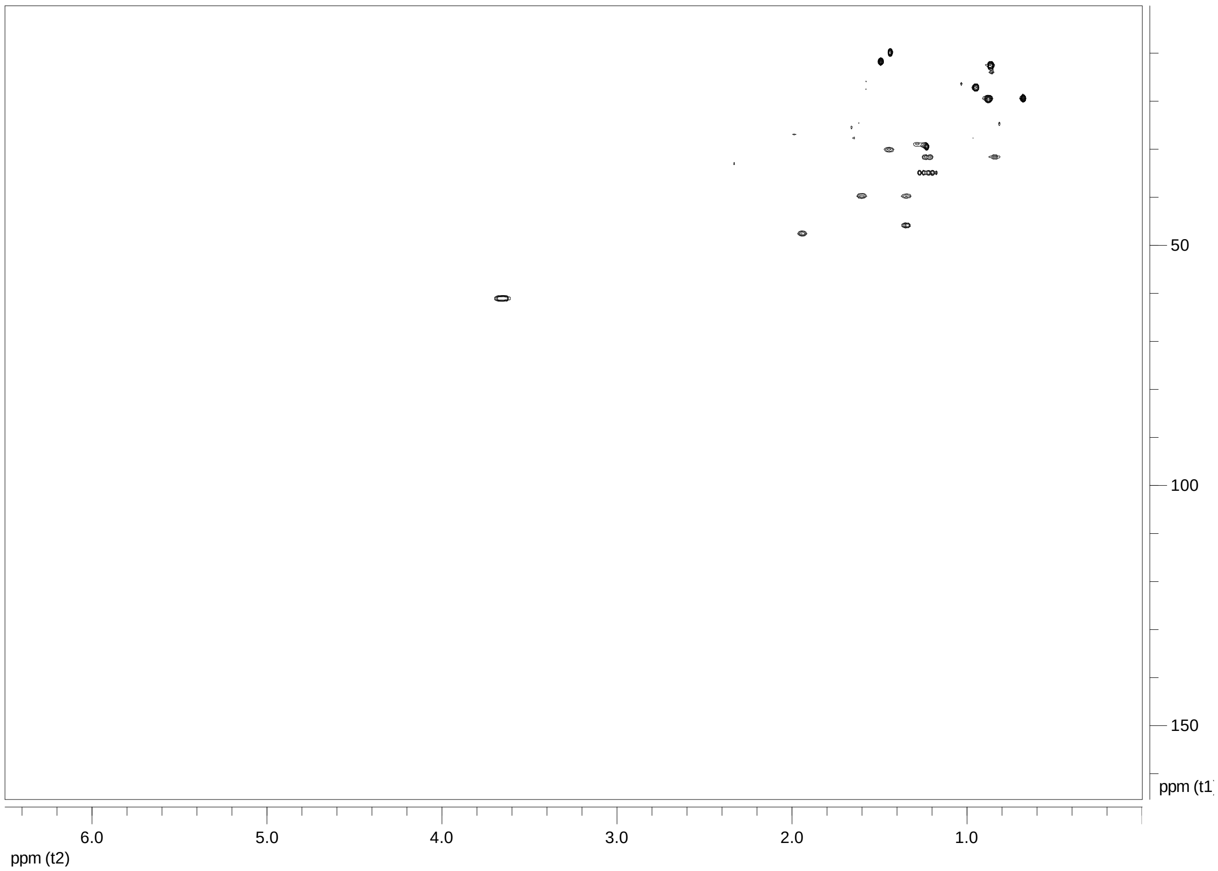

Supplementary Fig. 39. HSQC spectrum (in $CDCl_3$) of compound **1c**.

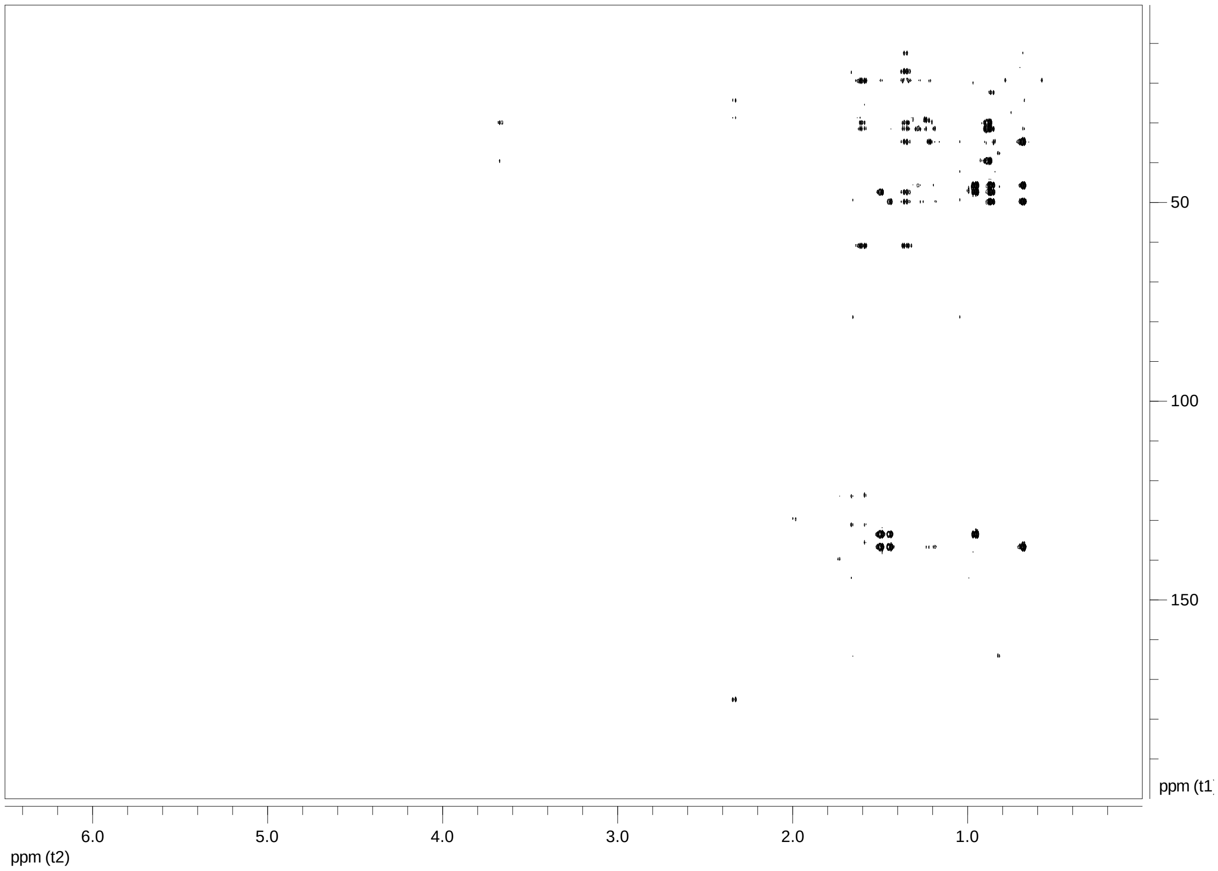

Supplementary Fig. 40. HMBC spectrum (in $CDCl_3$) of compound **1c**.

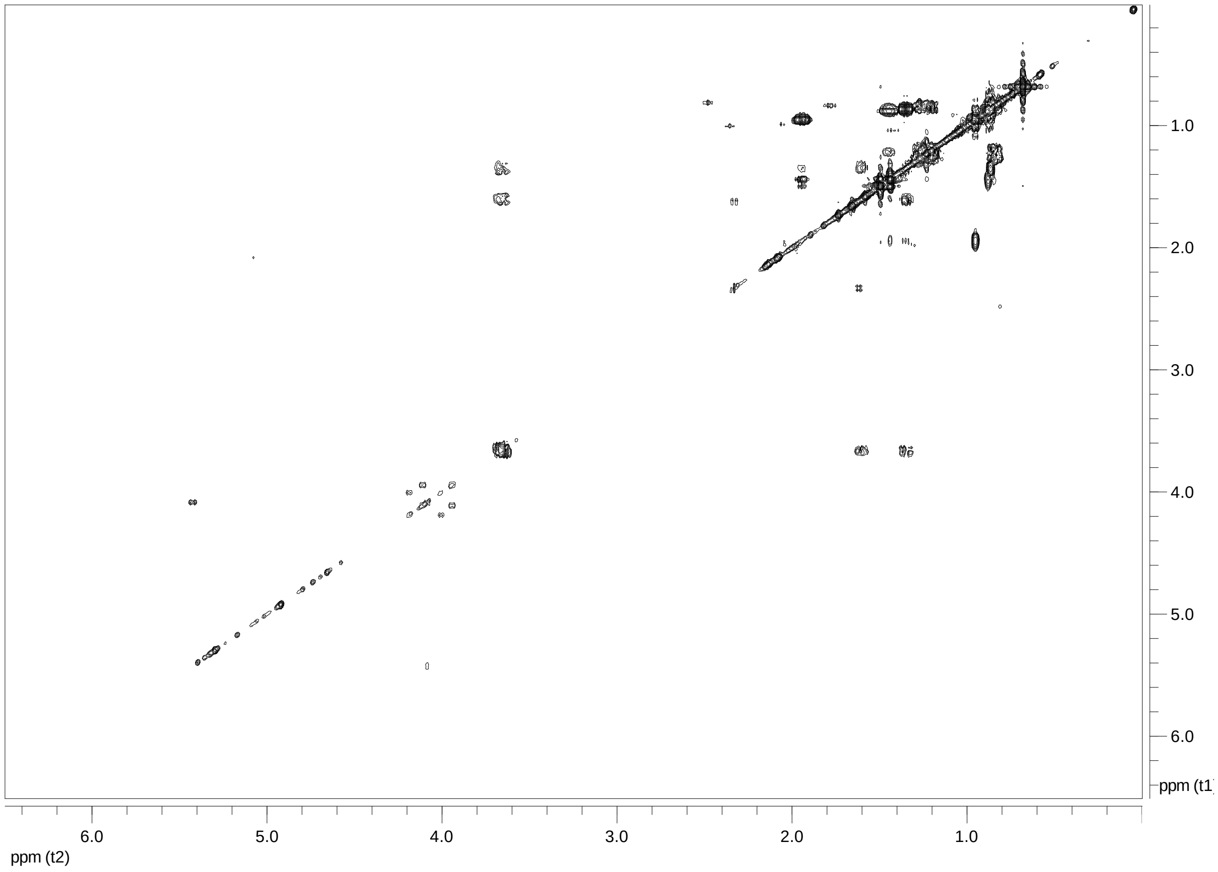

Supplementary Fig. 41. COSY spectrum (in $CDCl_3$) of compound **1c**.

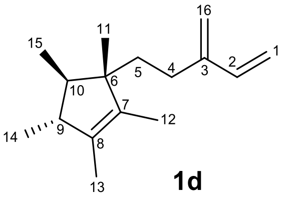

1d

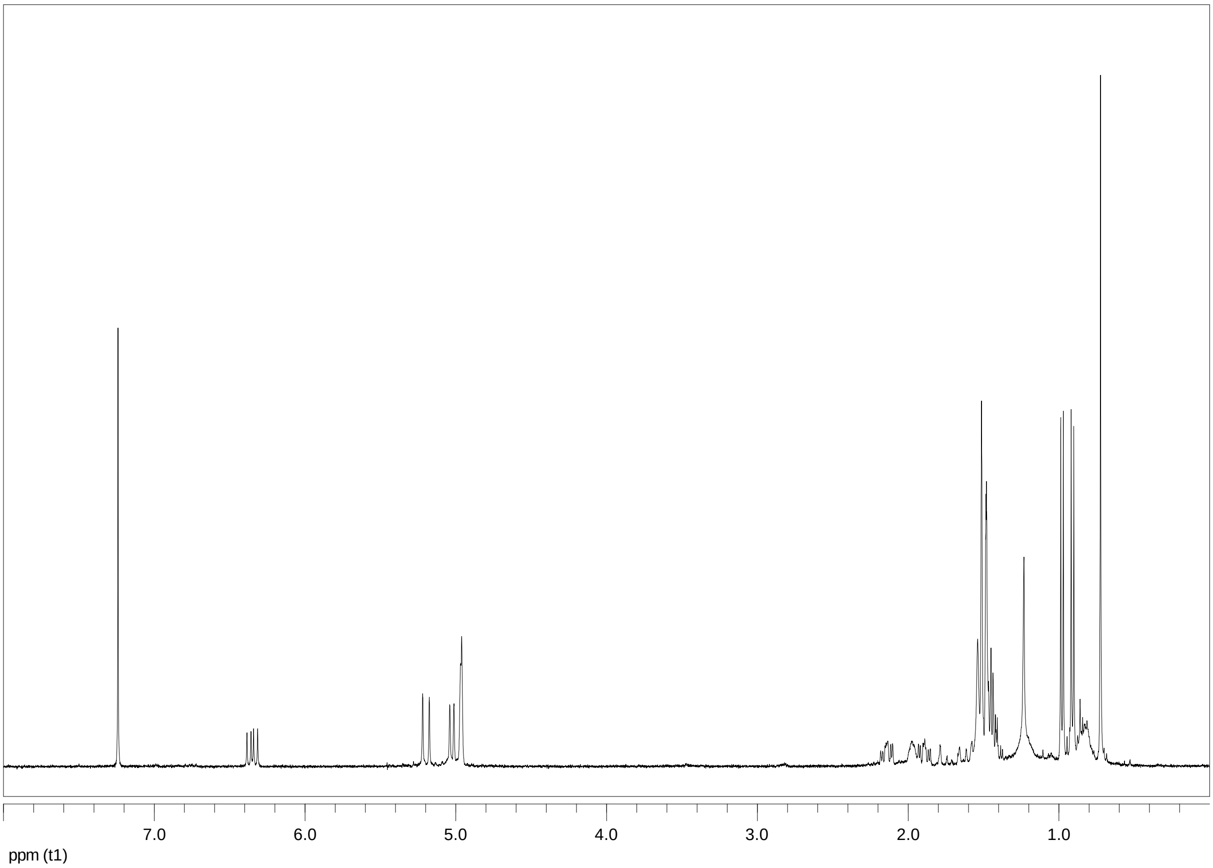

Supplementary Fig. 42. ^{1}H NMR spectrum (in $CDCl_3$) of compound **1d**.

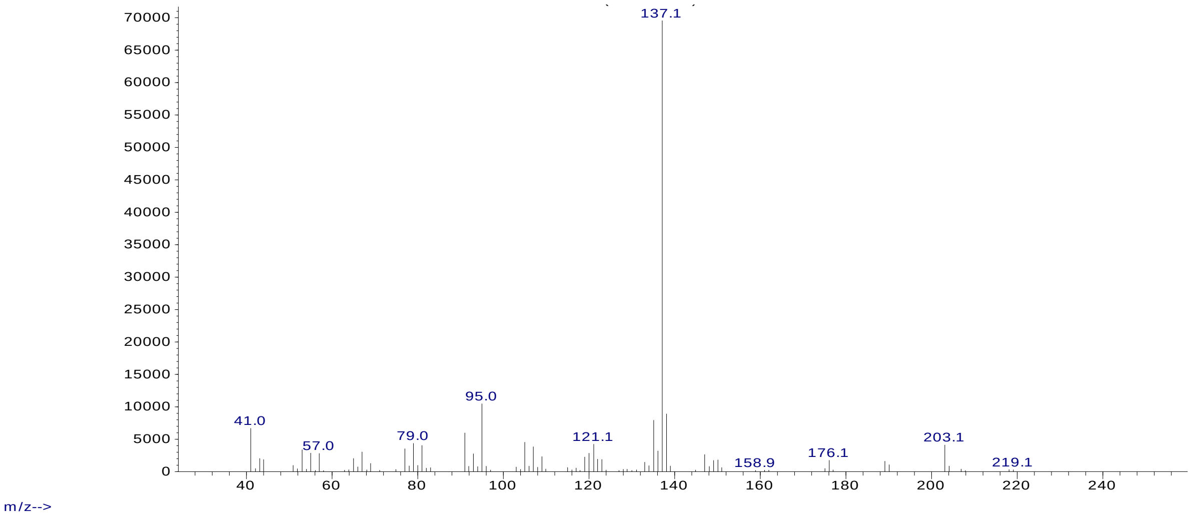

Supplementary Fig. 43. LR-EI-MS spectrum of compound **1d**.

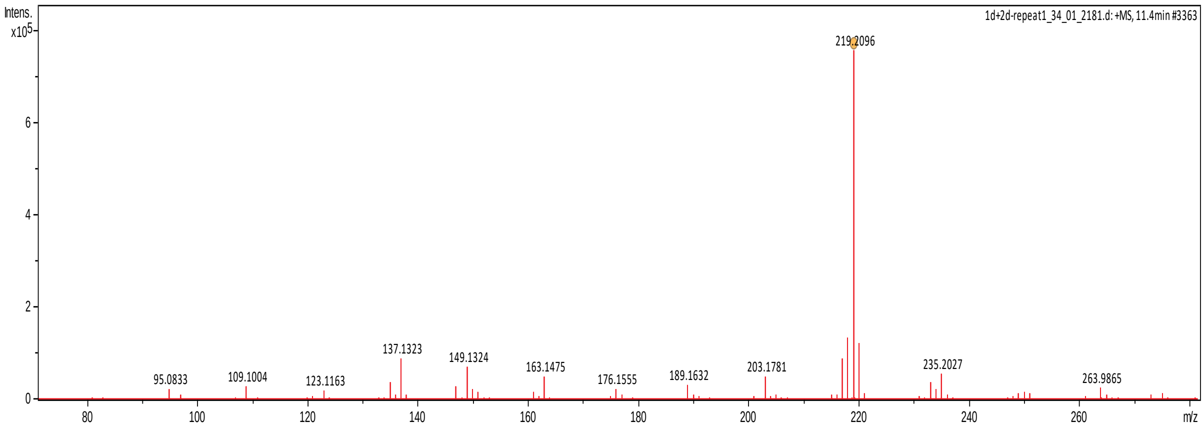

Supplementary Fig. 44. HR-APCI-MS spectrum of compound **1d**.

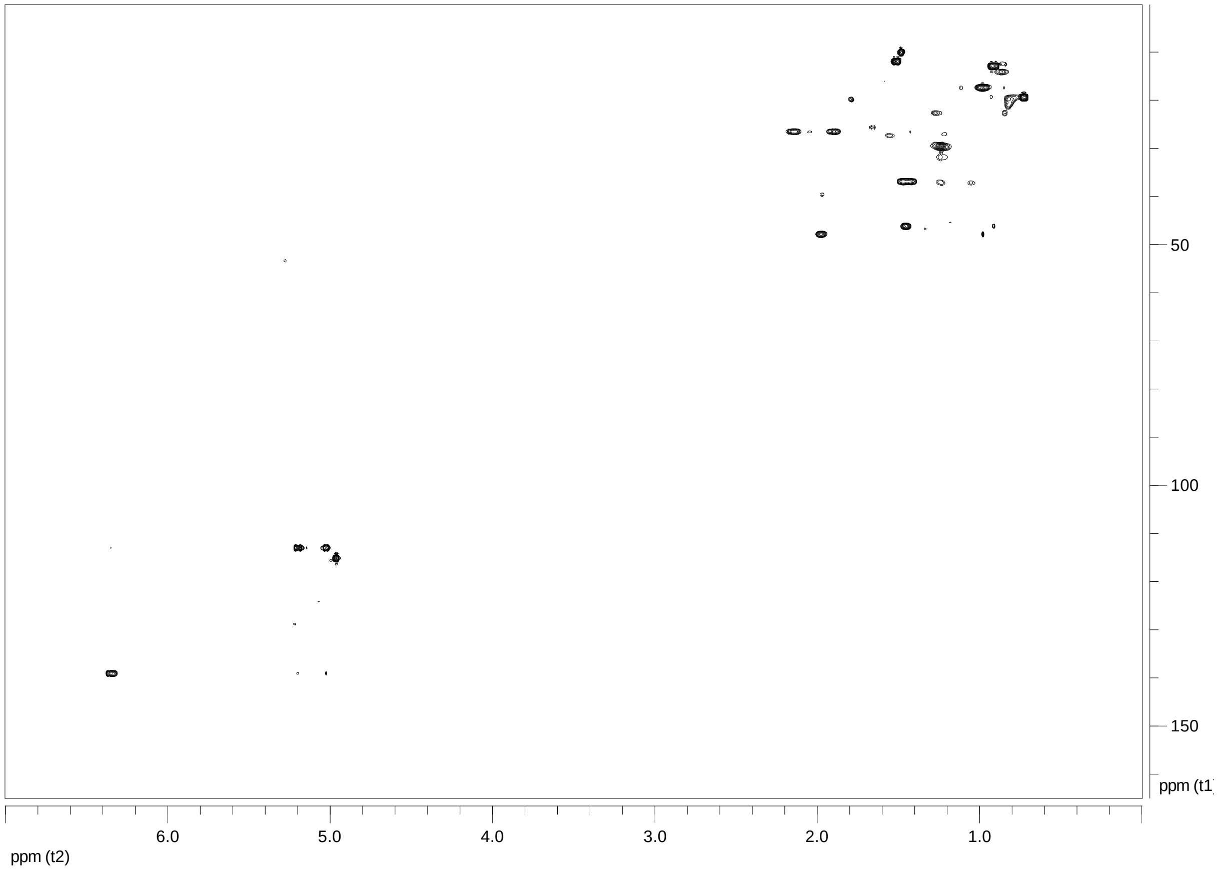

Supplementary Fig. 45. HSQC spectrum (in $CDCl_3$) of compound **1d**.

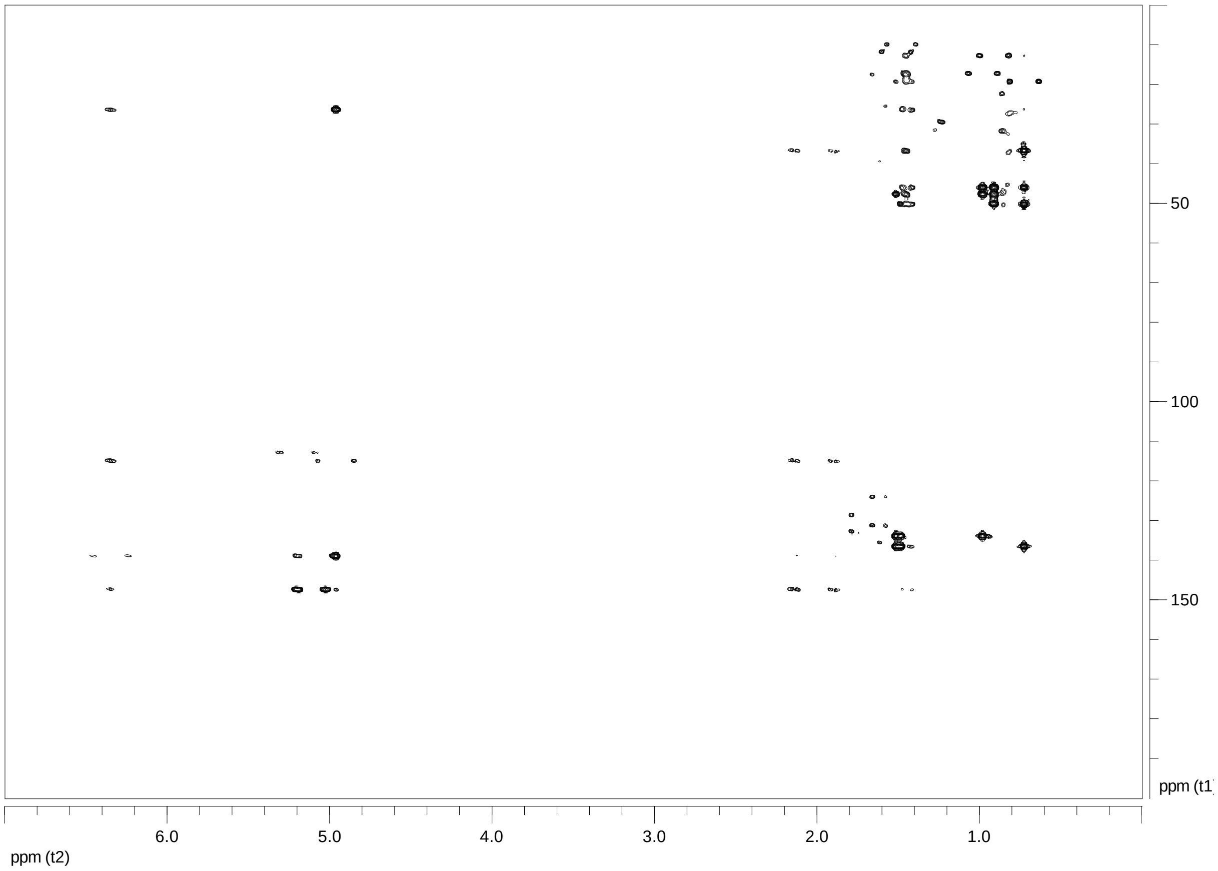

Supplementary Fig. 46. HMBC spectrum (in $CDCl_3$) of compound **1d**.

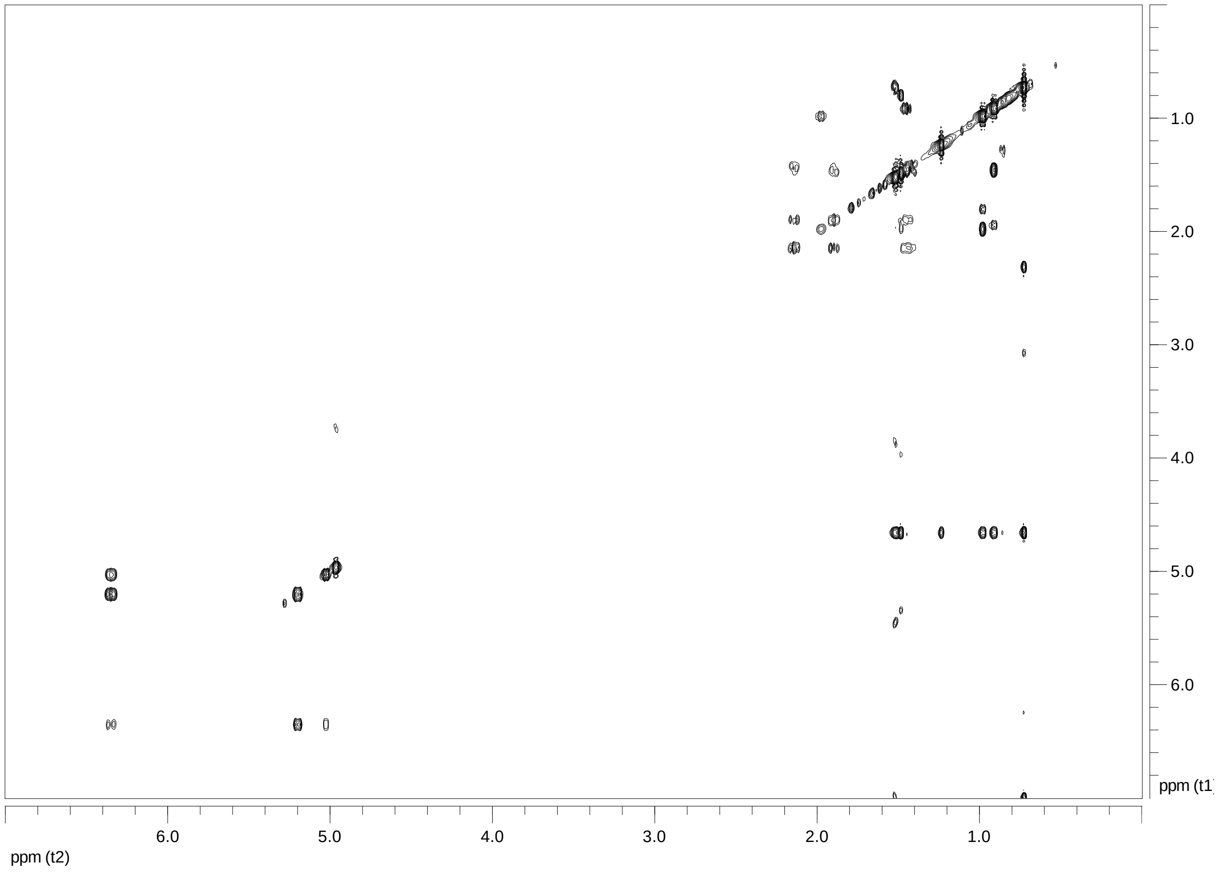

Supplementary Fig. 47. COSY spectrum (in $CDCl_3$) of compound **1d**.

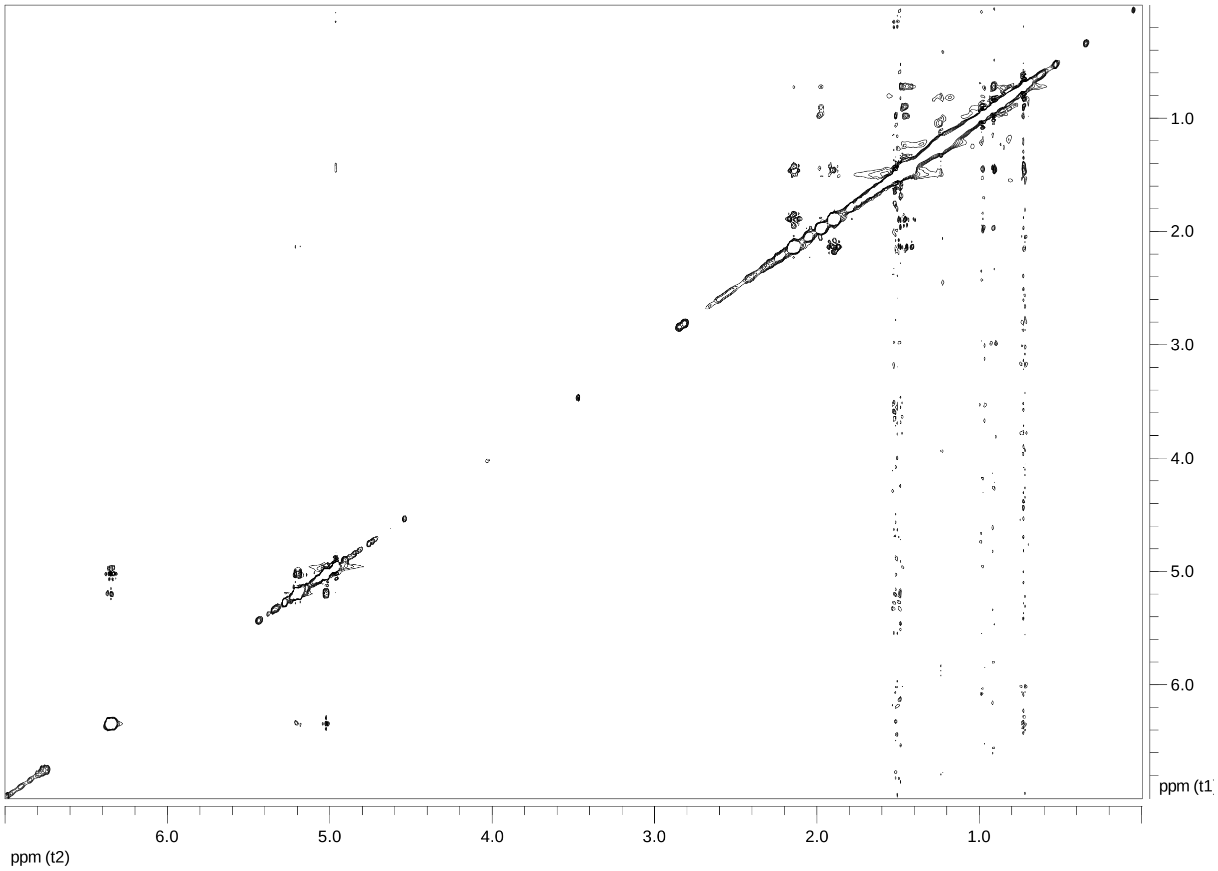

Supplementary Fig. 48. NOESY spectrum (in $CDCl_3$) of compound **1d**.

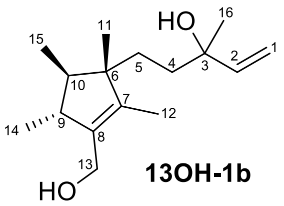

13OH-1b

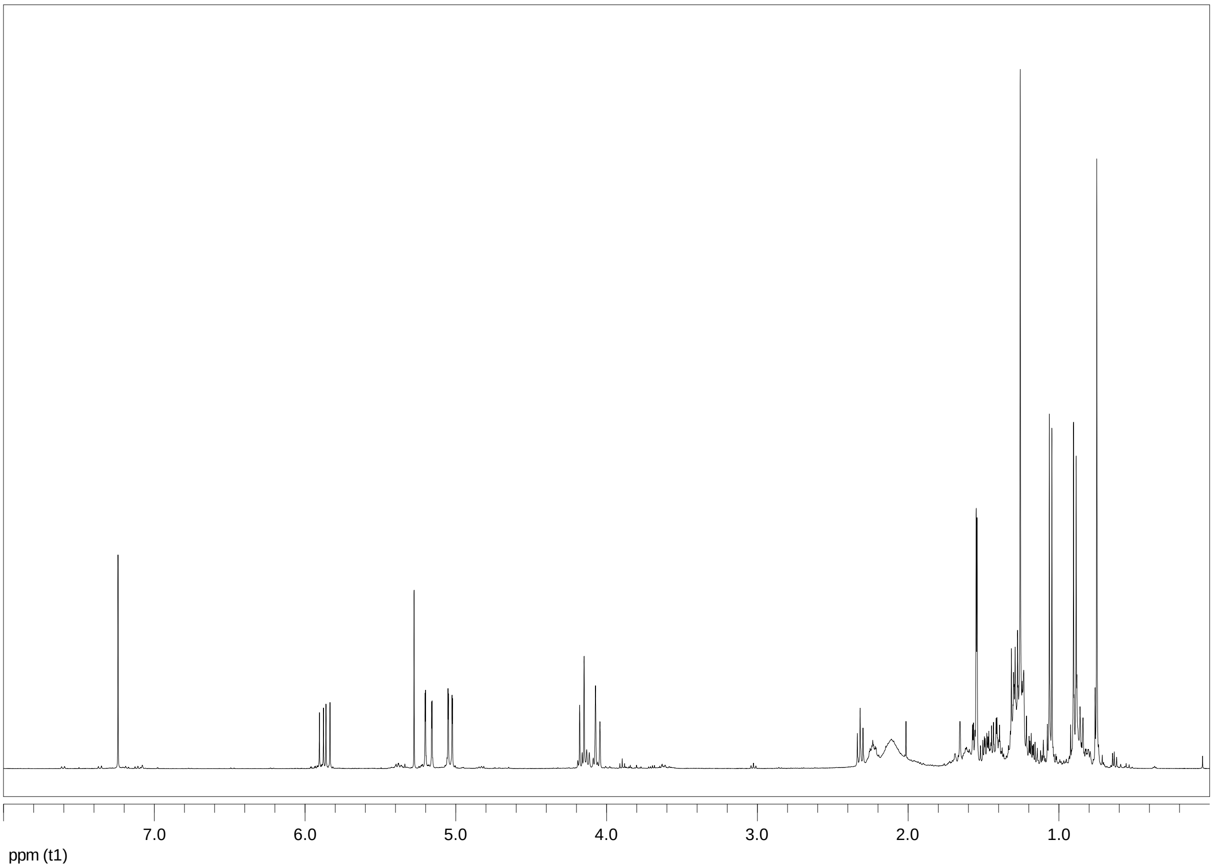

Supplementary Fig. 49. ^{1}H NMR spectrum (in $CDCl_3$) of compound **13OH-1b**.

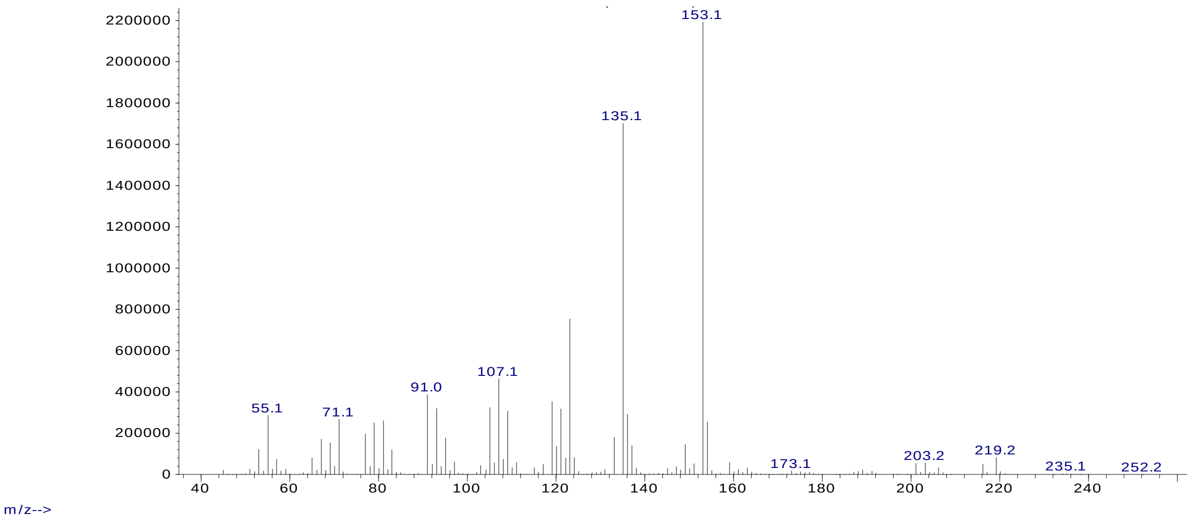

Supplementary Fig. 50. LR-EI-MS spectrum of compound **13OH-1b**.

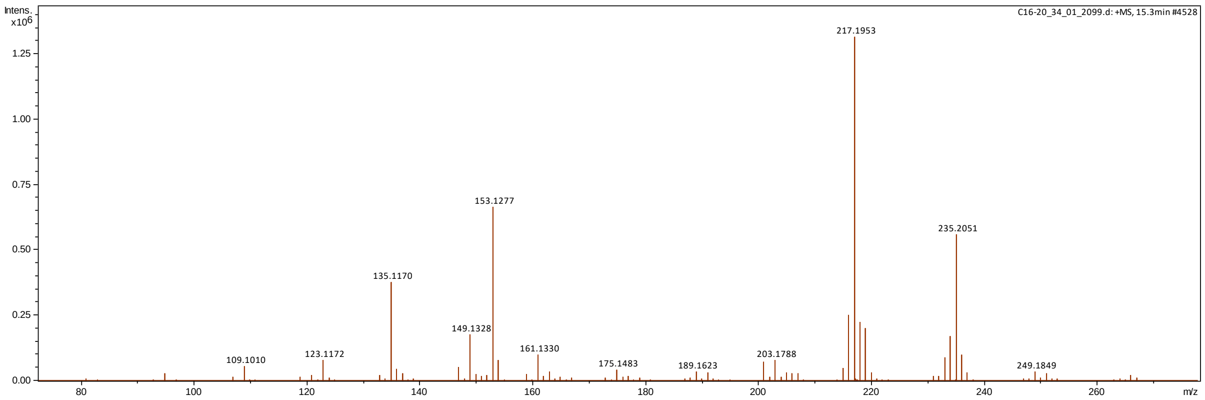

Supplementary Fig. 51. HR-APCI-MS spectrum of compound **13OH-1b**.

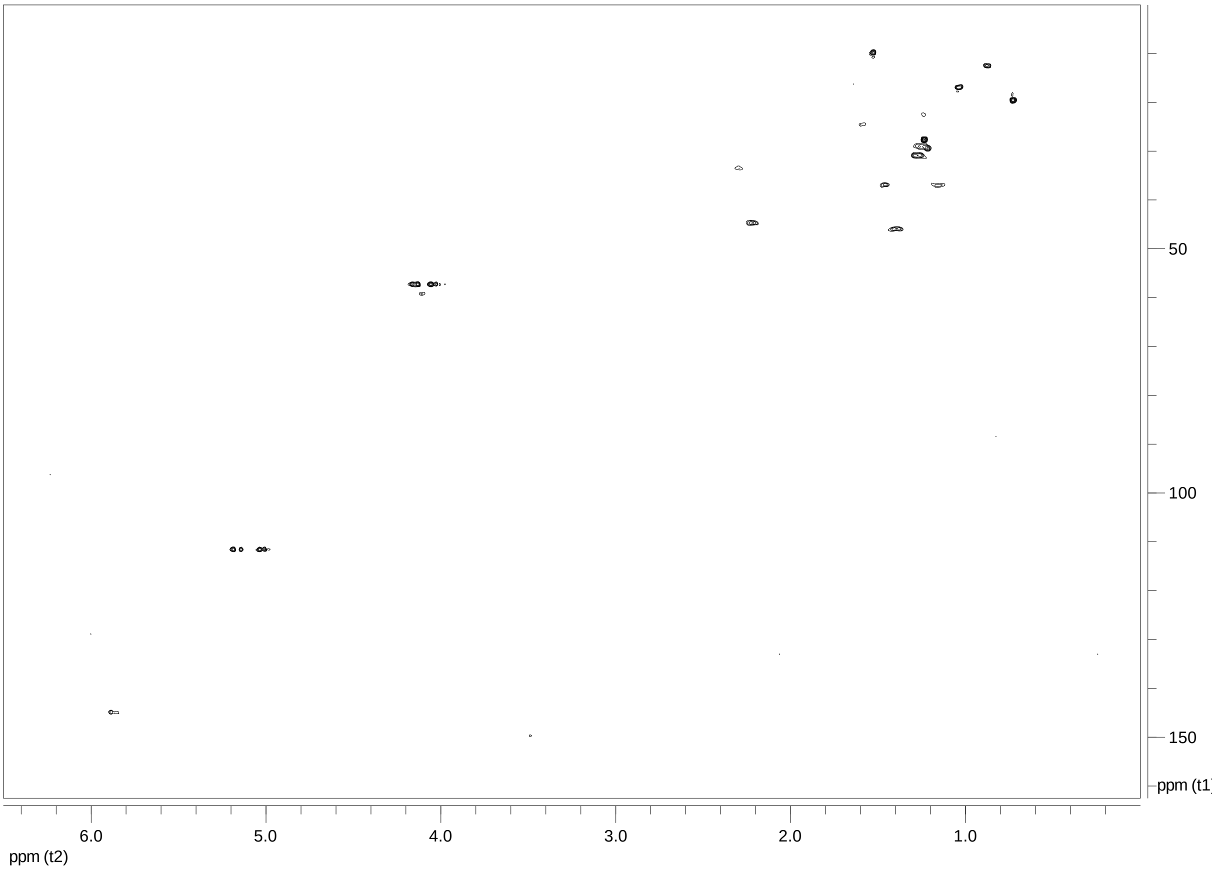

Supplementary Fig. 52. HSQC spectrum (in $CDCl_3$) of compound **13OH-1b**.

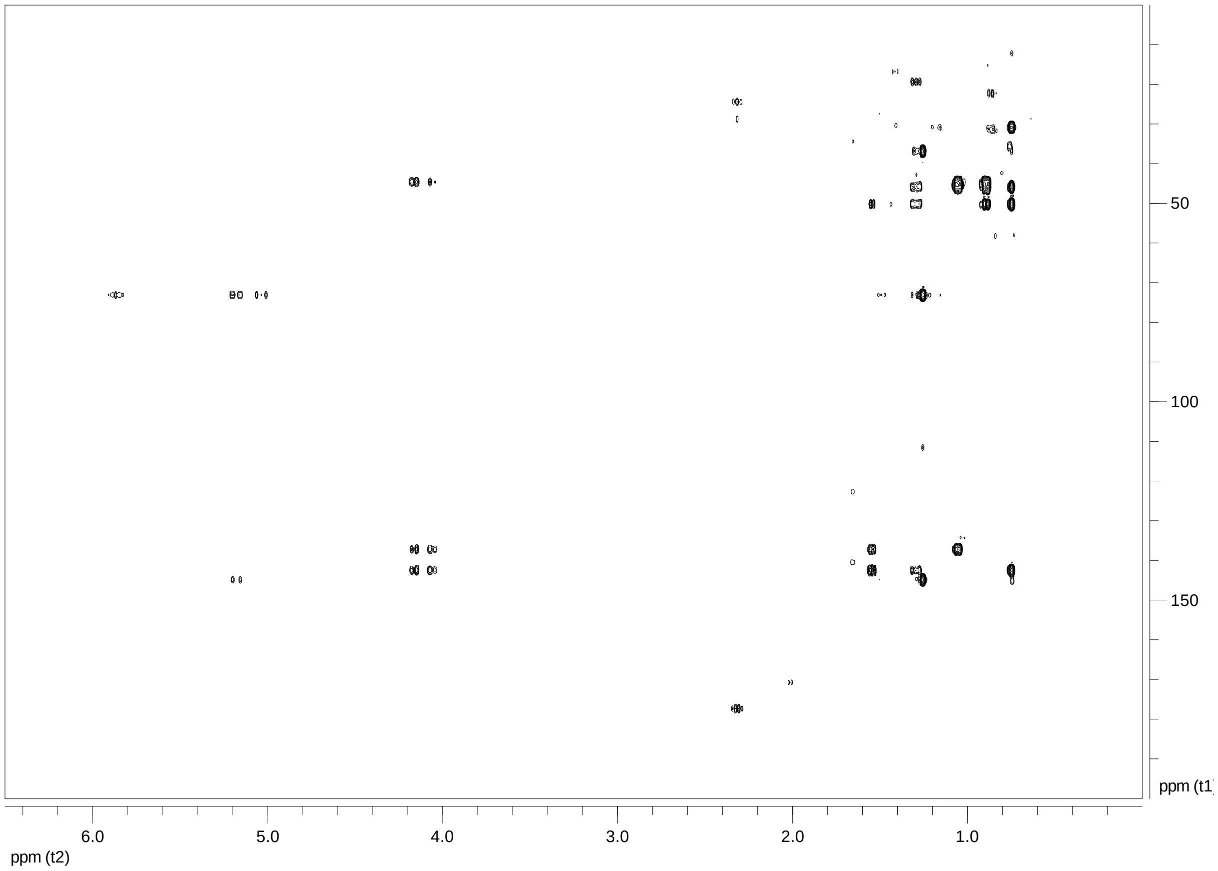

Supplementary Fig. 53. HMBC spectrum (in $CDCl_3$) of compound **13OH-1b**.

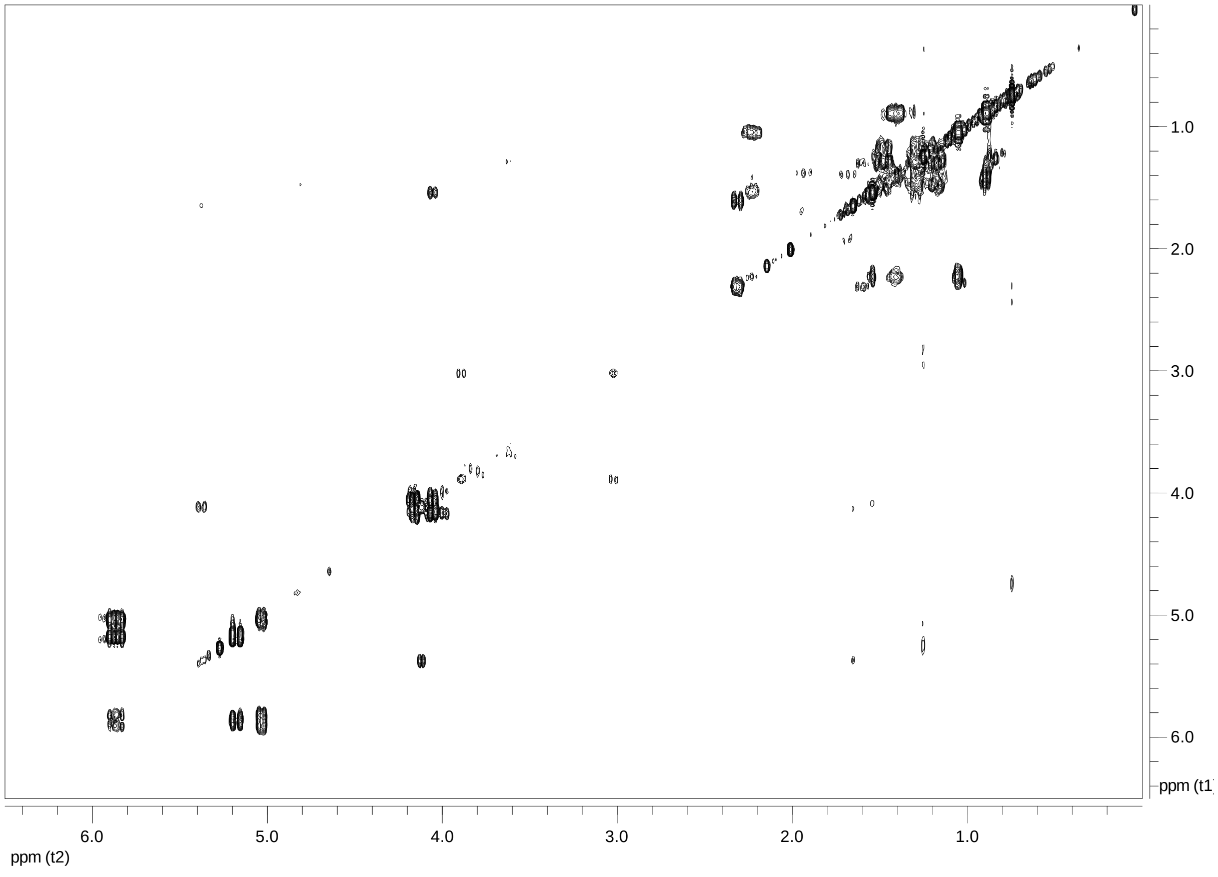

Supplementary Fig. 54. COSY spectrum (in $CDCl_3$) of compound **13OH-1b**.

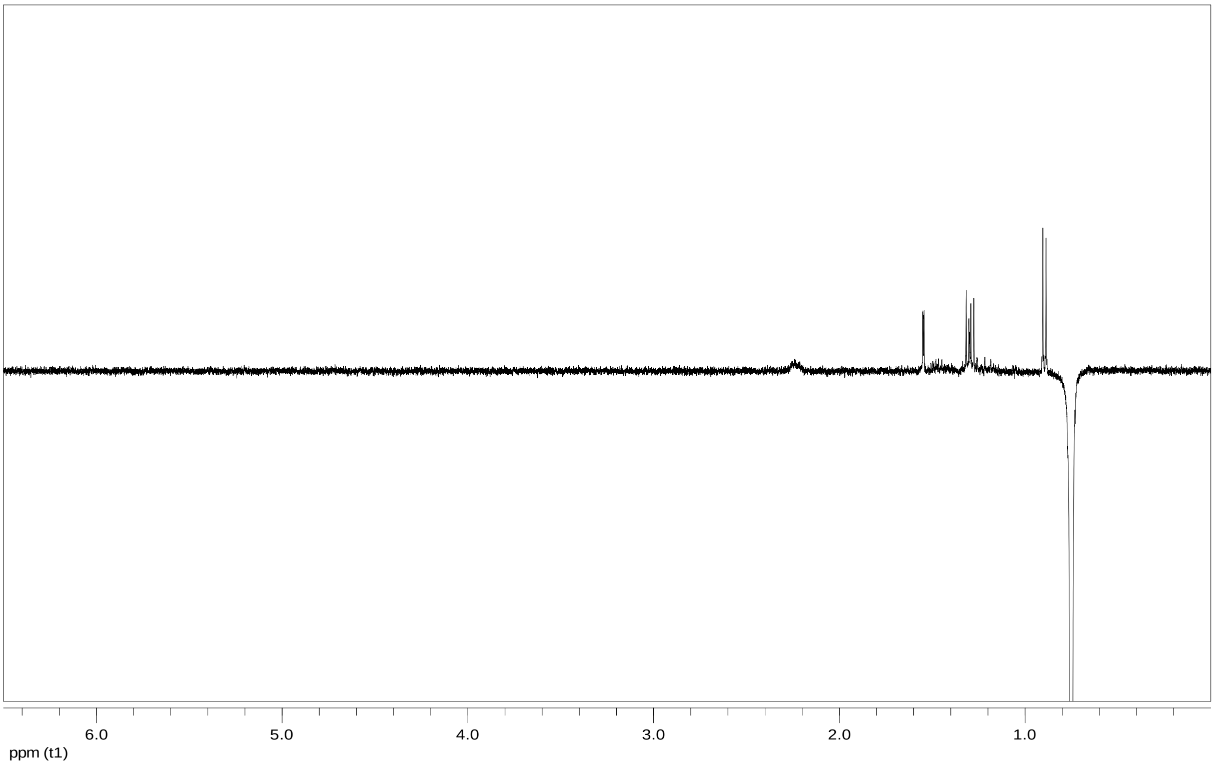

Supplementary Fig. 55. 1D NOE difference spectrum (in $CDCl_3$) of compound **13OH-1b** upon irradiation of H_3-11.

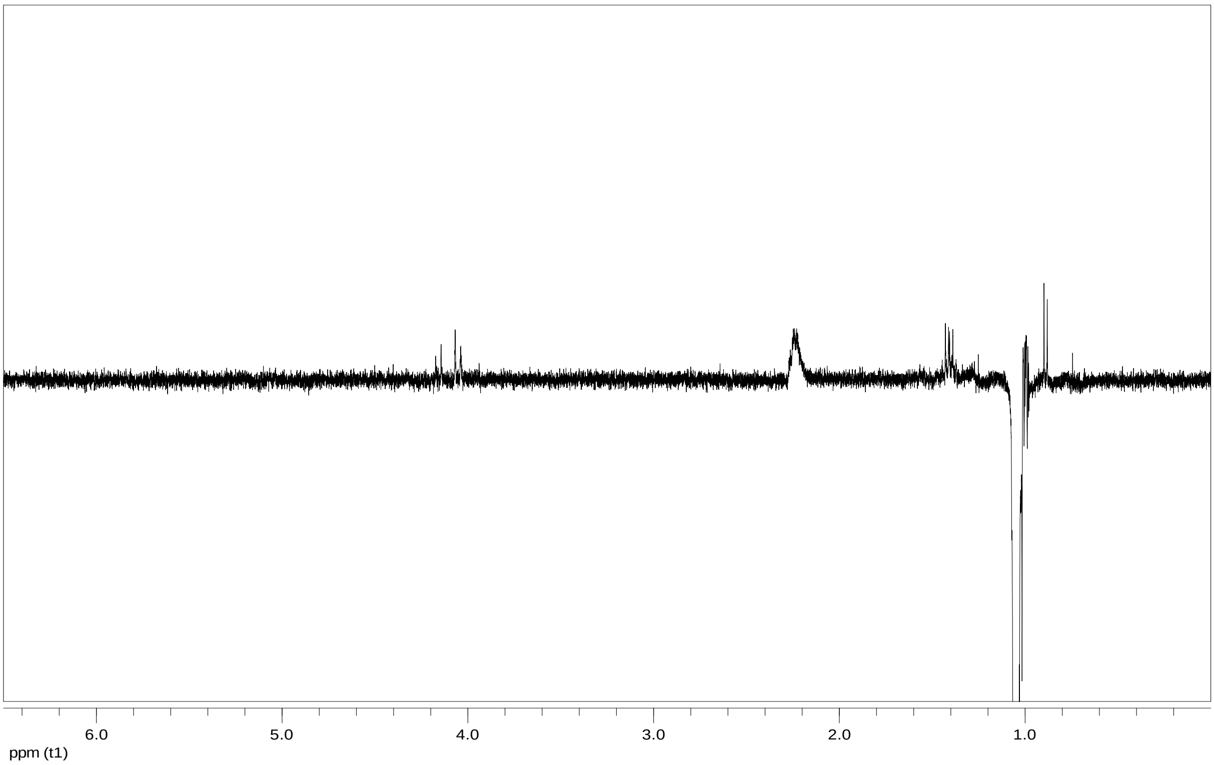

Supplementary Fig. 56. 1D NOE difference spectrum (in $CDCl_3$) of compound **13OH-1b** upon irradiation of H_3-14.

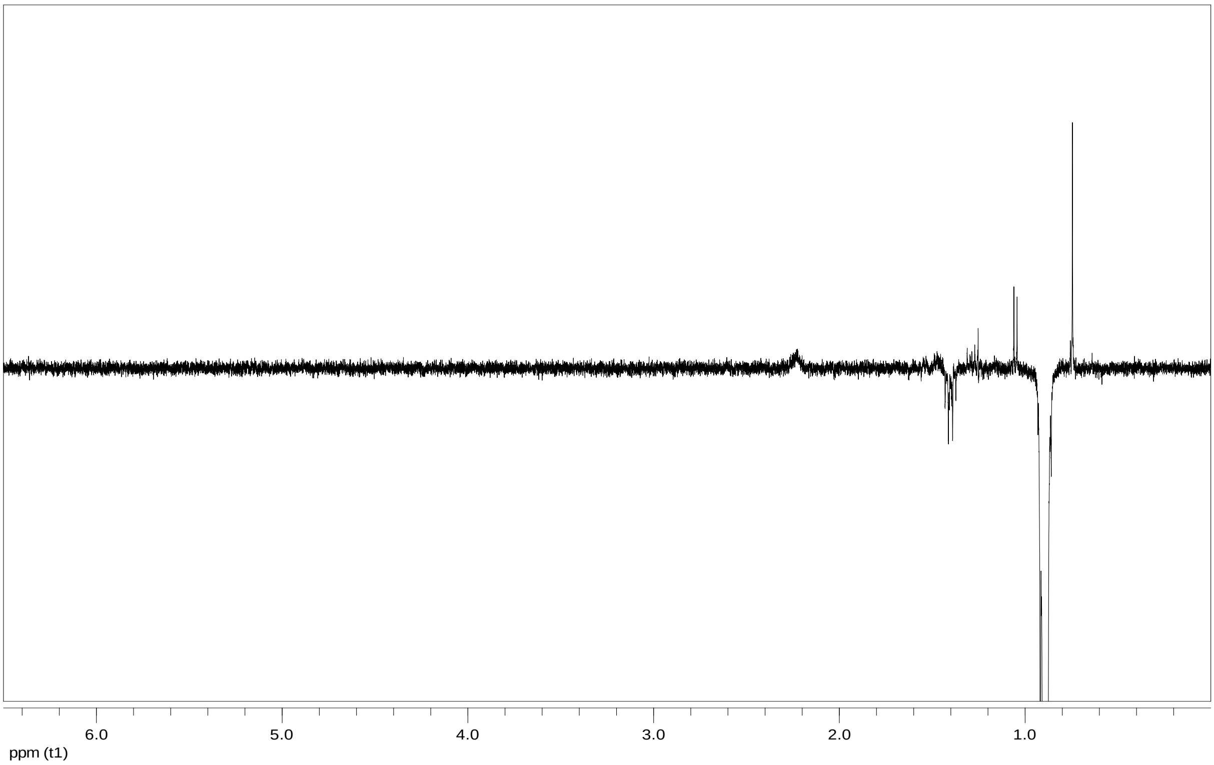

Supplementary Fig. 57. 1D NOE difference spectrum (in $CDCl_3$) of compound **13OH-1b** upon irradiation of H_3-15.

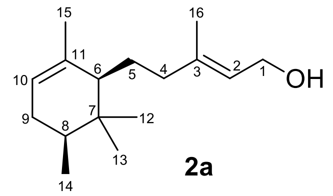

2a

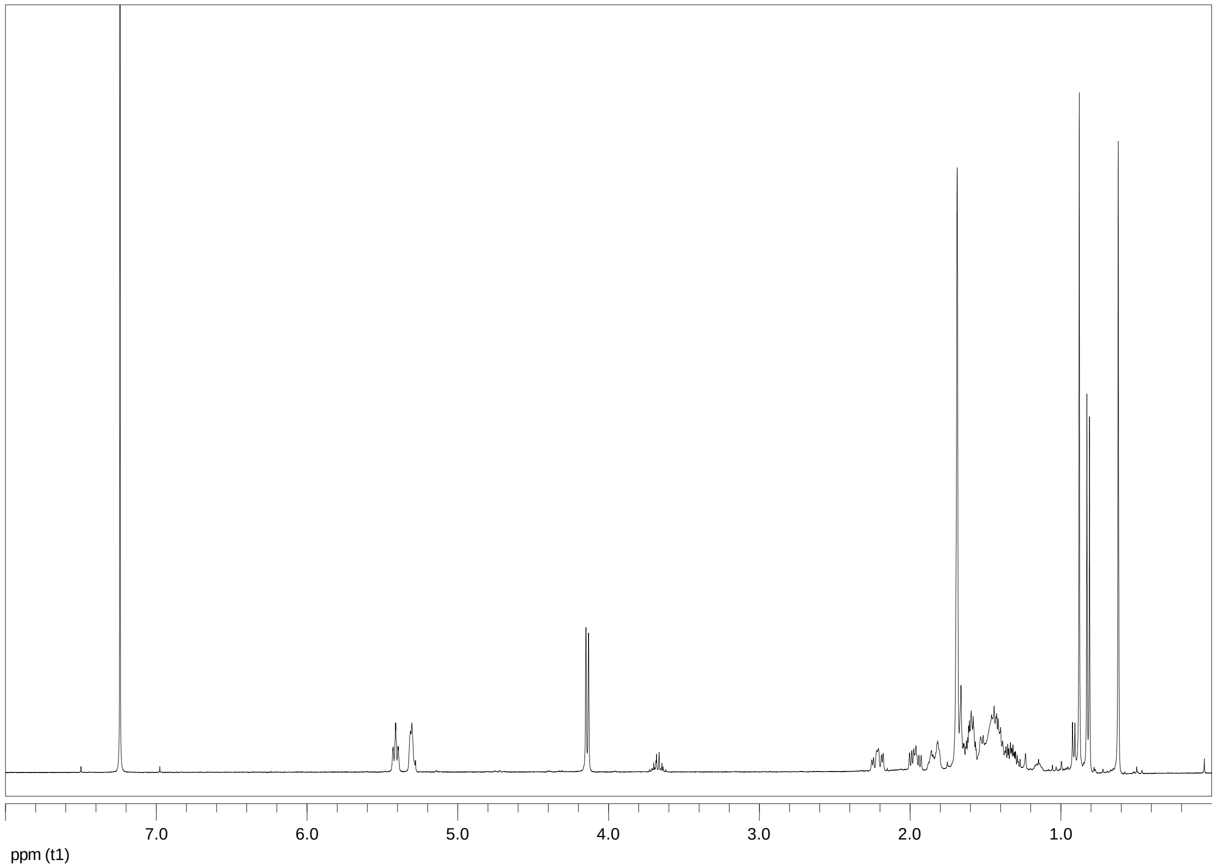

Supplementary Fig. 58. ^{1}H NMR spectrum (in $CDCl_3$) of compound **2a**.

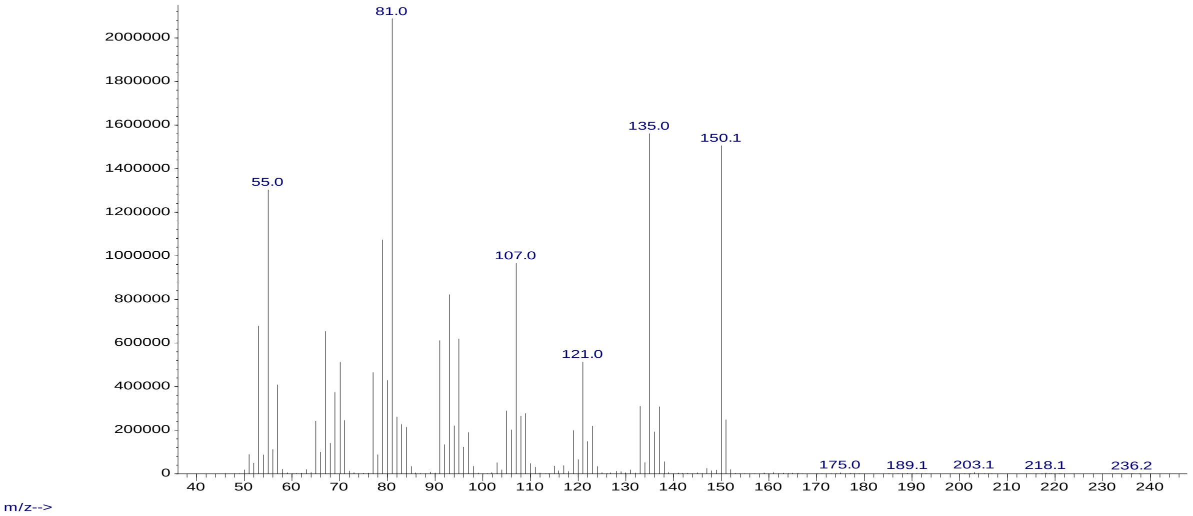

Supplementary Fig. 59. LR-EI-MS spectrum of compound **2a**.

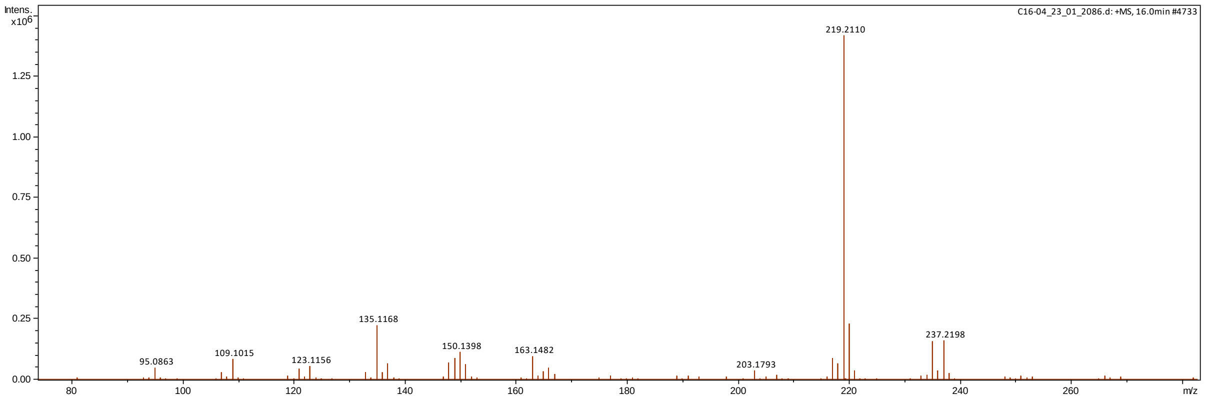

Supplementary Fig. 60. HR-APCI-MS spectrum of compound **2a**.

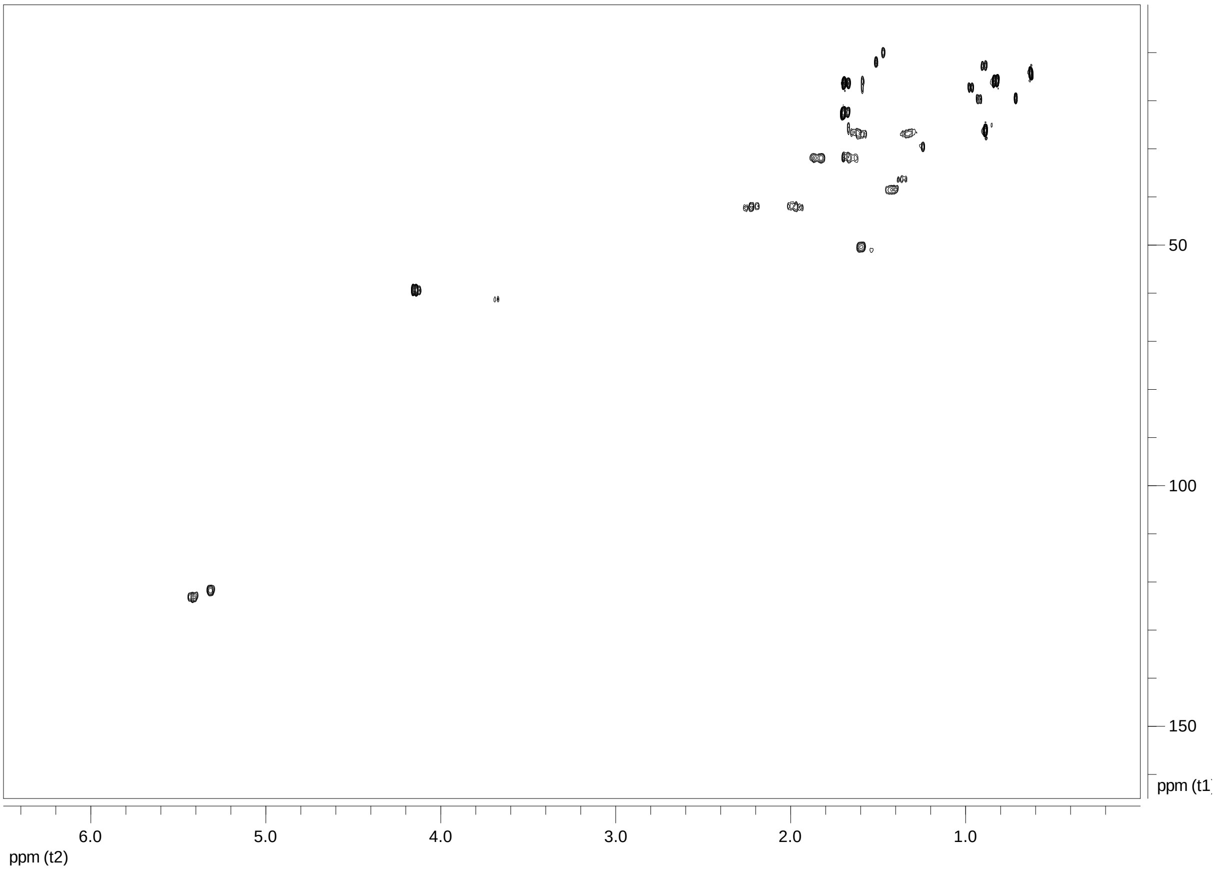

Supplementary Fig. 61. HSQC spectrum (in $CDCl_3$) of compound **2a**.

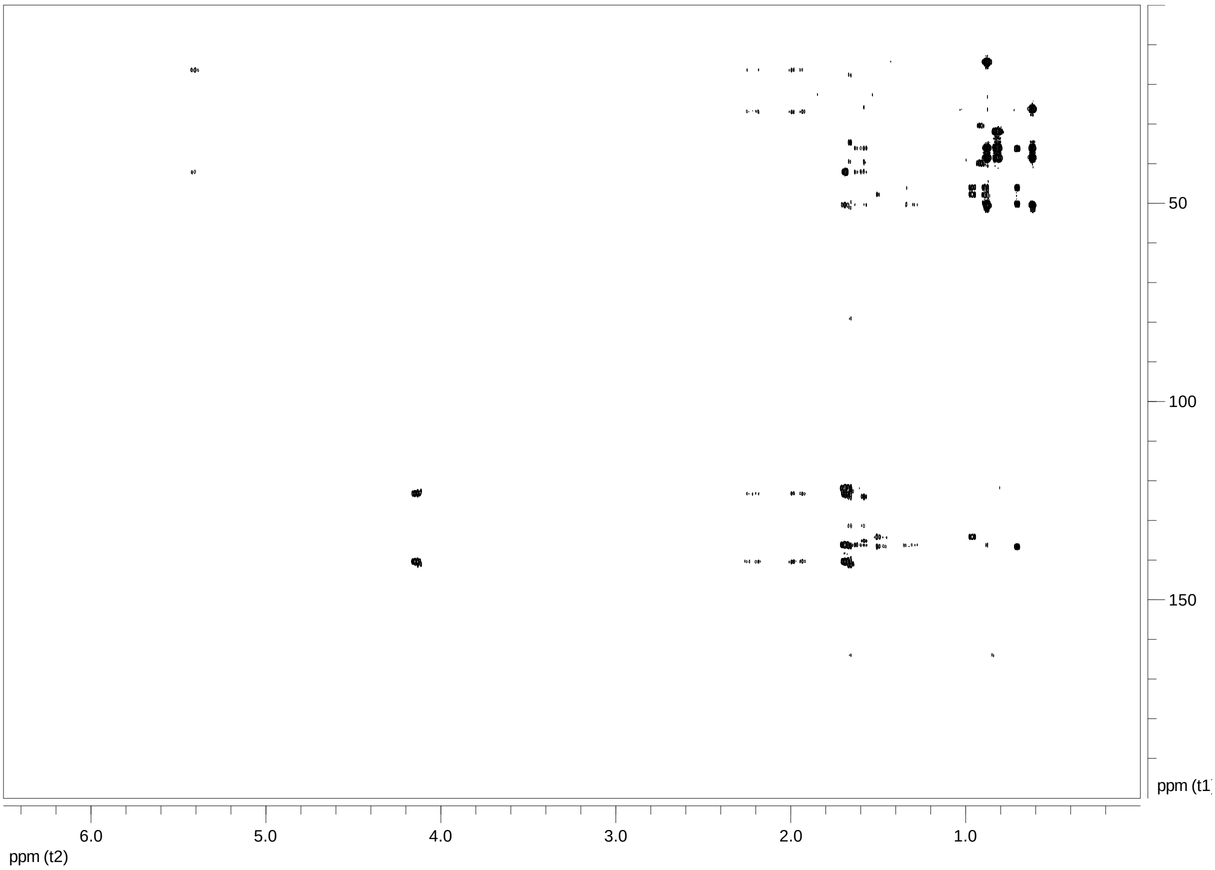

Supplementary Fig. 62. HMBC spectrum (in $CDCl_3$) of compound **2a**.

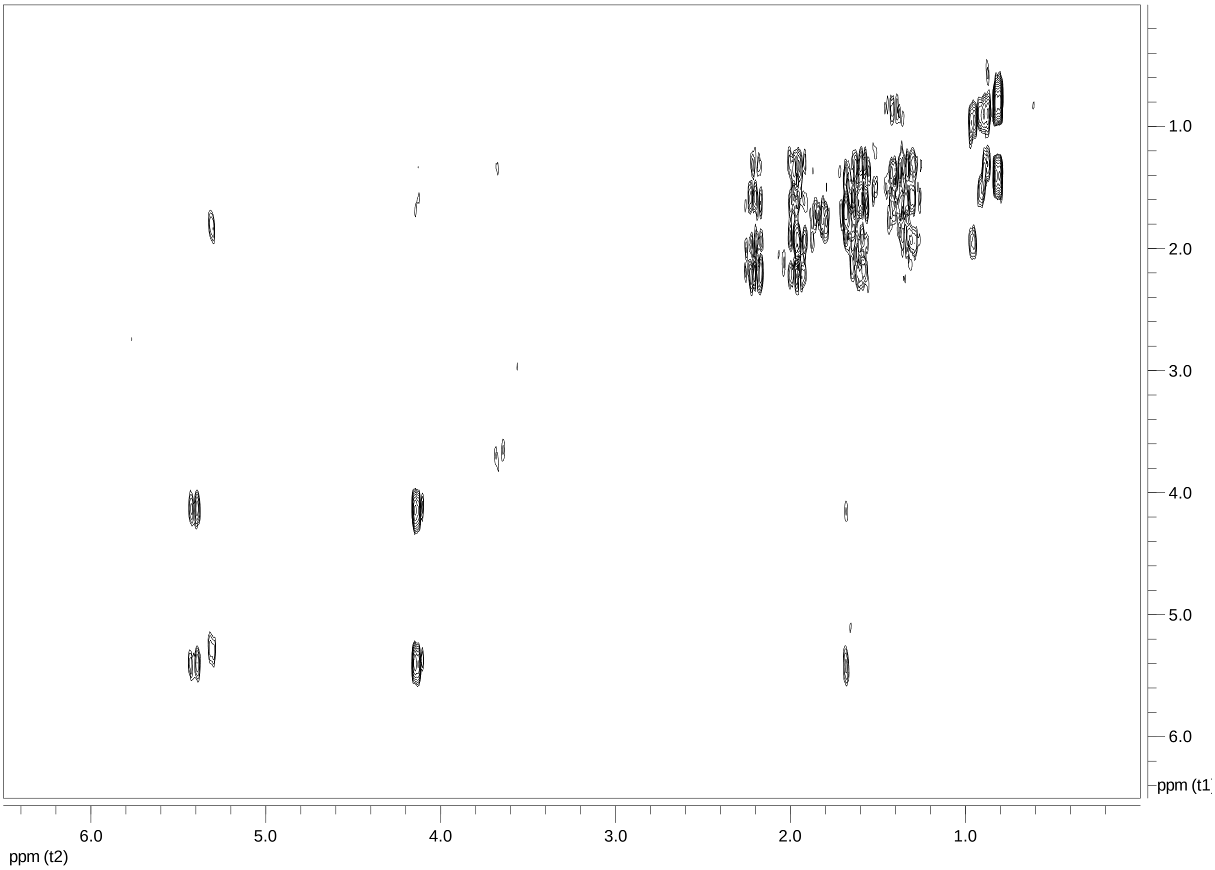

Supplementary Fig. 63. COSY spectrum (in $CDCl_3$) of compound **2a**.

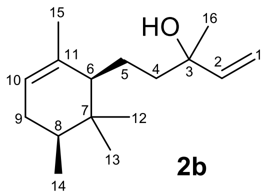

2b

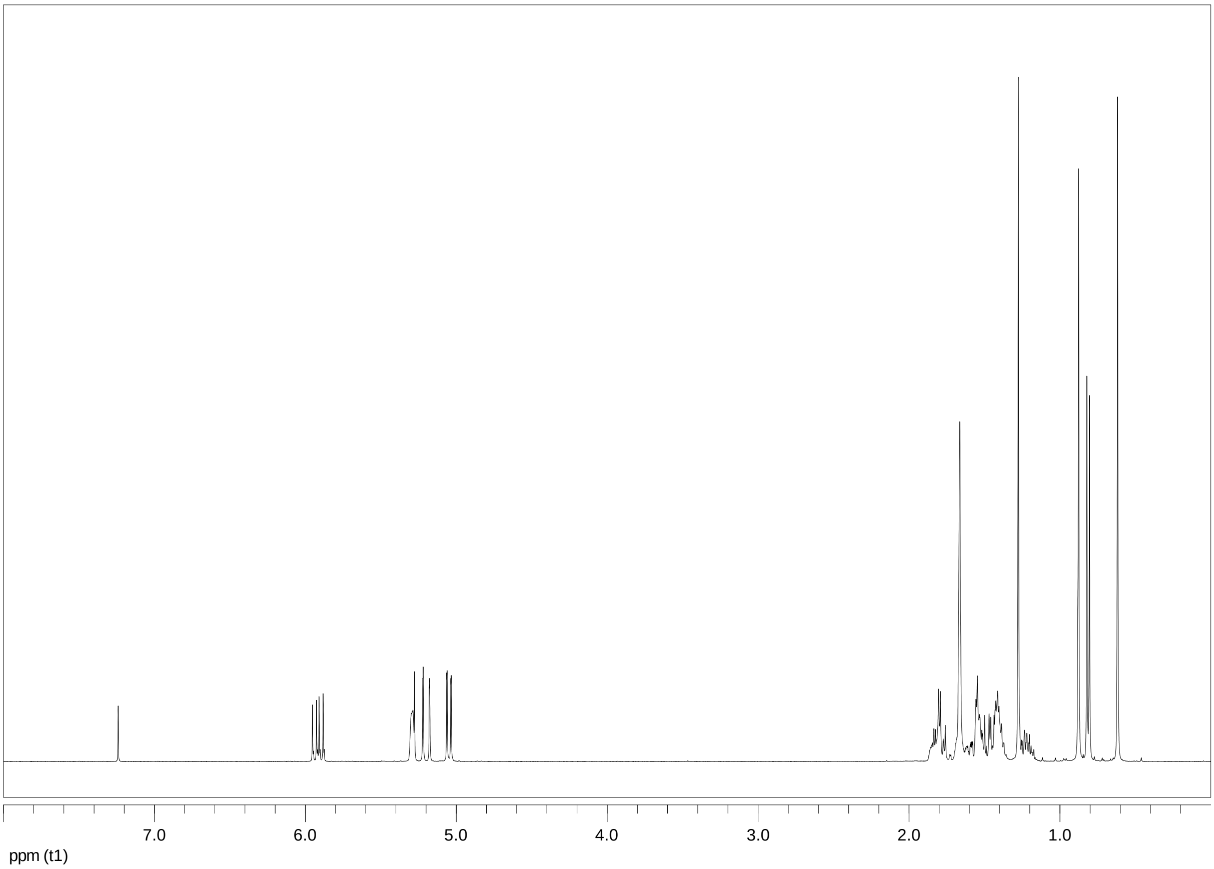

Supplementary Fig. 64. ^{1}H NMR spectrum (in $CDCl_3$) of compound **2b**.

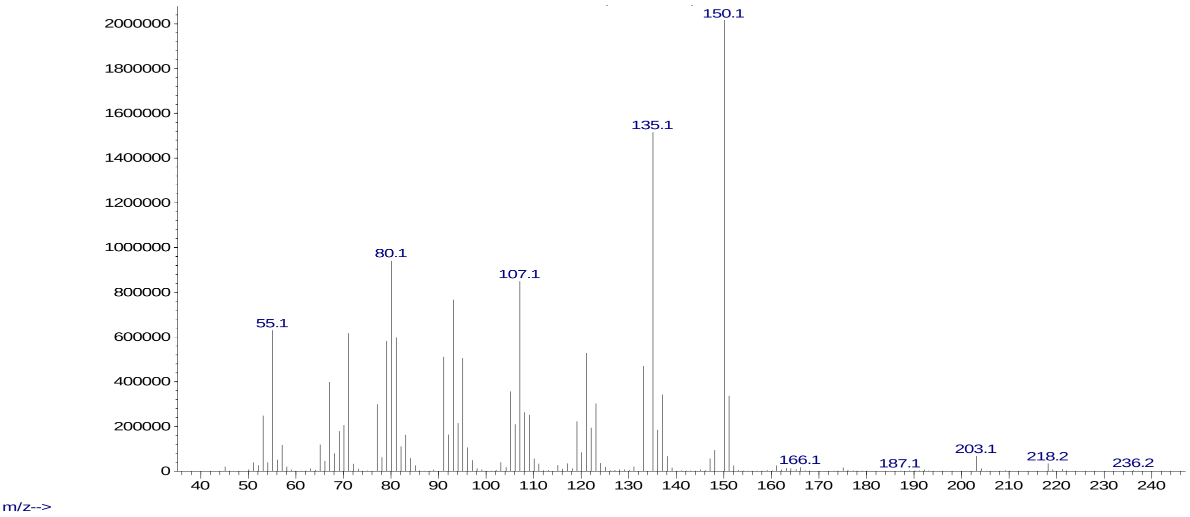

Supplementary Fig. 65. LR-EI-MS spectrum of compound **2b**.

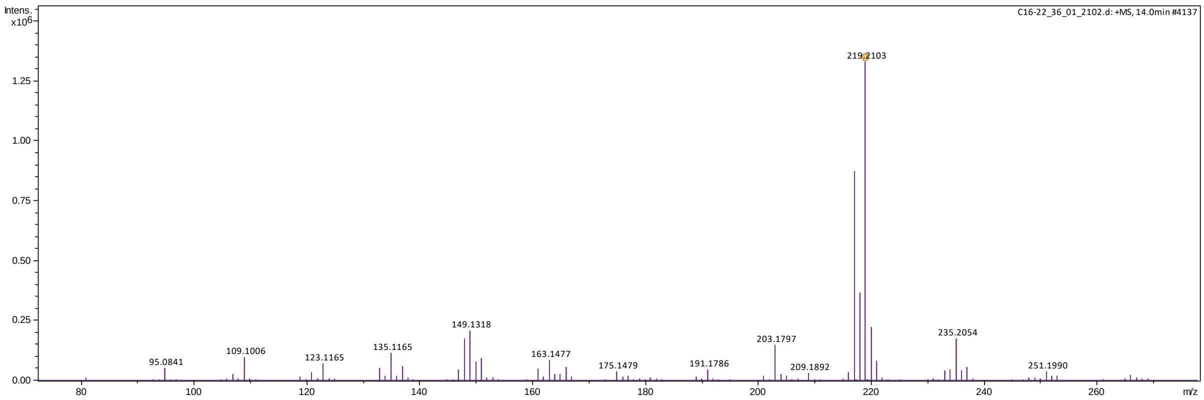

Supplementary Fig. 66. HR-APCI-MS spectrum of compound **2b**.

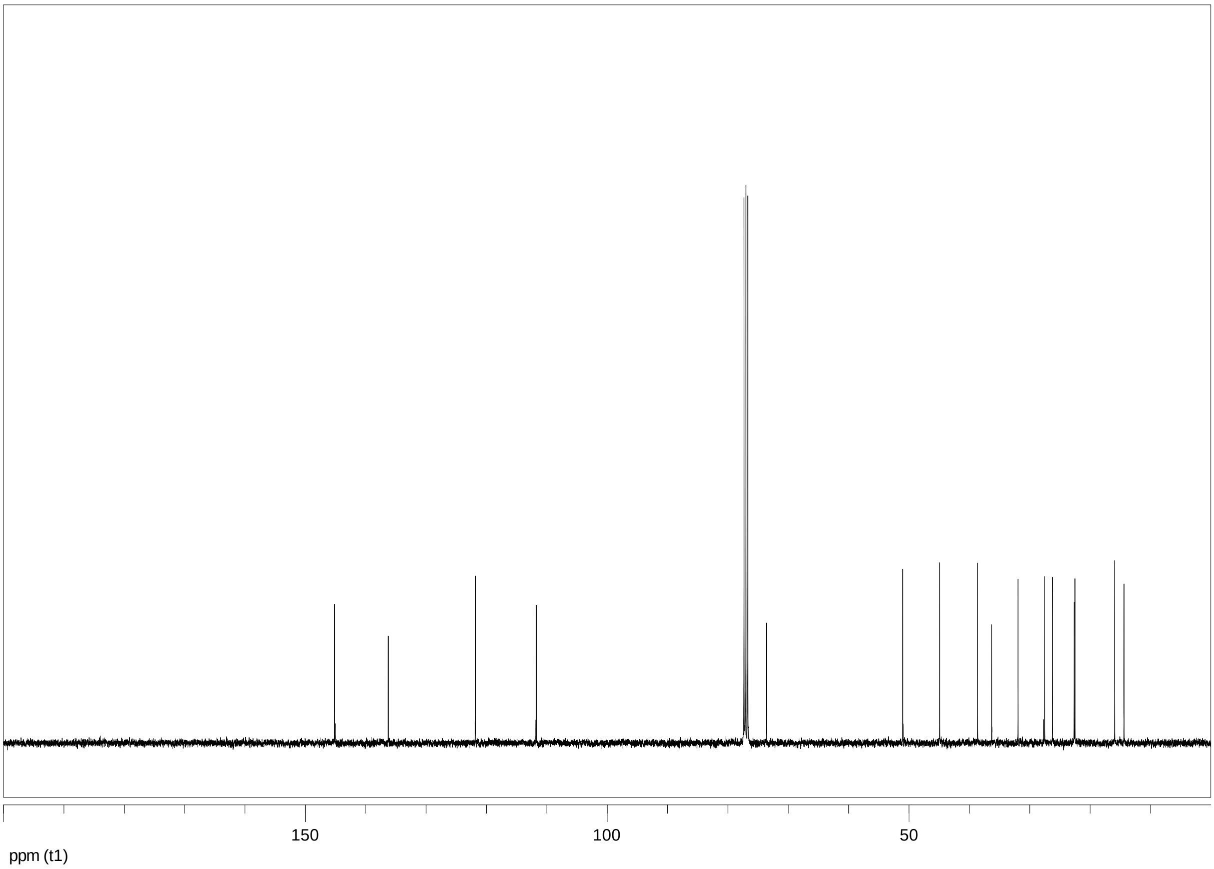

Supplementary Fig. 67. ^{13}C NMR spectrum (in $CDCl_3$) of compound **2b**.

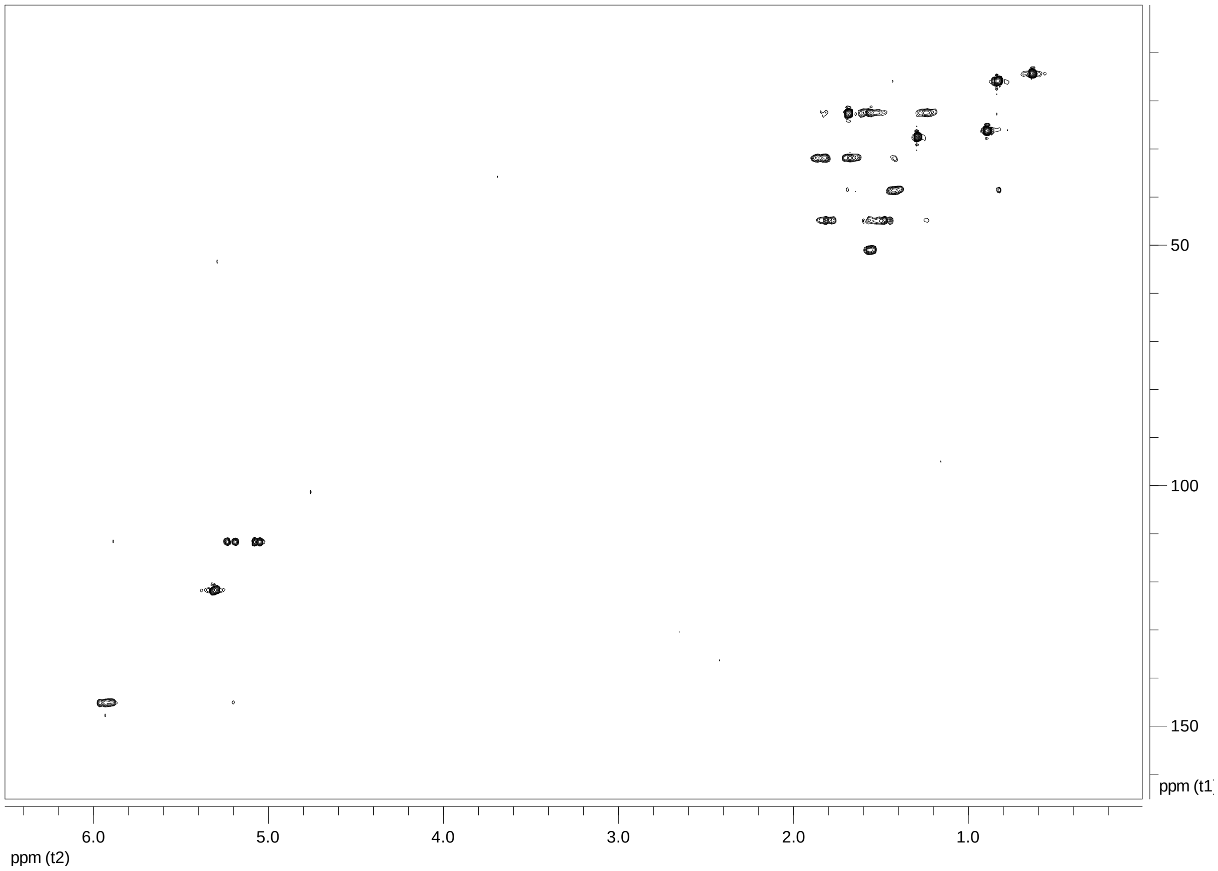

Supplementary Fig. 68. HSQC spectrum (in $CDCl_3$) of compound **2b**.

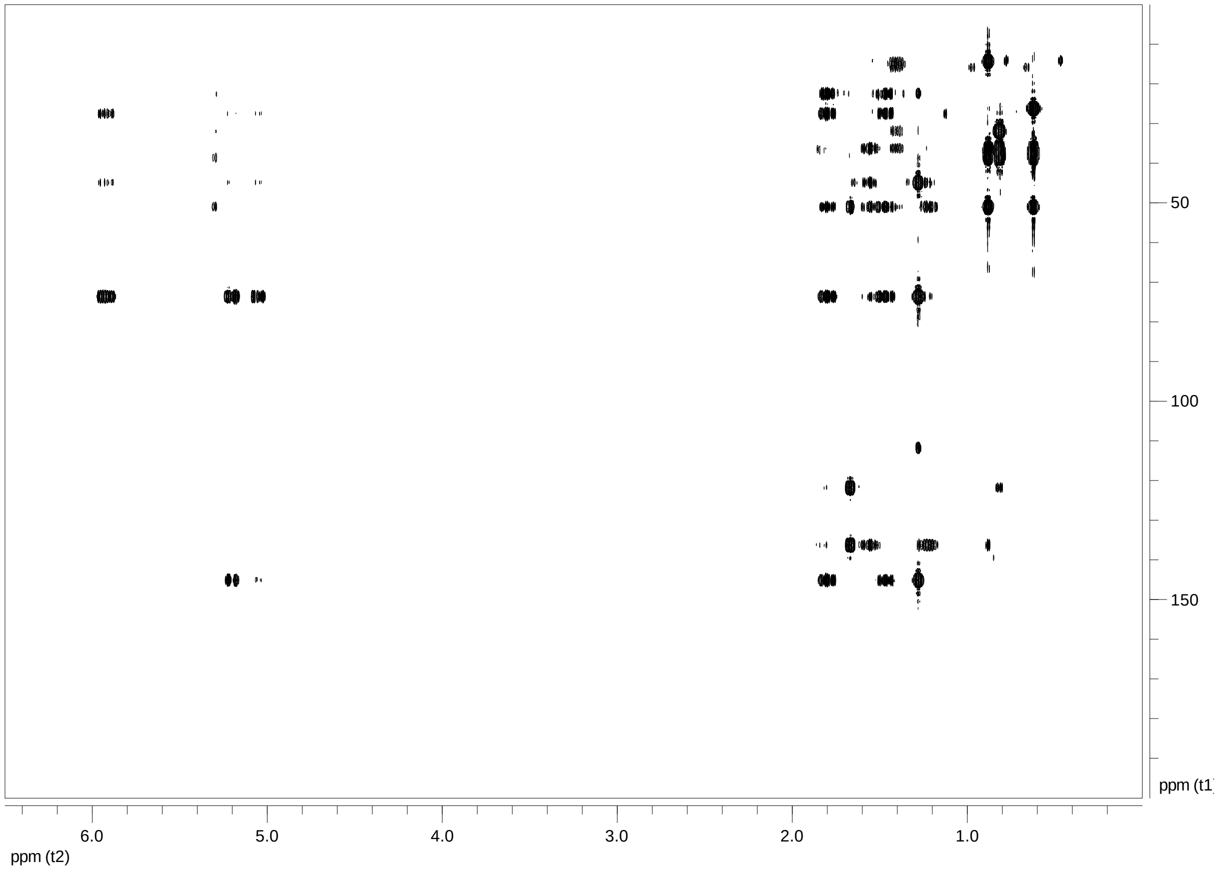

Supplementary Fig. 69. HMBC spectrum (in $CDCl_3$) of compound **2b**.

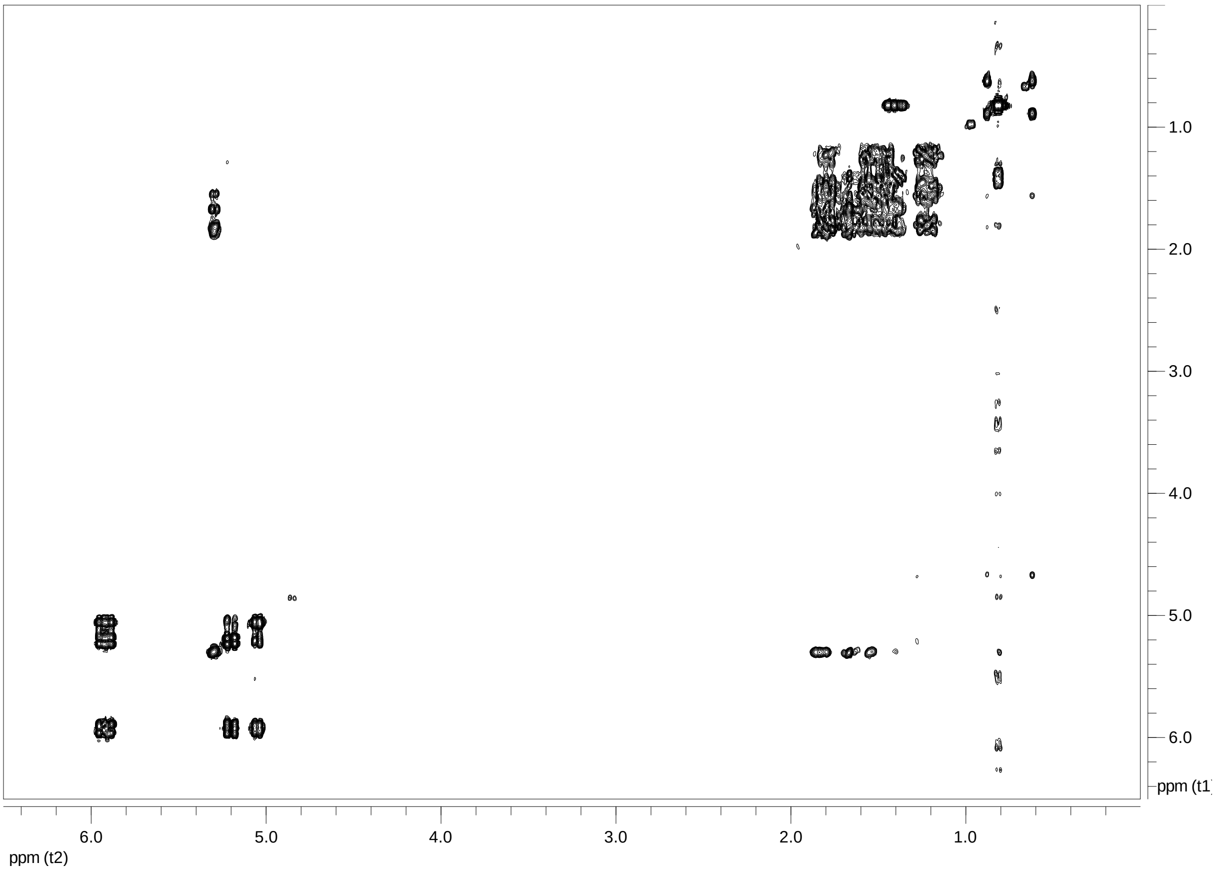

Supplementary Fig. 70. COSY spectrum (in $CDCl_3$) of compound **2b**.

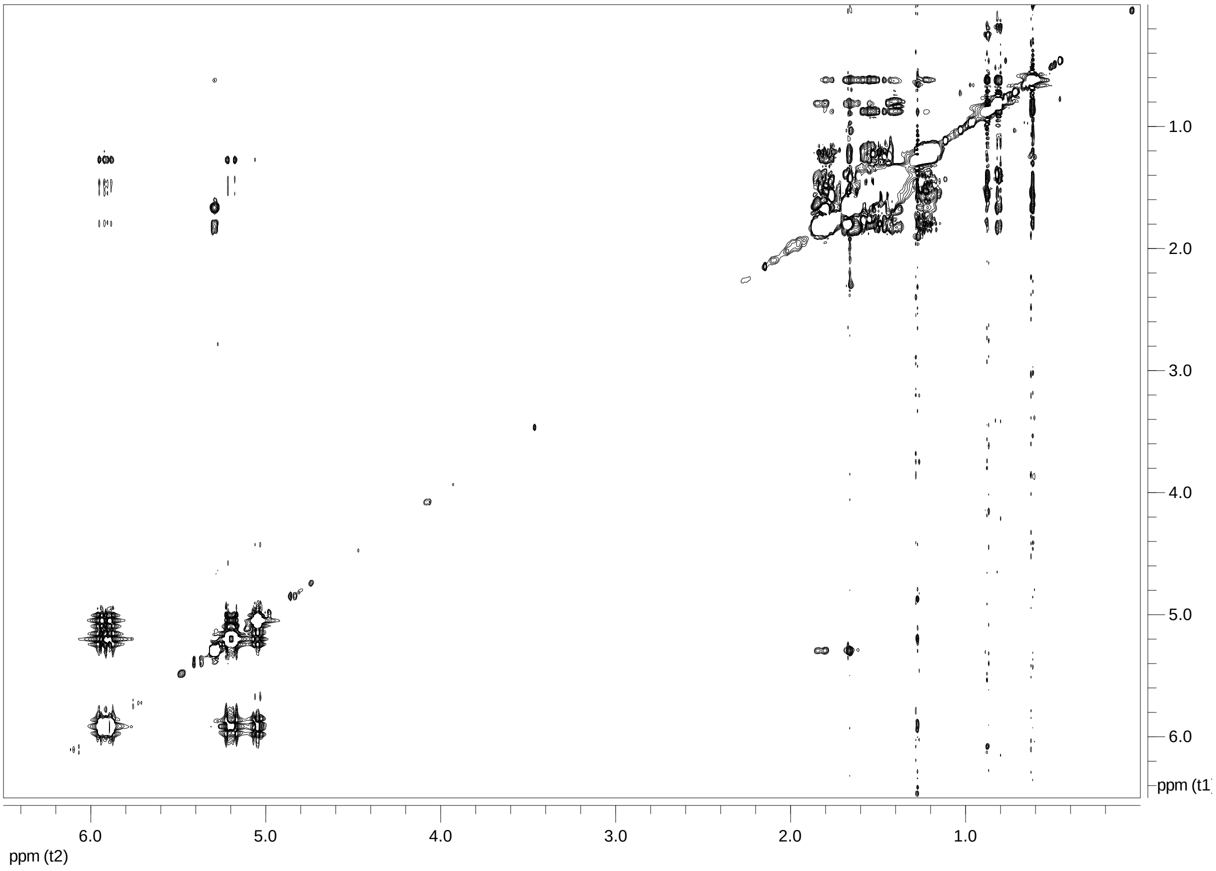

Supplementary Fig. 71. NOESY spectrum (in $CDCl_3$) of compound **2b**.

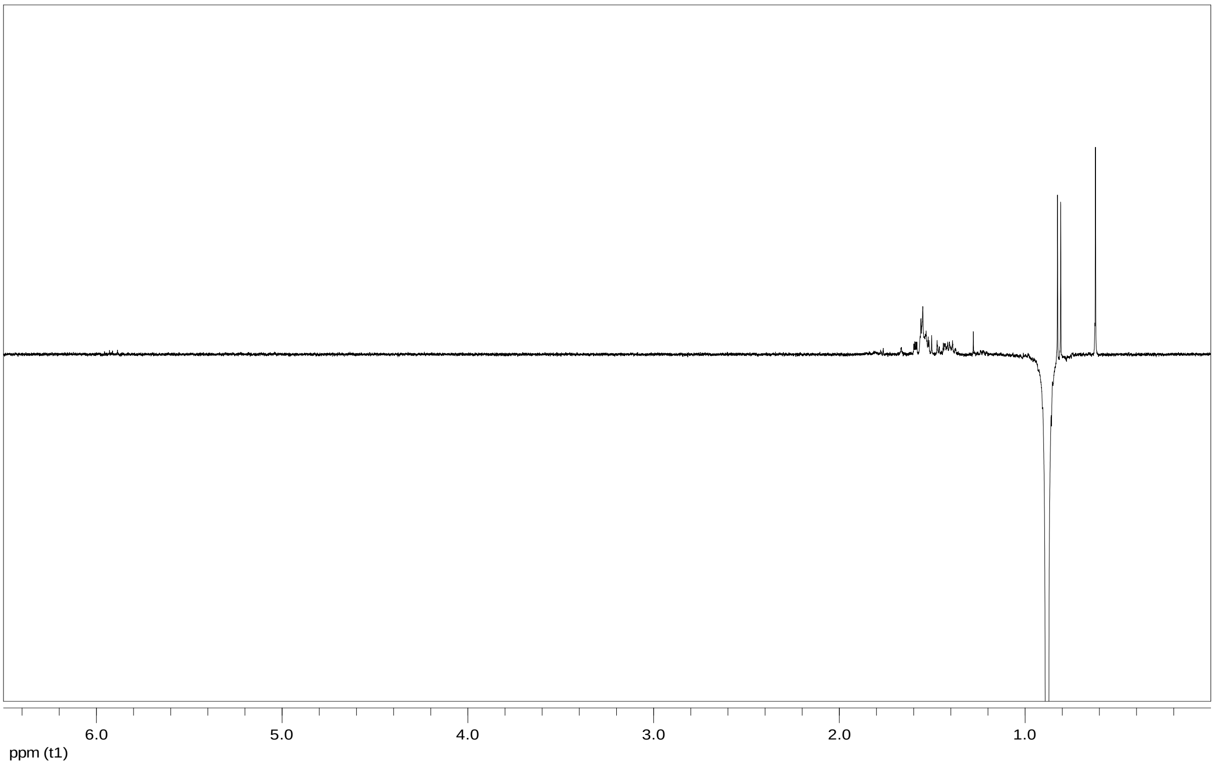

Supplementary Fig. 72. 1D NOE difference spectrum (in $CDCl_3$) of compound **2b** upon irradiation of H_3-12.

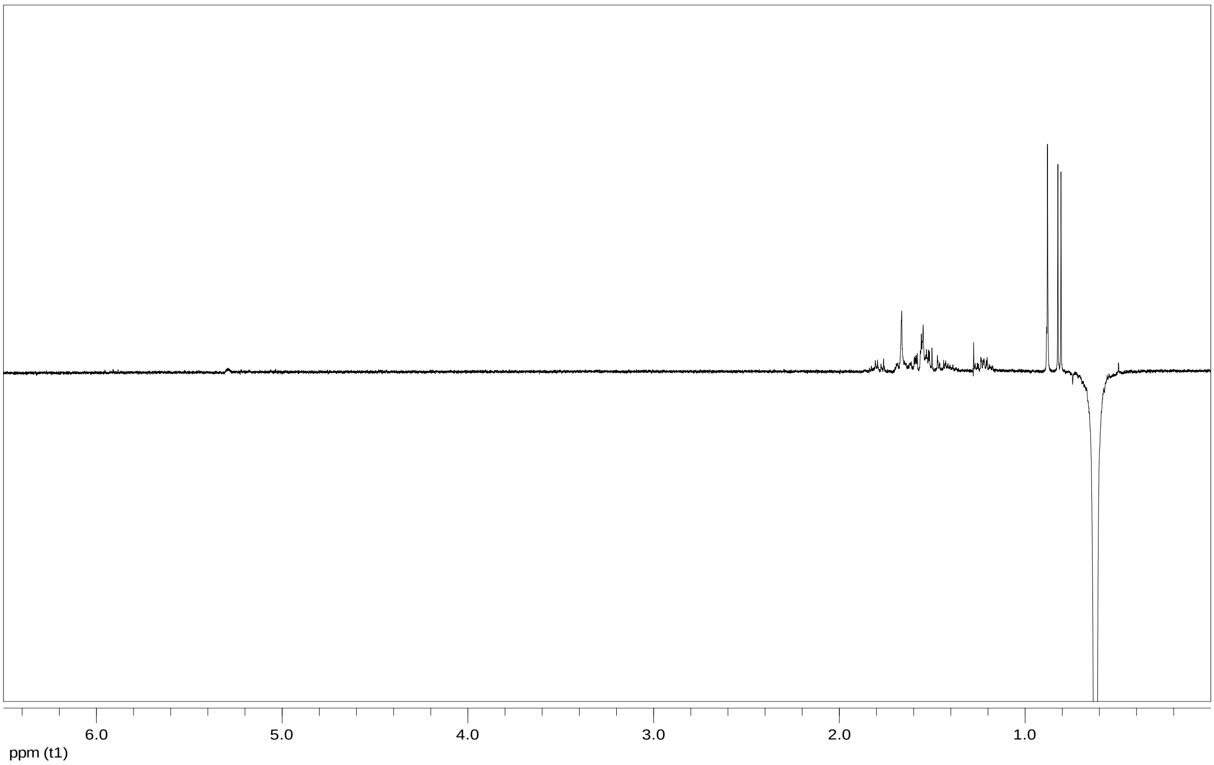

Supplementary Fig. 73. 1D NOE difference spectrum (in $CDCl_3$) of compound **2b** upon irradiation of H_3-13.

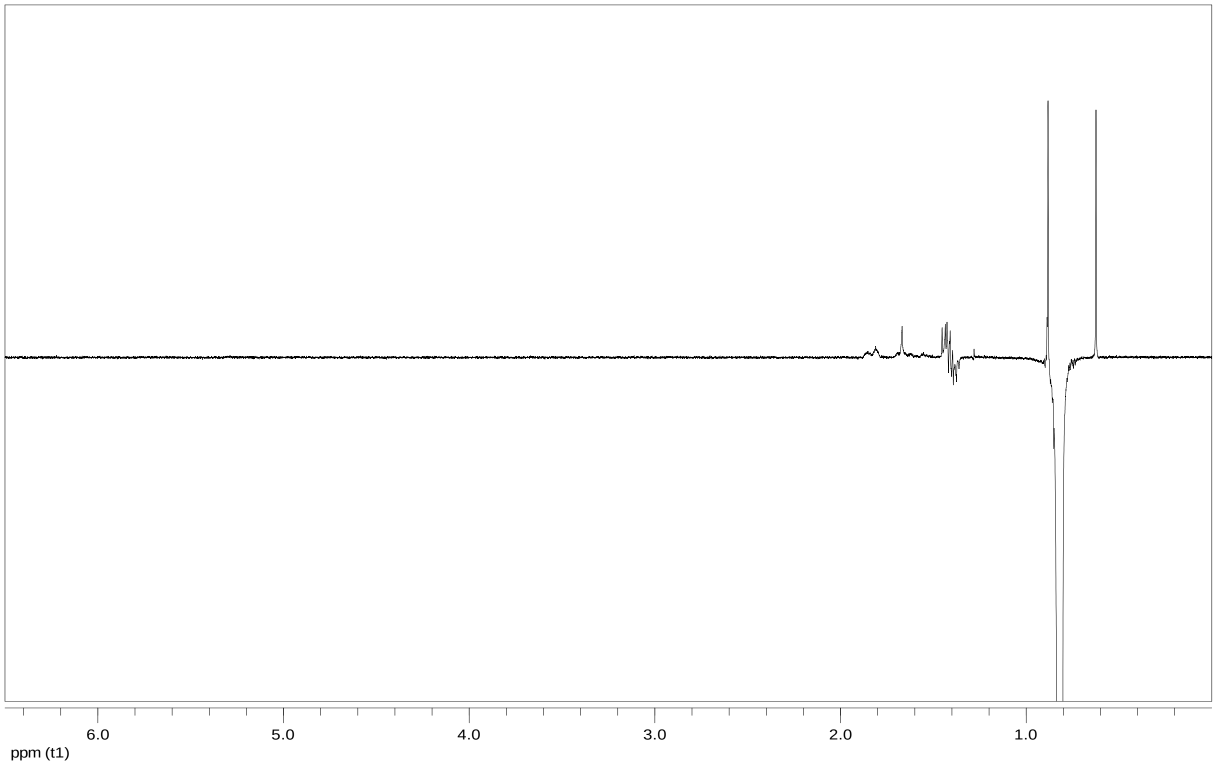

Supplementary Fig. 74. 1D NOE difference spectrum (in $CDCl_3$) of compound **2b** upon irradiation of H_3-14.

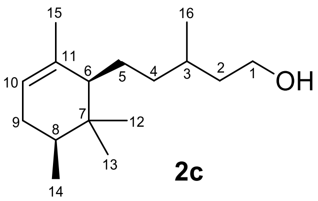

2c

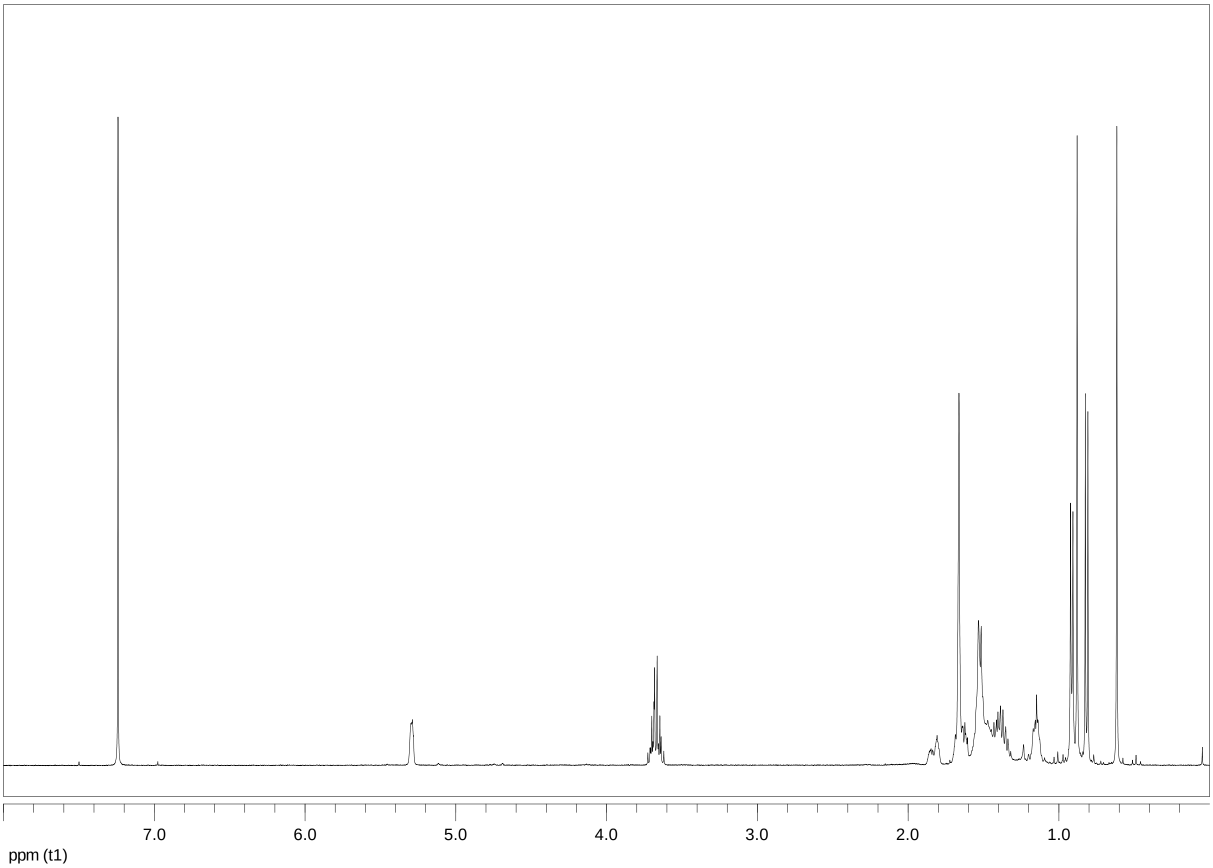

Supplementary Fig. 75. ^{1}H NMR spectrum (in $CDCl_3$) of compound **2c**.

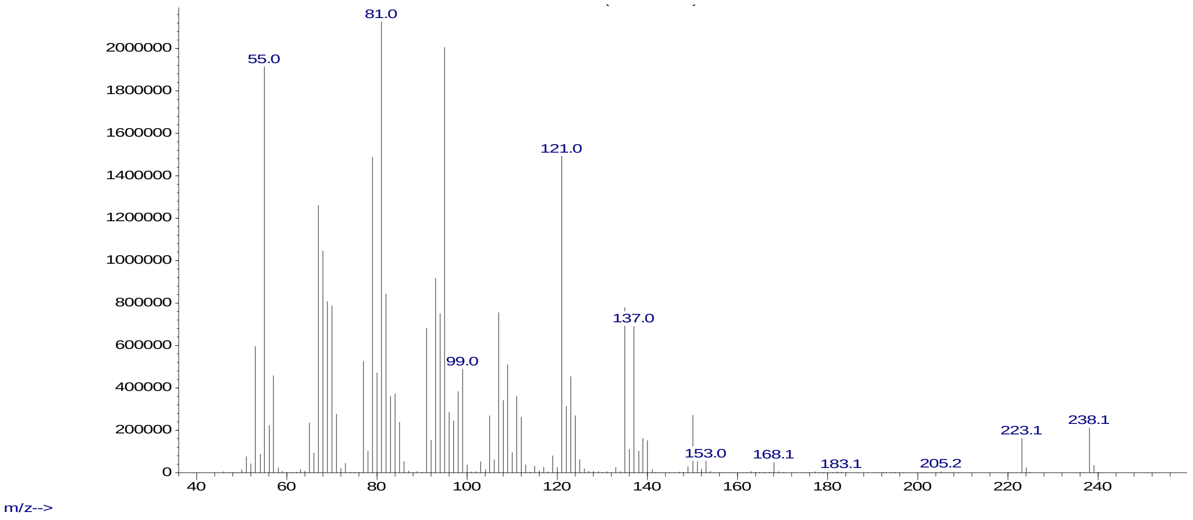

Supplementary Fig. 76. LR-EI-MS spectrum of compound **2c**.

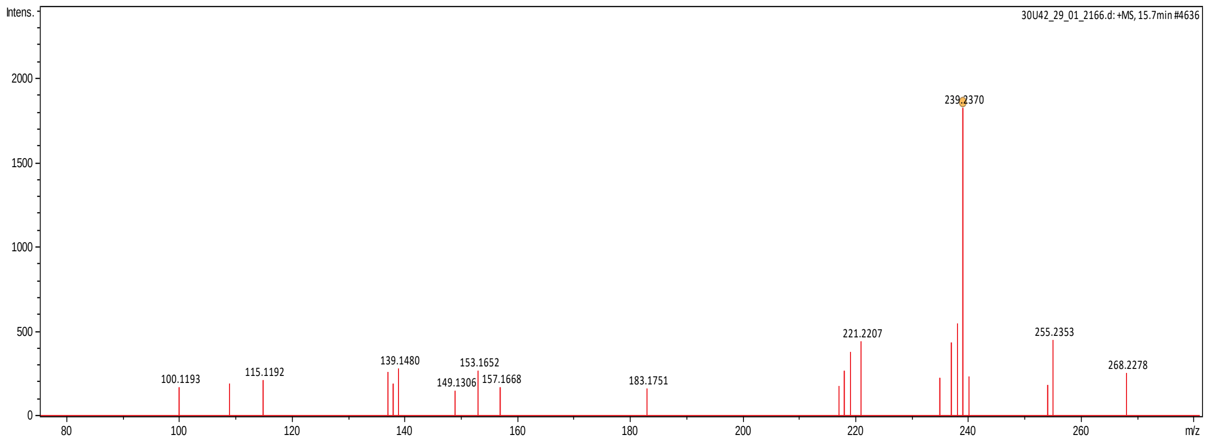

Supplementary Fig. 77. HR-APCI-MS spectrum of compound **2c**.

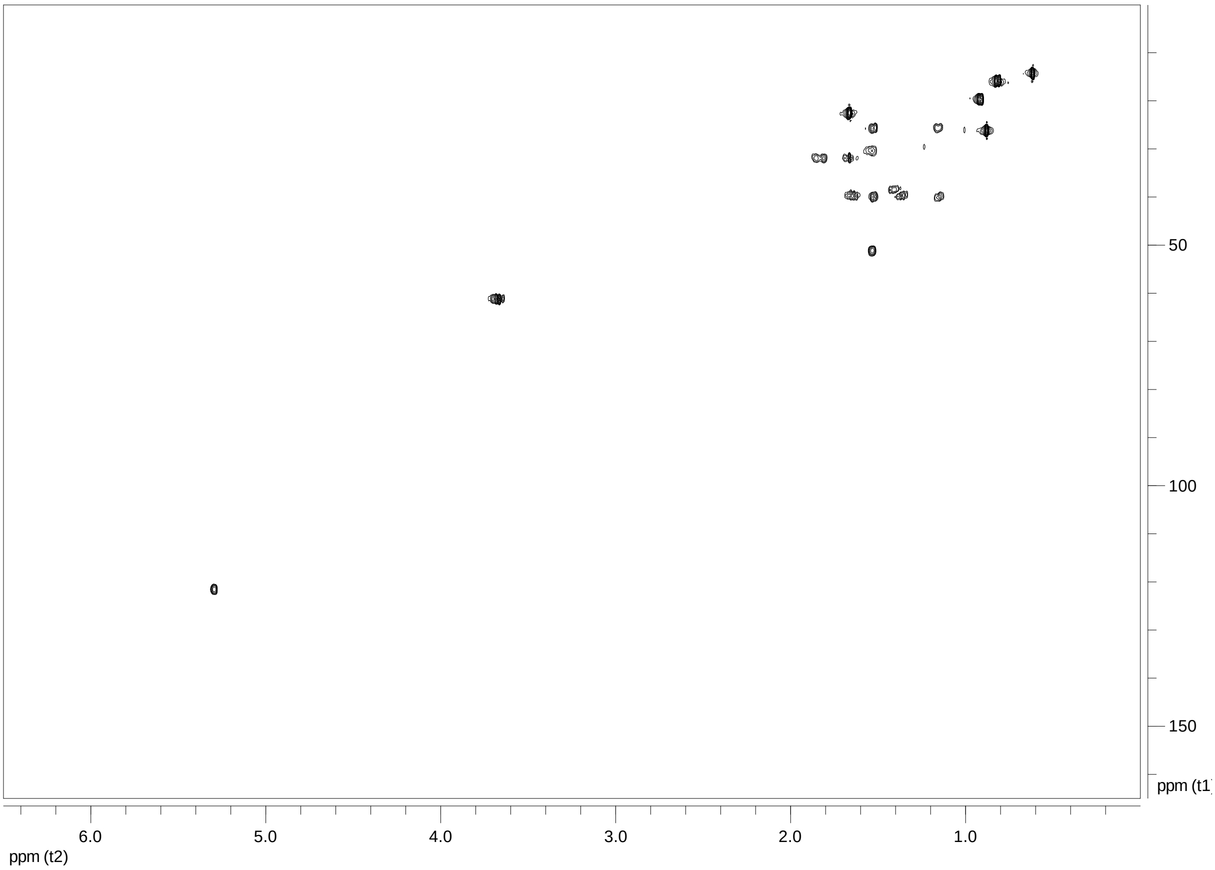

Supplementary Fig. 78. HSQC spectrum (in $CDCl_3$) of compound **2c**.

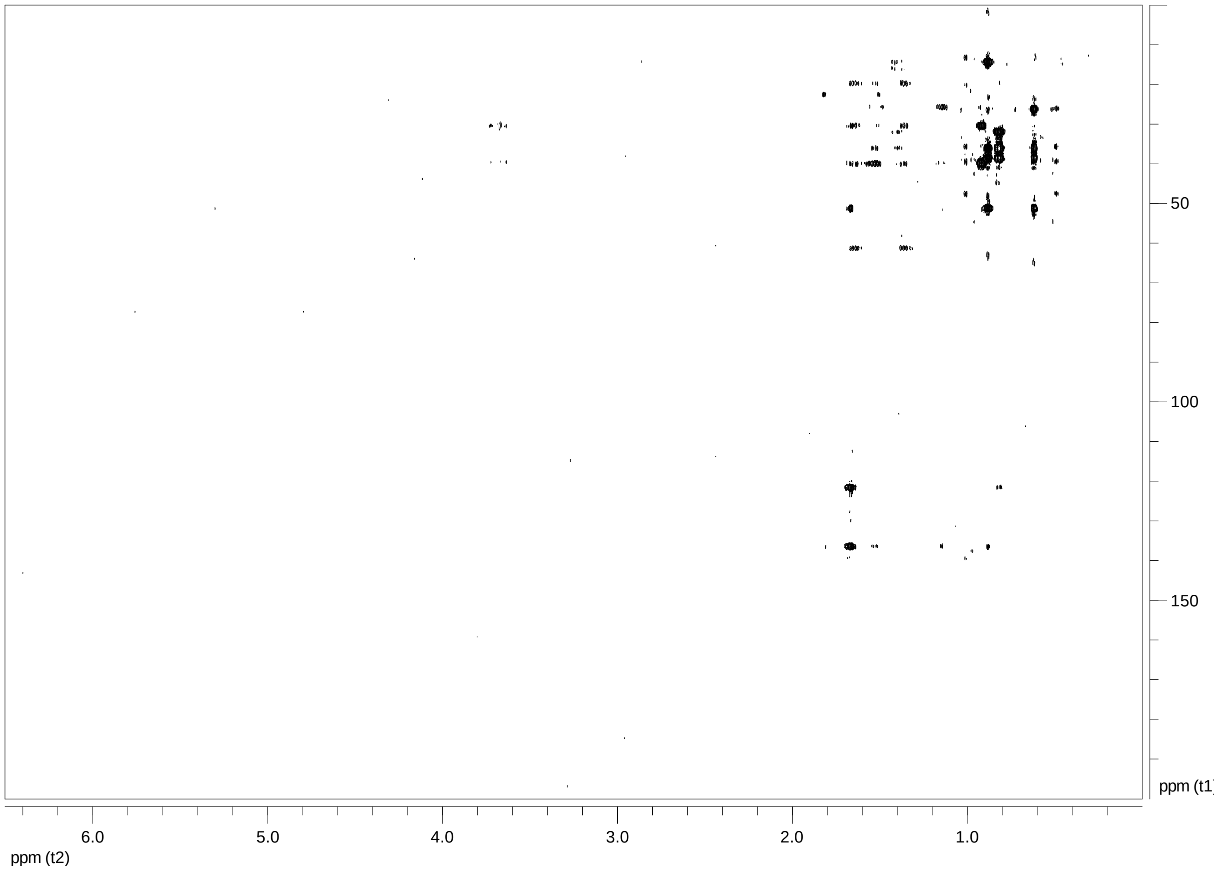

Supplementary Fig. 79. HMBC spectrum (in $CDCl_3$) of compound **2c**.

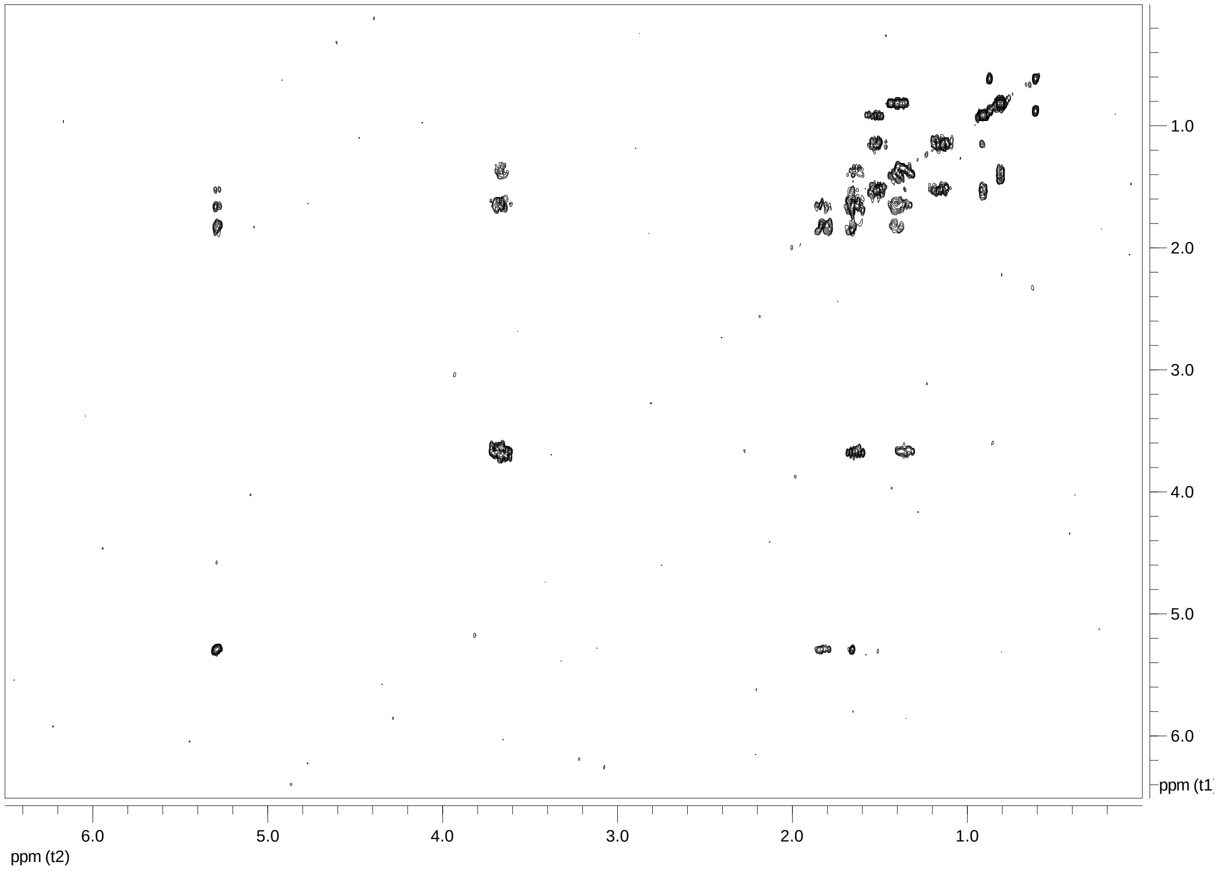

Supplementary Fig. 80. COSY spectrum (in $CDCl_3$) of compound **2c**.

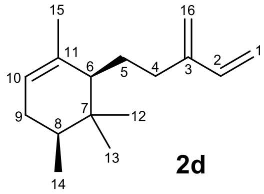

2d

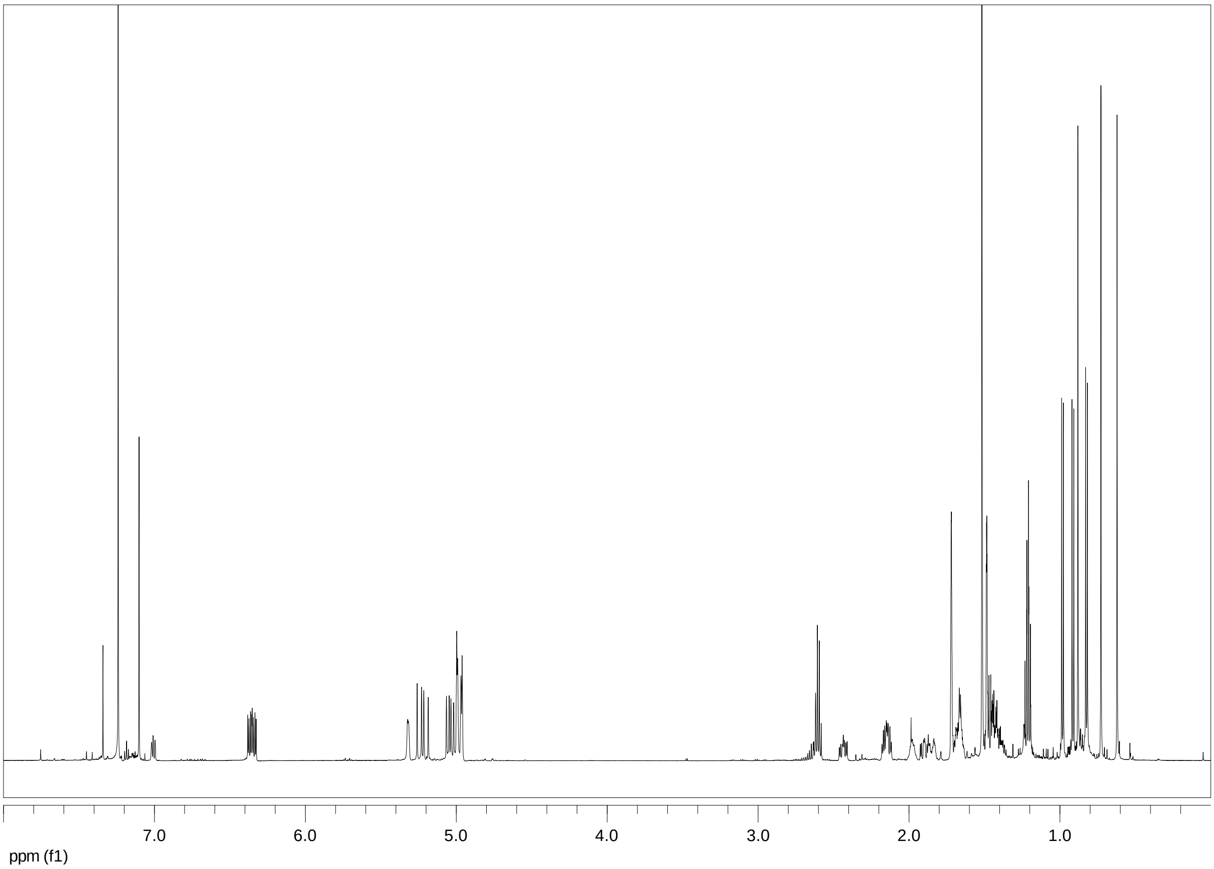

Supplementary Fig. 81. ^{1}H NMR spectrum (in $CDCl_3$) of compound **2d** (as a 1:1 mixture with **1d**).

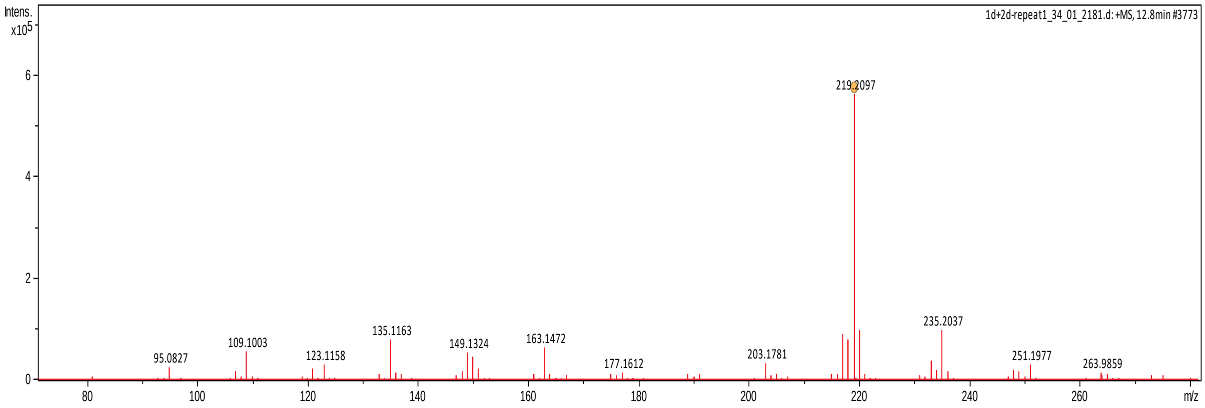

Supplementary Fig. 82. HR-APCI-MS spectrum of compound **2d**.

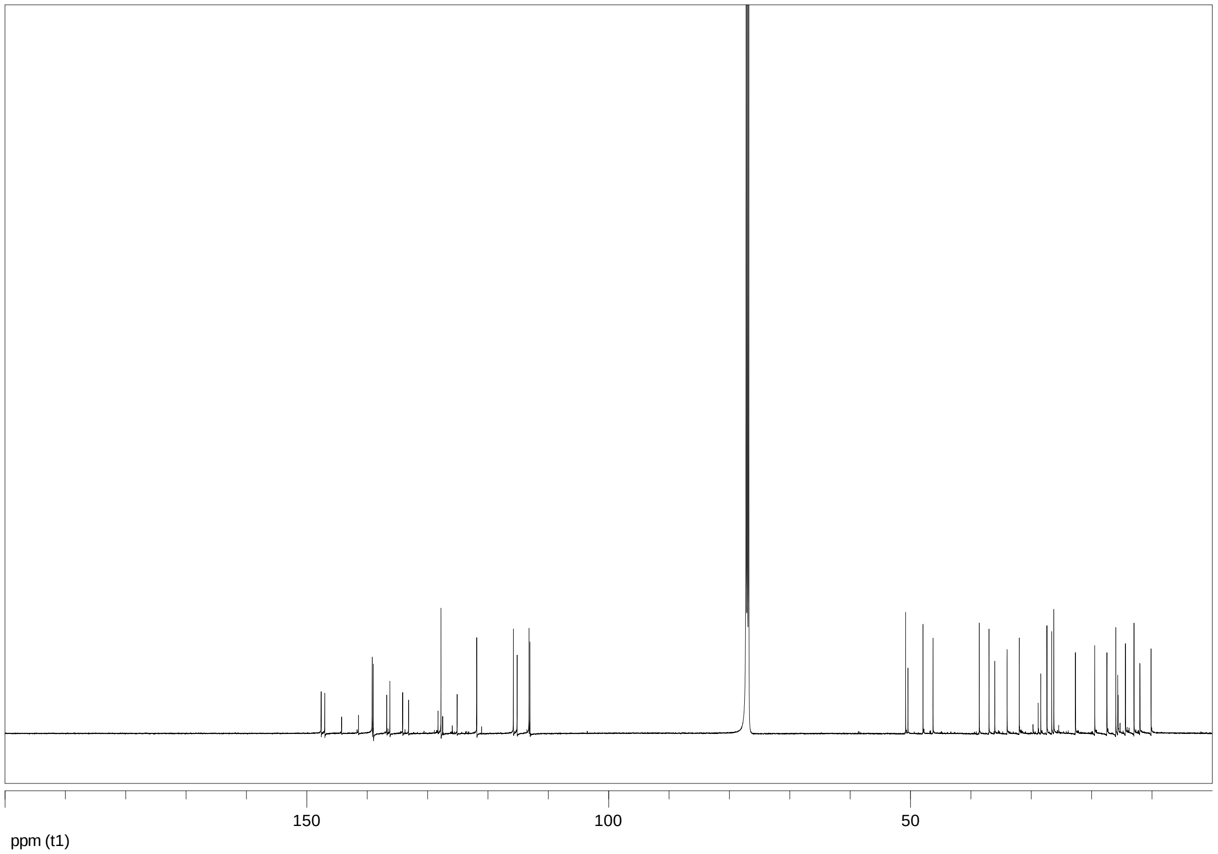

Supplementary Fig. 83. ^{13}C NMR spectrum (in $CDCl_3$) of compound **2d** (as a 1:1 mixture with **1d**).

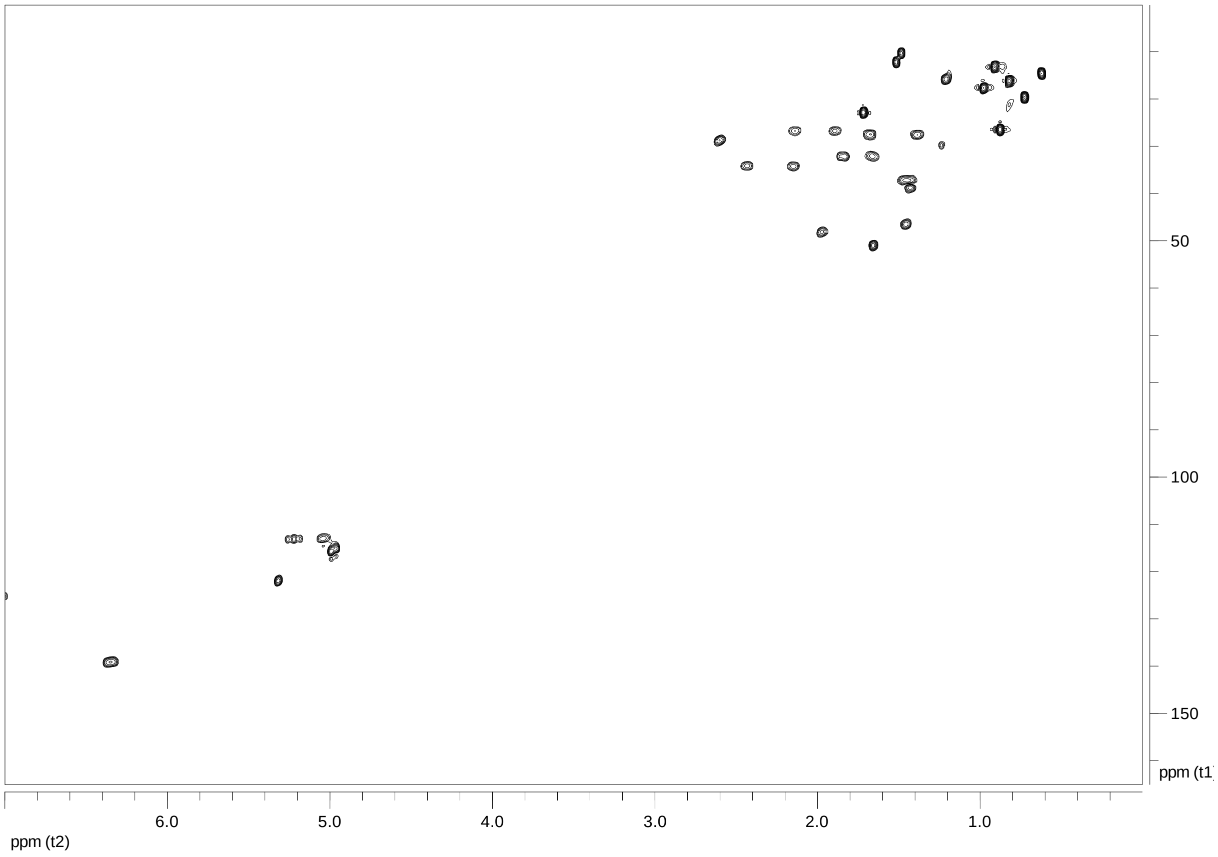

Supplementary Fig. 84. HSQC spectrum (in $CDCl_3$) of compound **2d** (as a 1:1 mixture with **1d**).

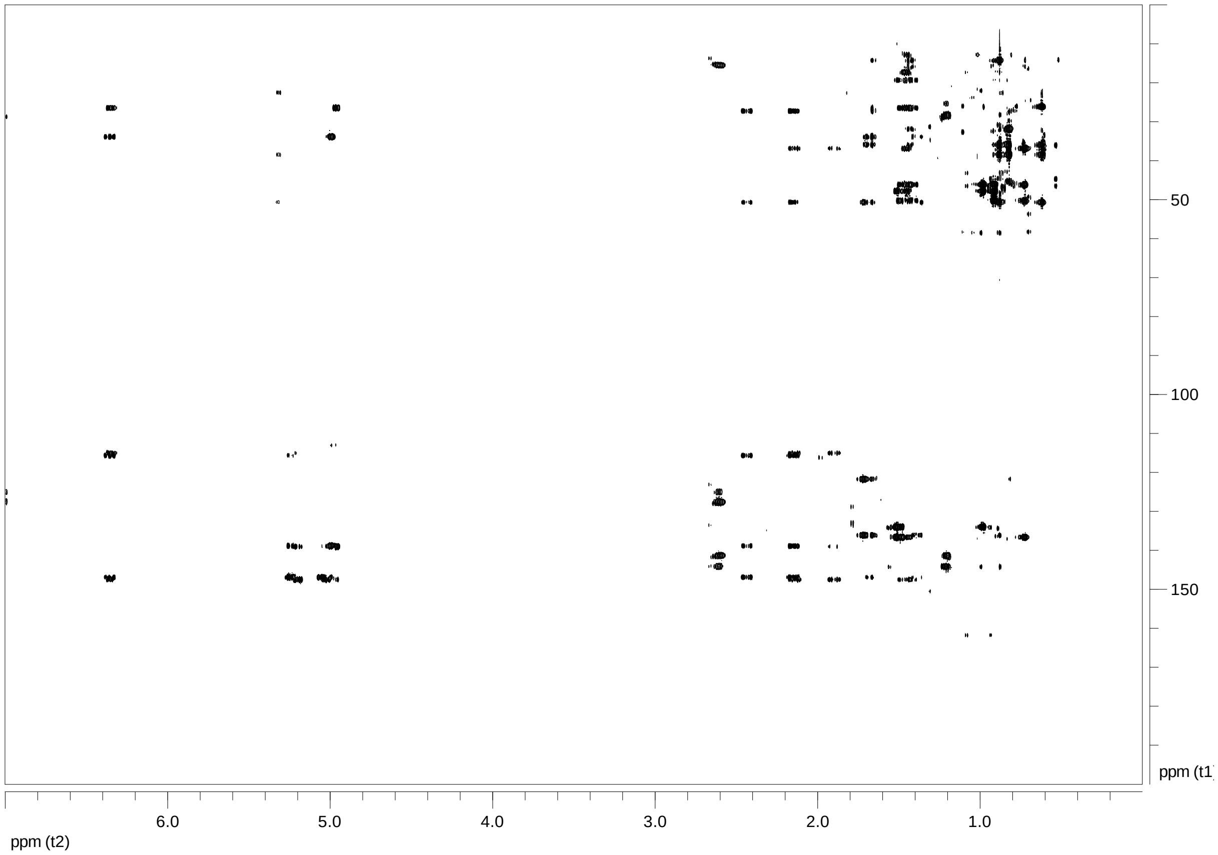

Supplementary Fig. 85. HMBC spectrum (in $CDCl_3$) of compound **2d** (as a 1:1 mixture with **1d**).

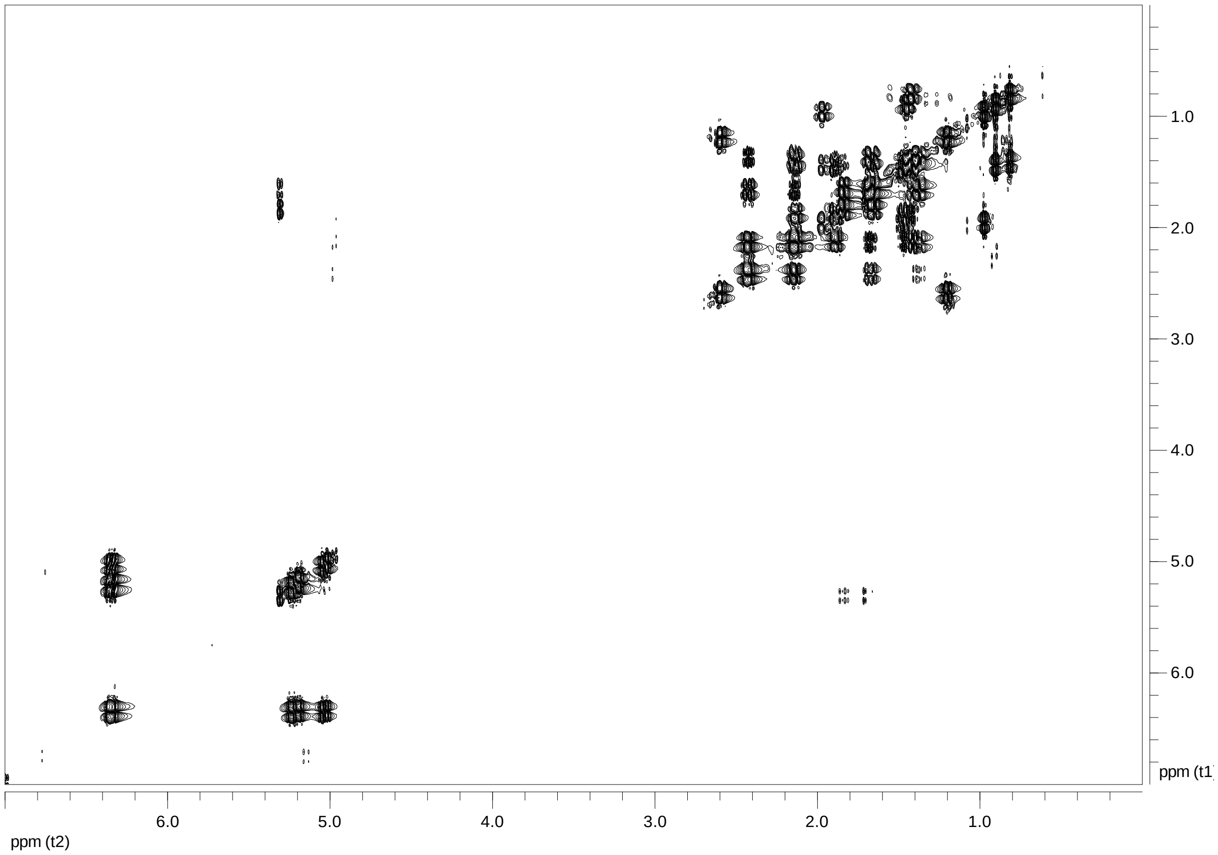

Supplementary Fig. 86. COSY spectrum (in $CDCl_3$) of compound **2d** (as a 1:1 mixture with **1d**).

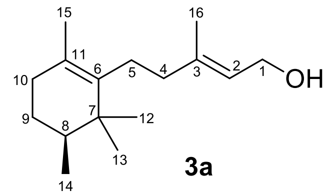

3a

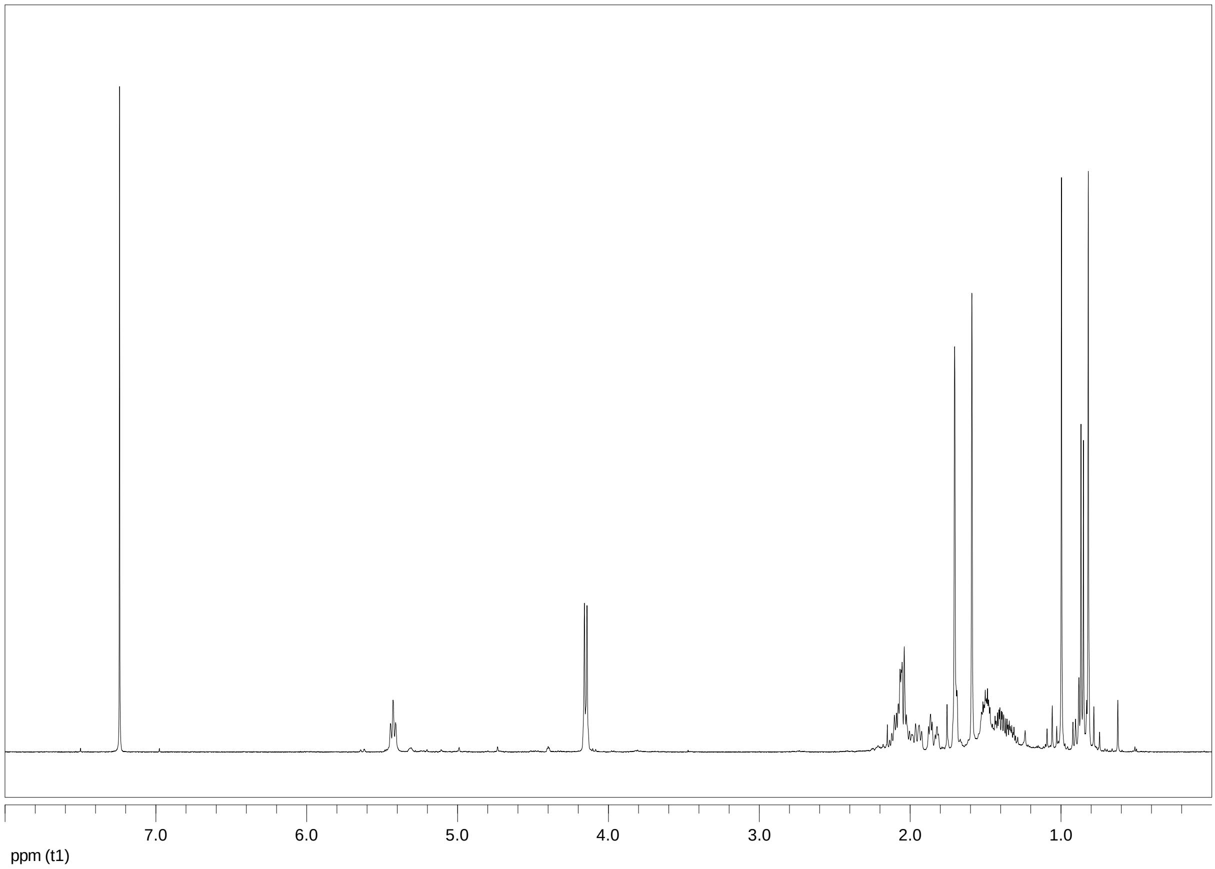

Supplementary Fig. 87. ^{1}H NMR spectrum (in $CDCl_3$) of compound **3a**.

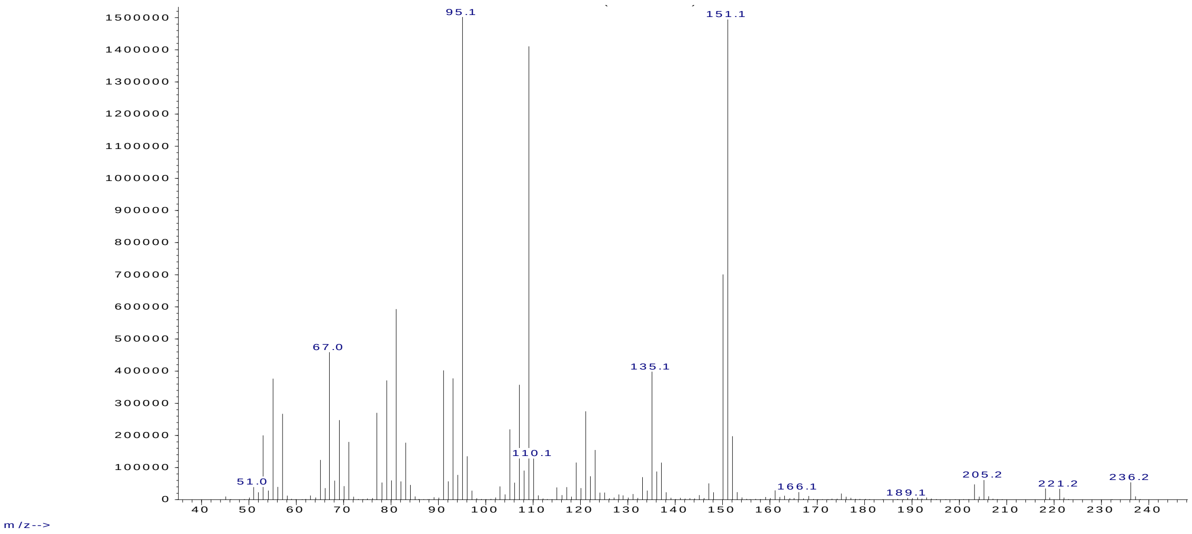

Supplementary Fig. 88. LR-EI-MS spectrum of compound **3a**.

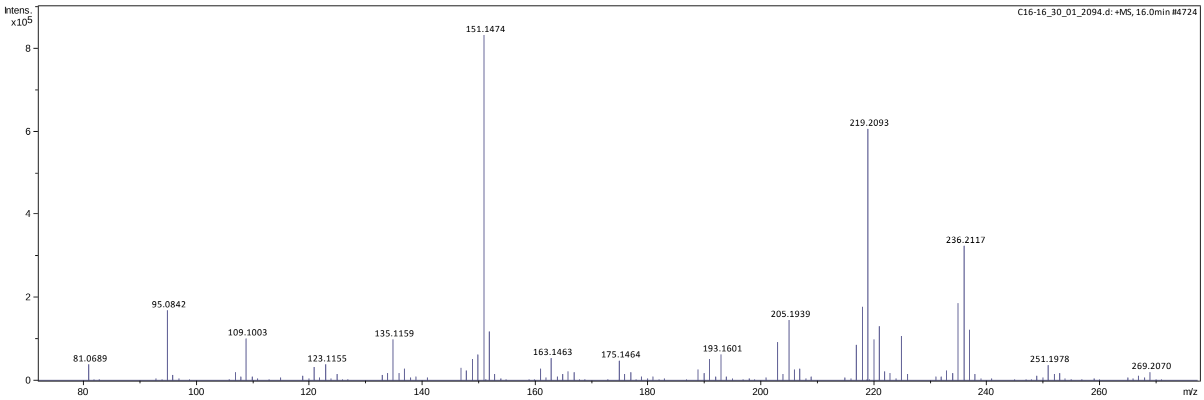

Supplementary Fig. 89. HR-APCI-MS spectrum of compound **3a**.

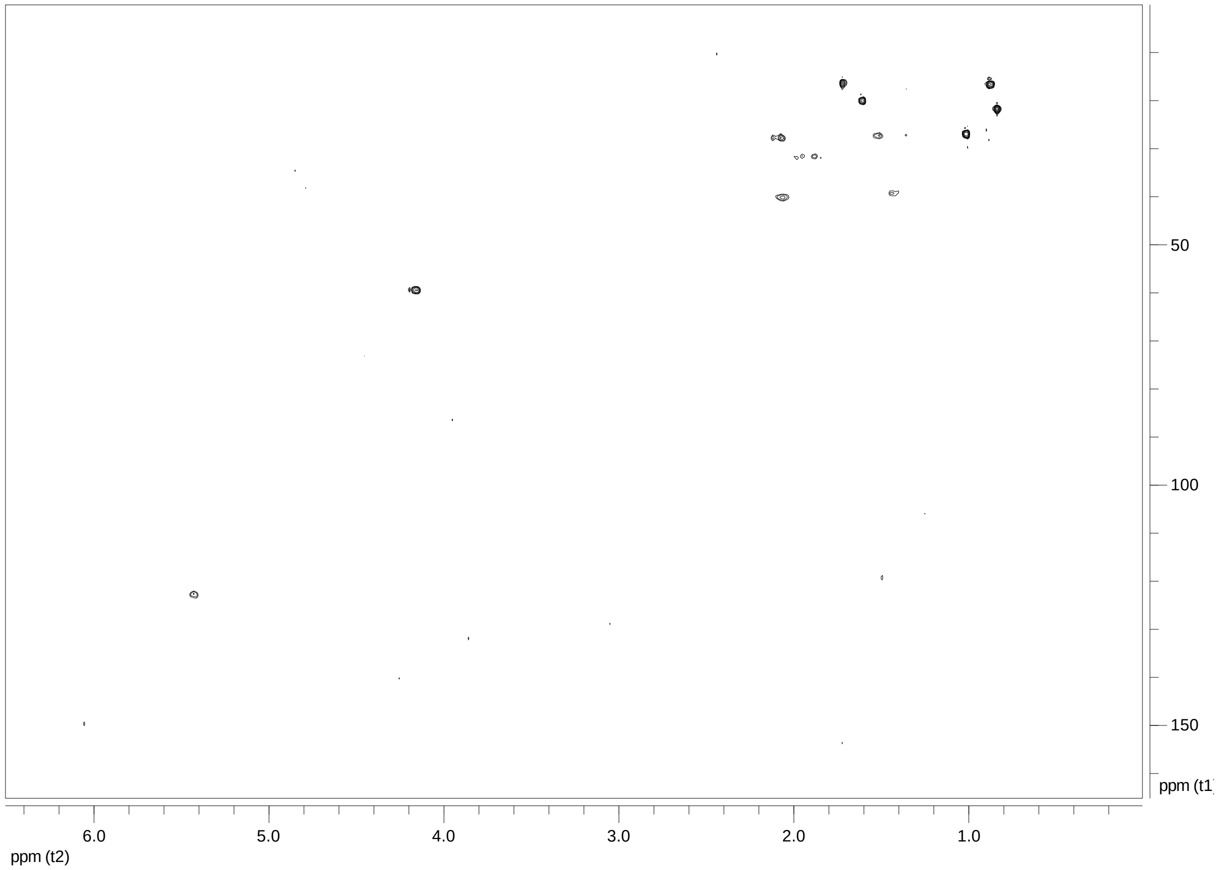

Supplementary Fig. 90. HSQC spectrum (in $CDCl_3$) of compound **3a**.

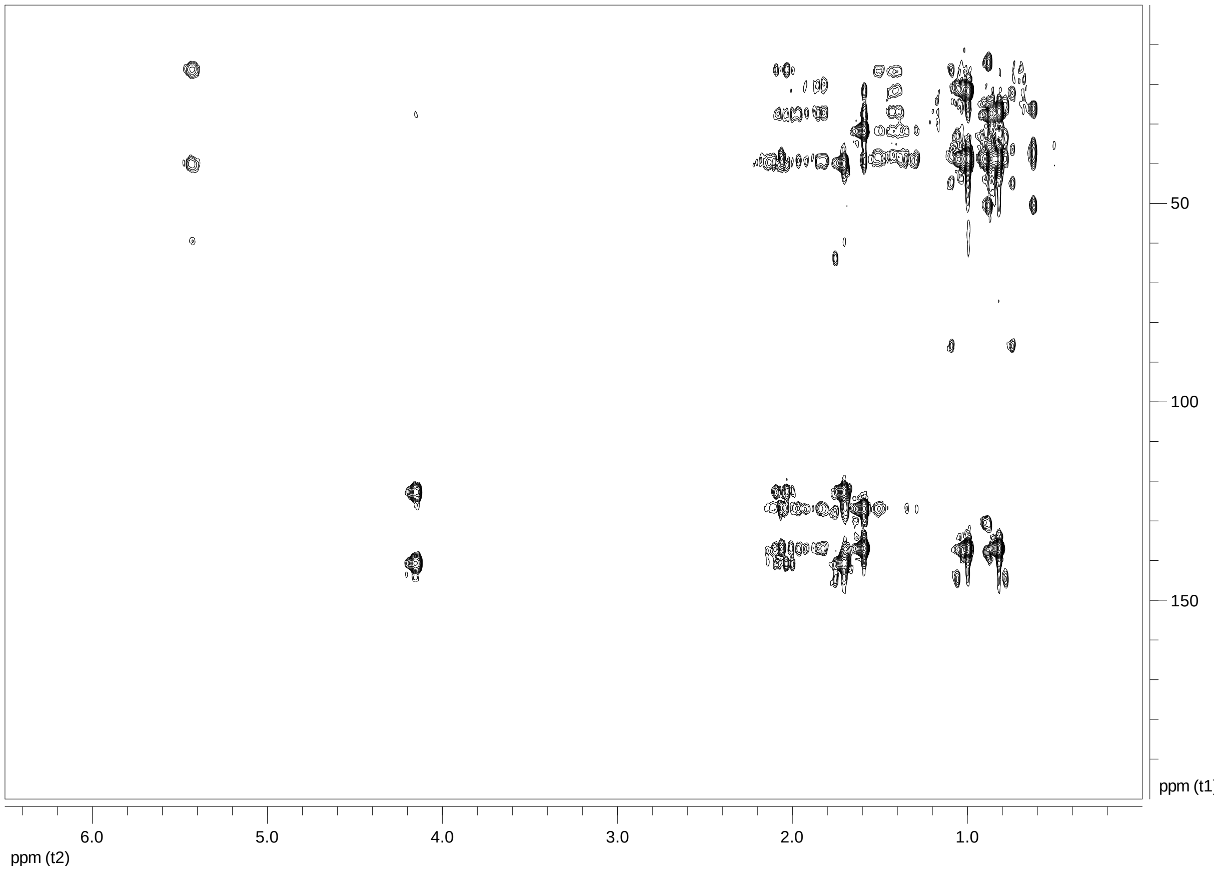

Supplementary Fig. 91. HMBC spectrum (in $CDCl_3$) of compound **3a**.

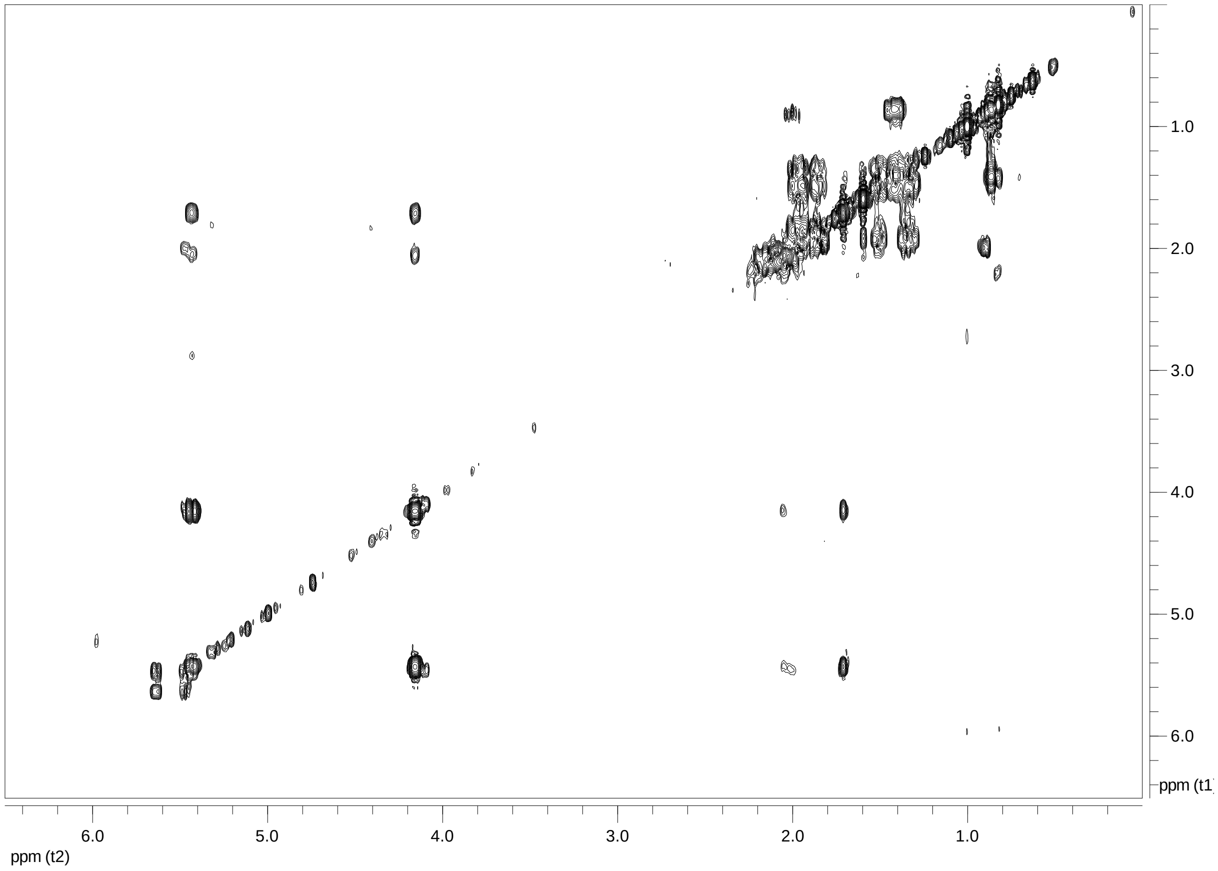

Supplementary Fig. 92. COSY spectrum (in $CDCl_3$) of compound **3a**.

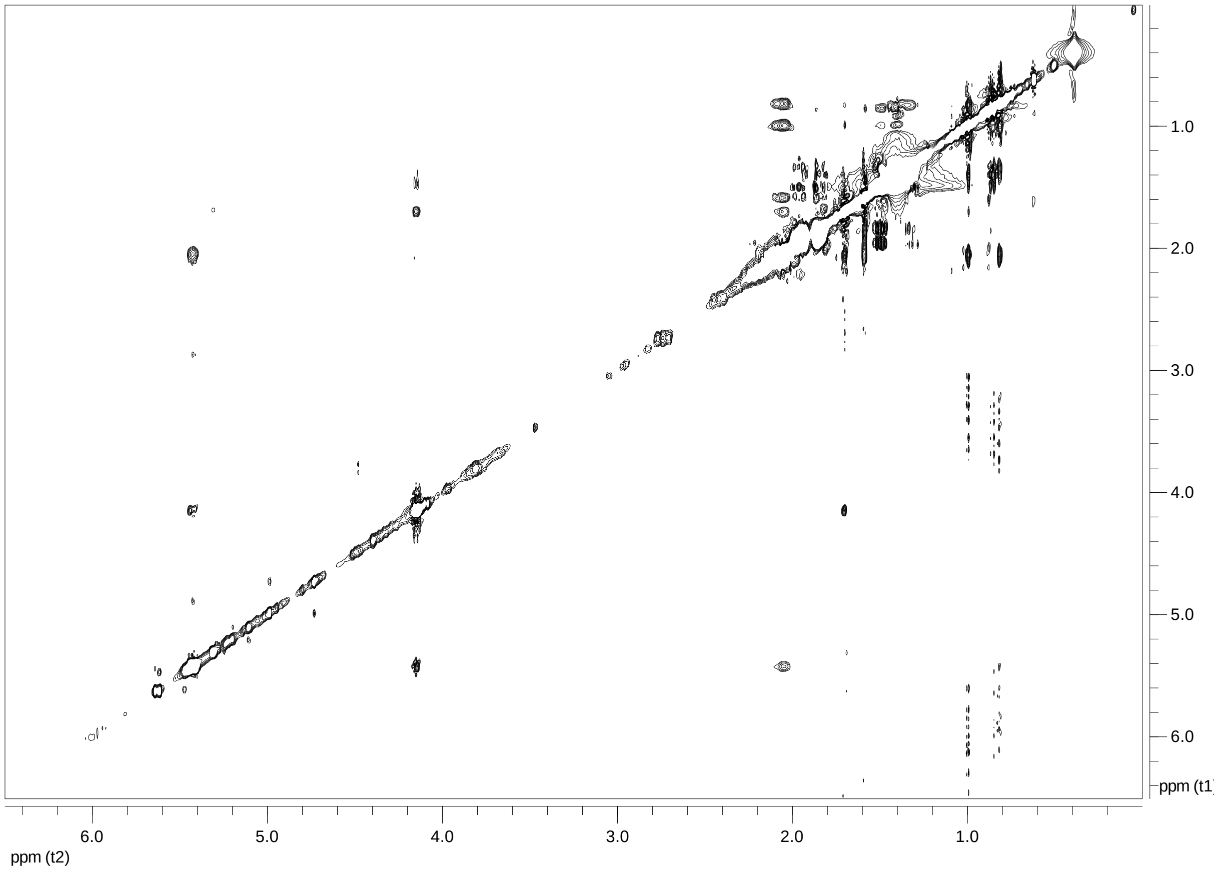

Supplementary Fig. 93. NOESY spectrum (in $CDCl_3$) of compound **3a**.

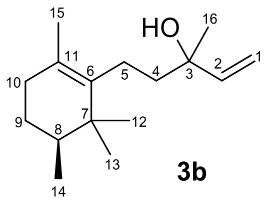

3b

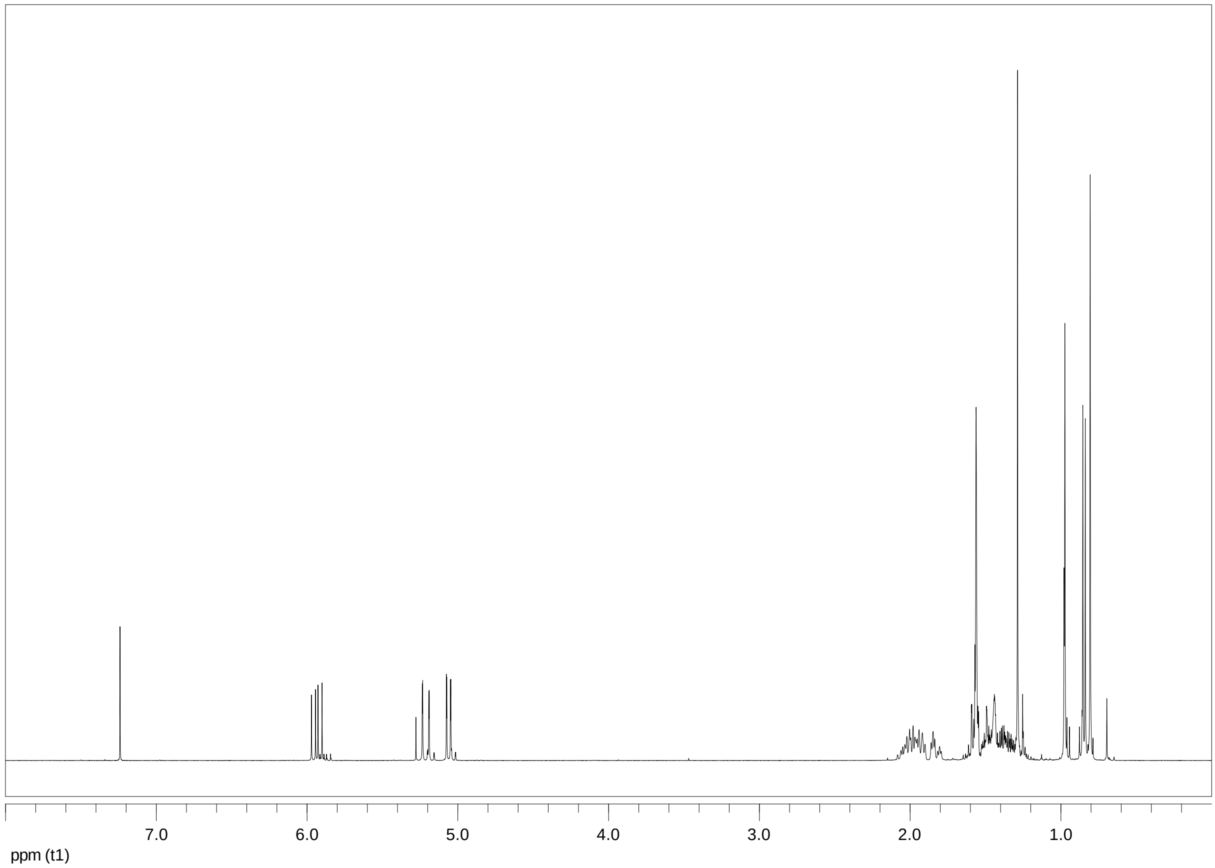

Supplementary Fig. 94. ^{1}H NMR spectrum (in $CDCl_3$) of compound **3b**.

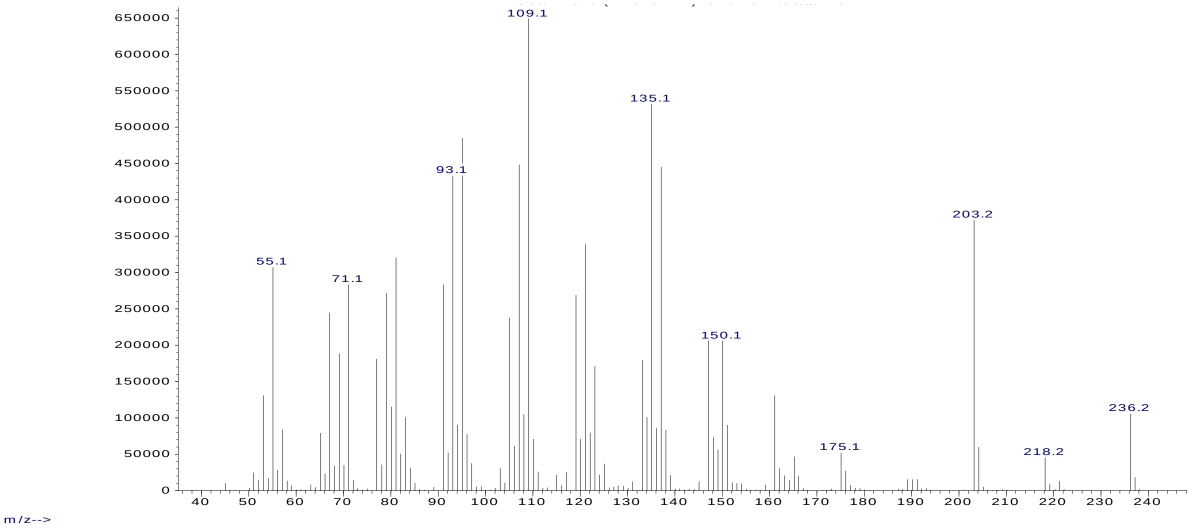

Supplementary Fig. 95. LR-EI-MS spectrum of compound **3b**.

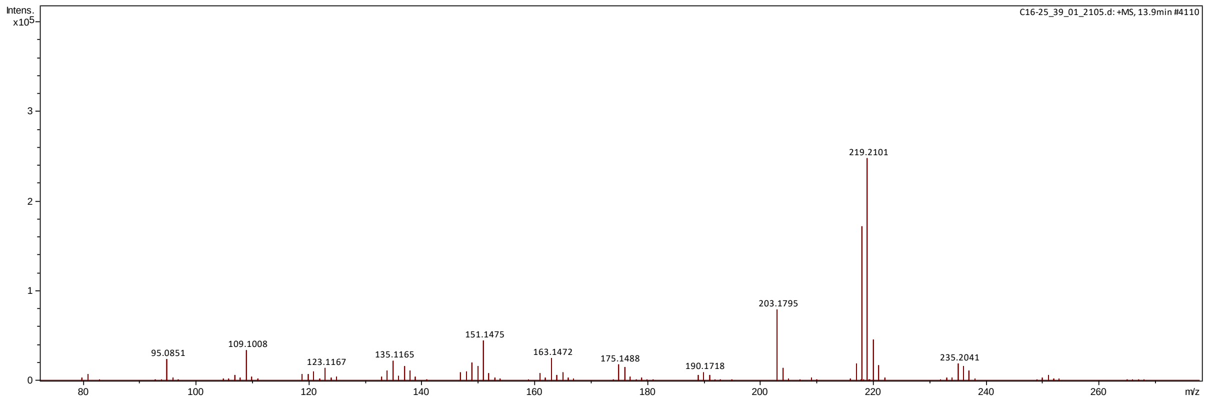

Supplementary Fig. 96. HR-APCI-MS spectrum of compound **3b**.

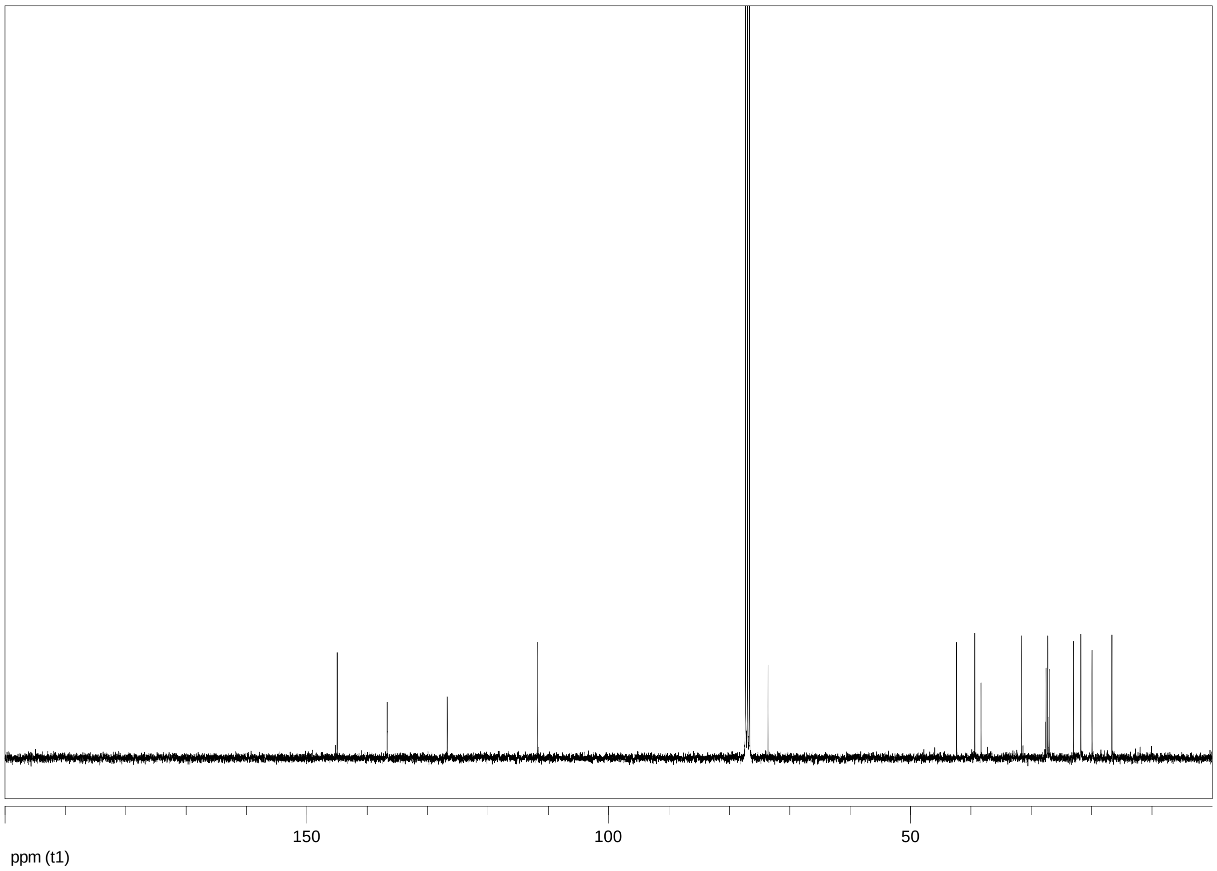

Supplementary Fig. 97. ^{13}C NMR spectrum (in $CDCl_3$) of compound **3b**.

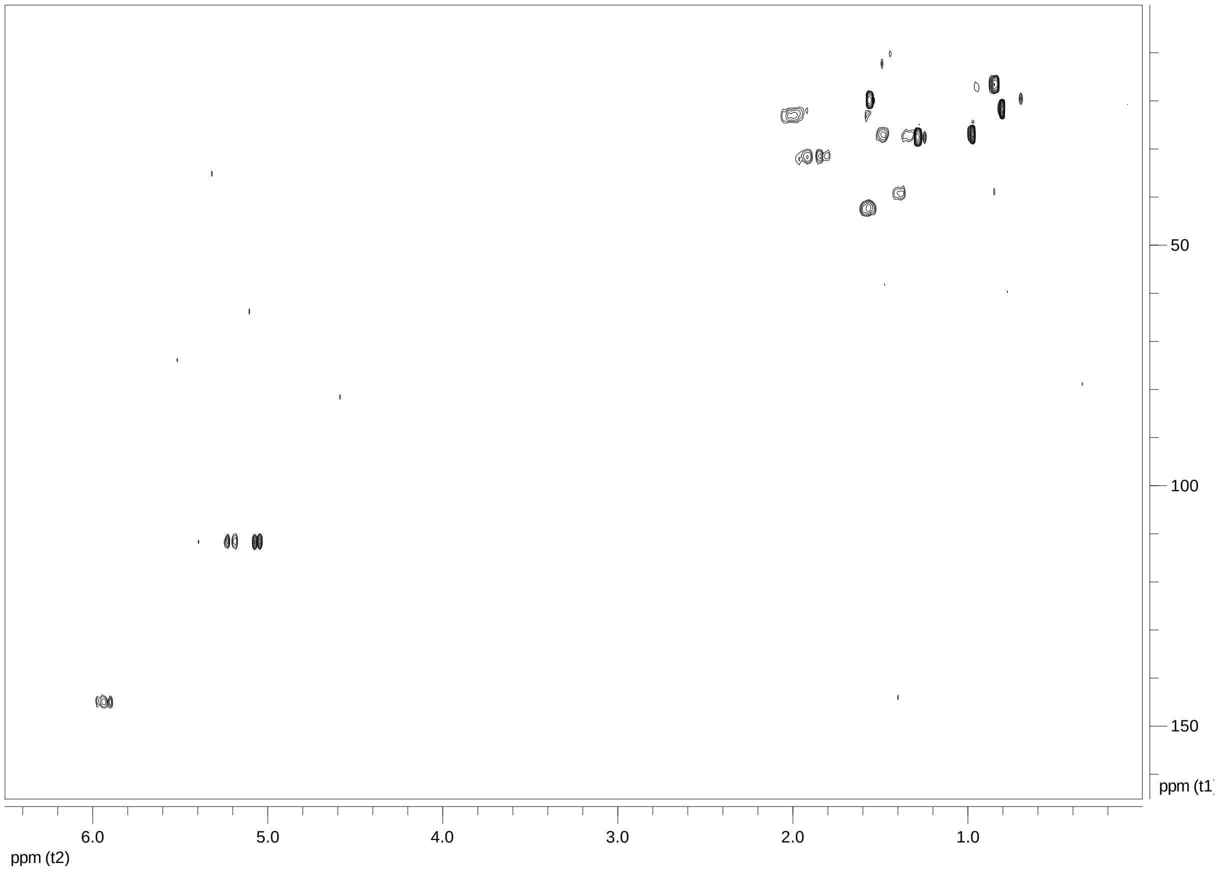

Supplementary Fig. 98. HSQC spectrum (in $CDCl_3$) of compound **3b**.

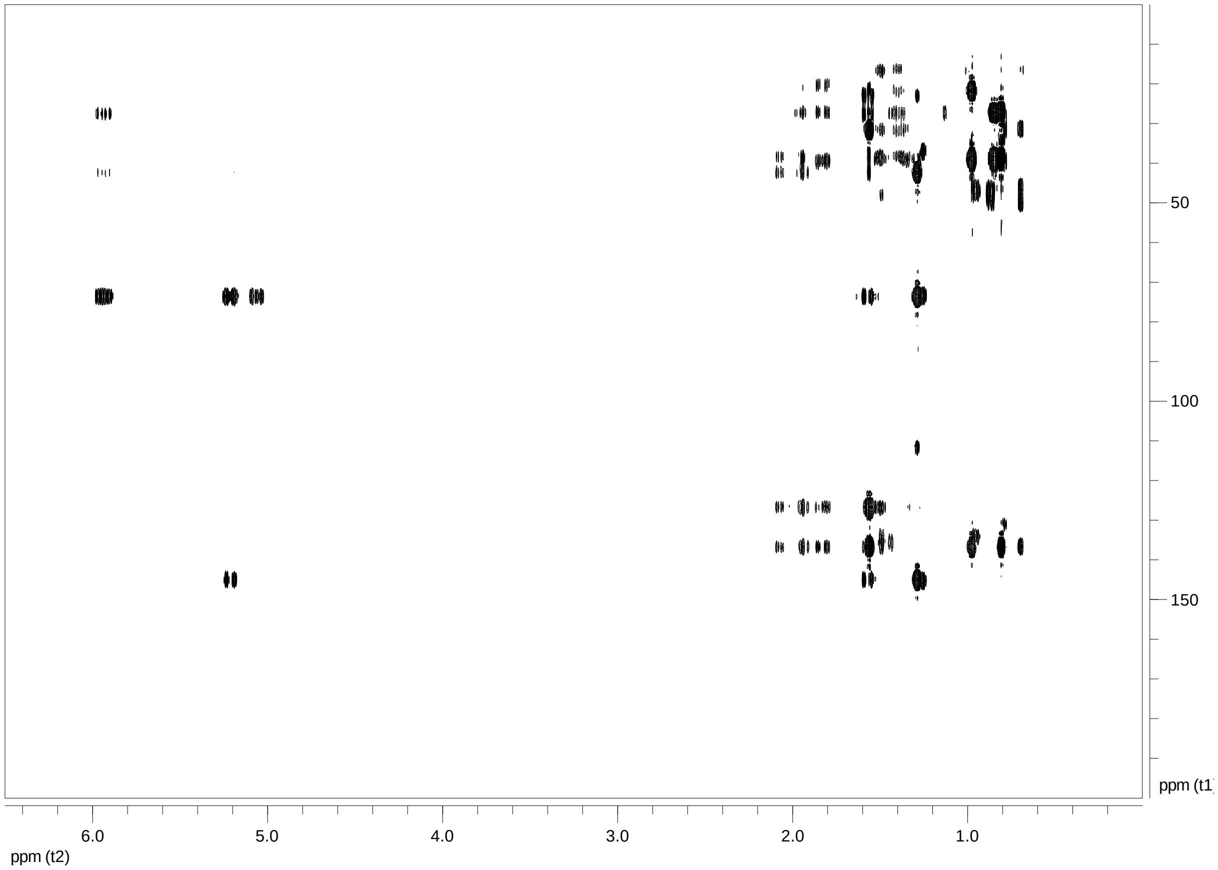

Supplementary Fig. 99. HMBC spectrum (in $CDCl_3$) of compound **3b**.

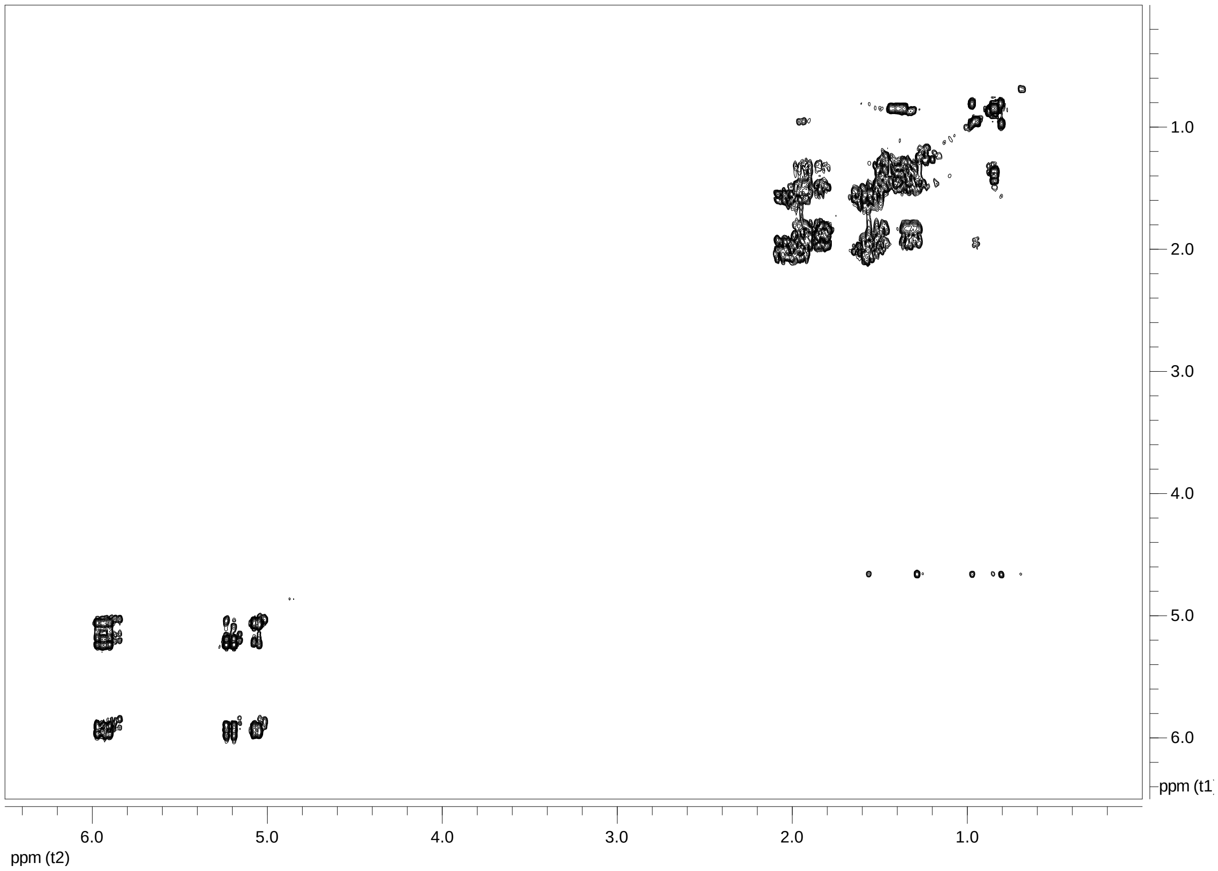

Supplementary Fig. 100. COSY spectrum (in $CDCl_3$) of compound **3b**.

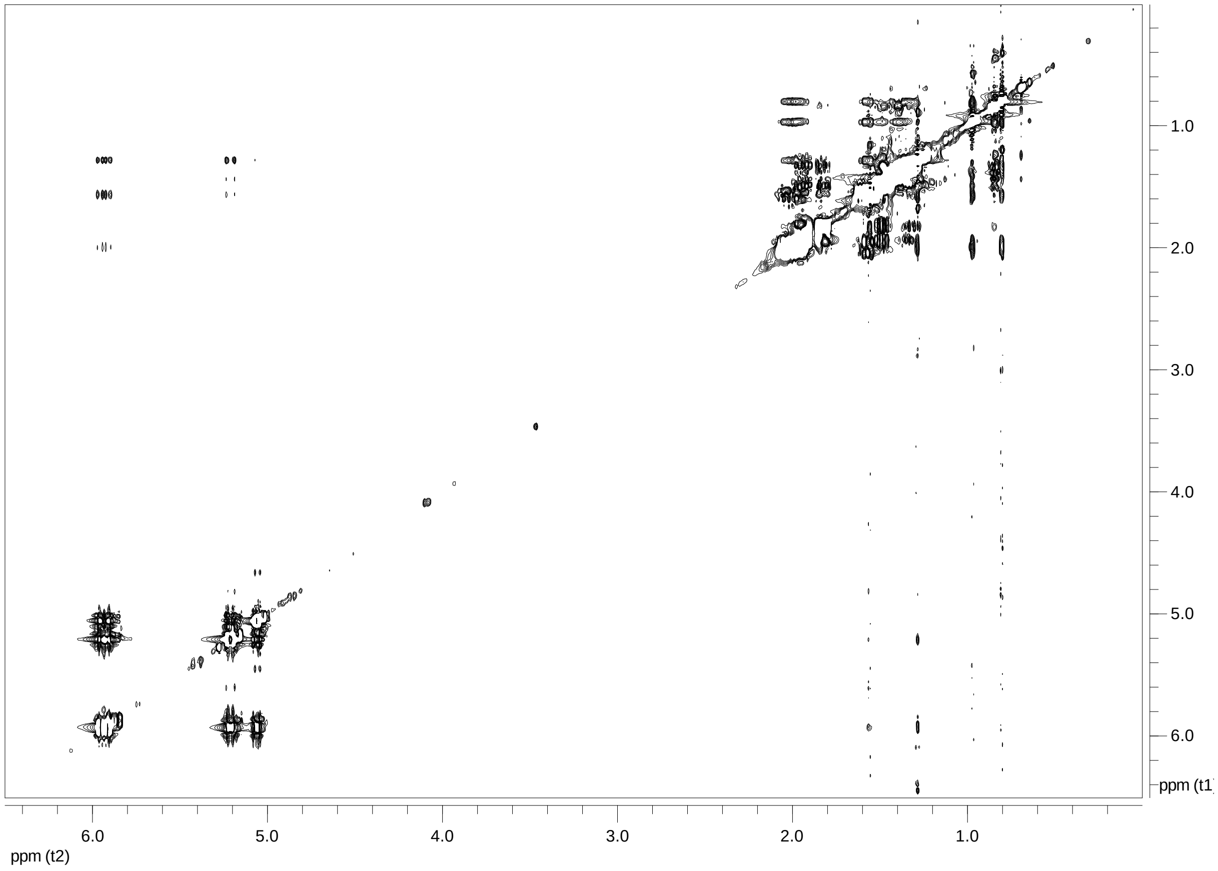

Supplementary Fig. 101. NOESY spectrum (in $CDCl_3$) of compound **3b**.

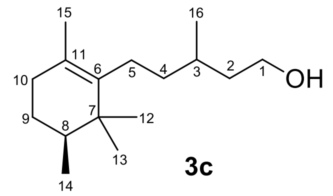

3c

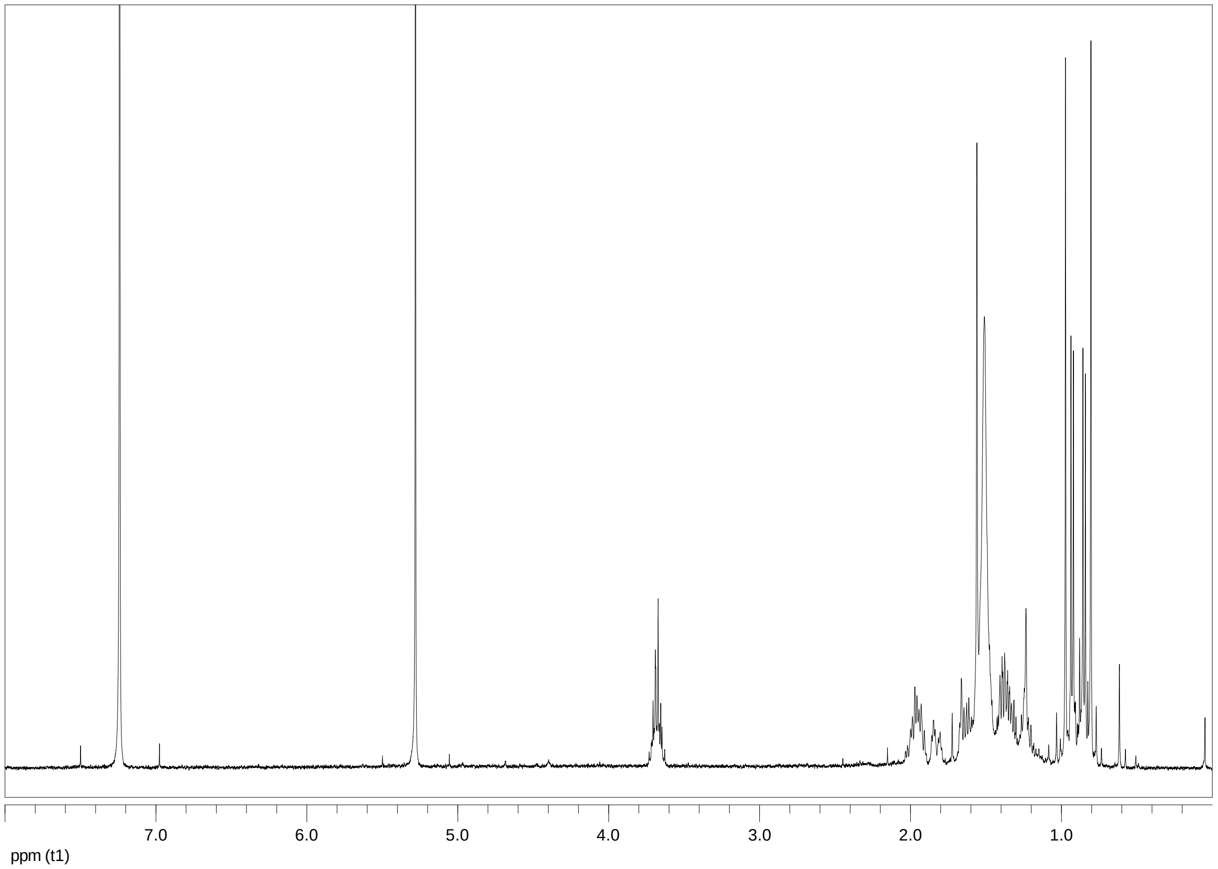

Supplementary Fig. 102. ^{1}H NMR spectrum (in $CDCl_3$) of compound **3c**.

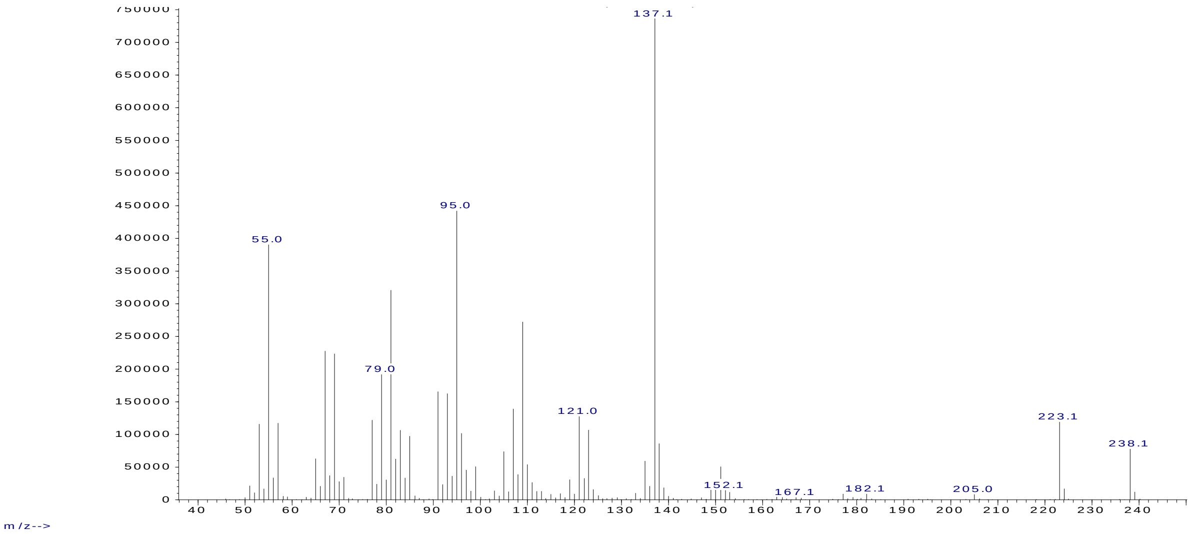

Supplementary Fig. 103. LR-EI-MS spectrum of compound **3c**.

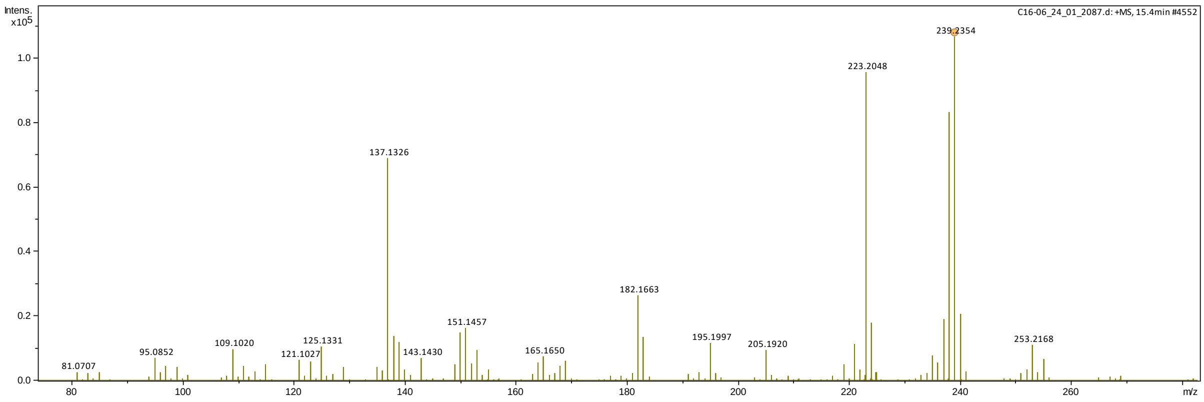

Supplementary Fig. 104. HR-APCI-MS spectrum of compound **3c**.

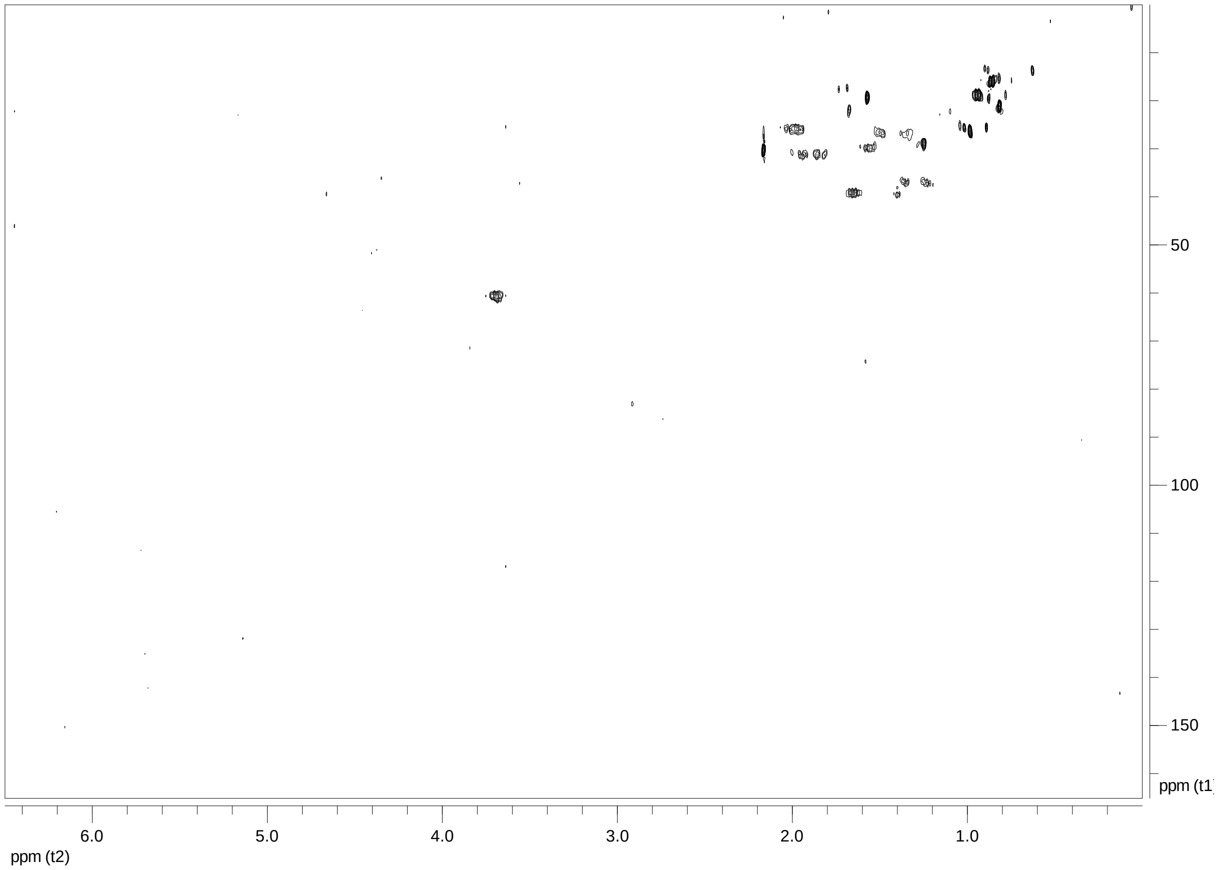

Supplementary Fig. 105. HSQC spectrum (in $CDCl_3$) of compound **3c**.

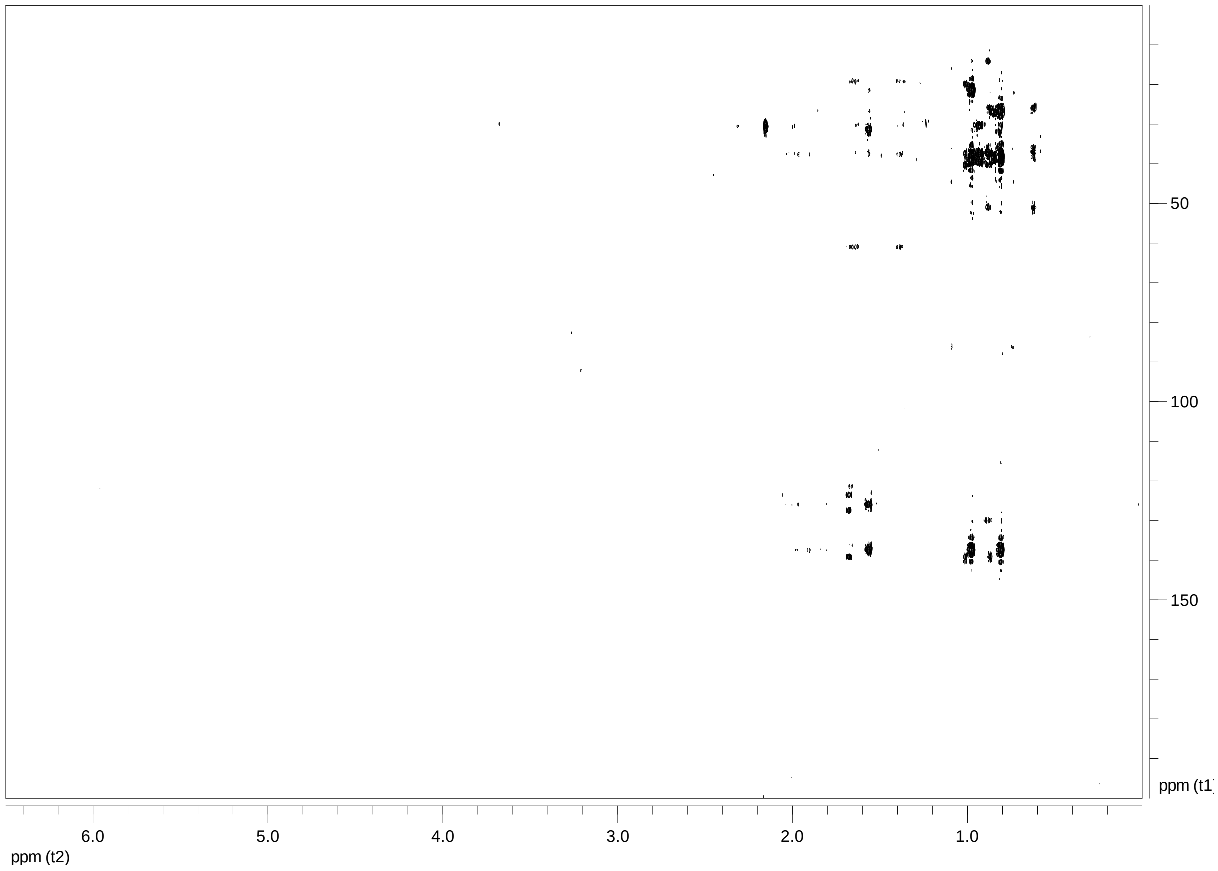

Supplementary Fig. 106. HMBC spectrum (in $CDCl_3$) of compound **3c**.

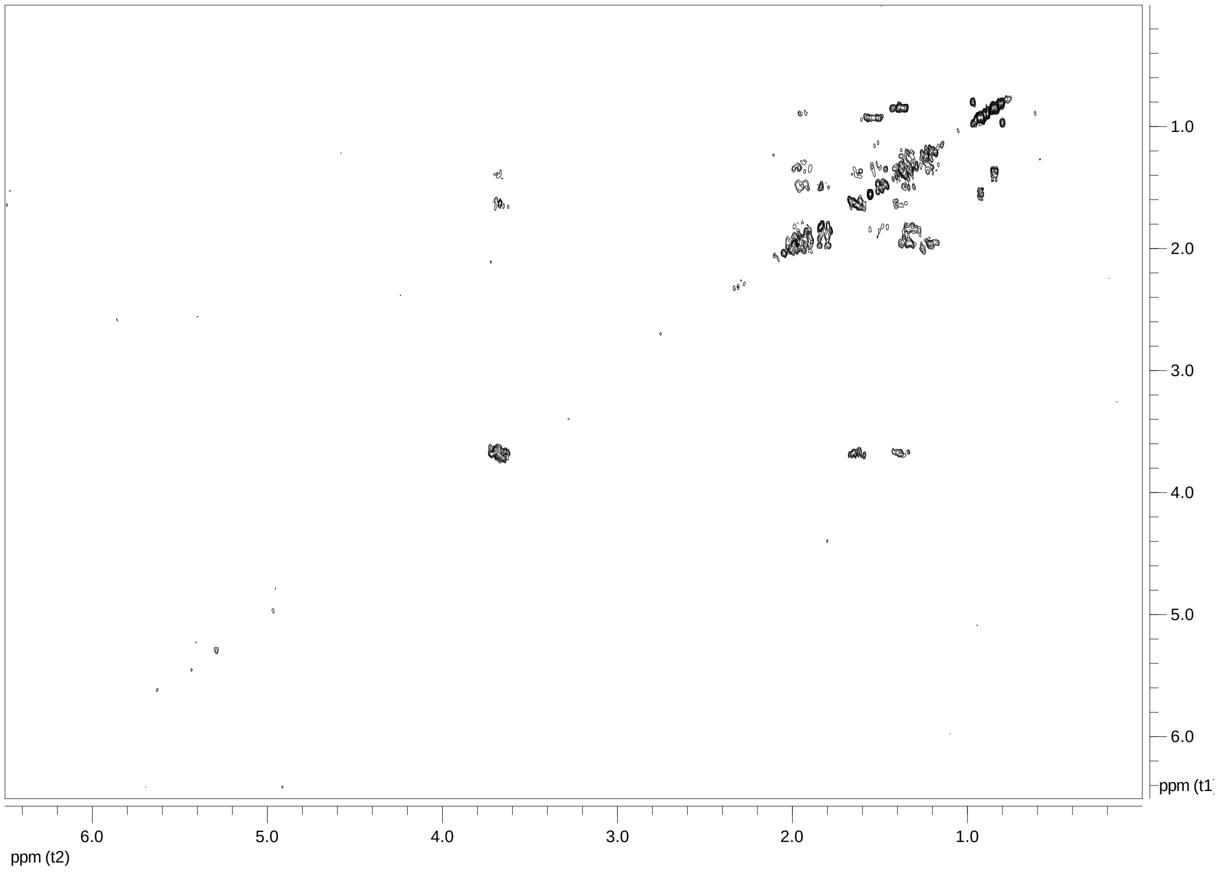

Supplementary Fig. 107. COSY spectrum (in $CDCl_3$) of compound **3c**.

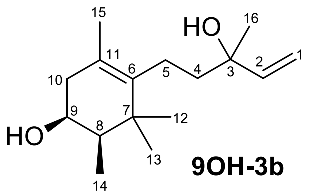

9OH-3b

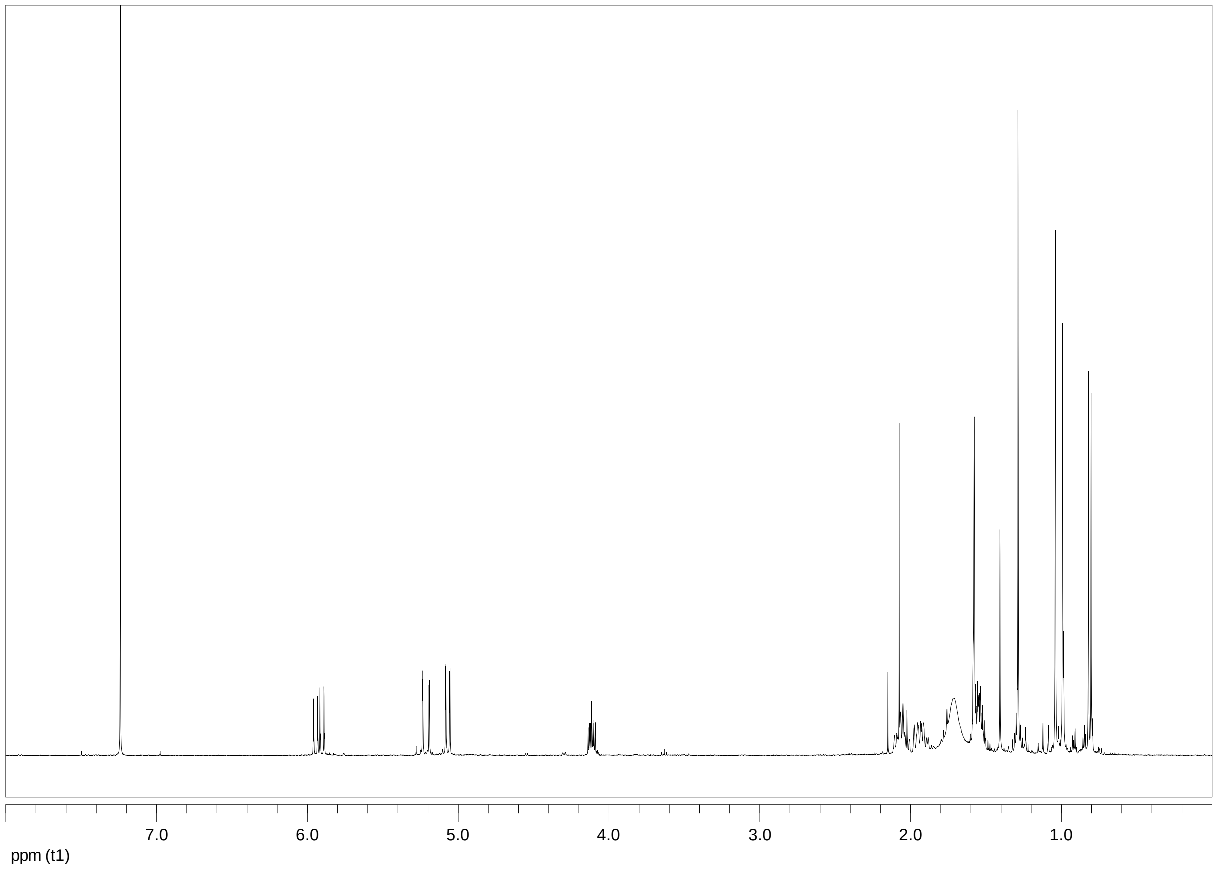

Supplementary Fig. 108. ^{1}H NMR spectrum (in $CDCl_3$) of compound **9OH-3b**.

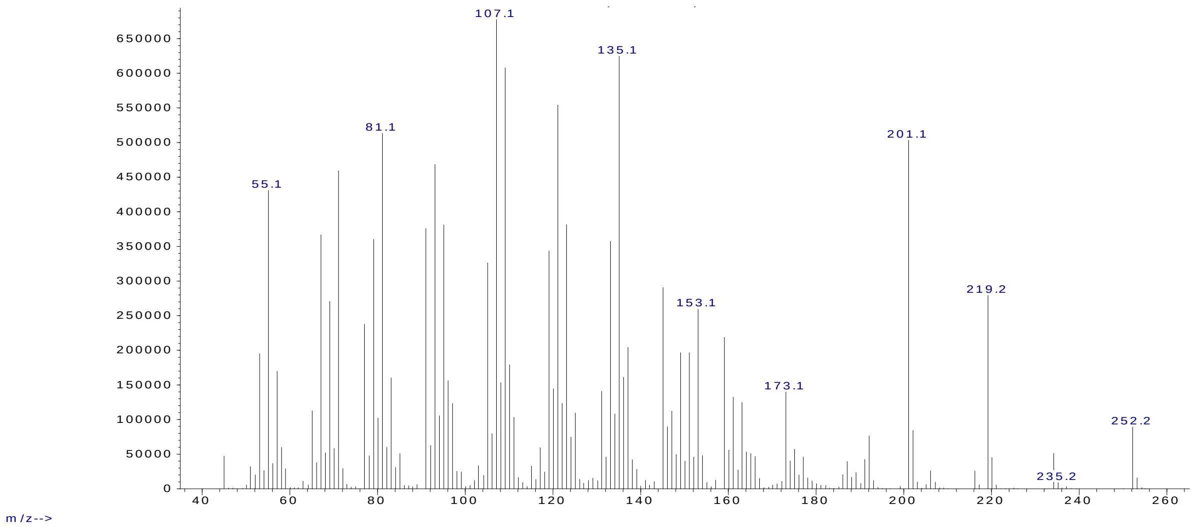

Supplementary Fig. 109. LR-EI-MS spectrum of compound **9OH-3b**.

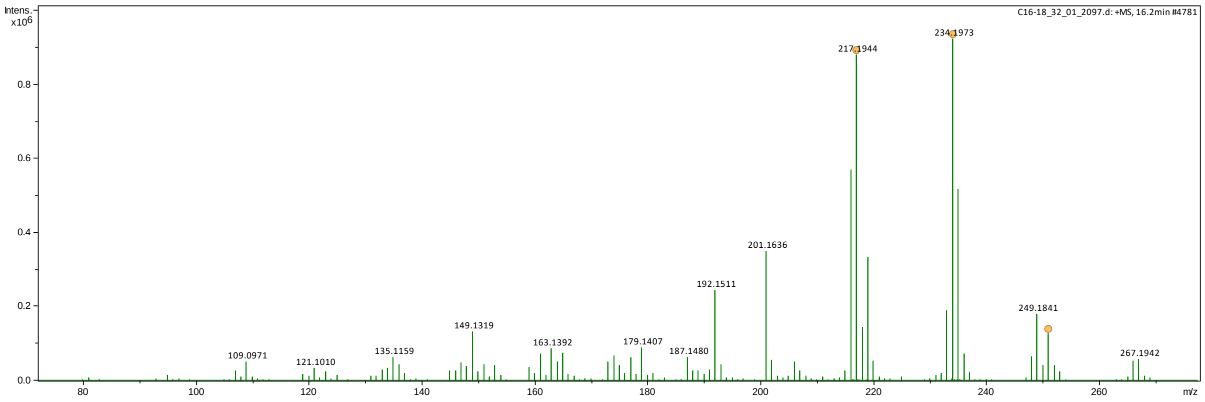

Supplementary Fig. 110. HR-APCI-MS spectrum of compound **9OH-3b**.

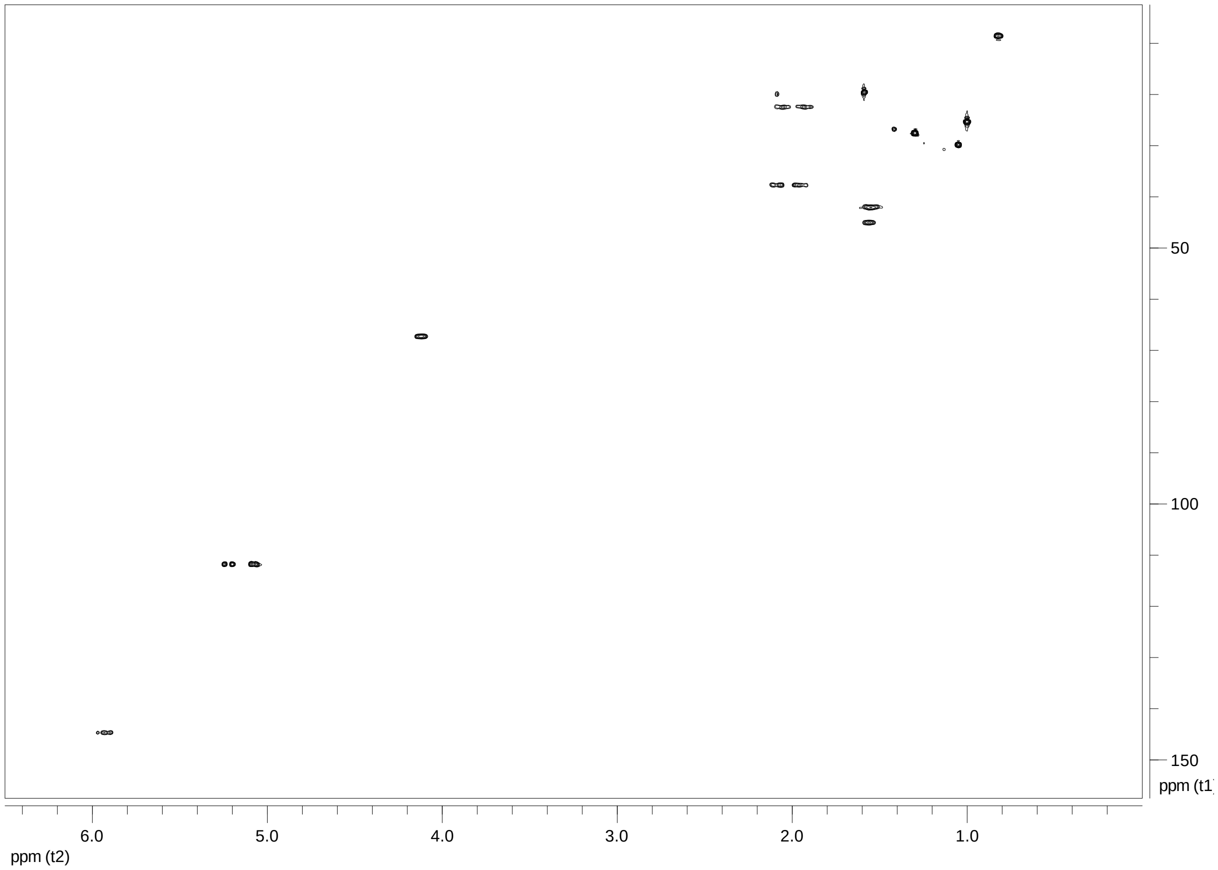

Supplementary Fig. 111. HSQC spectrum (in $CDCl_3$) of compound **9OH-3b**.

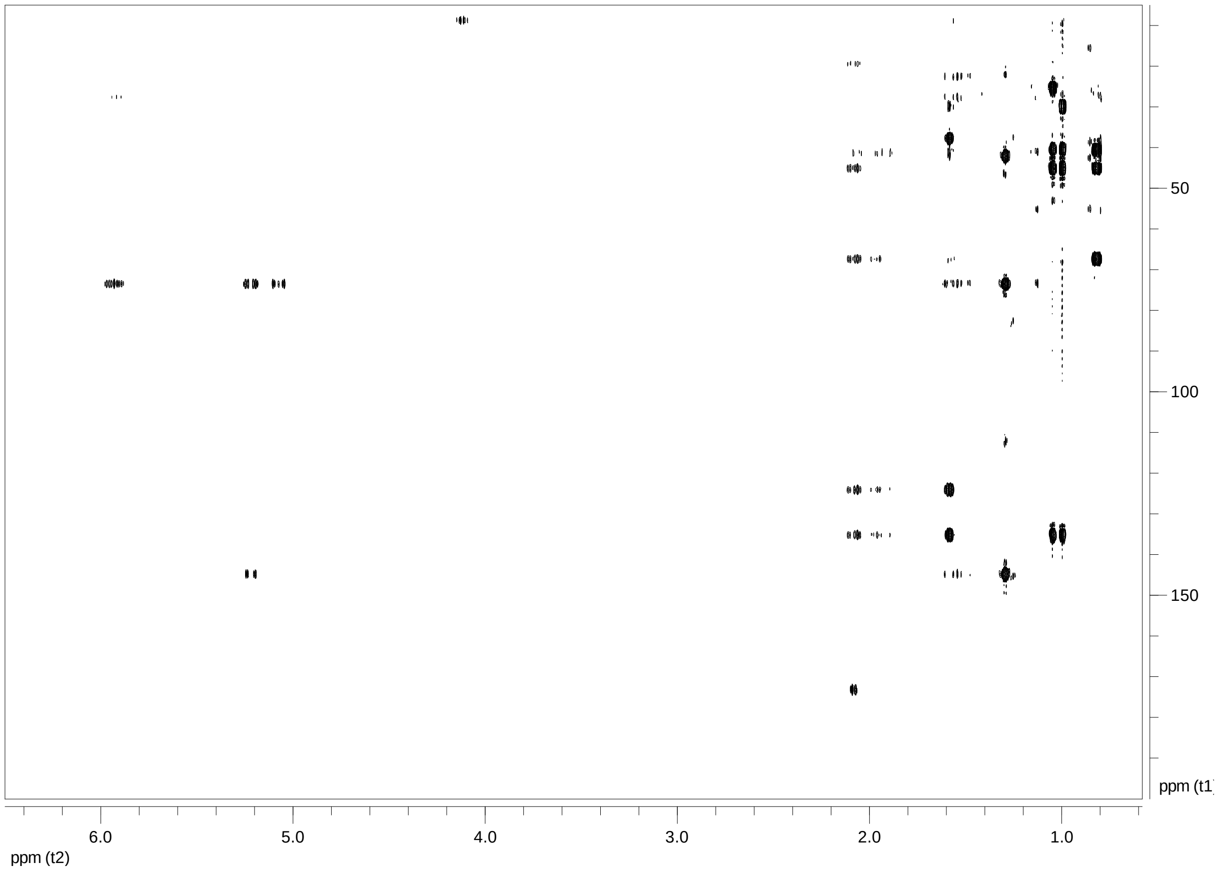

Supplementary Fig. 112. HMBC spectrum (in $CDCl_3$) of compound **9OH-3b**.

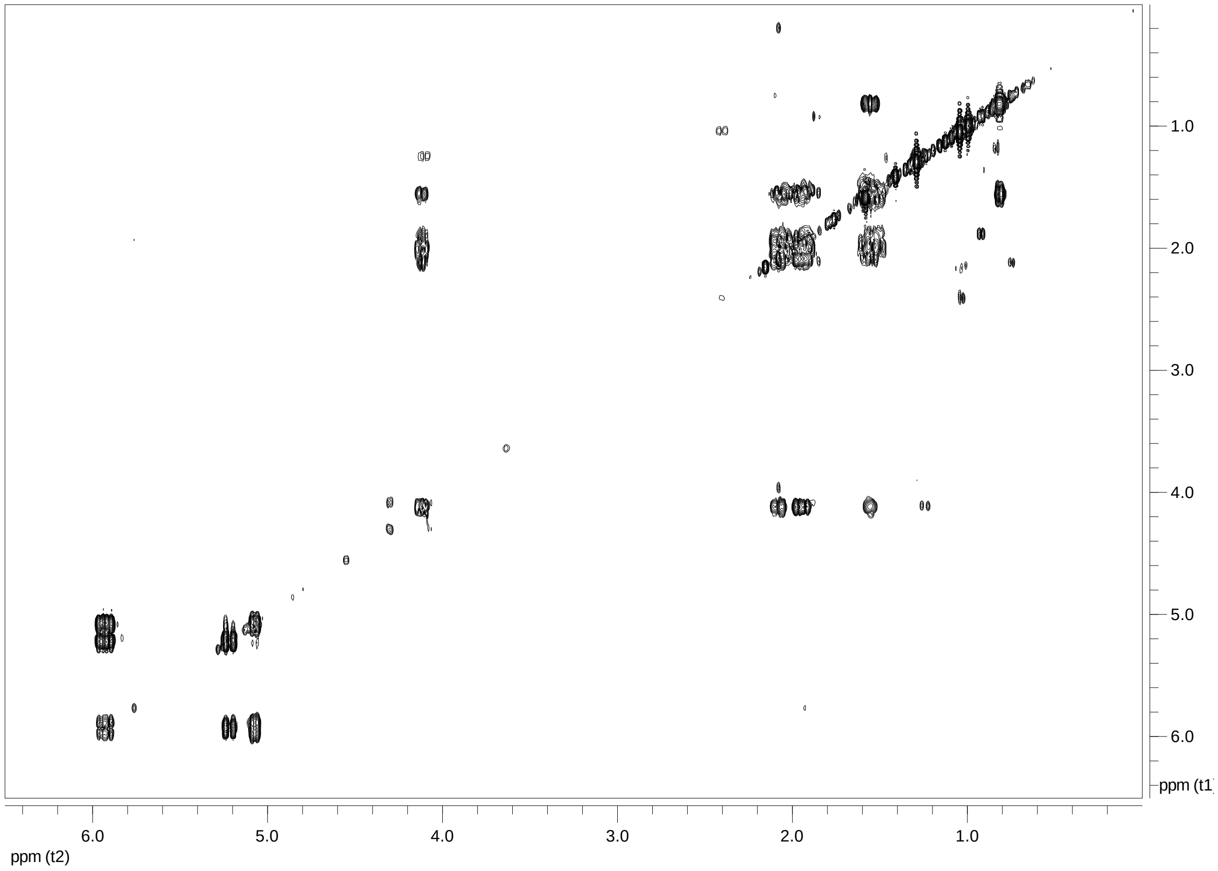

Supplementary Fig. 113. COSY spectrum (in $CDCl_3$) of compound **9OH-3b**.

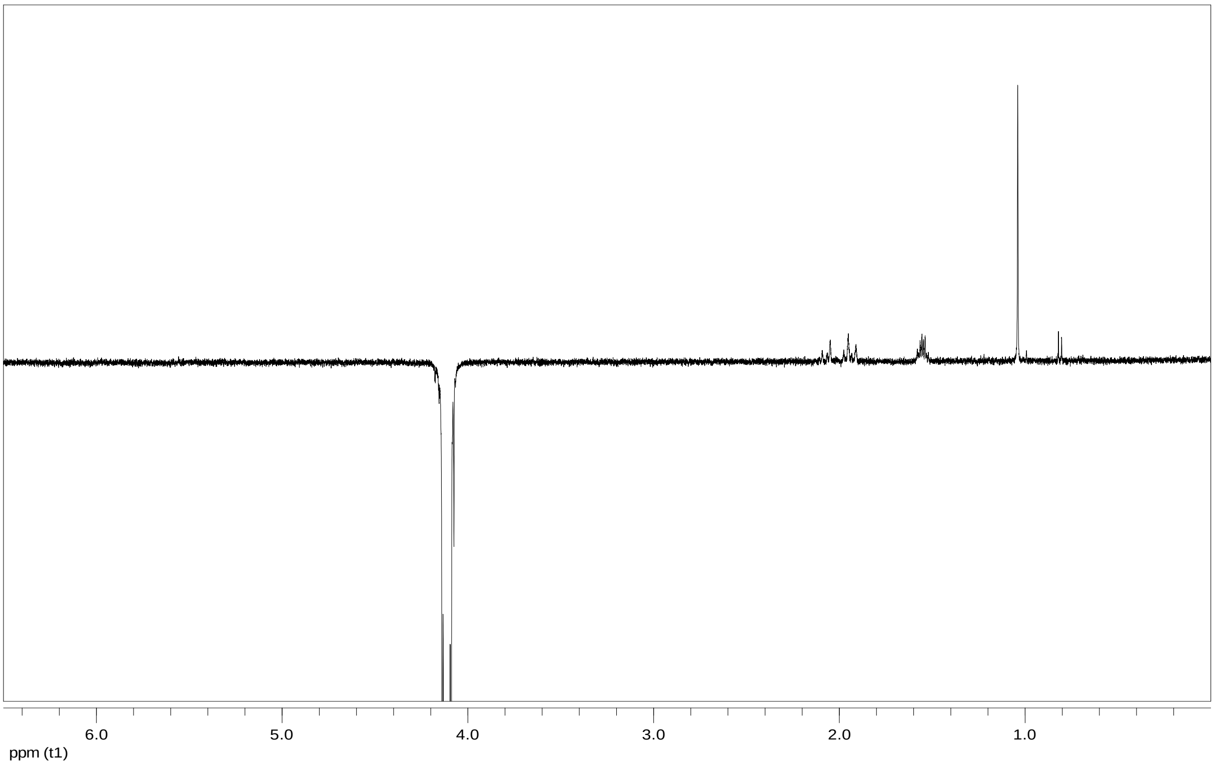

Supplementary Fig. 114. 1D NOE difference spectrum (in $CDCl_3$) of compound **9OH-3b** upon irradiation of H-9.

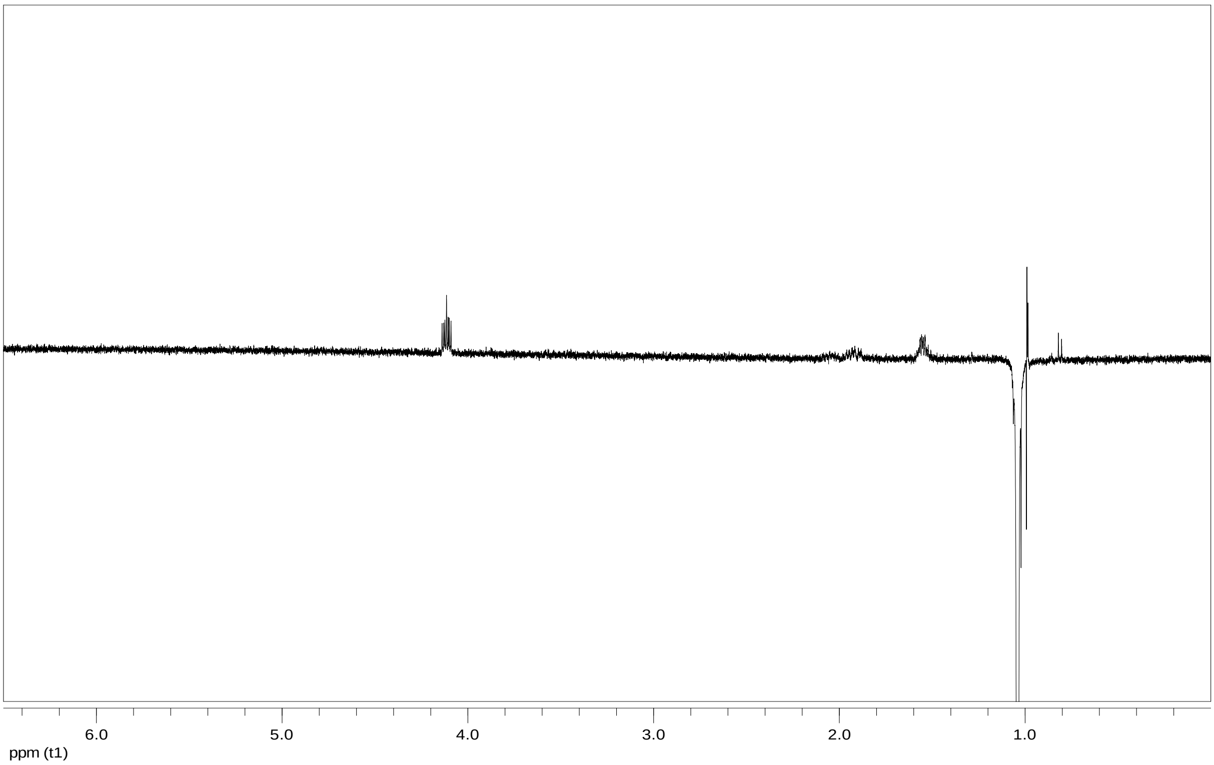

Supplementary Fig. 115. 1D NOE difference spectrum (in $CDCl_3$) of compound **9OH-3b** upon irradiation of H_3-12.

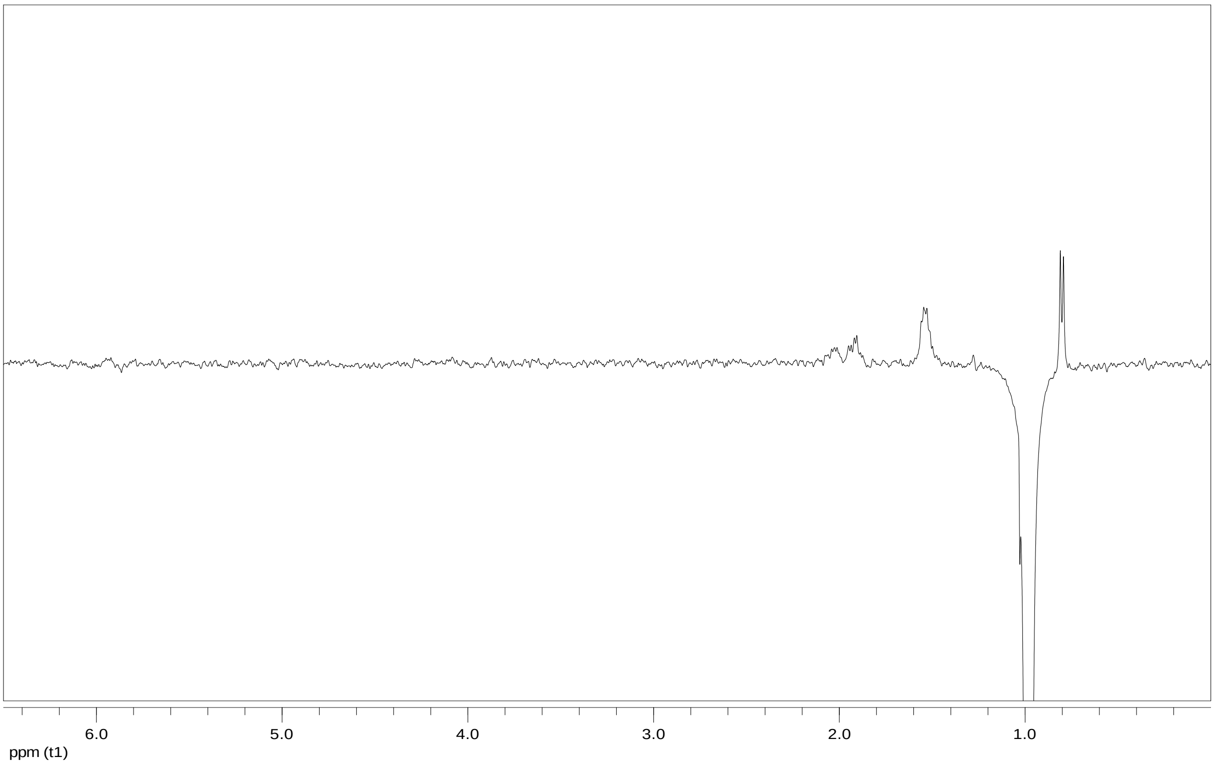

Supplementary Fig. 116. 1D NOE difference spectrum (in $CDCl_3$) of compound **9OH-3b** upon irradiation of H_3-13.

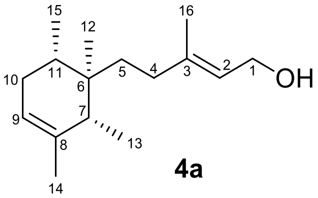

4a

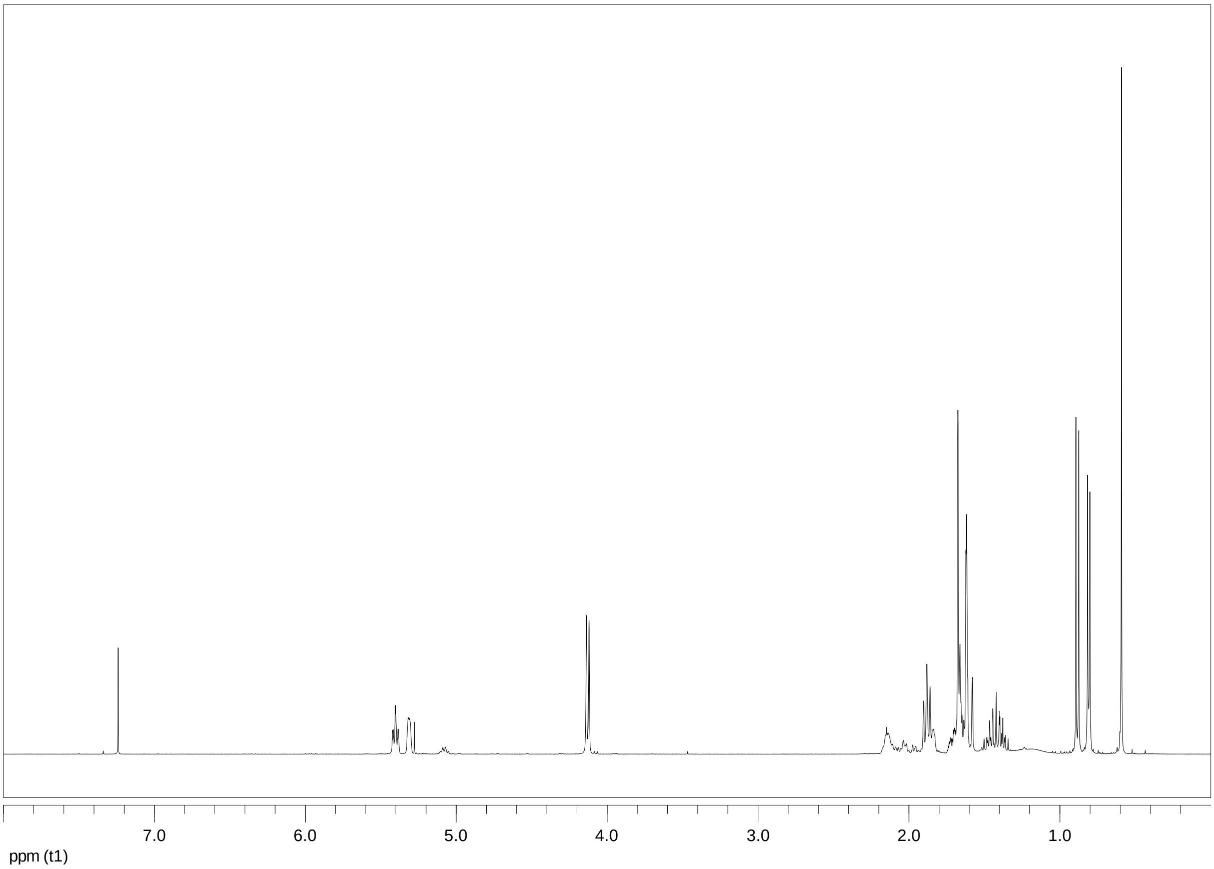

Supplementary Fig. 117. ^{1}H NMR spectrum (in $CDCl_3$) of compound **4a**.

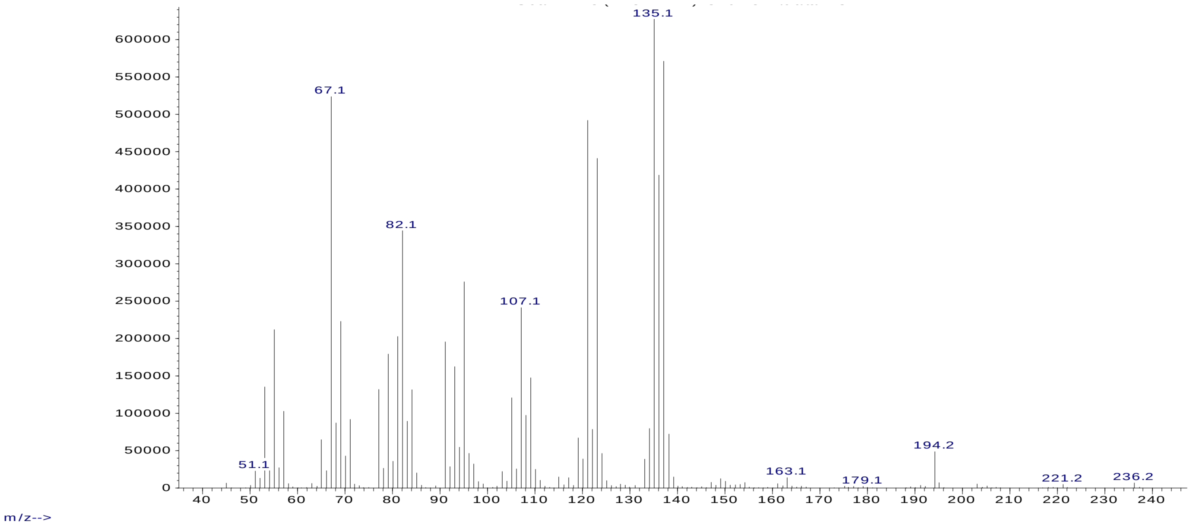

Supplementary Fig. 118. LR-EI-MS spectrum of compound **4a**.

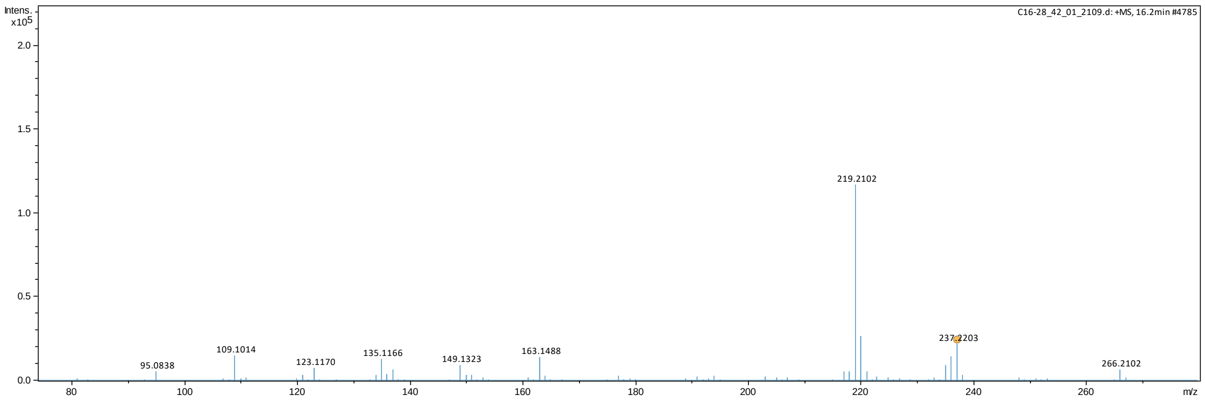

Supplementary Fig. 119. HR-APCI-MS spectrum of compound **4a**.

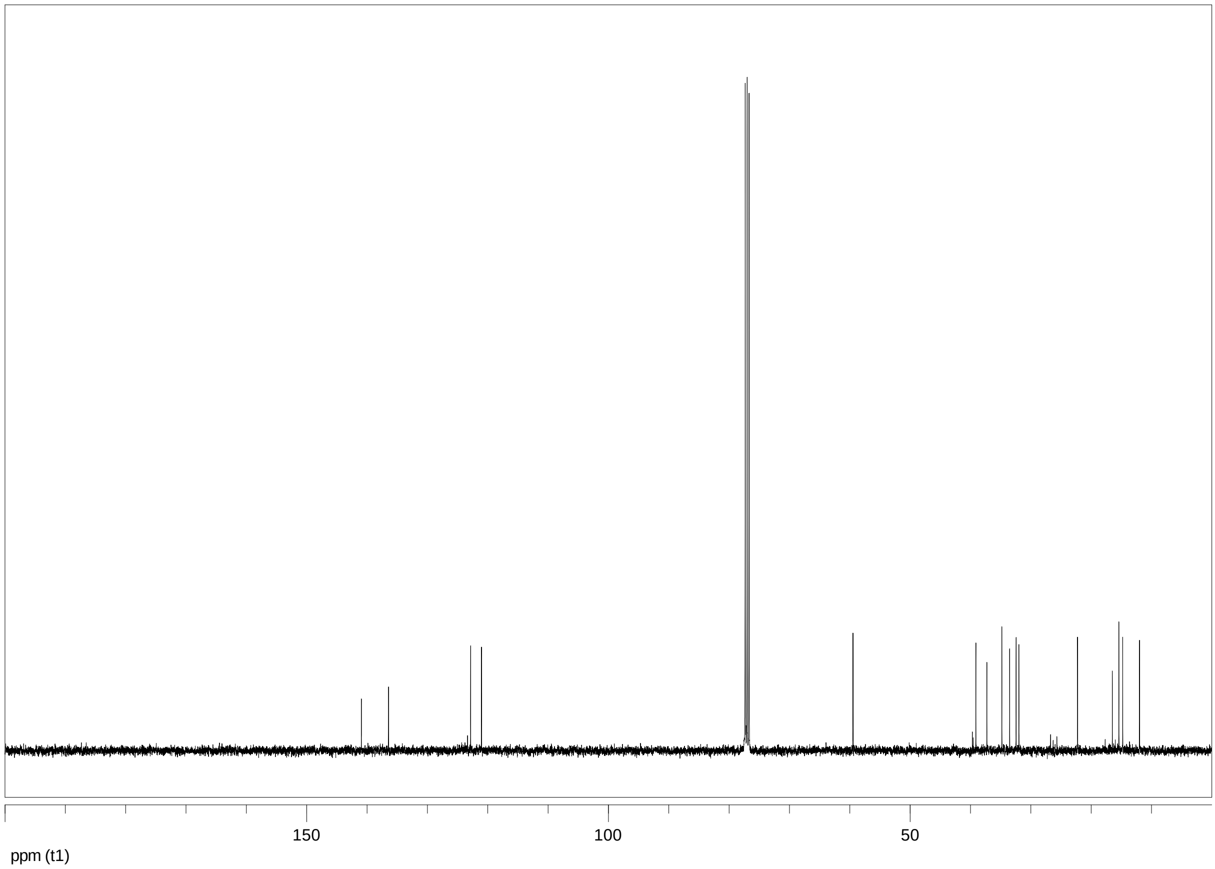

Supplementary Fig. 120. ^{13}C NMR spectrum (in $CDCl_3$) of compound **4a**.

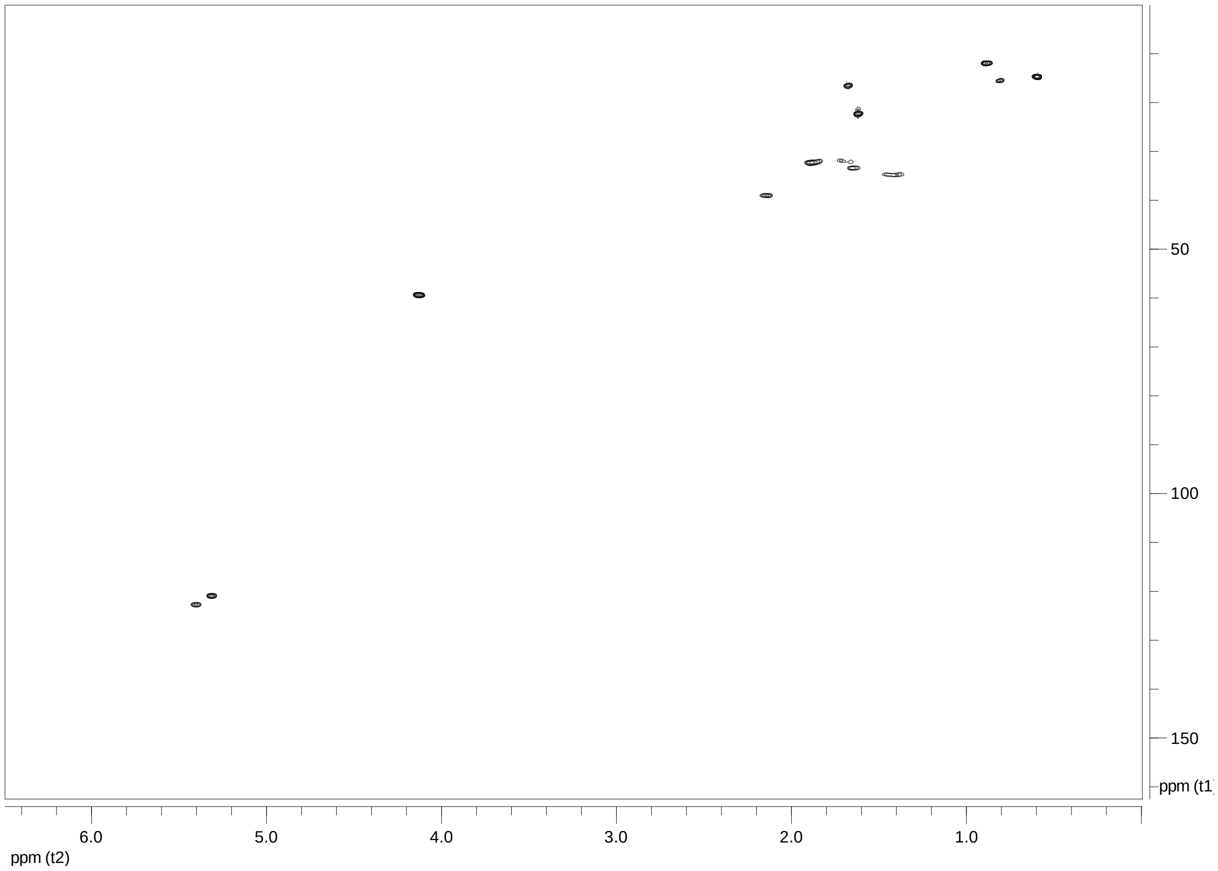

Supplementary Fig. 121. HSQC spectrum (in $CDCl_3$) of compound **4a**.

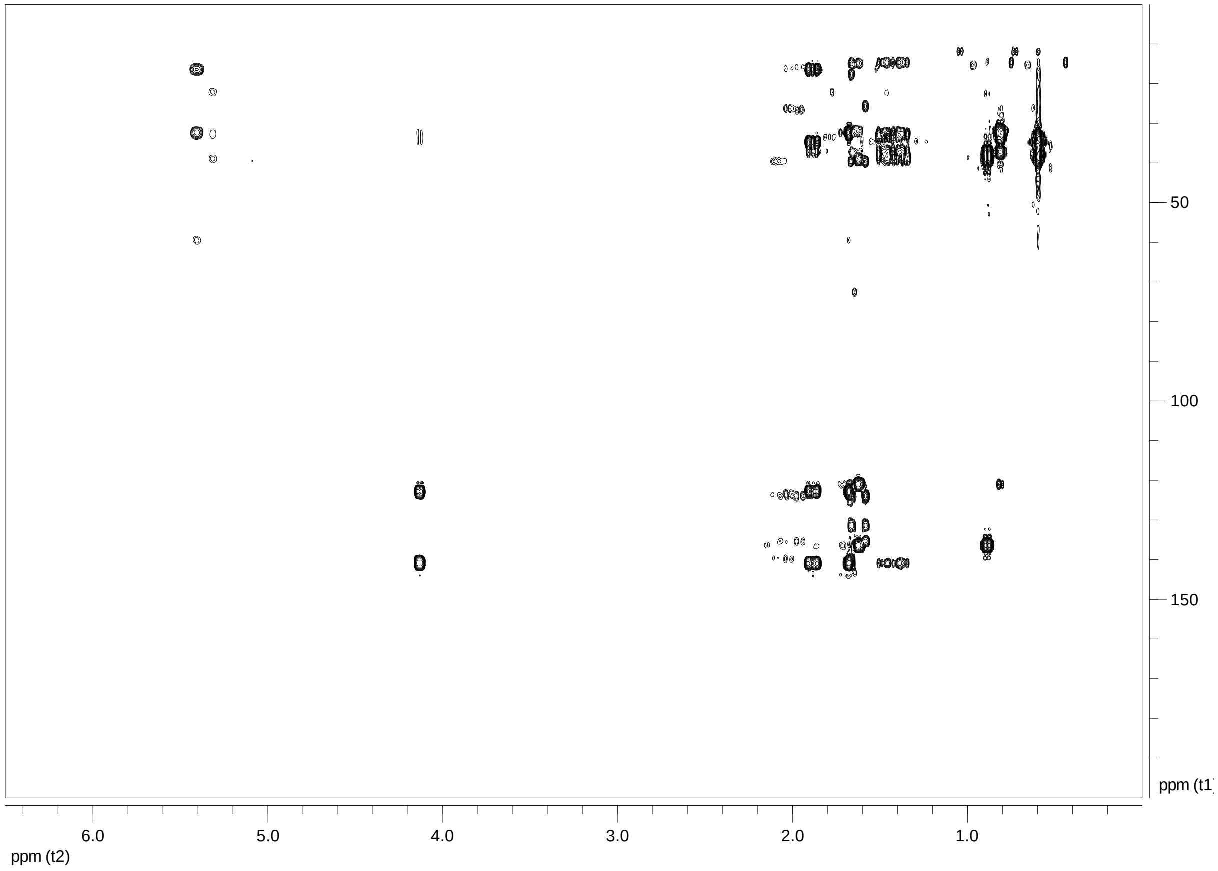

Supplementary Fig. 122. HMBC spectrum (in $CDCl_3$) of compound **4a**.

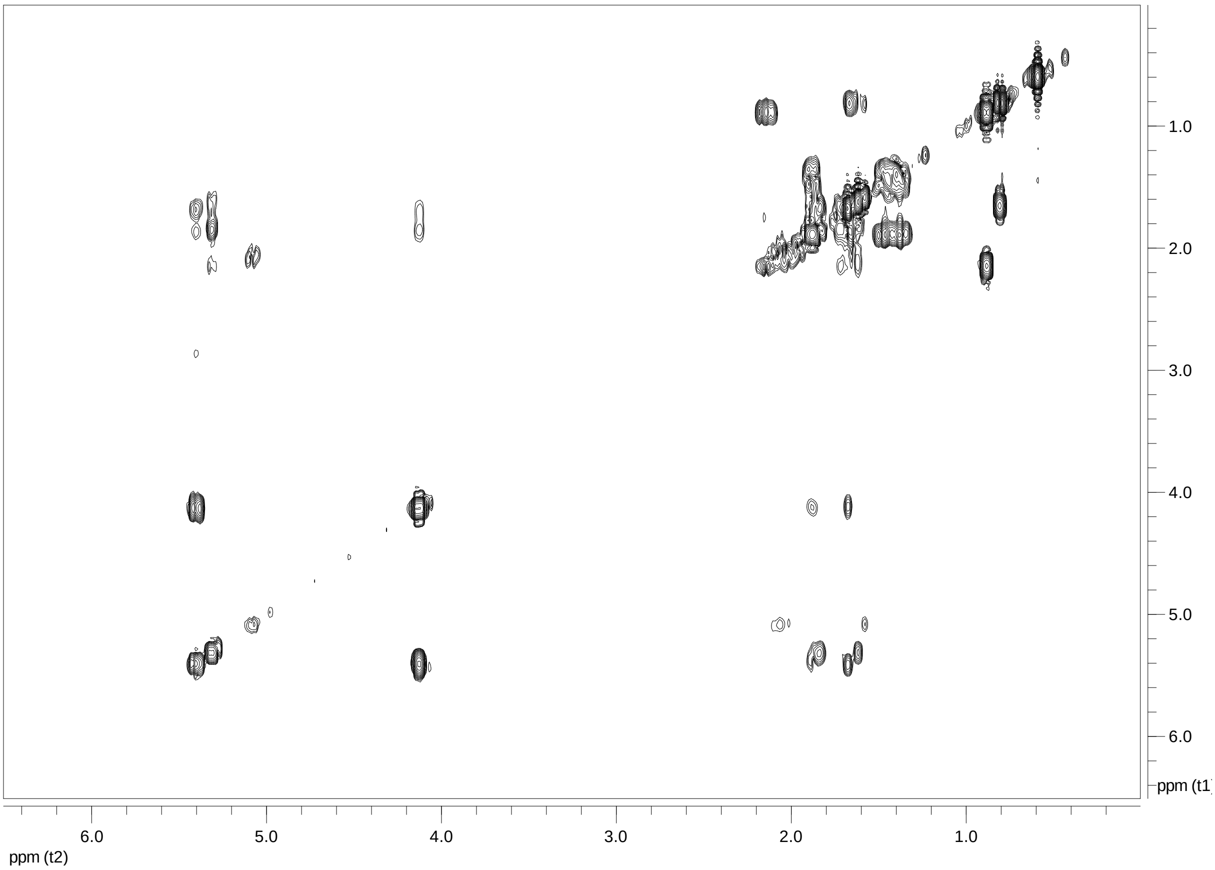

Supplementary Fig. 123. COSY spectrum (in $CDCl_3$) of compound **4a**.

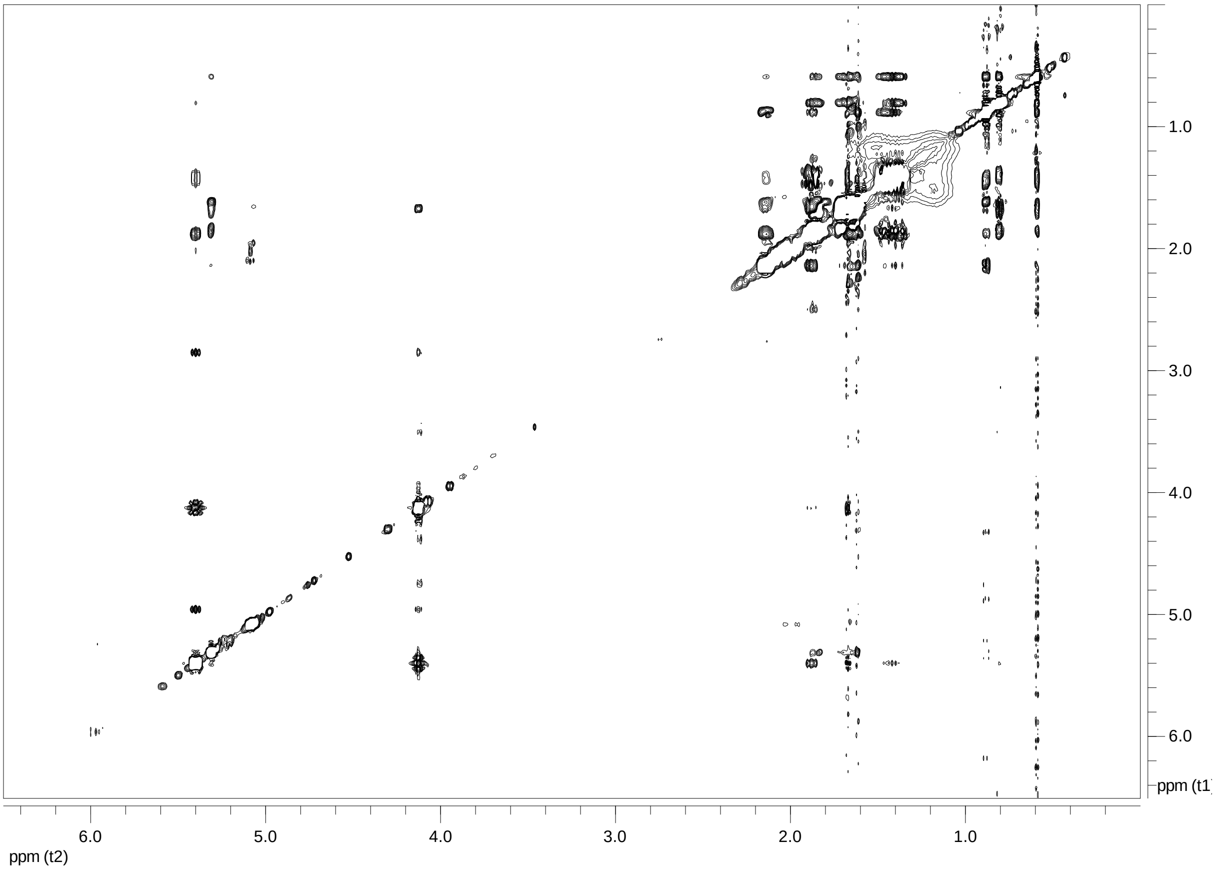

Supplementary Fig. 124. NOESY spectrum (in $CDCl_3$) of compound **4a**.

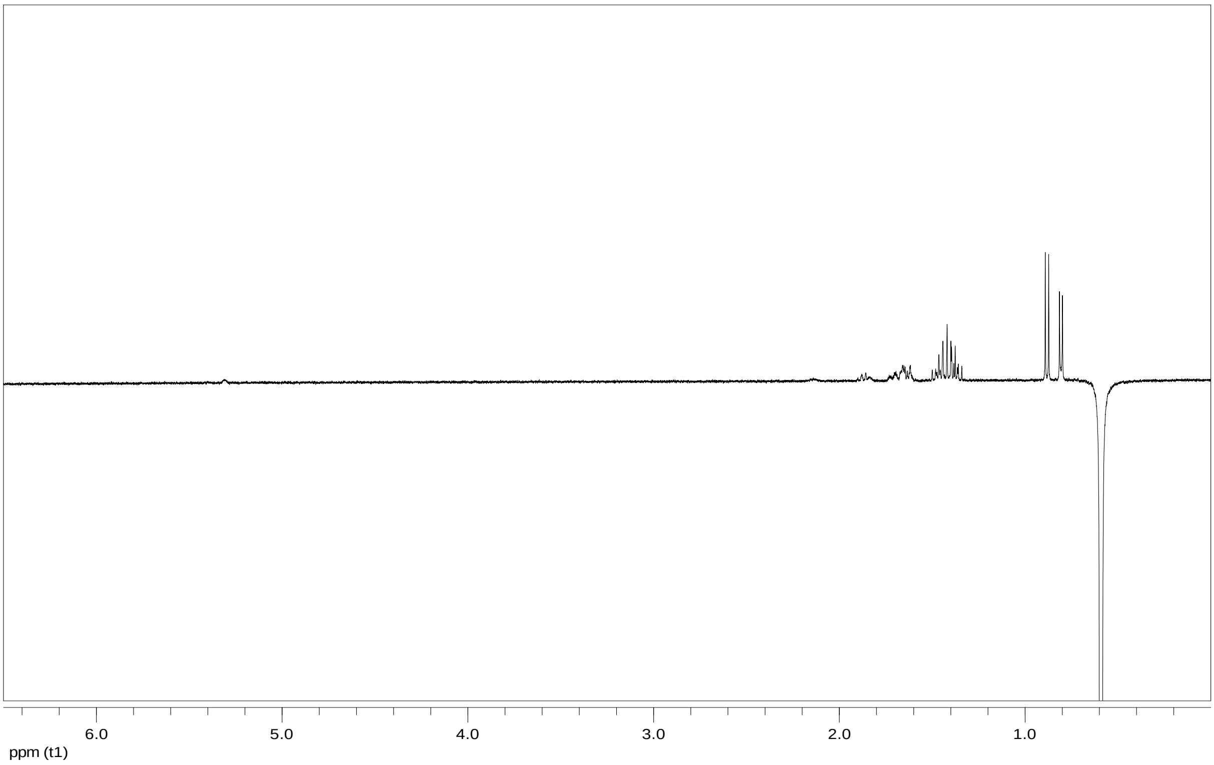

Supplementary Fig. 125. 1D NOE difference spectrum (in $CDCl_3$) of compound **4a** upon irradiation of H_3-12.

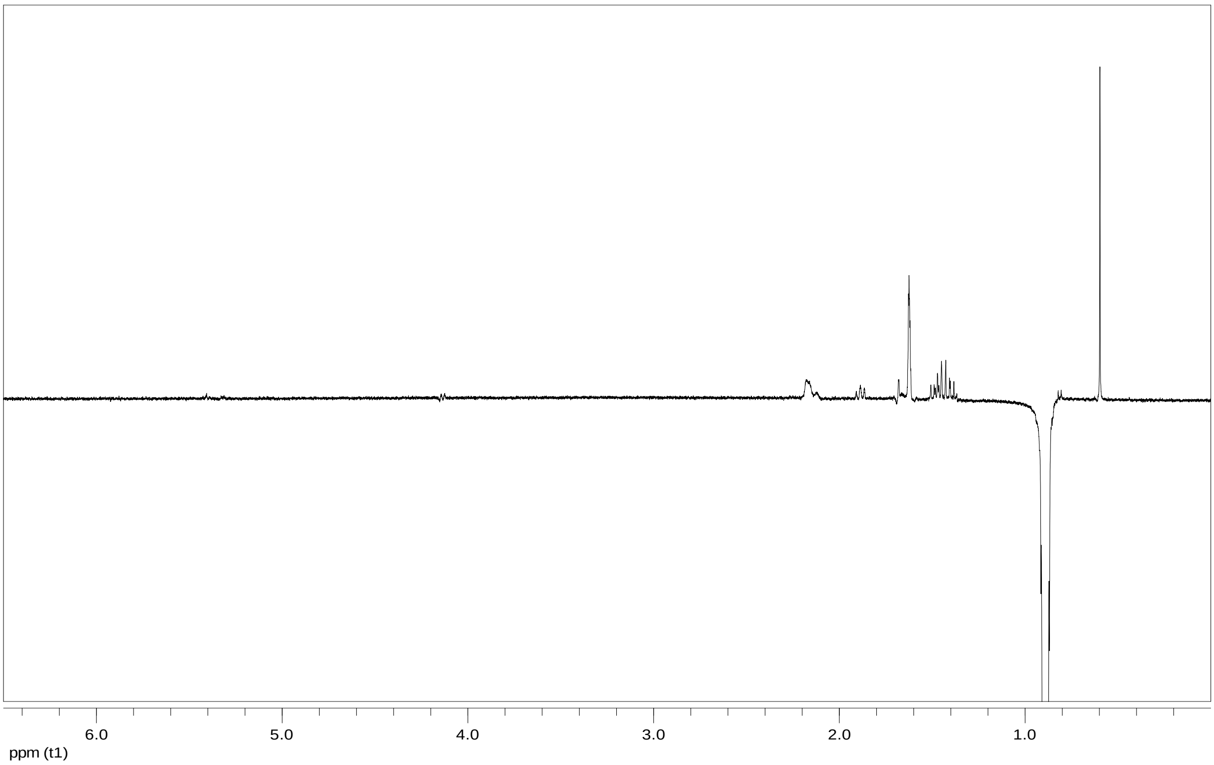

Supplementary Fig. 126. 1D NOE difference spectrum (in $CDCl_3$) of compound **4a** upon irradiation of H_3-13.

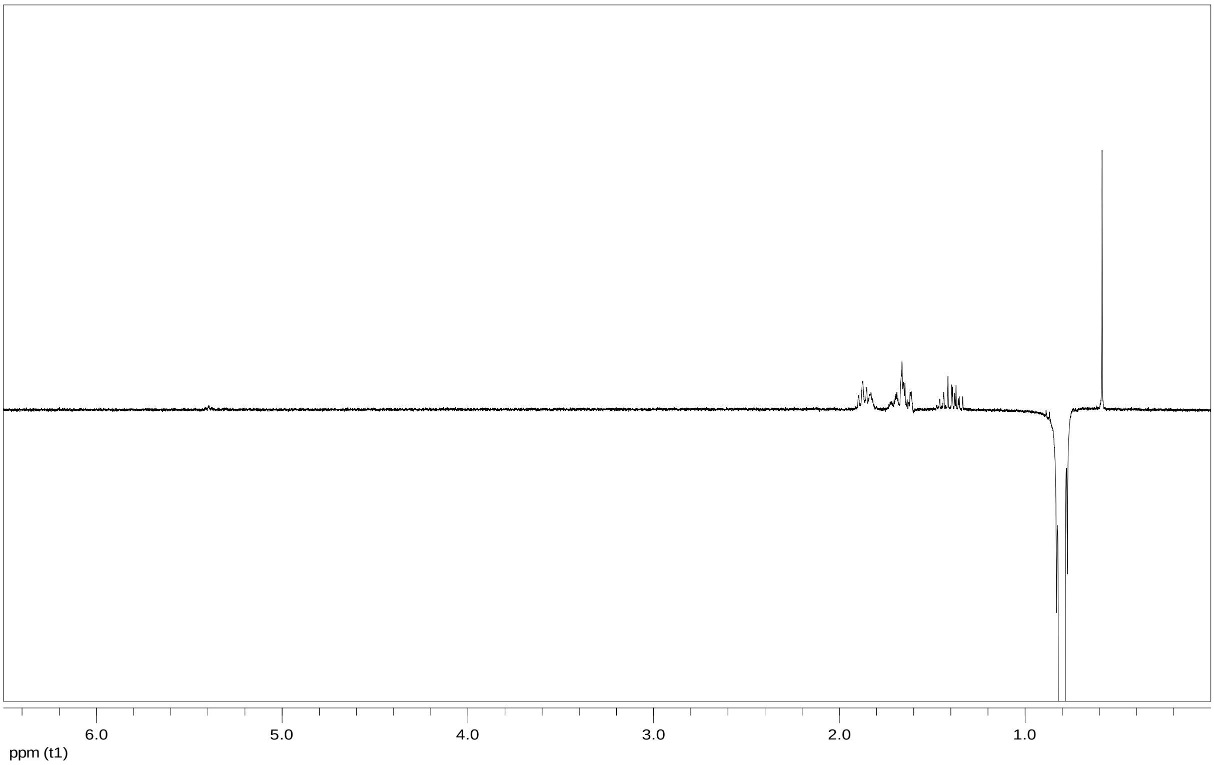

Supplementary Fig. 127. 1D NOE difference spectrum (in $CDCl_3$) of compound **4a** upon irradiation of H_3-15.

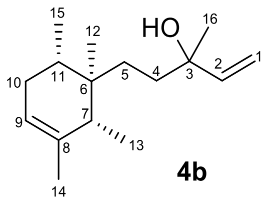

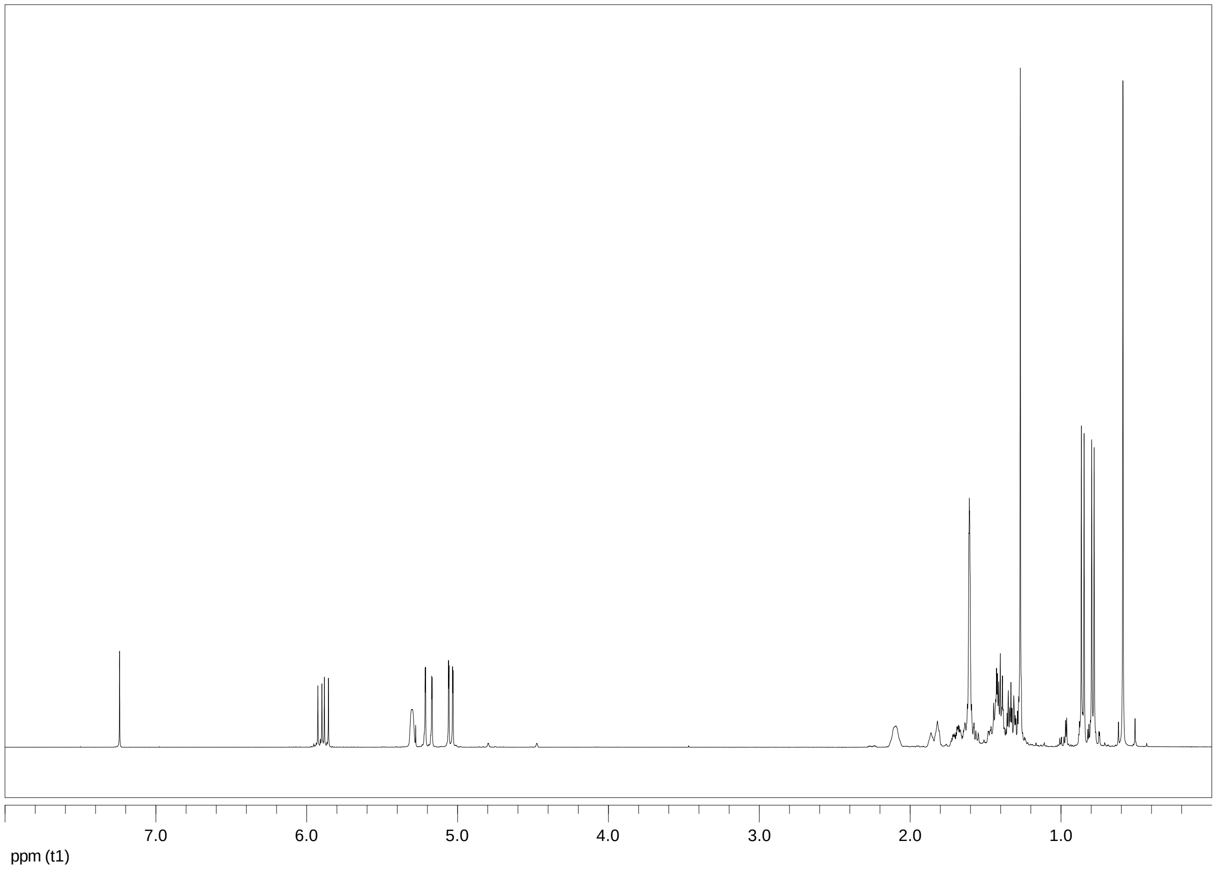

Supplementary Fig. 128. ^{1}H NMR spectrum (in $CDCl_3$) of compound **4b**.

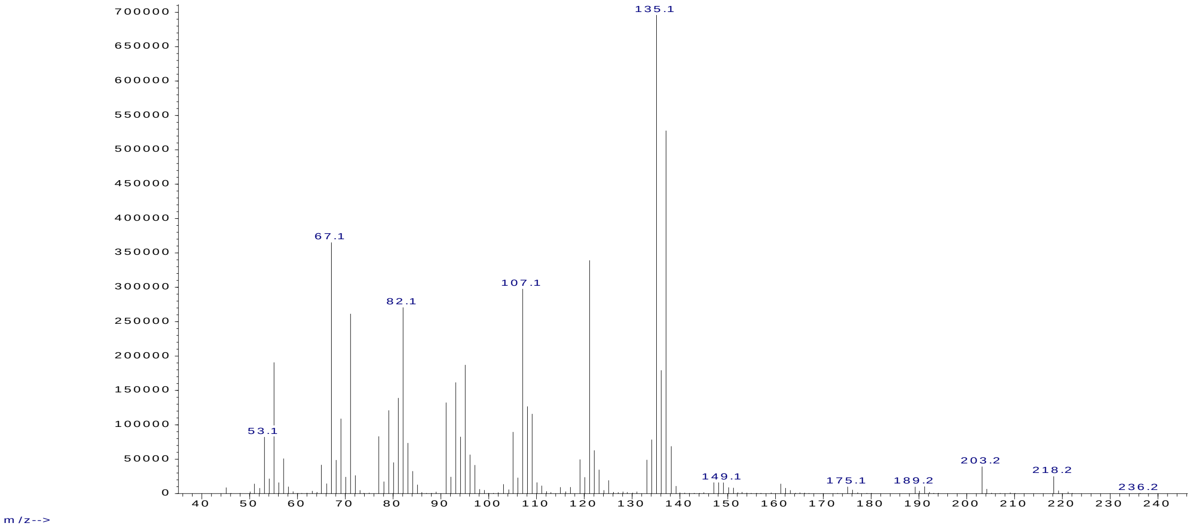

Supplementary Fig. 129. LR-EI-MS spectrum of compound **4b**.

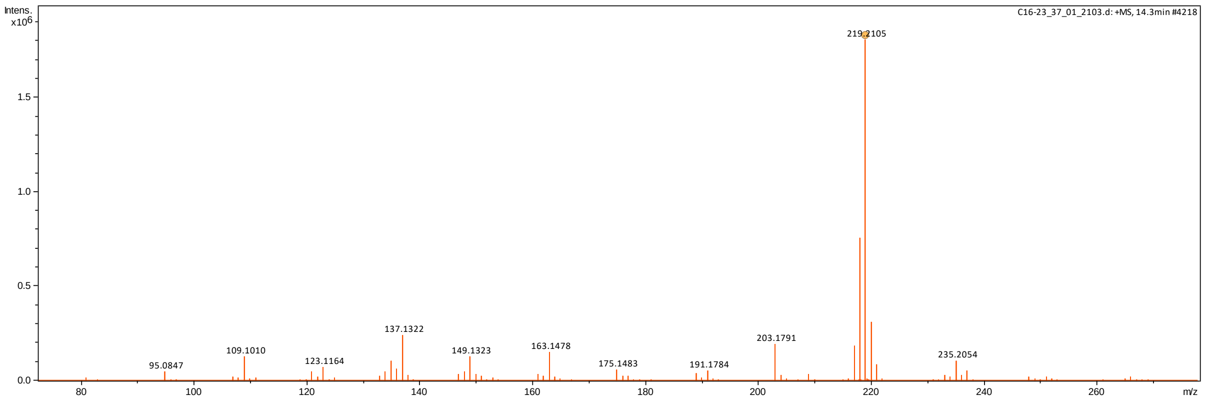

Supplementary Fig. 130. HR-APCI-MS spectrum of compound **4b**.

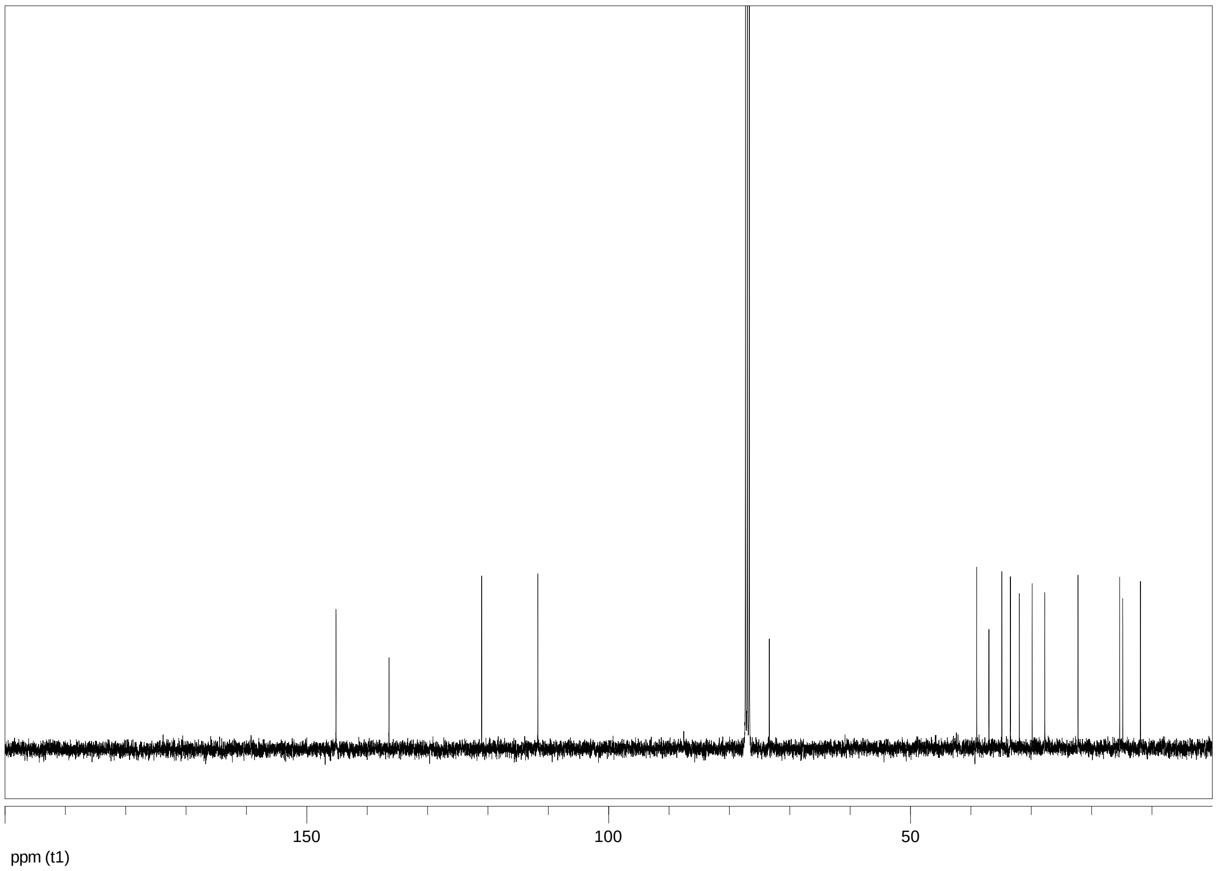

Supplementary Fig. 131. ^{13}C NMR spectrum (in $CDCl_3$) of compound **4b**.

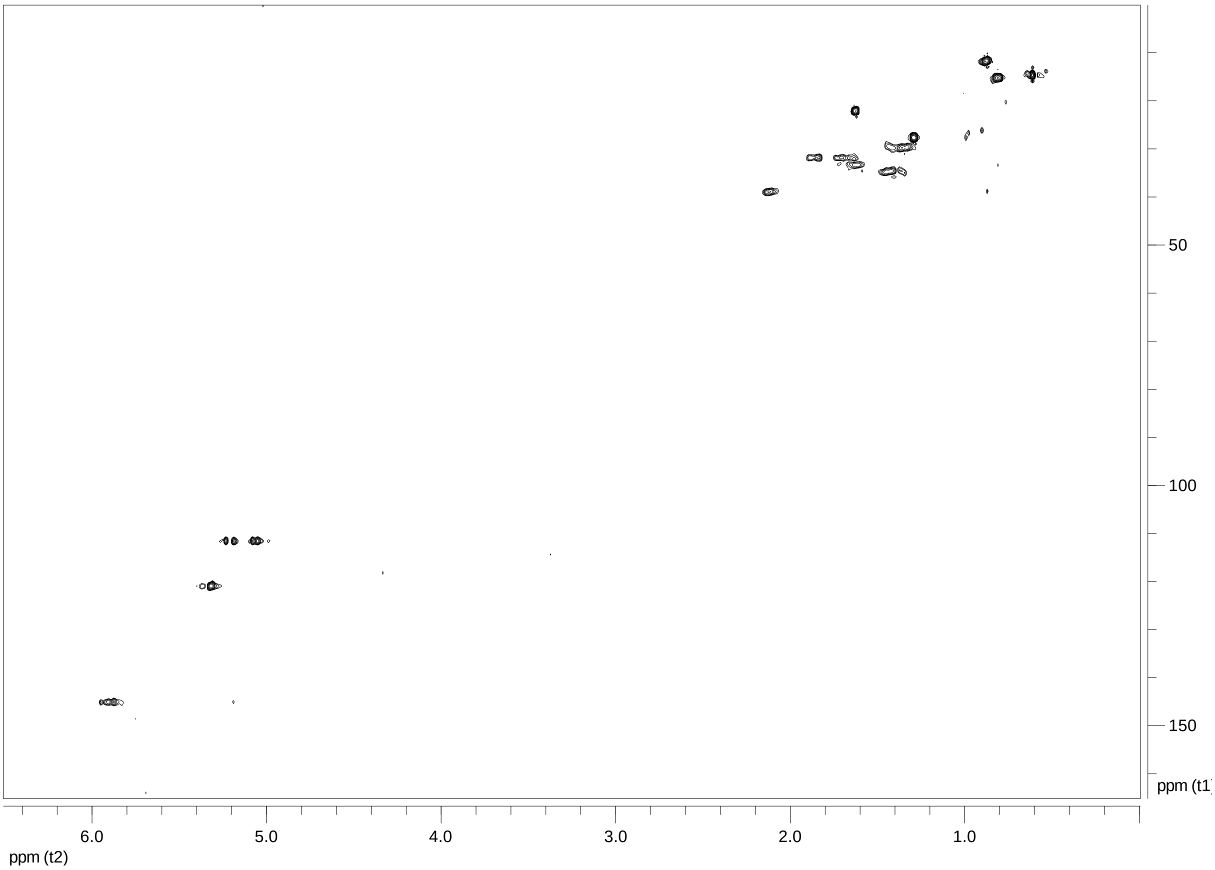

Supplementary Fig. 132. HSQC spectrum (in $CDCl_3$) of compound **4b**.

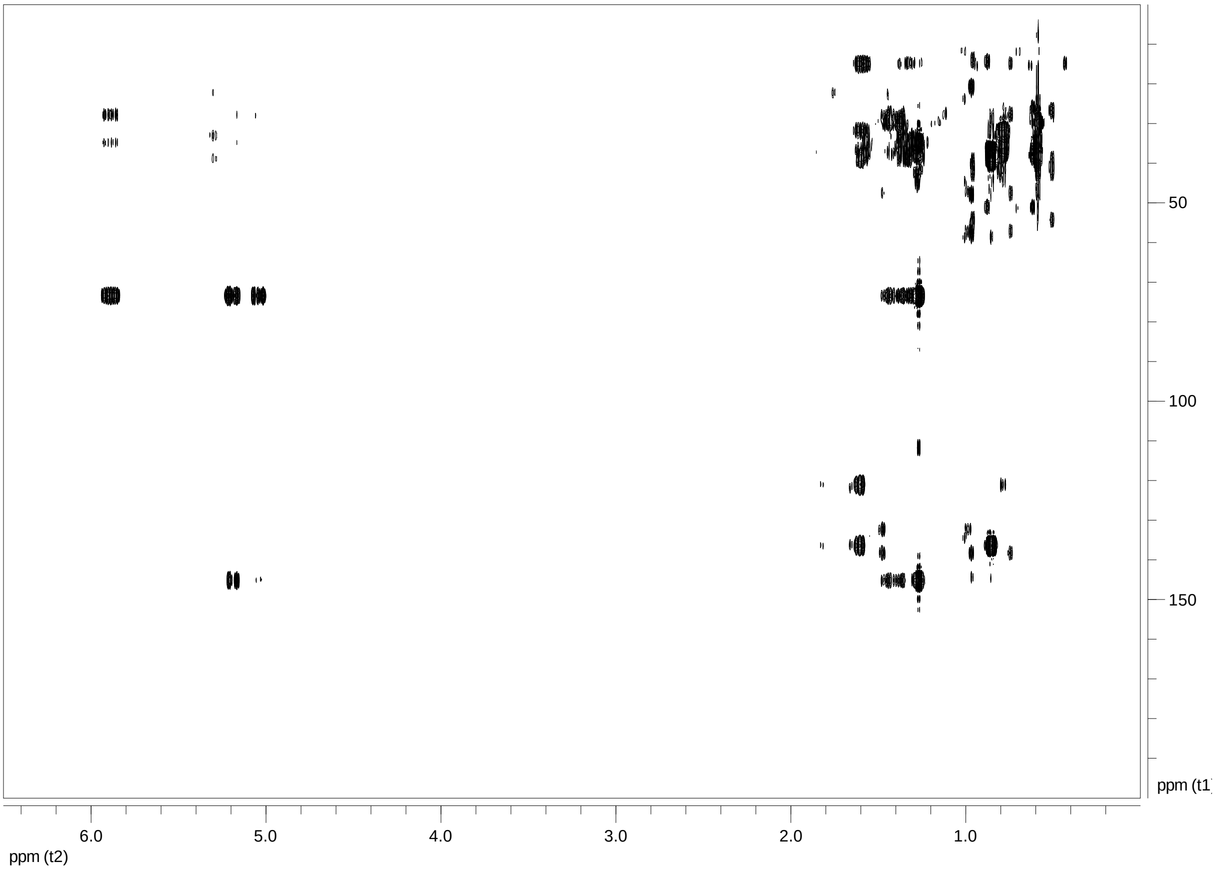

Supplementary Fig. 133. HMBC spectrum (in $CDCl_3$) of compound **4b**.

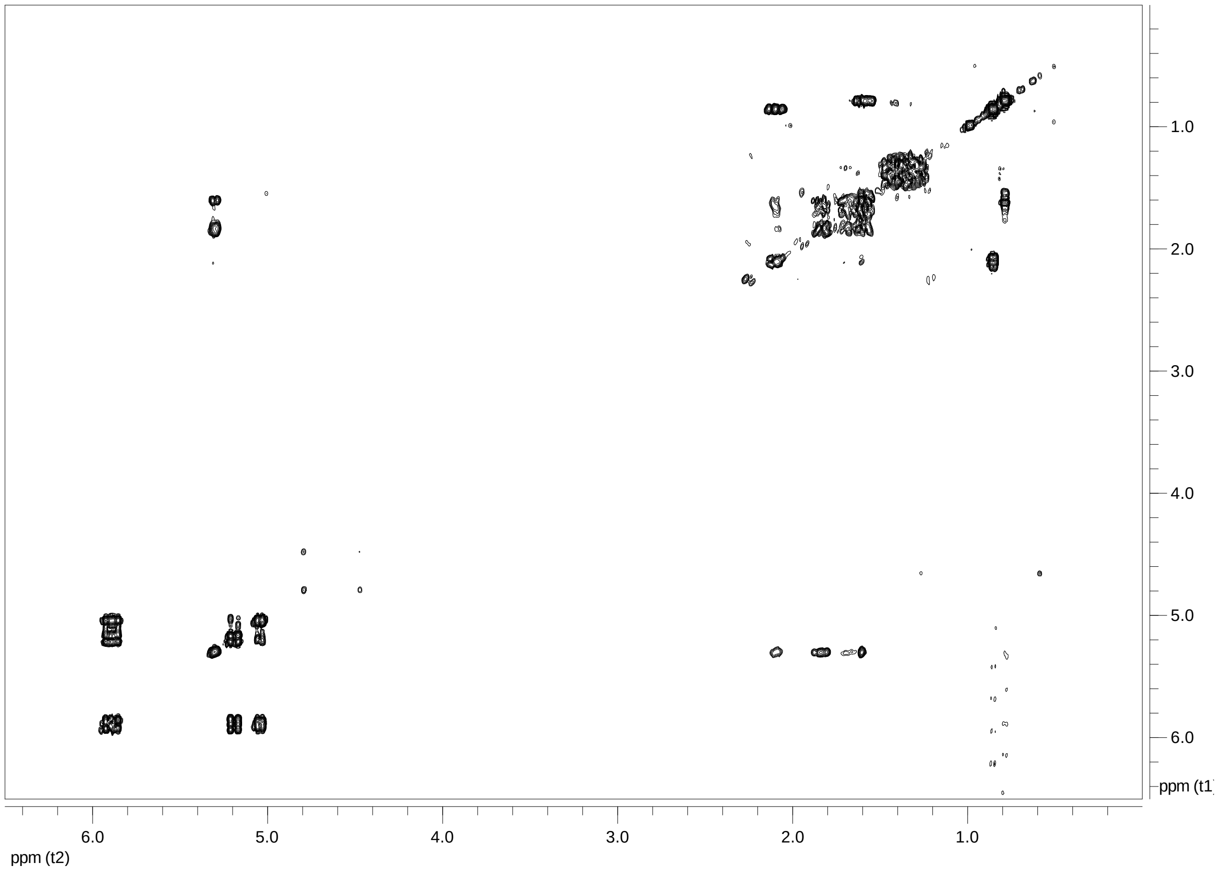

Supplementary Fig. 134. COSY spectrum (in $CDCl_3$) of compound **4b**.

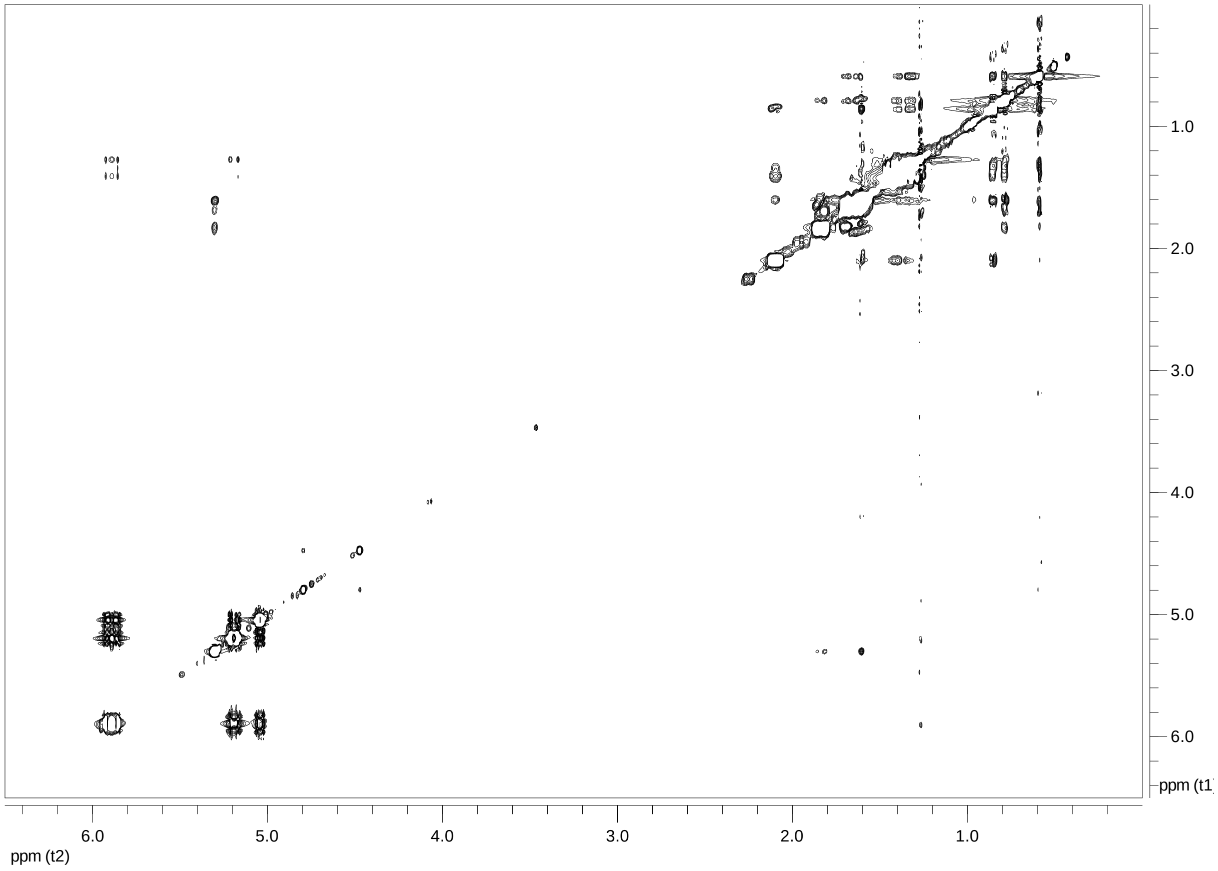

Supplementary Fig. 135. NOESY spectrum (in $CDCl_3$) of compound **4b**.

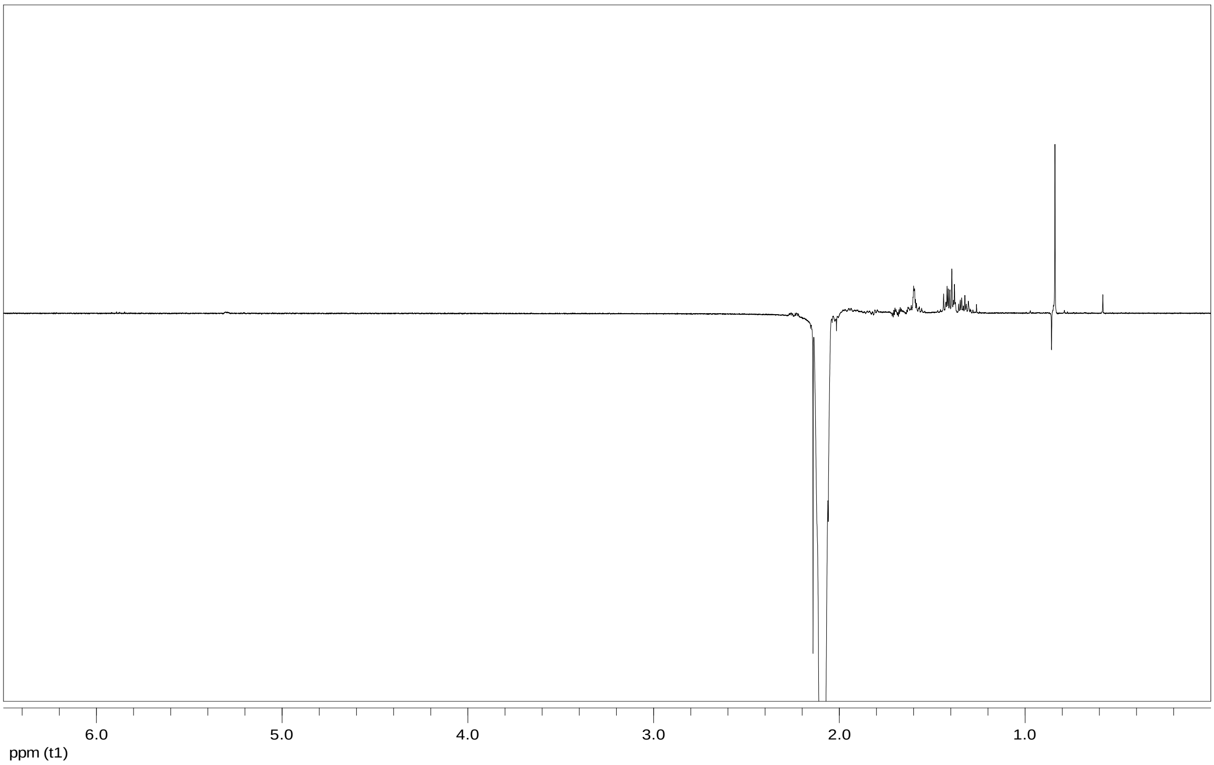

Supplementary Fig. 136. 1D NOE difference spectrum (in $CDCl_3$) of compound **4b** upon irradiation of H-7.

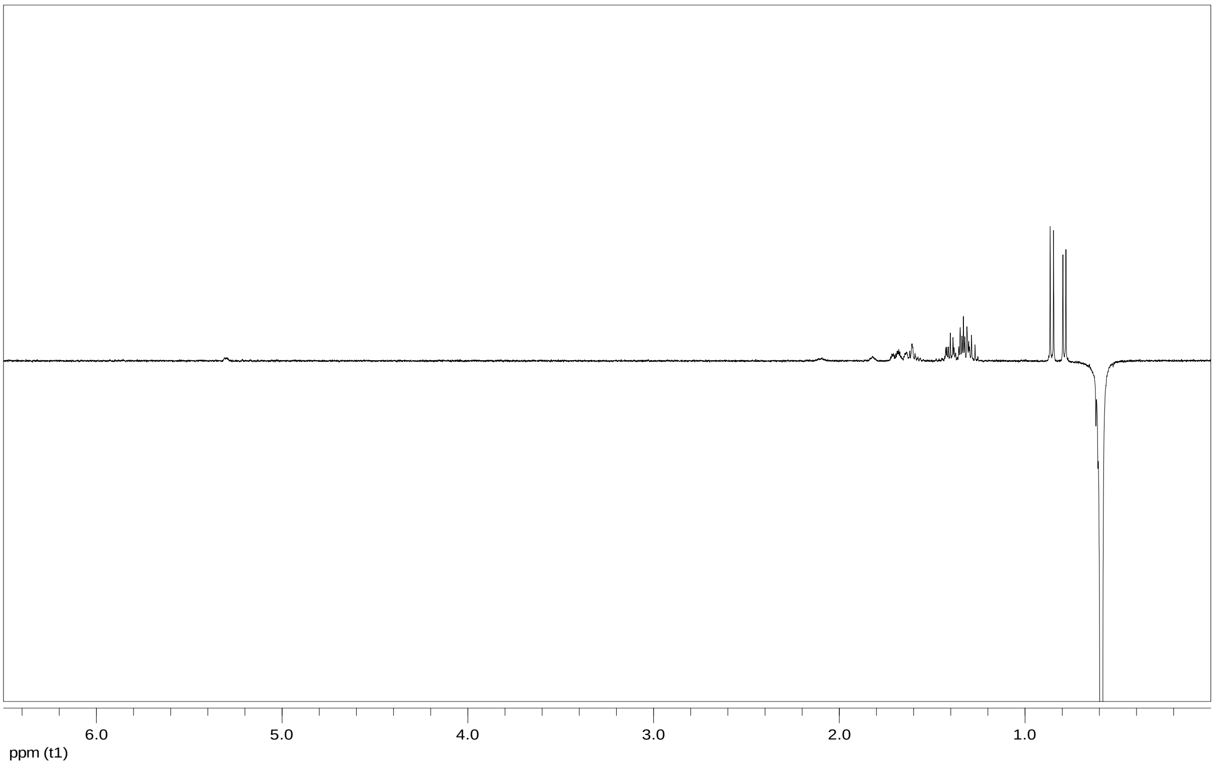

Supplementary Fig. 137. 1D NOE difference spectrum (in $CDCl_3$) of compound **4b** upon irradiation of H_3-12.

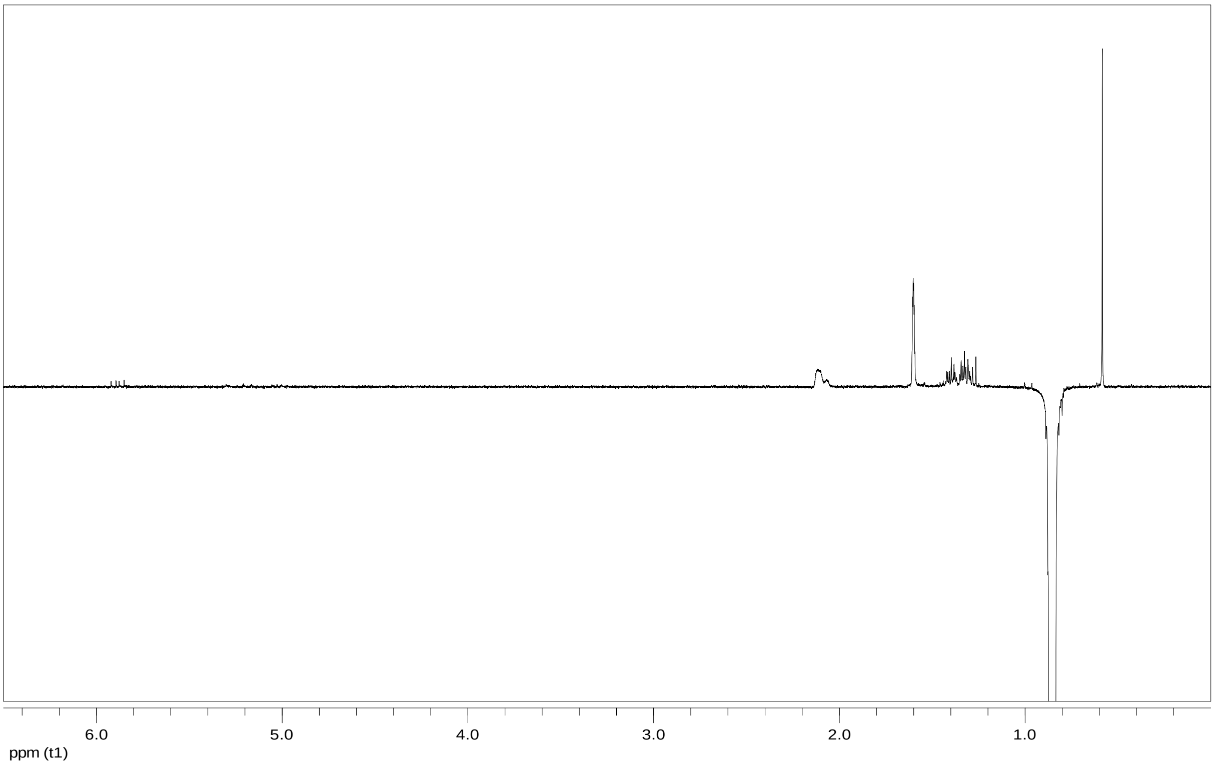

Supplementary Fig. 138. 1D NOE difference spectrum (in $CDCl_3$) of compound **4b** upon irradiation of H_3-13.

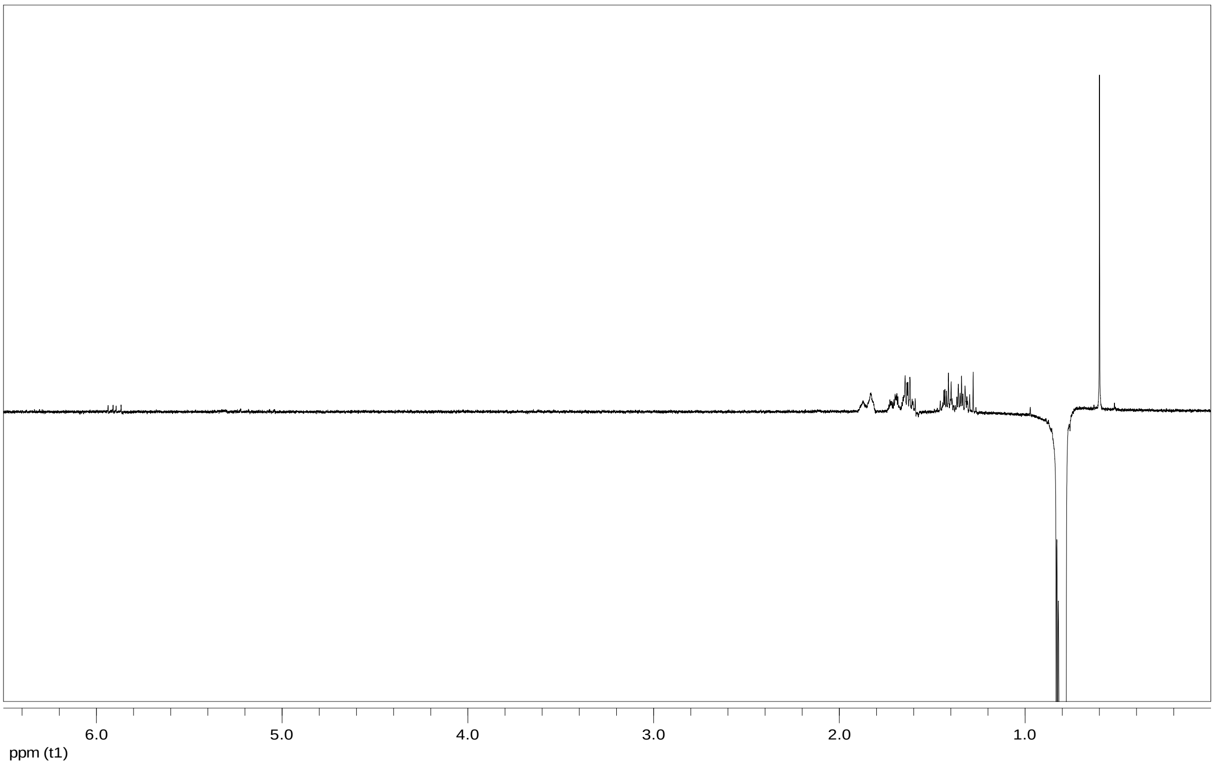

Supplementary Fig. 139. 1D NOE difference spectrum (in $CDCl_3$) of compound **4b** upon irradiation of H_3-15.

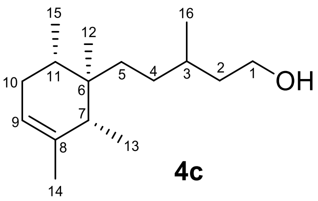

4c

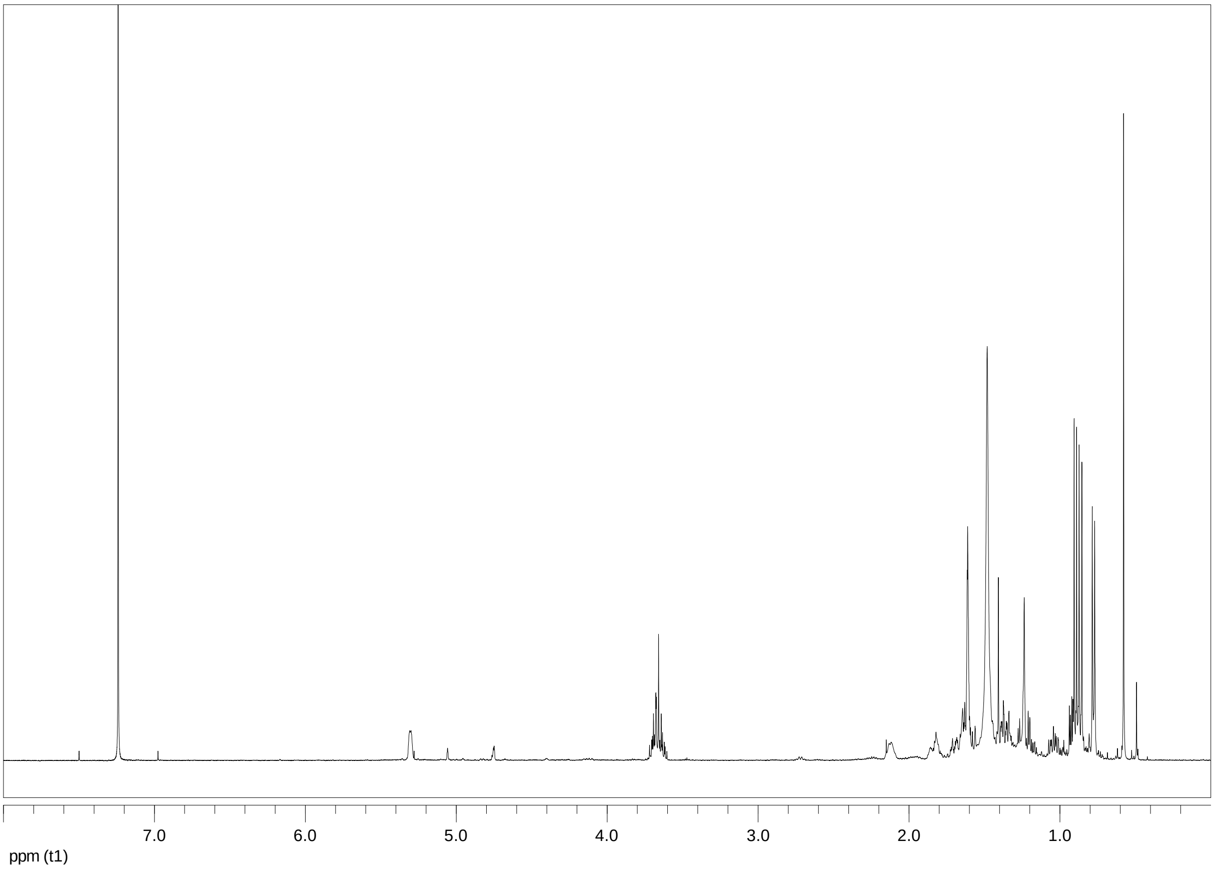

Supplementary Fig. 140. ^{1}H NMR spectrum (in $CDCl_3$) of compound **4c**.

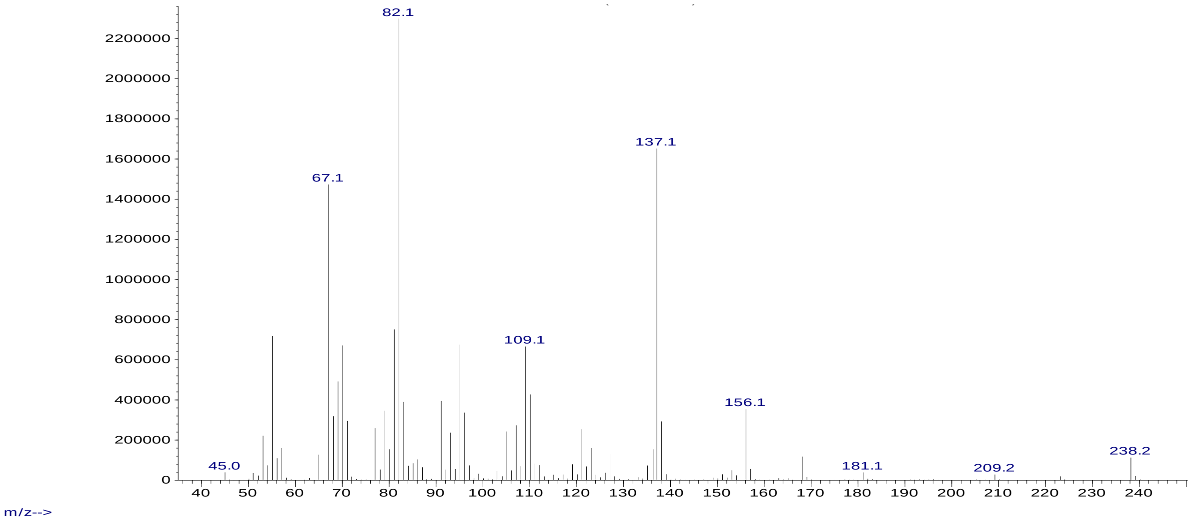

Supplementary Fig. 141. LR-EI-MS spectrum of compound **4c**.

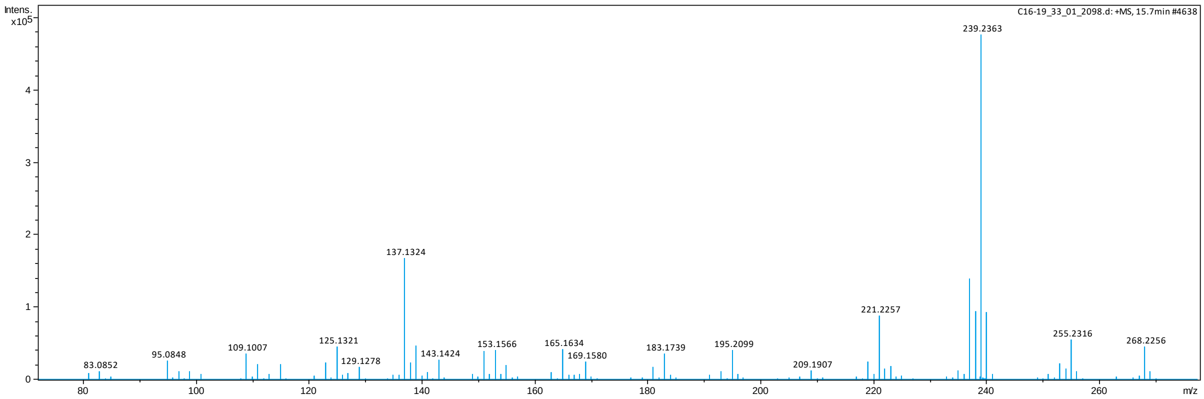

Supplementary Fig. 142. HR-APCI-MS spectrum of compound **4c**.

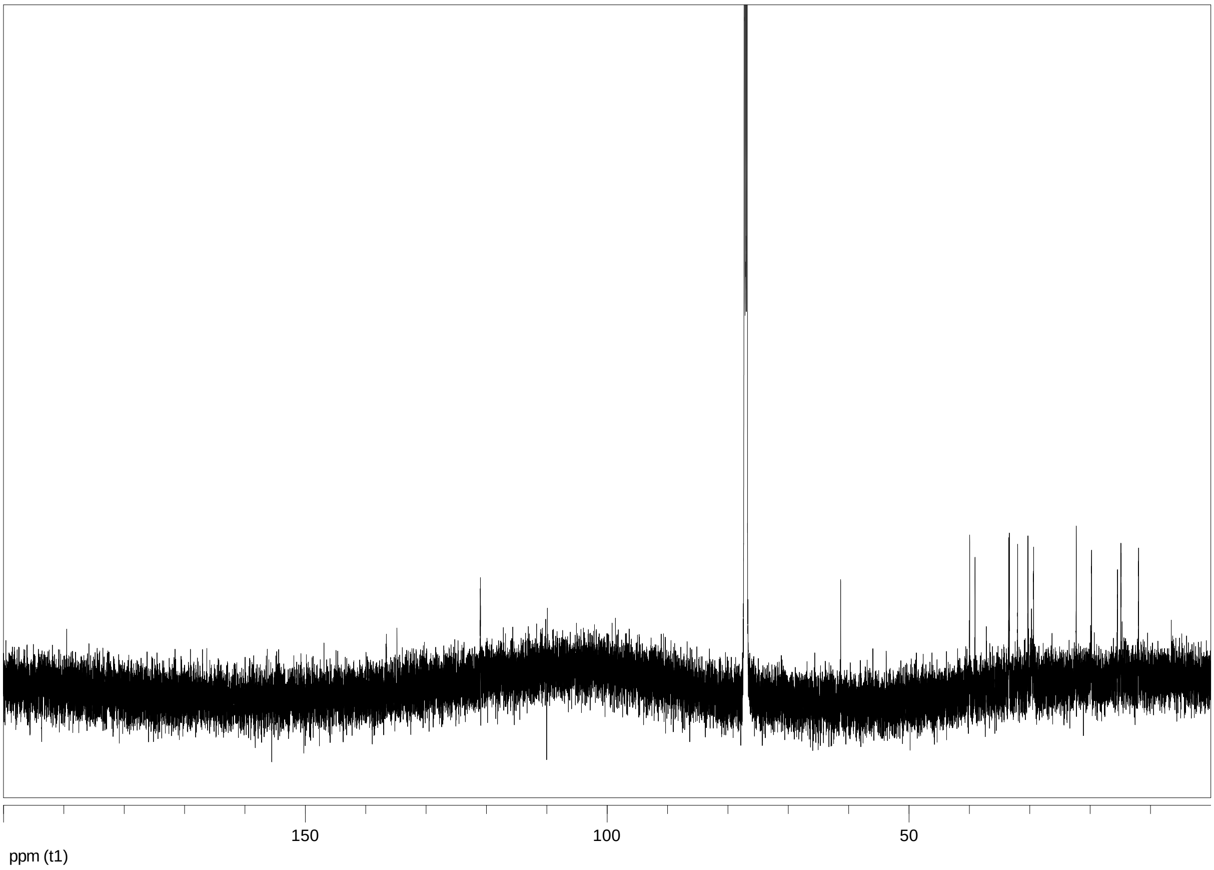

Supplementary Fig. 143. ^{13}C NMR spectrum (in $CDCl_3$) of compound **4c**.

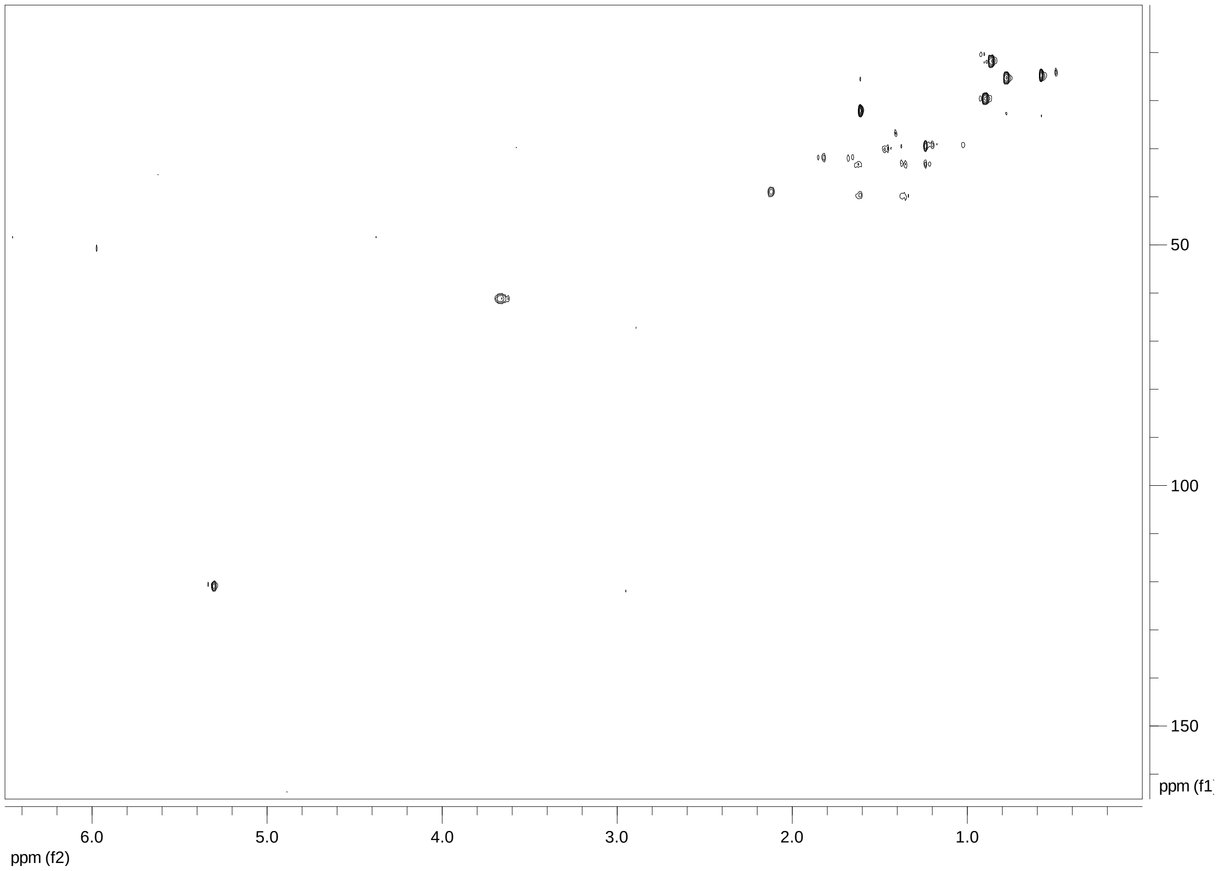

Supplementary Fig. 144. HSQC spectrum (in $CDCl_3$) of compound **4c**.

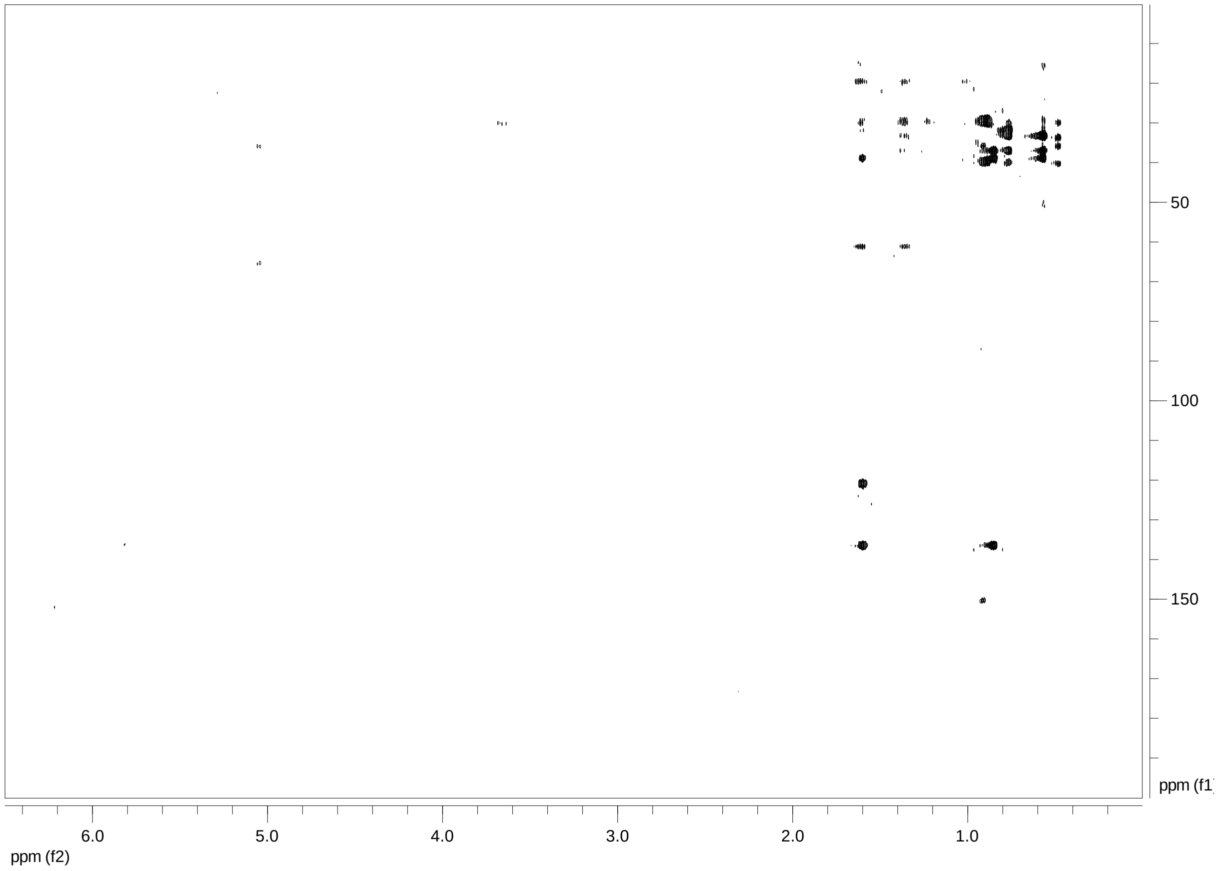

Supplementary Fig. 145. HMBC spectrum (in $CDCl_3$) of compound **4c**.

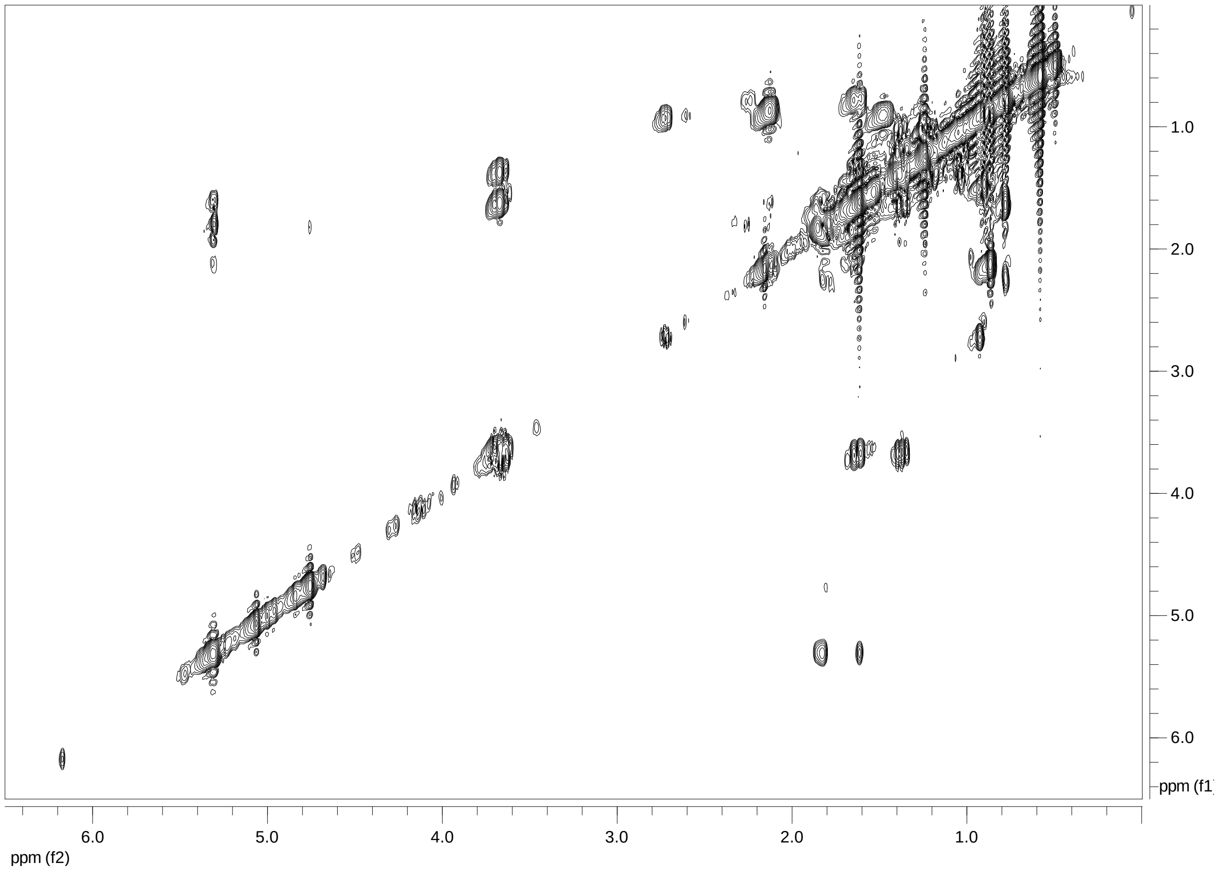

Supplementary Fig. 146. COSY spectrum (in $CDCl_3$) of compound **4c**.

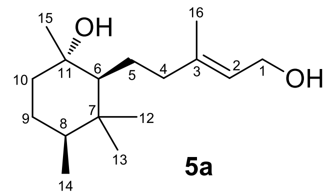

5a

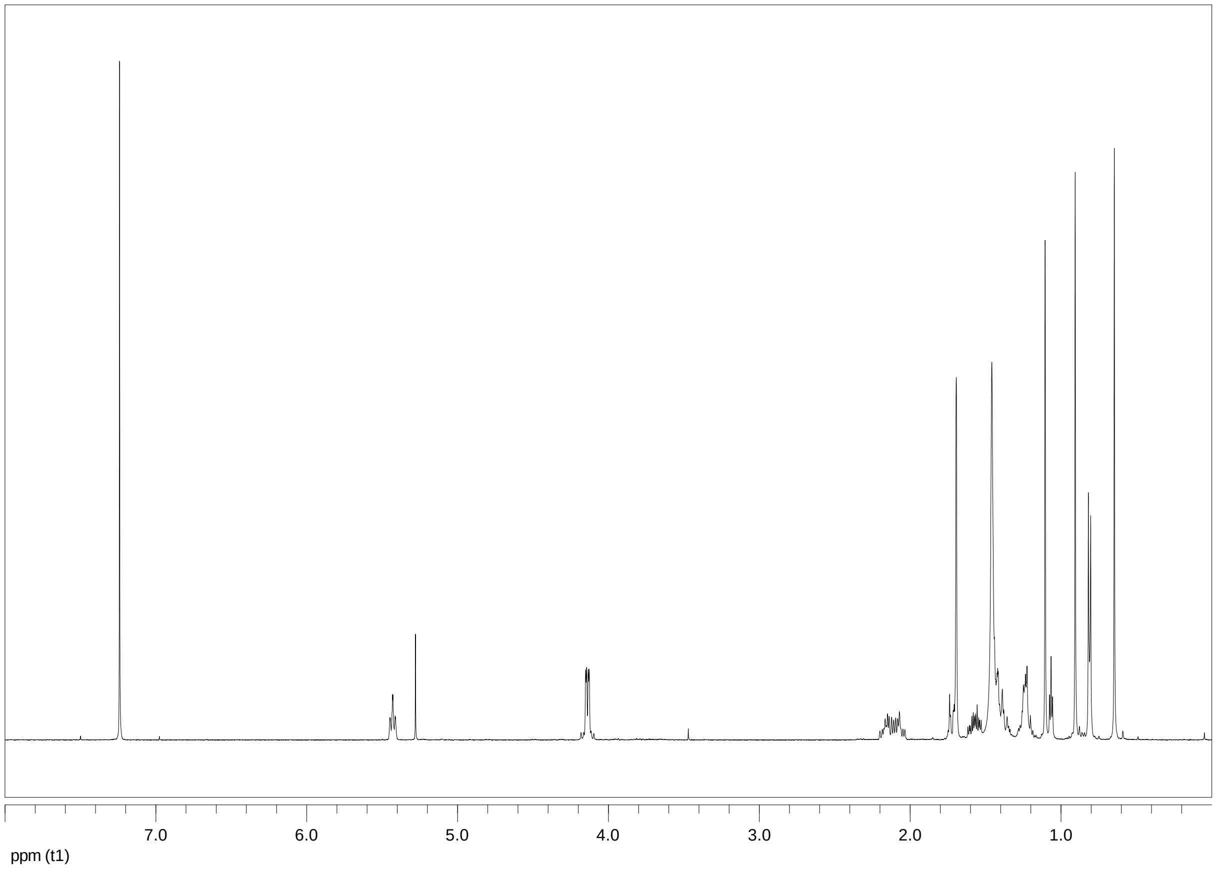

Supplementary Fig. 147. ^{1}H NMR spectrum (in $CDCl_3$) of compound **5a**.

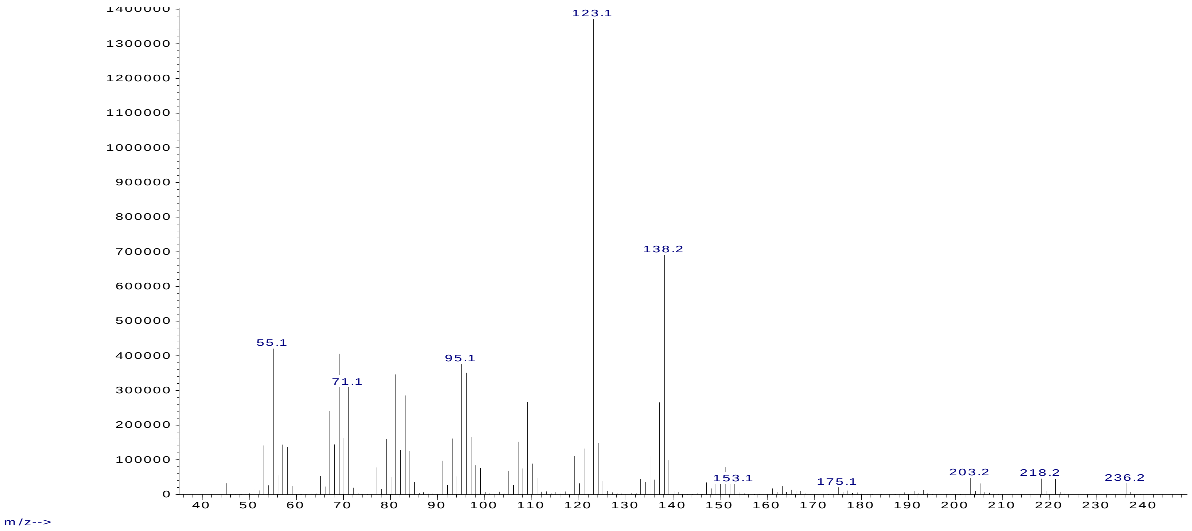

Supplementary Fig. 148. LR-EI-MS spectrum of compound **5a**.

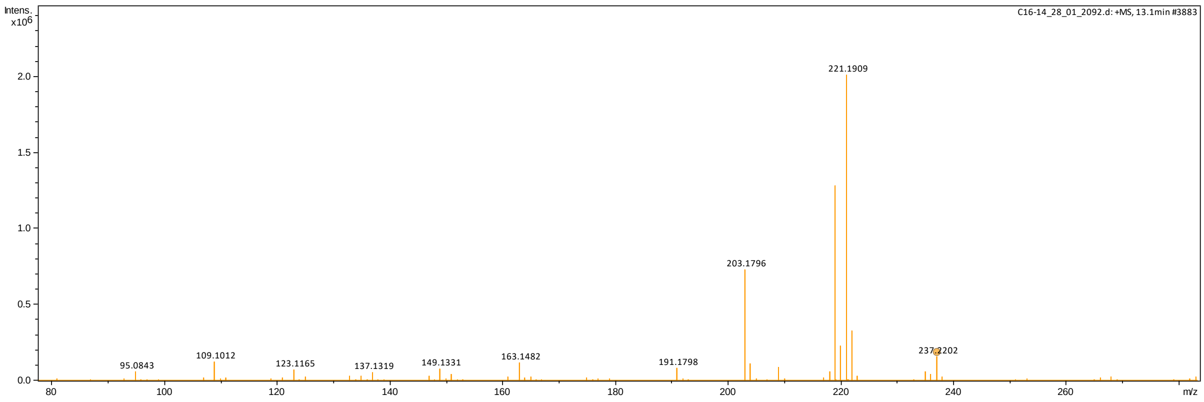

Supplementary Fig. 149. HR-APCI-MS spectrum of compound **5a**.

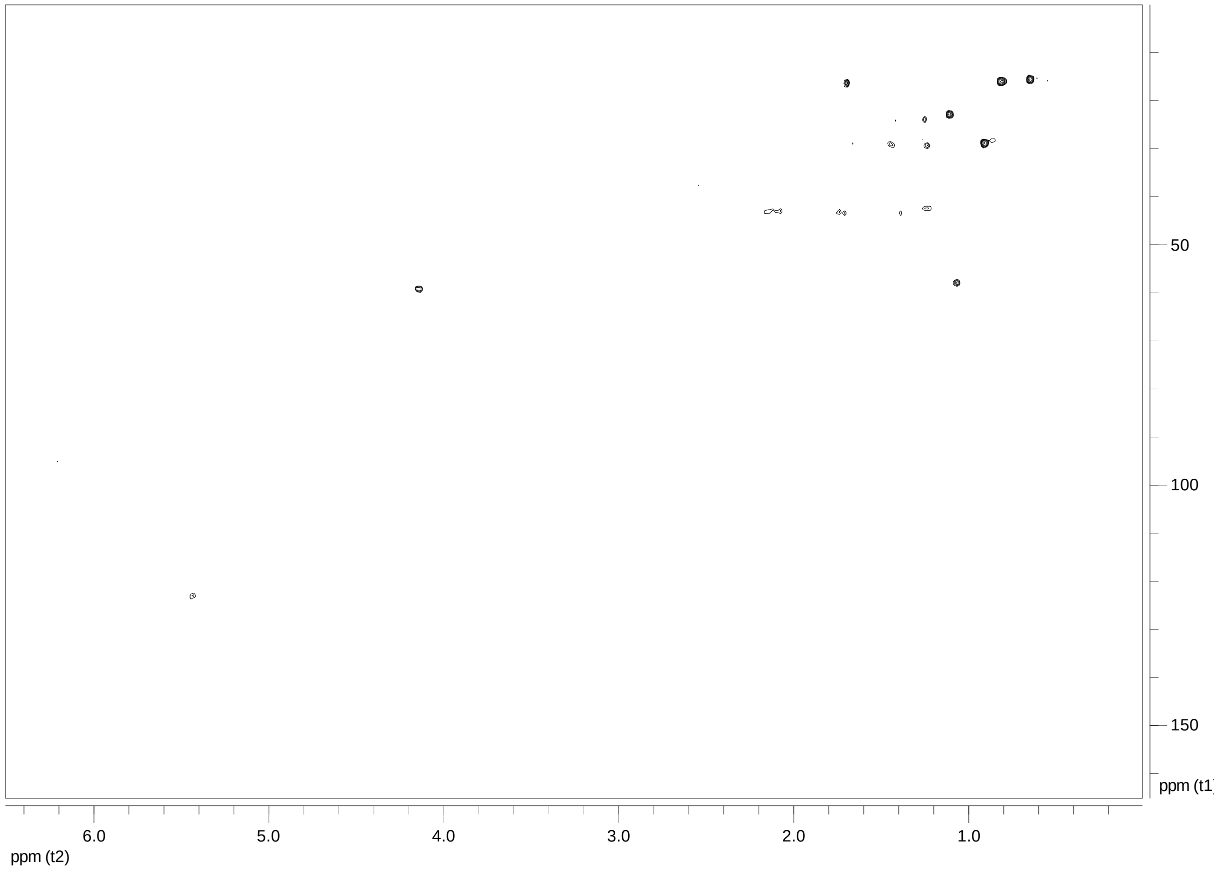

Supplementary Fig. 150. HSQC spectrum (in $CDCl_3$) of compound **5a**.

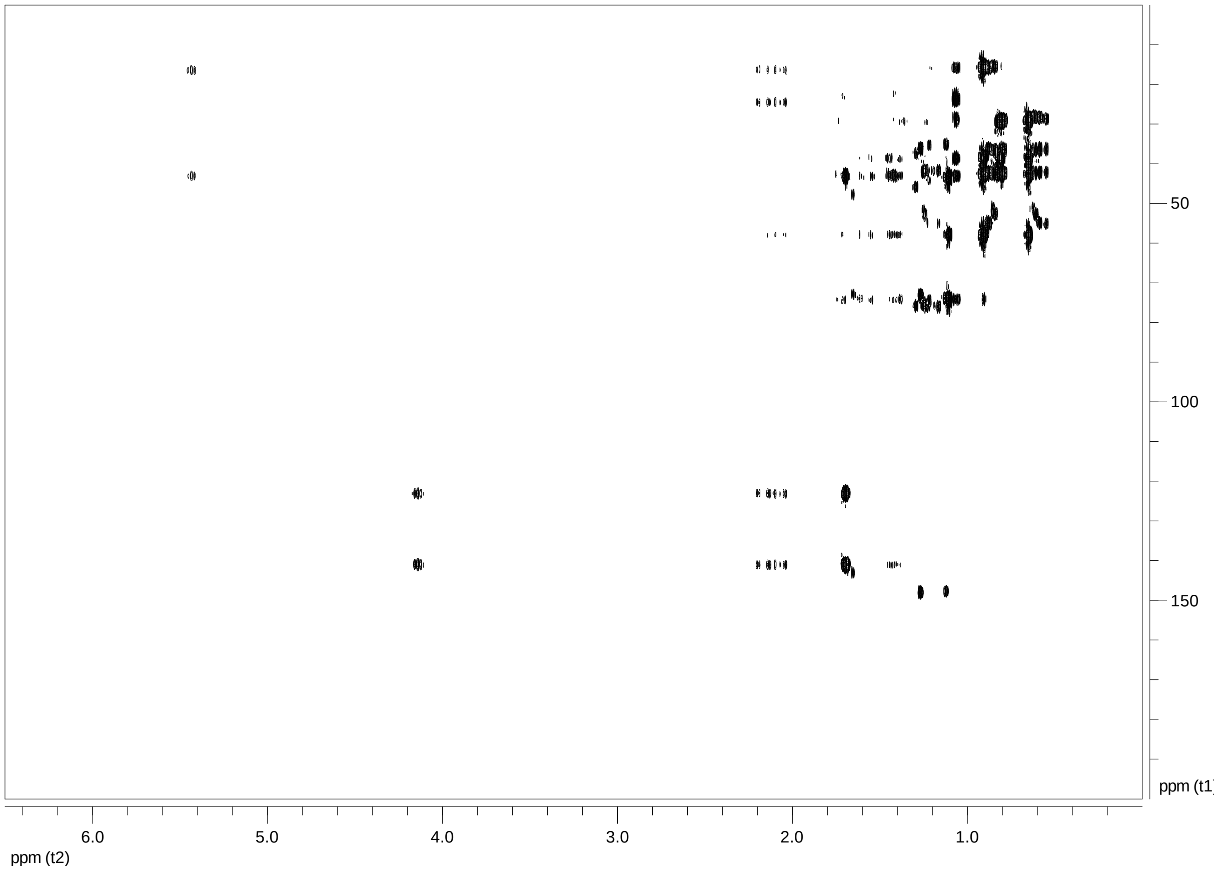

Supplementary Fig. 151. HMBC spectrum (in $CDCl_3$) of compound **5a**.

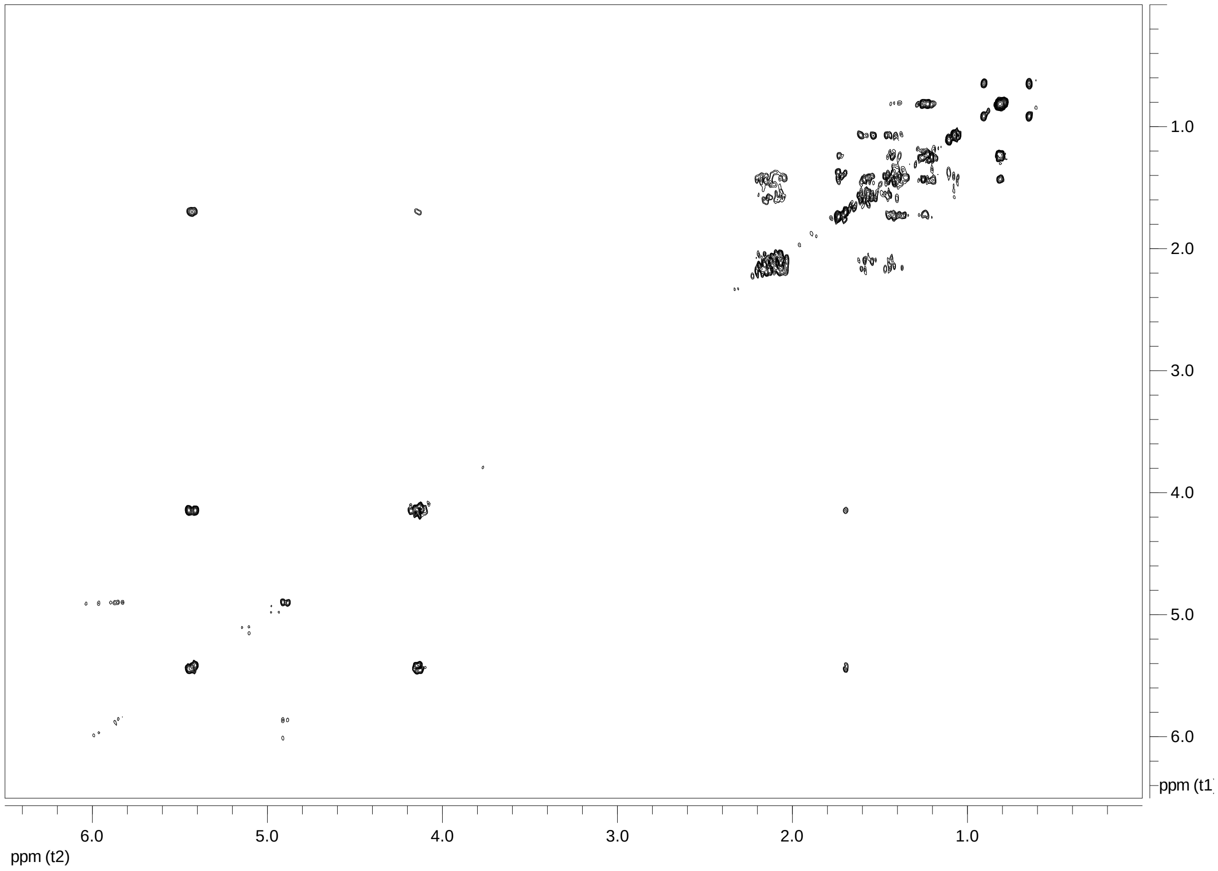

Supplementary Fig. 152. COSY spectrum (in $CDCl_3$) of compound **5a**.

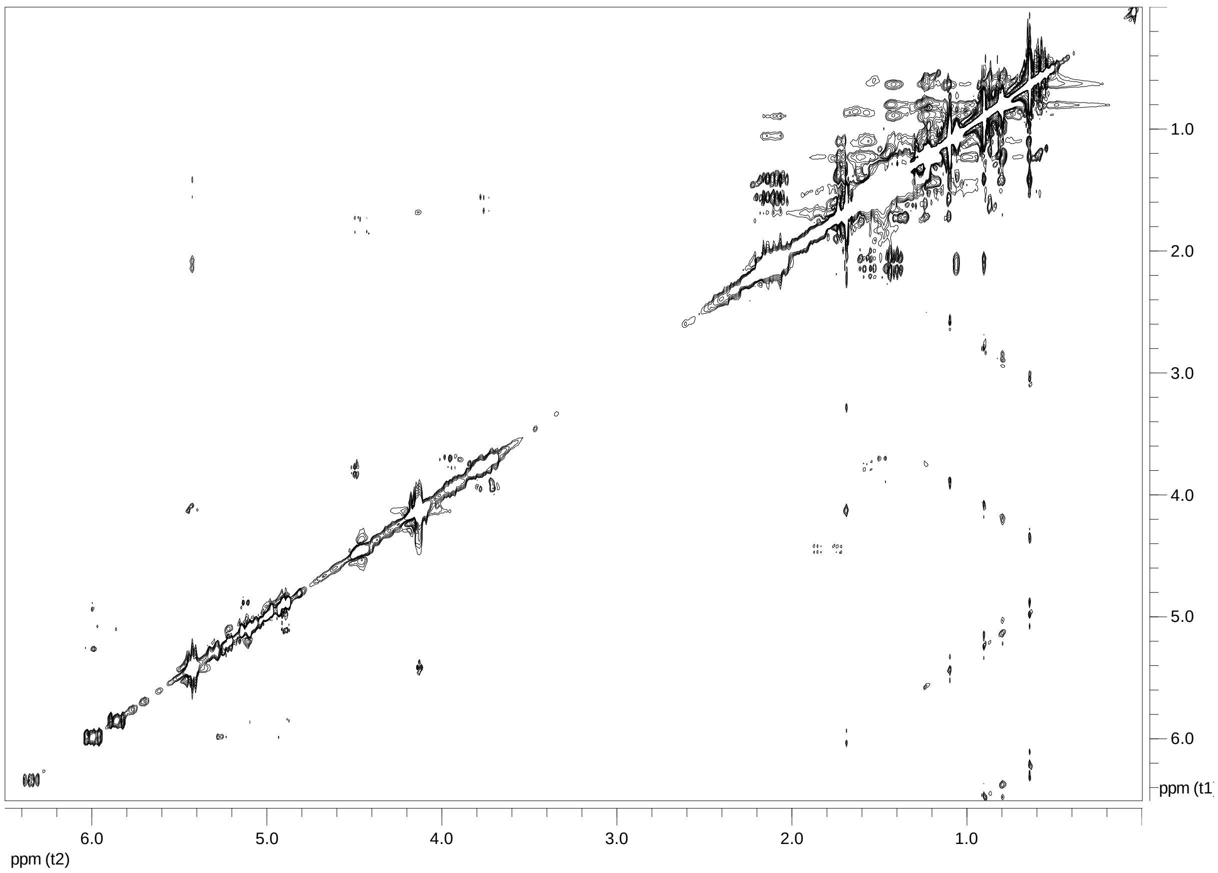

Supplementary Fig. 153. NOESY spectrum (in $CDCl_3$) of compound **5a**.

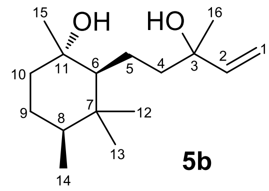

5b

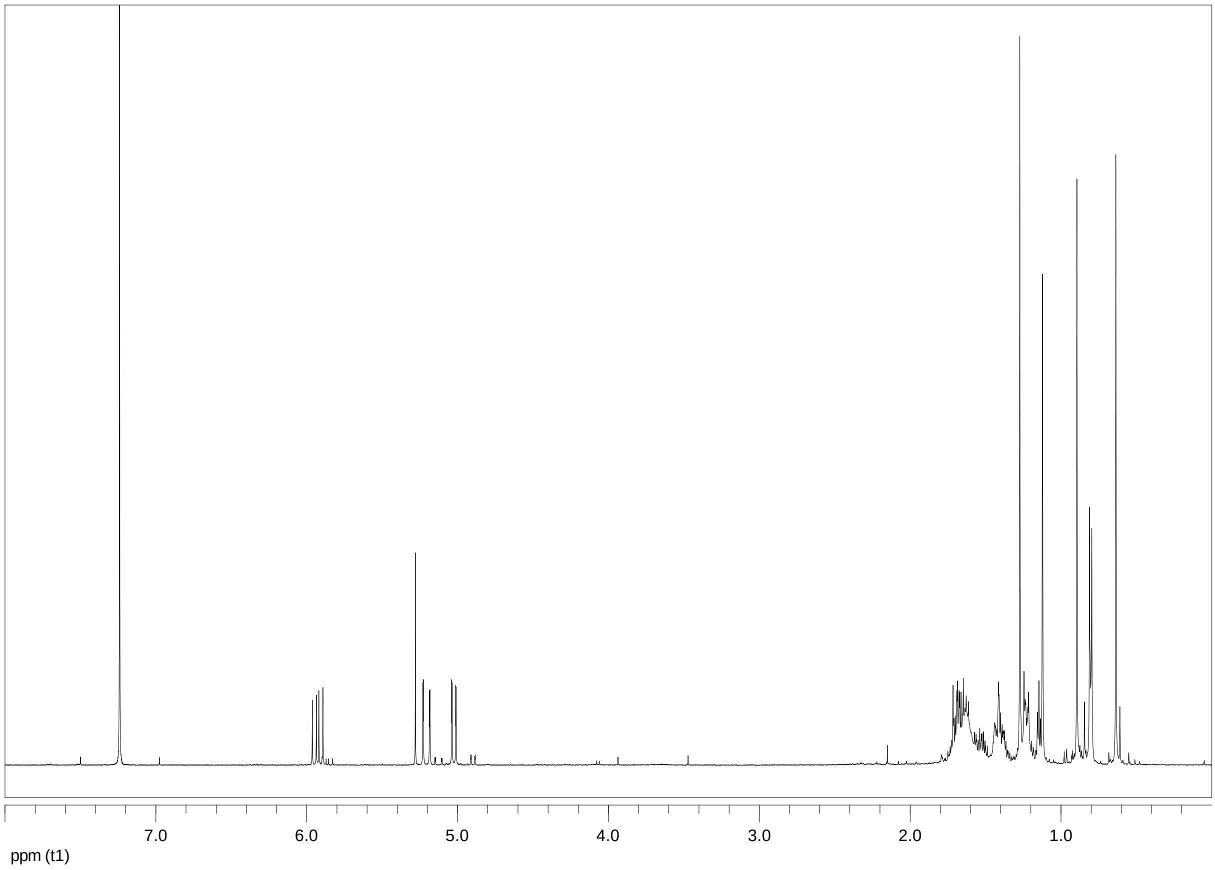

Supplementary Fig. 154. ^{1}H NMR spectrum (in $CDCl_3$) of compound **5b**.

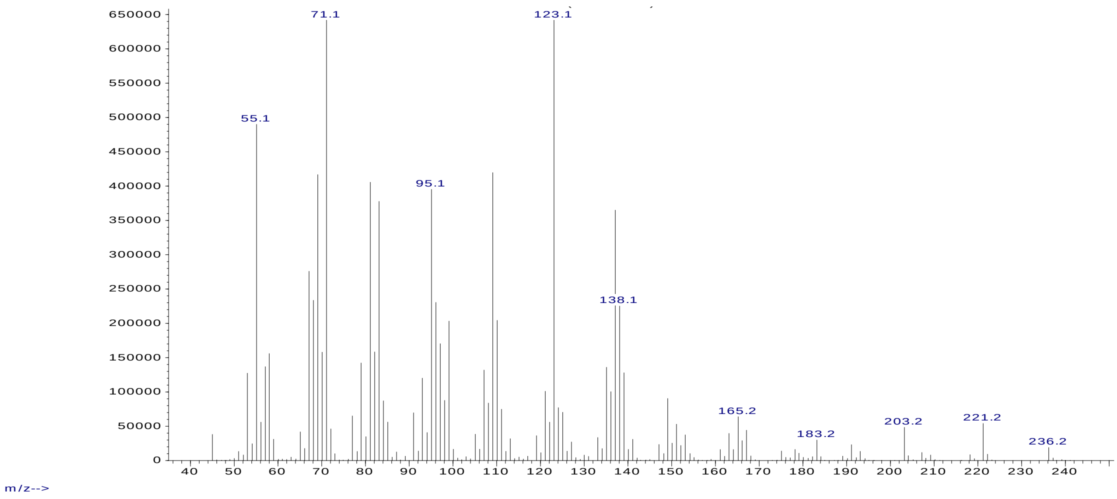

Supplementary Fig. 155. LR-EI-MS spectrum of compound **5b**.

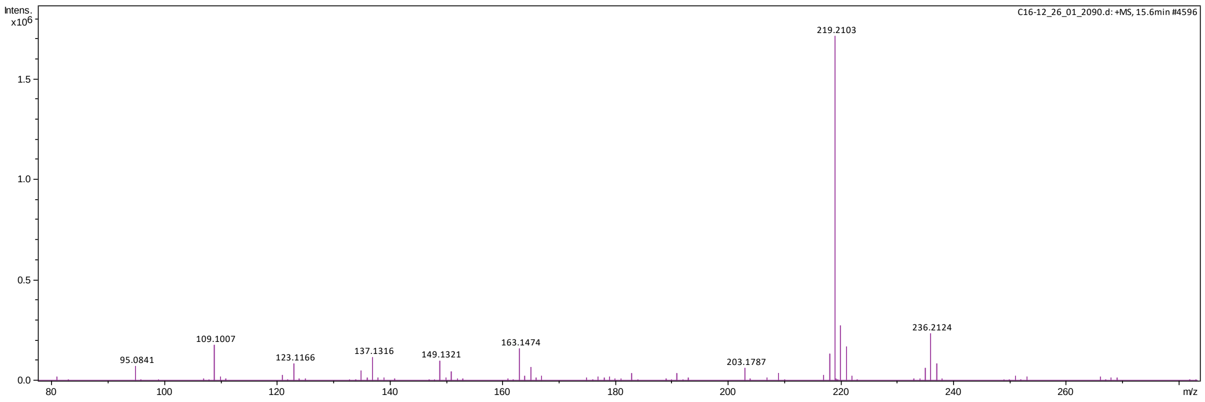

Supplementary Fig. 156. HR-APCI-MS spectrum of compound **5b**.

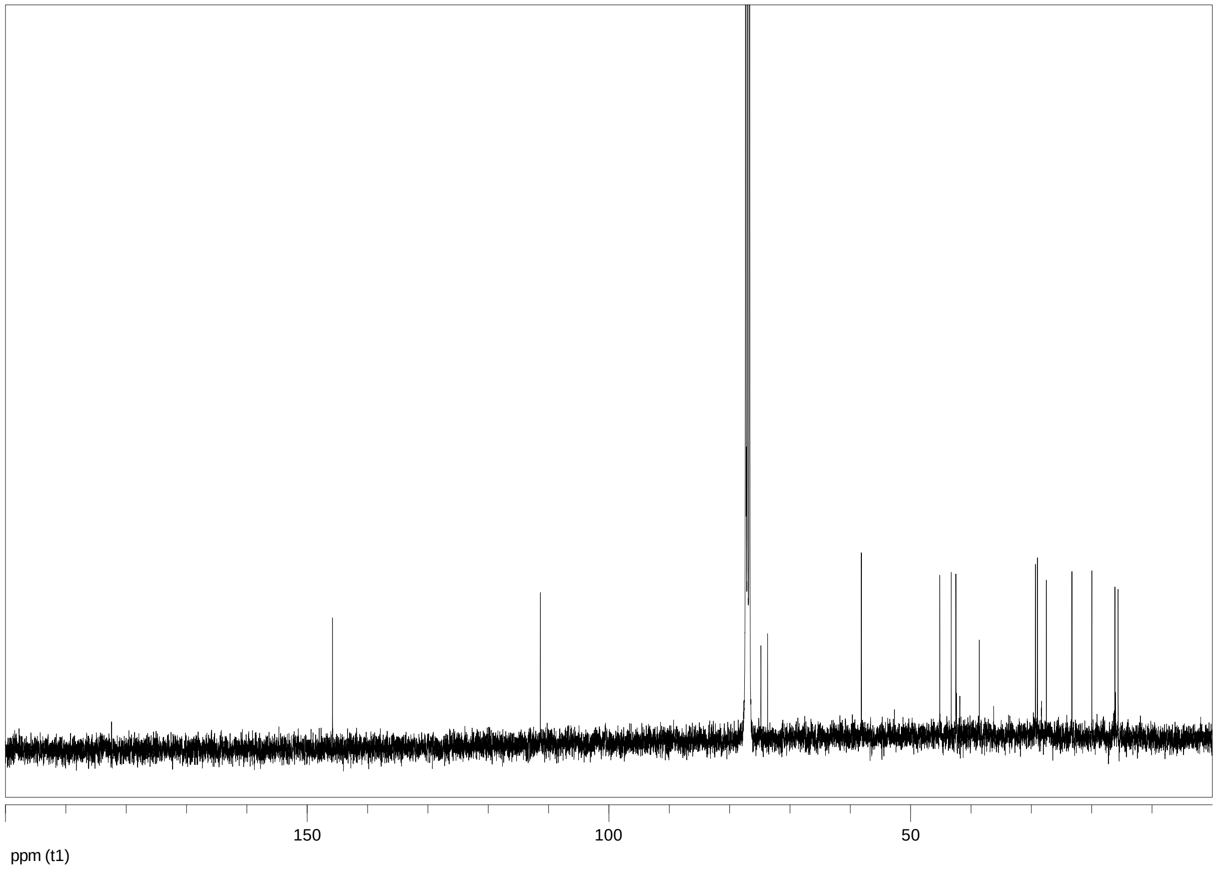

Supplementary Fig. 157. ^{13}C NMR spectrum (in $CDCl_3$) of compound **5b**.

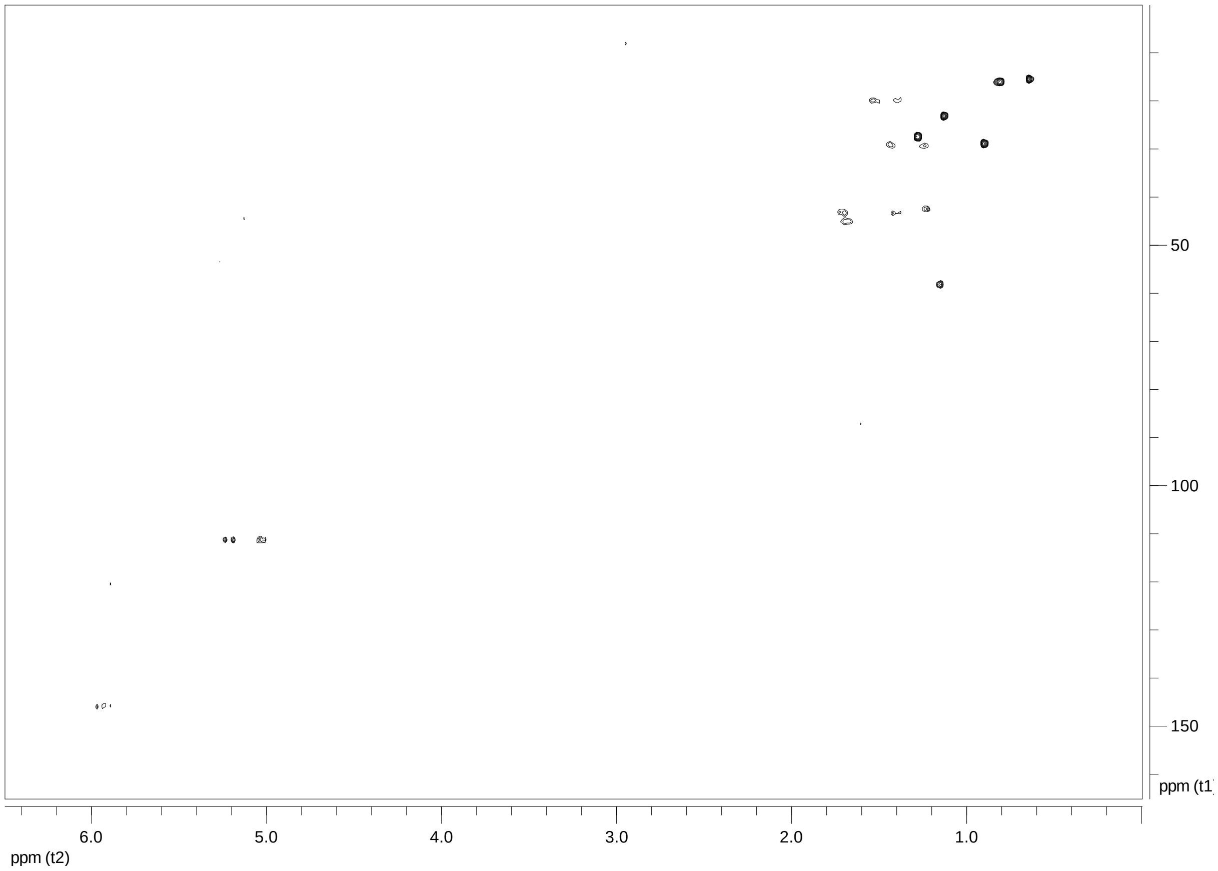

Supplementary Fig. 158. HSQC spectrum (in $CDCl_3$) of compound **5b**.

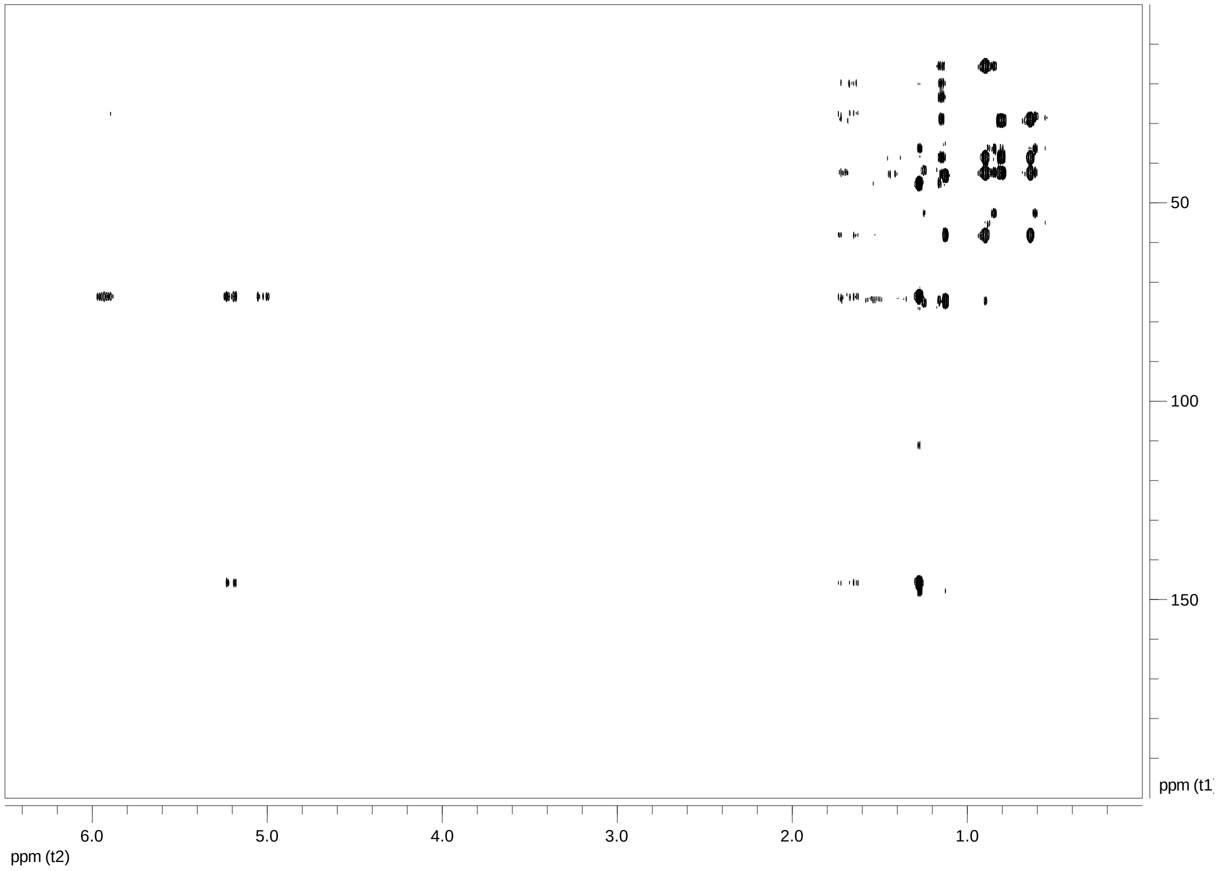

Supplementary Fig. 159. HMBC spectrum (in $CDCl_3$) of compound **5b**.

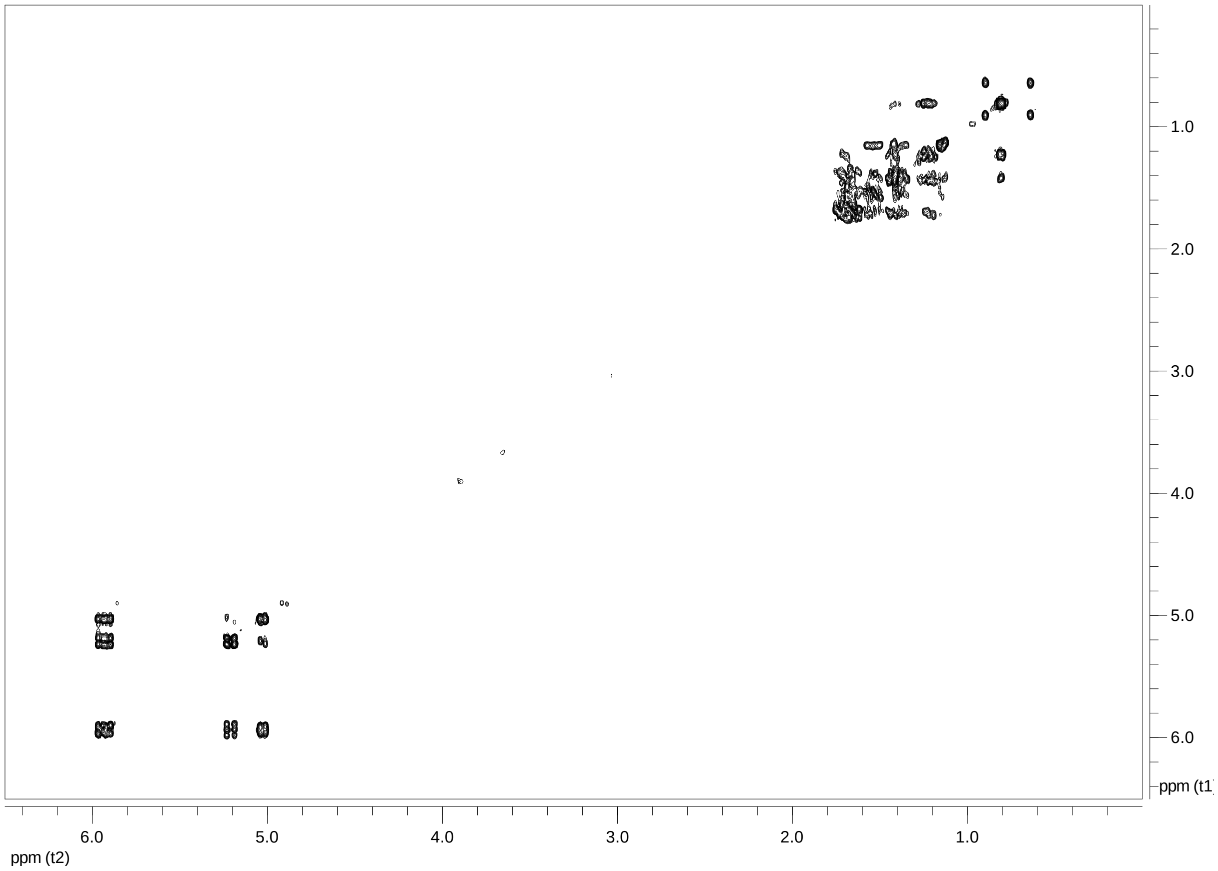

Supplementary Fig. 160. COSY spectrum (in $CDCl_3$) of compound **5b**.

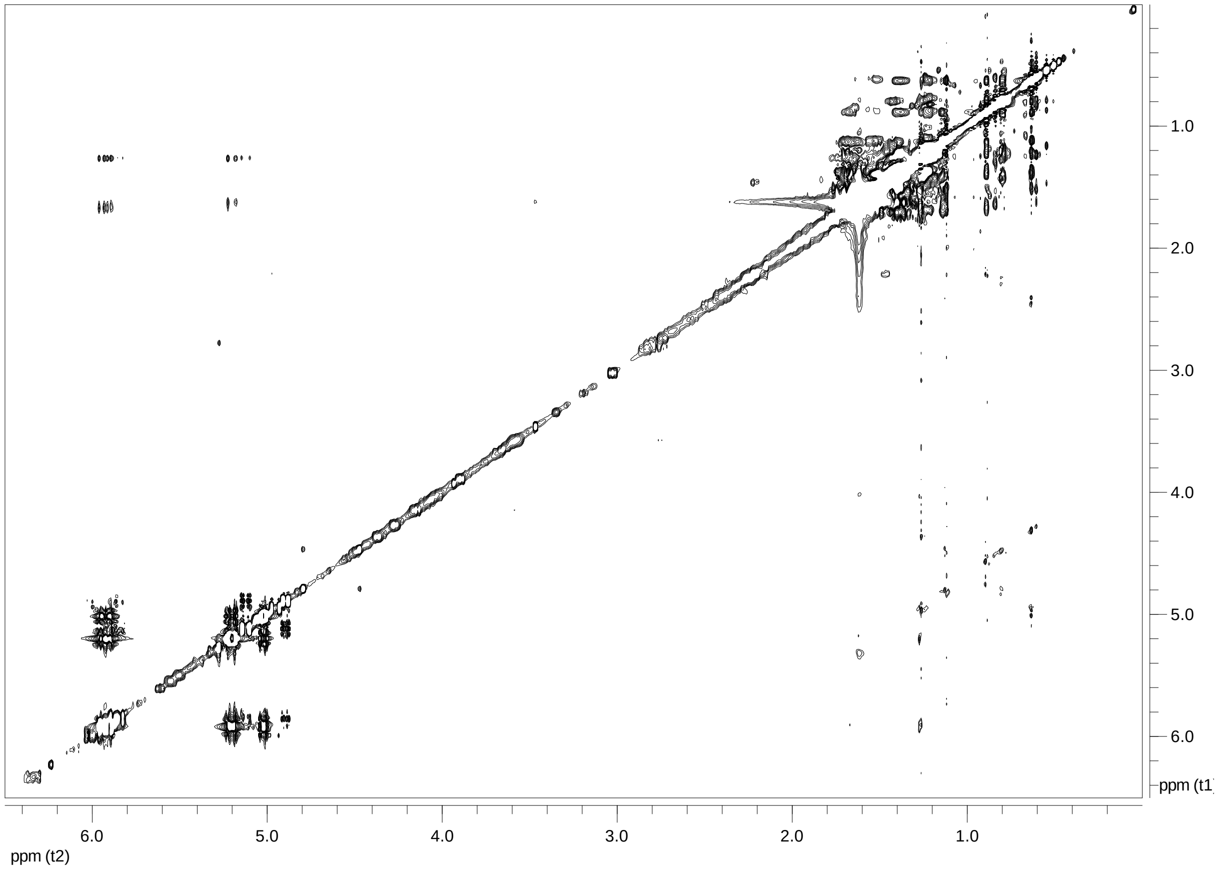

Supplementary Fig. 161. NOESY spectrum (in $CDCl_3$) of compound **5b**.

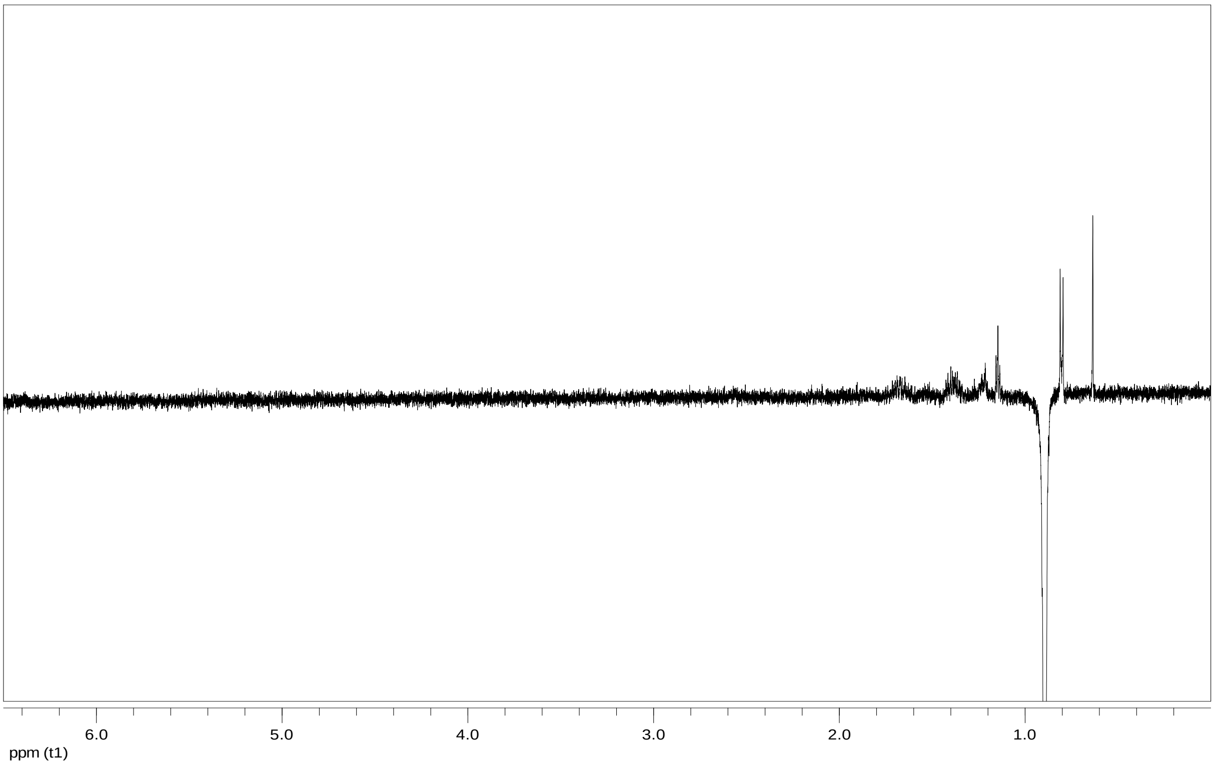

Supplementary Fig. 162. 1D NOE difference spectrum (in $CDCl_3$) of compound **5b** upon irradiation of H_3-12.

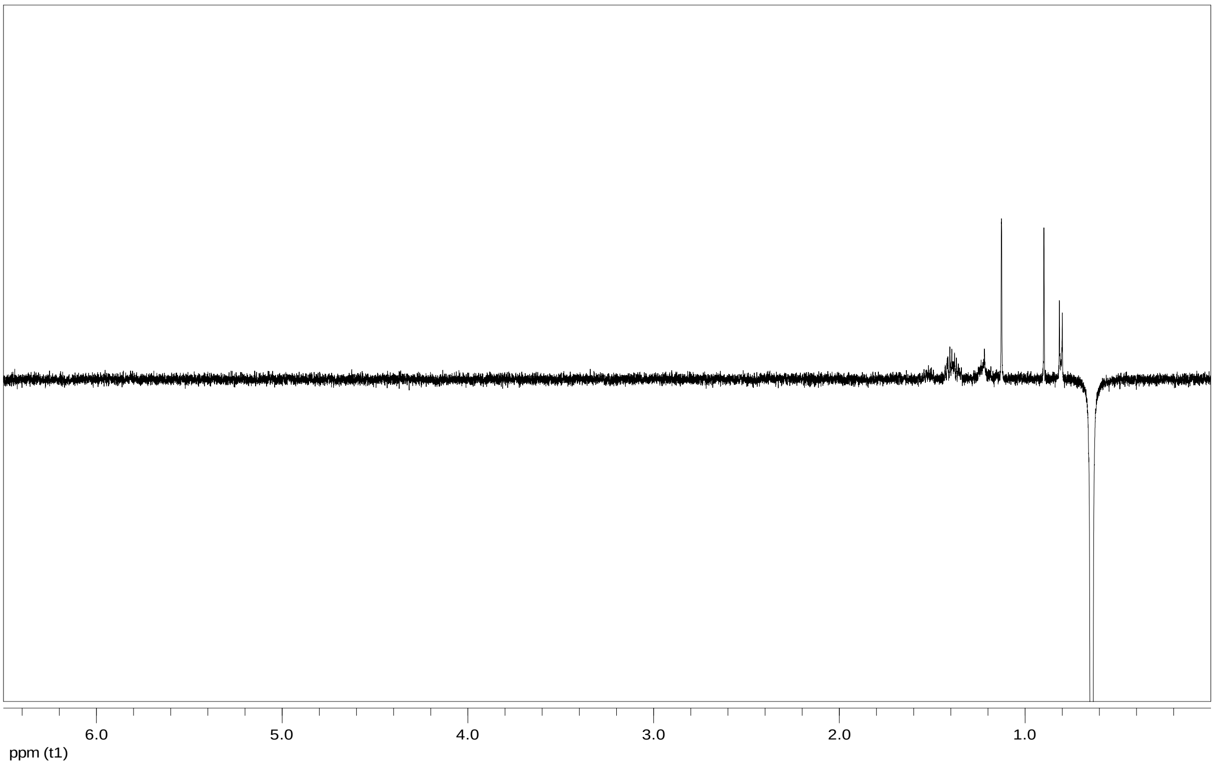

Supplementary Fig. 163. 1D NOE difference spectrum (in $CDCl_3$) of compound **5b** upon irradiation of H_3-13.

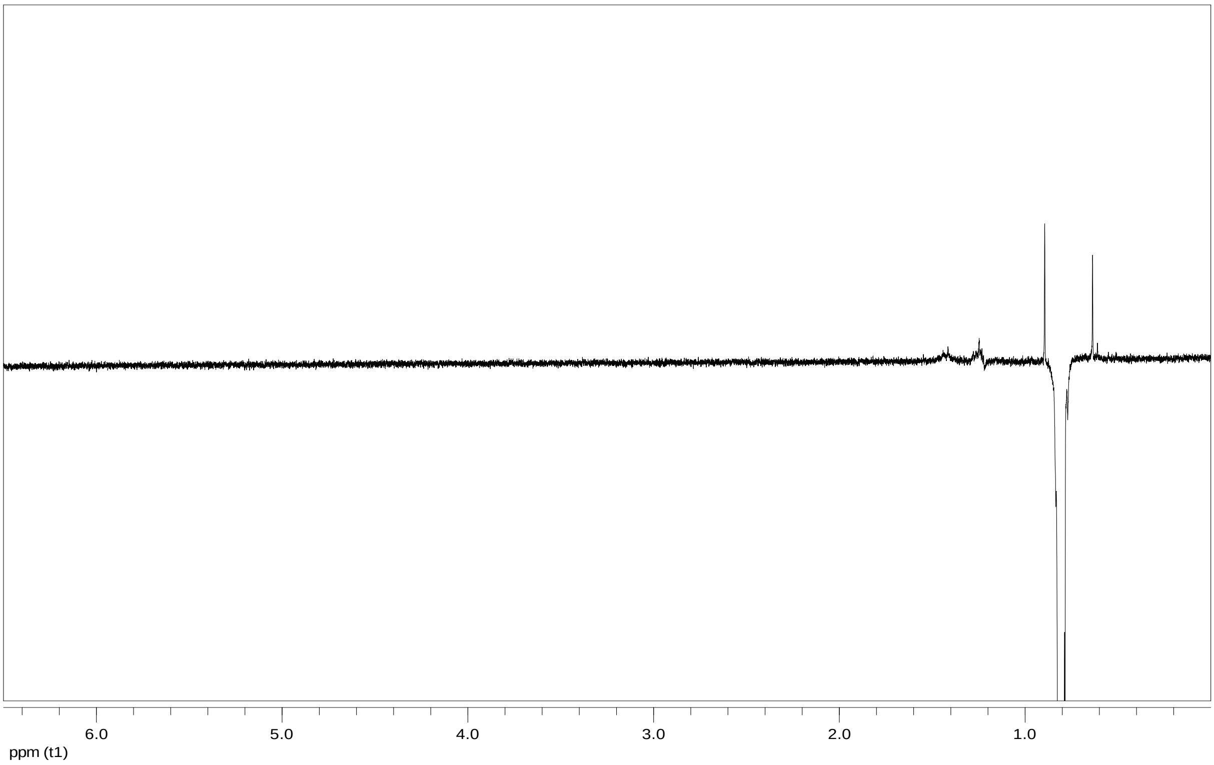

Supplementary Fig. 164. 1D NOE difference spectrum (in $CDCl_3$) of compound **5b** upon irradiation of H_3-14.

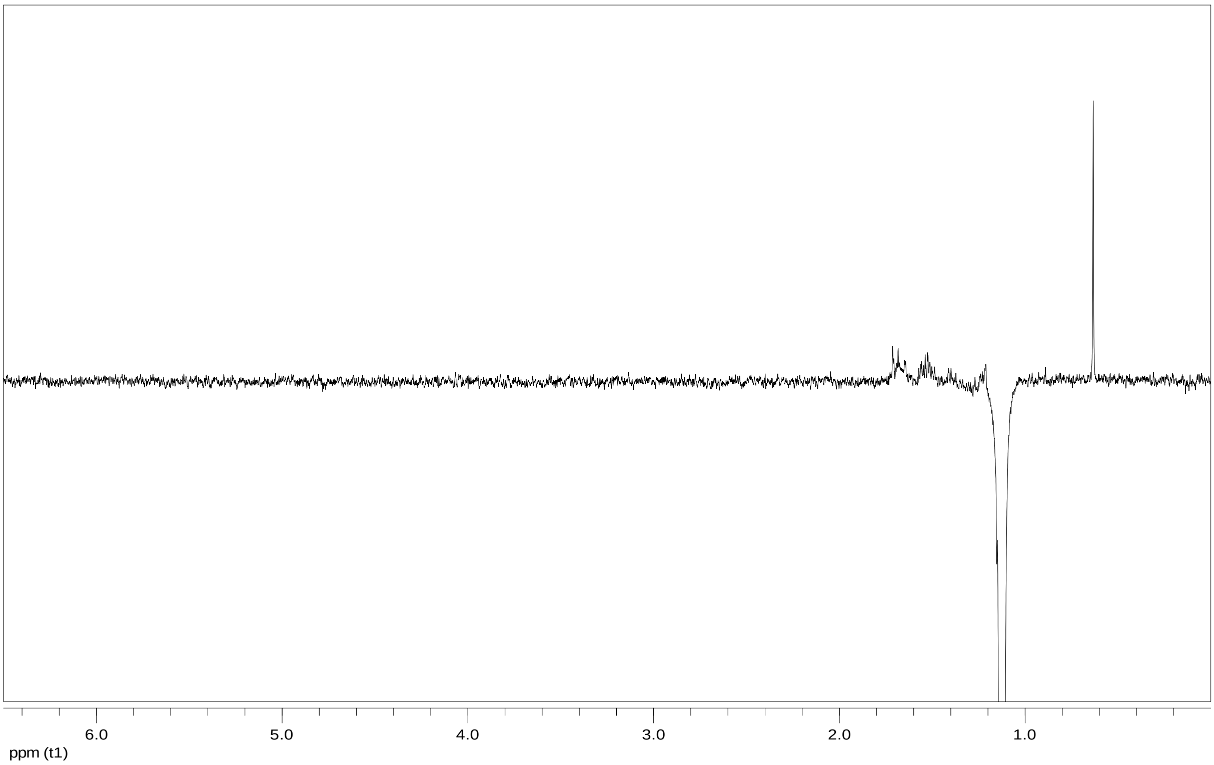

Supplementary Fig. 165. 1D NOE difference spectrum (in $CDCl_3$) of compound **5b** upon irradiation of H_3-15.

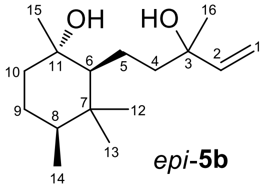

epi-**5b**

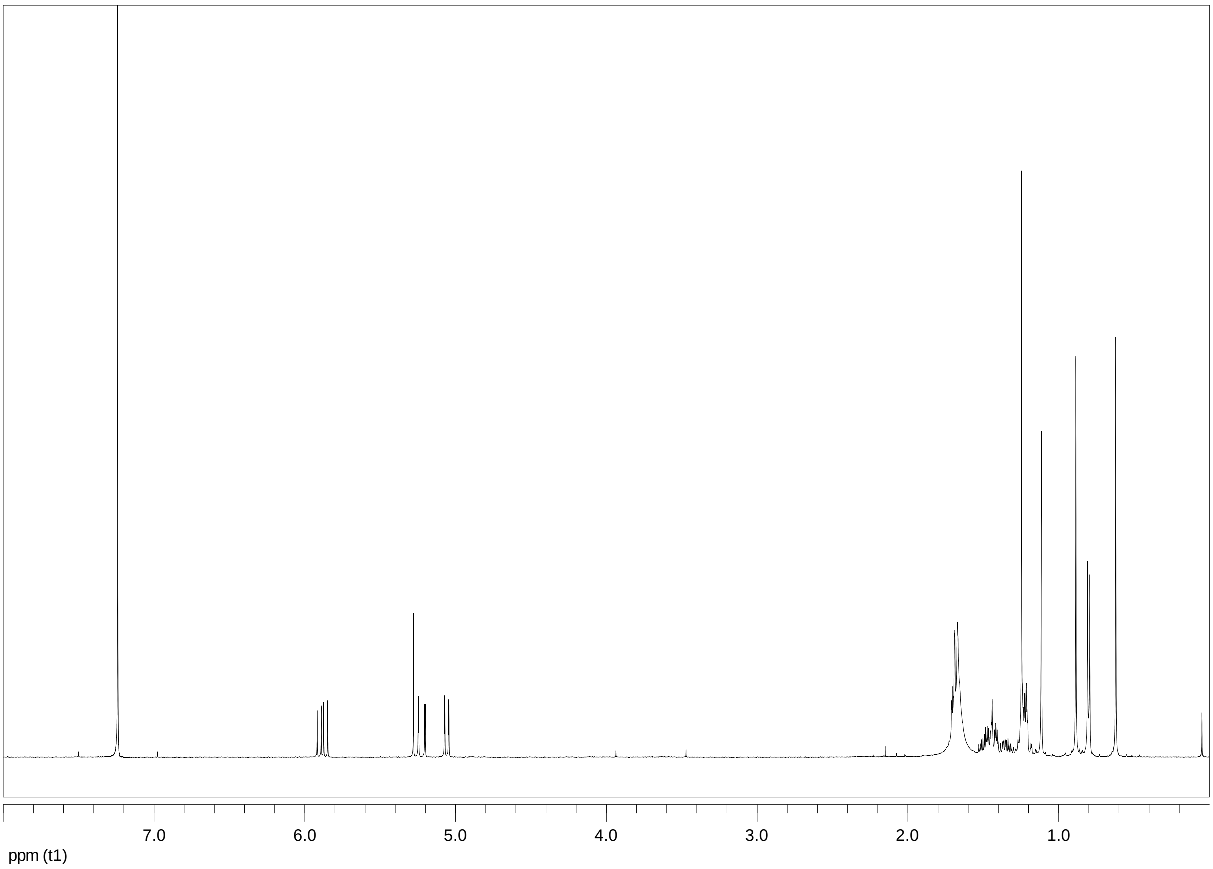

Supplementary Fig. 166. ^{1}H NMR spectrum (in $CDCl_3$) of compound ***epi*-5b**.

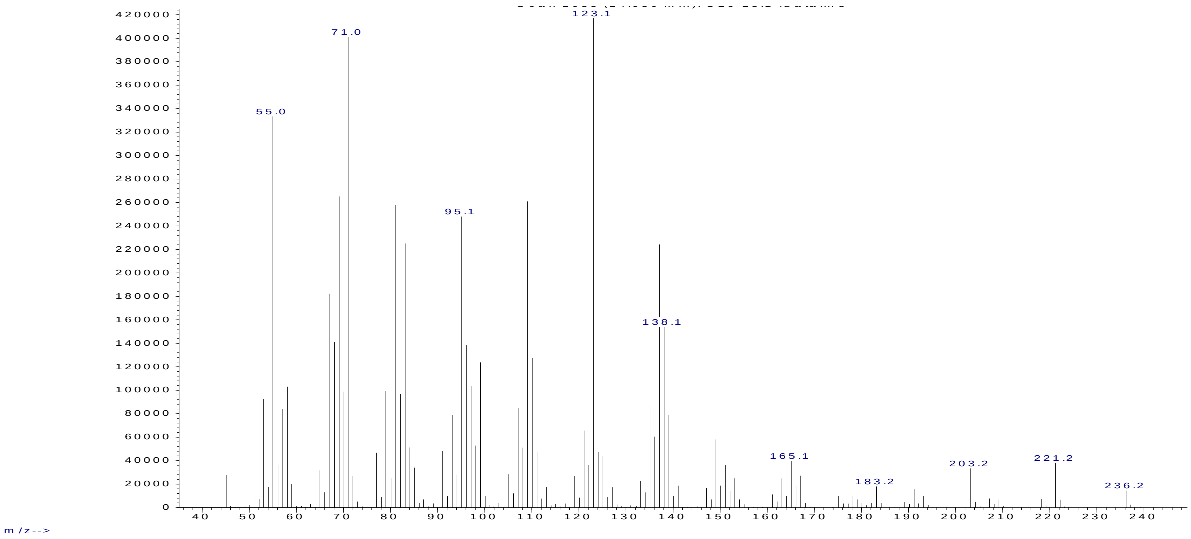

Supplementary Fig. 167. LR-EI-MS spectrum of compound ***epi*-5b**.

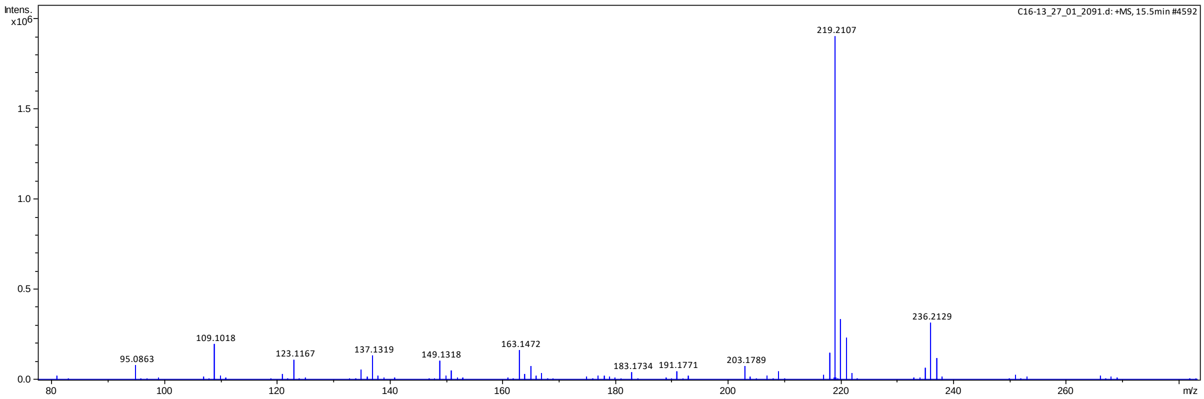

Supplementary Fig. 168. HR-APCI-MS spectrum of compound ***epi*-5b**.

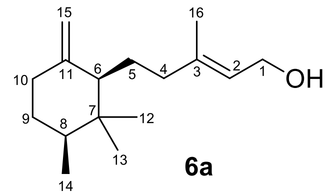

6a

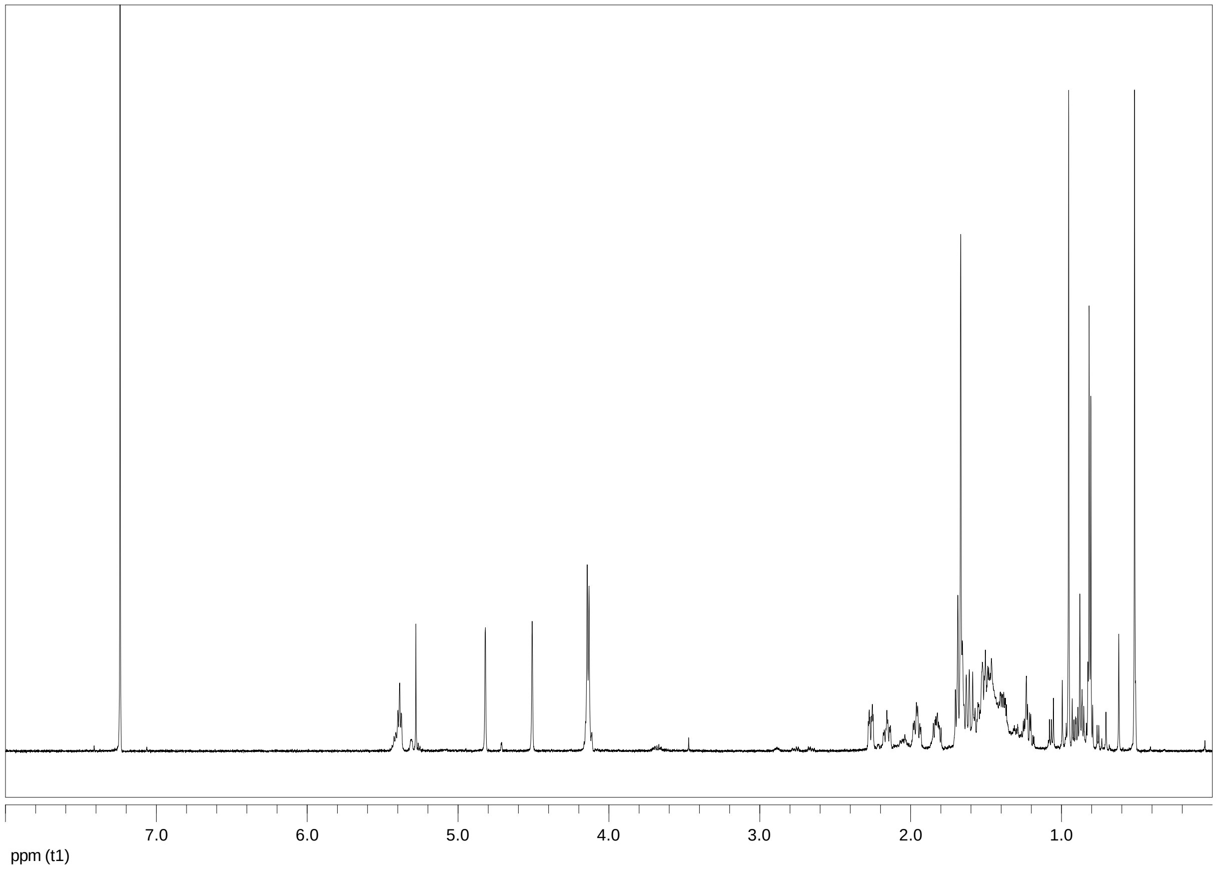

Supplementary Fig. 169. ^{1}H NMR spectrum (in $CDCl_3$) of compound **6a**.

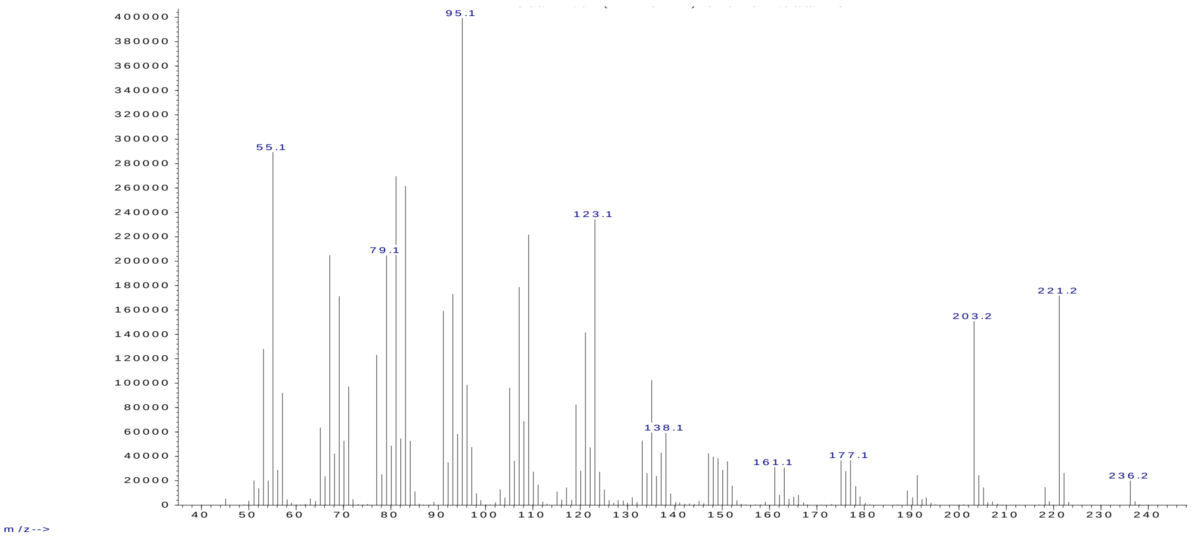

Supplementary Fig. 170. LR-EI-MS spectrum of compound **6a**.

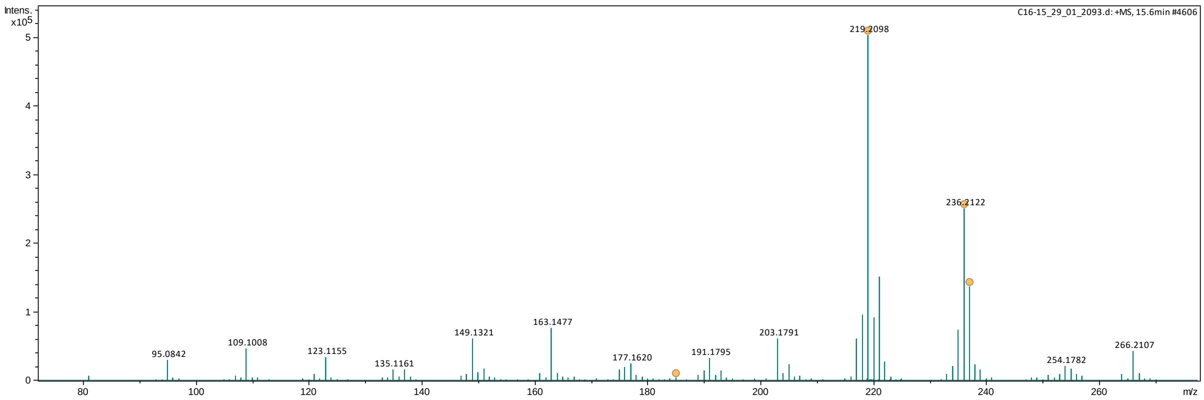

Supplementary Fig. 171. HR-APCI-MS spectrum of compound **6a**.

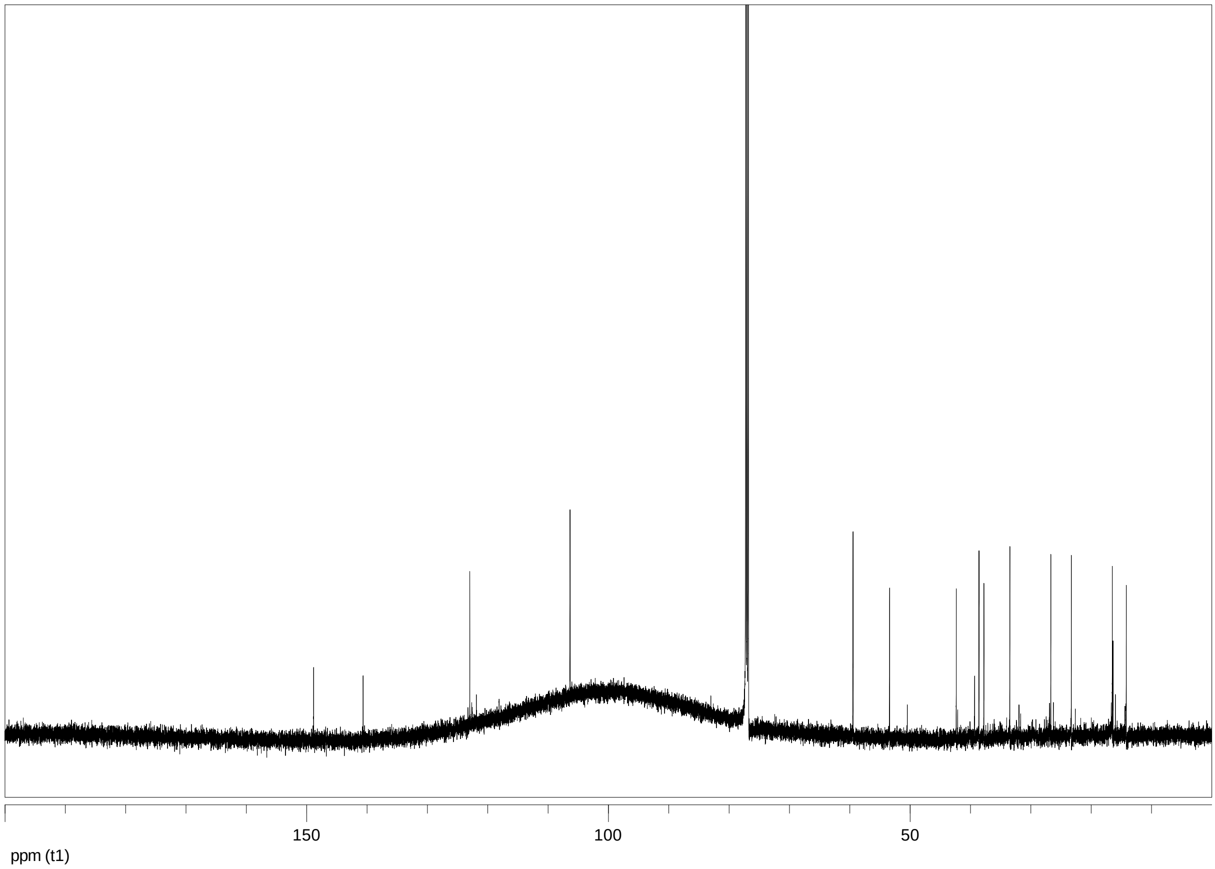

Supplementary Fig. 172. ^{13}C NMR spectrum (in $CDCl_3$) of compound **6a**.

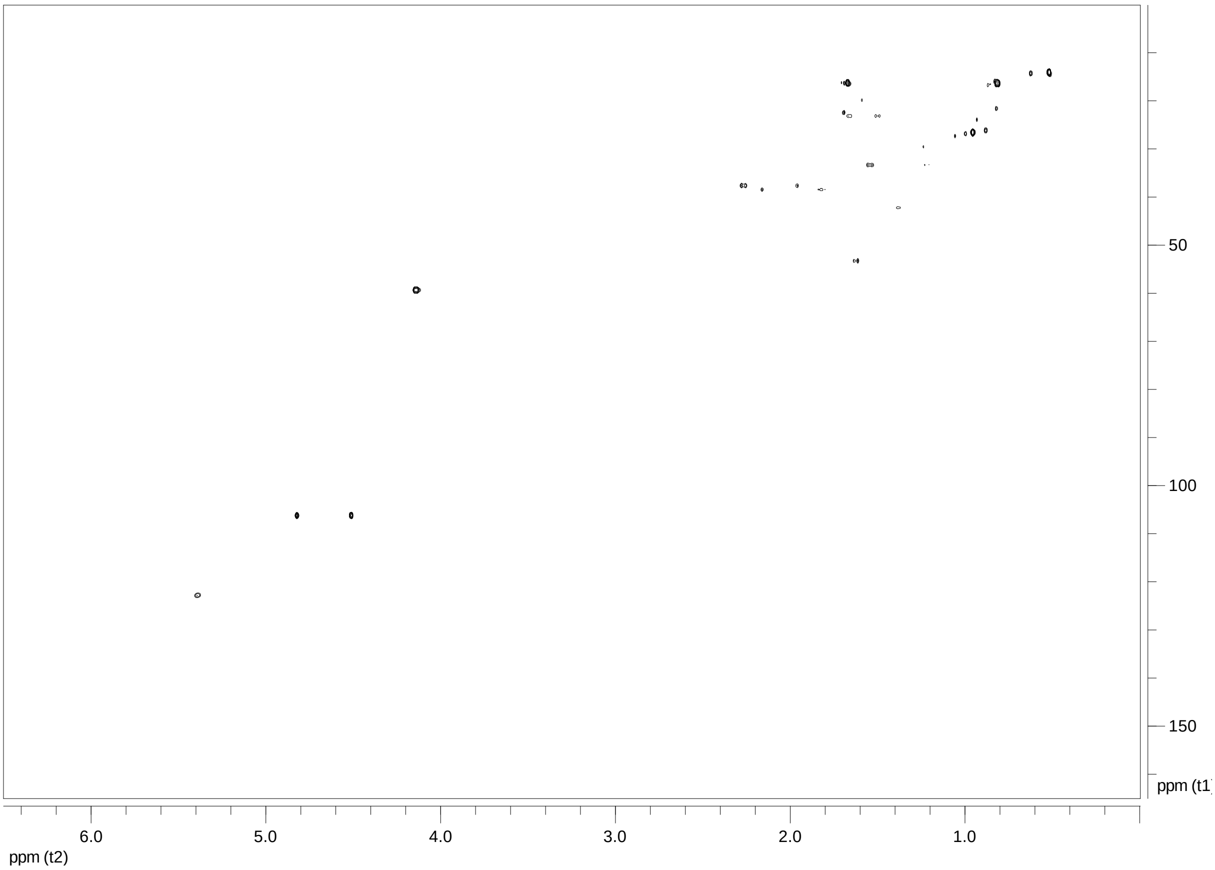

Supplementary Fig. 173. HSQC spectrum (in $CDCl_3$) of compound **6a**.

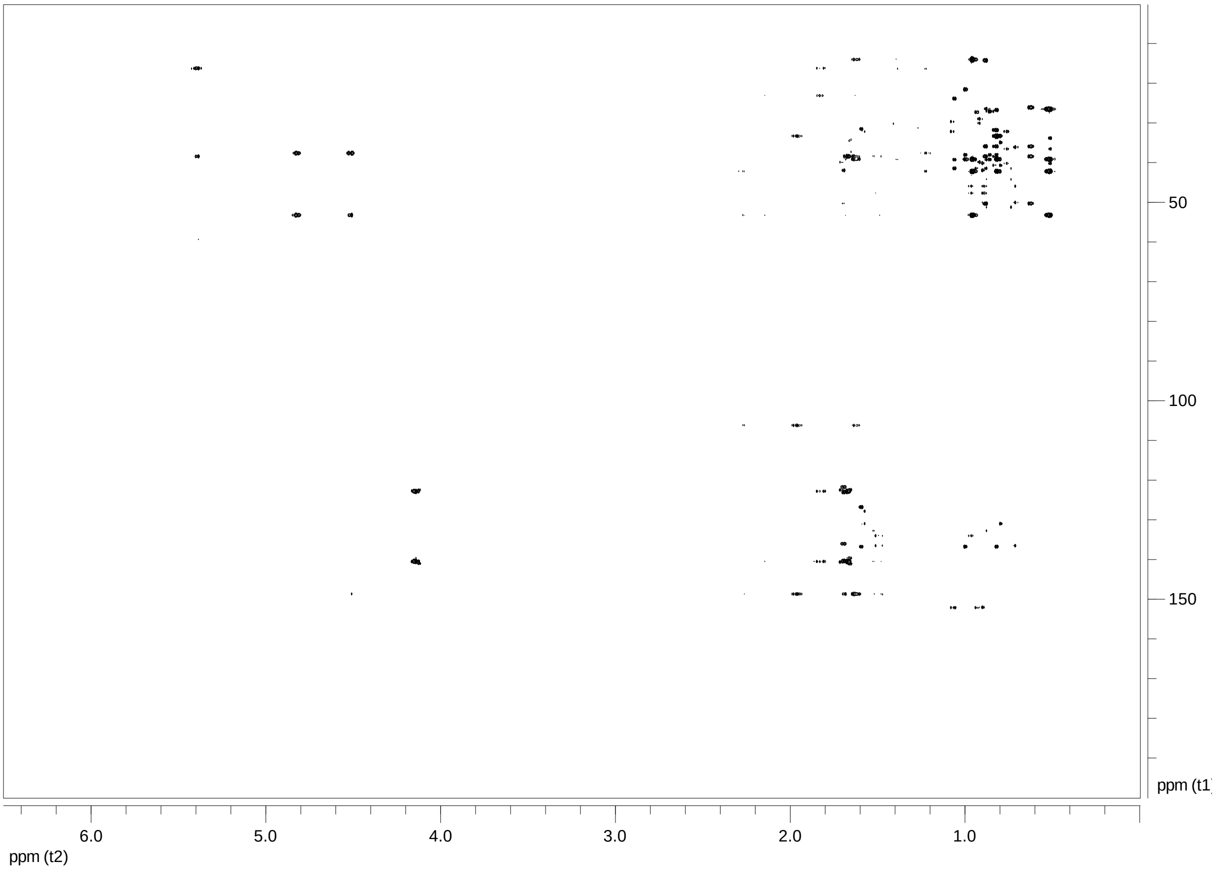

Supplementary Fig. 174. HMBC spectrum (in $CDCl_3$) of compound **6a**.

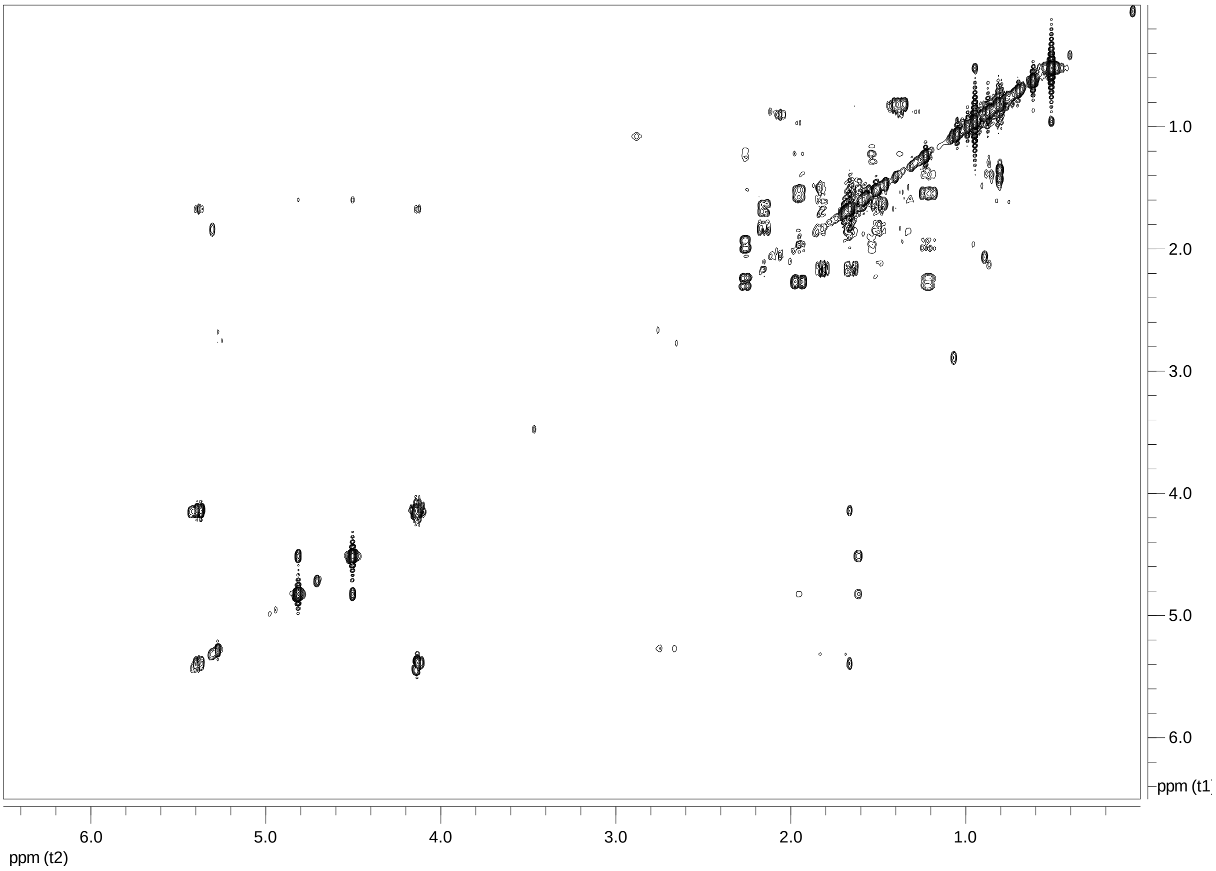

Supplementary Fig. 175. COSY spectrum (in $CDCl_3$) of compound **6a**.

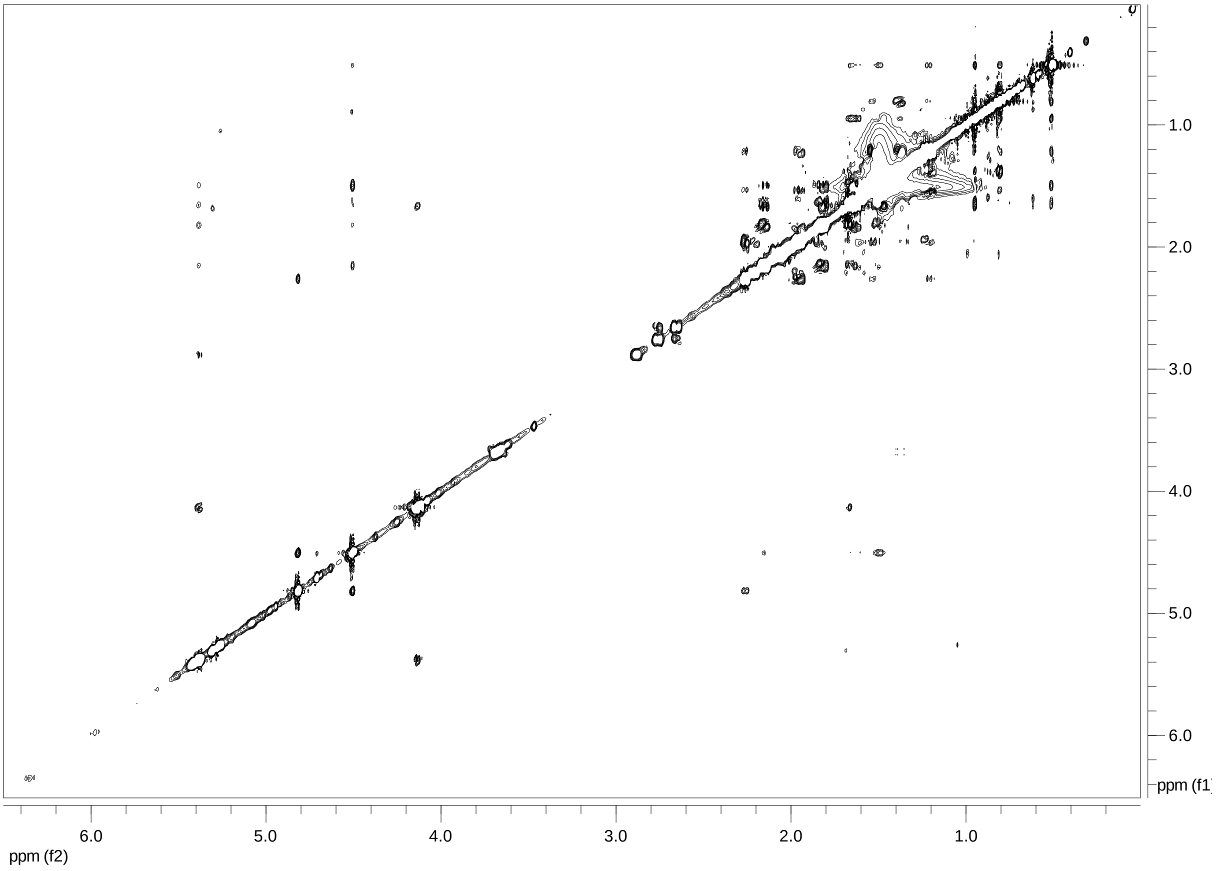

Supplementary Fig. 176. NOESY spectrum (in $CDCl_3$) of compound **6a**.

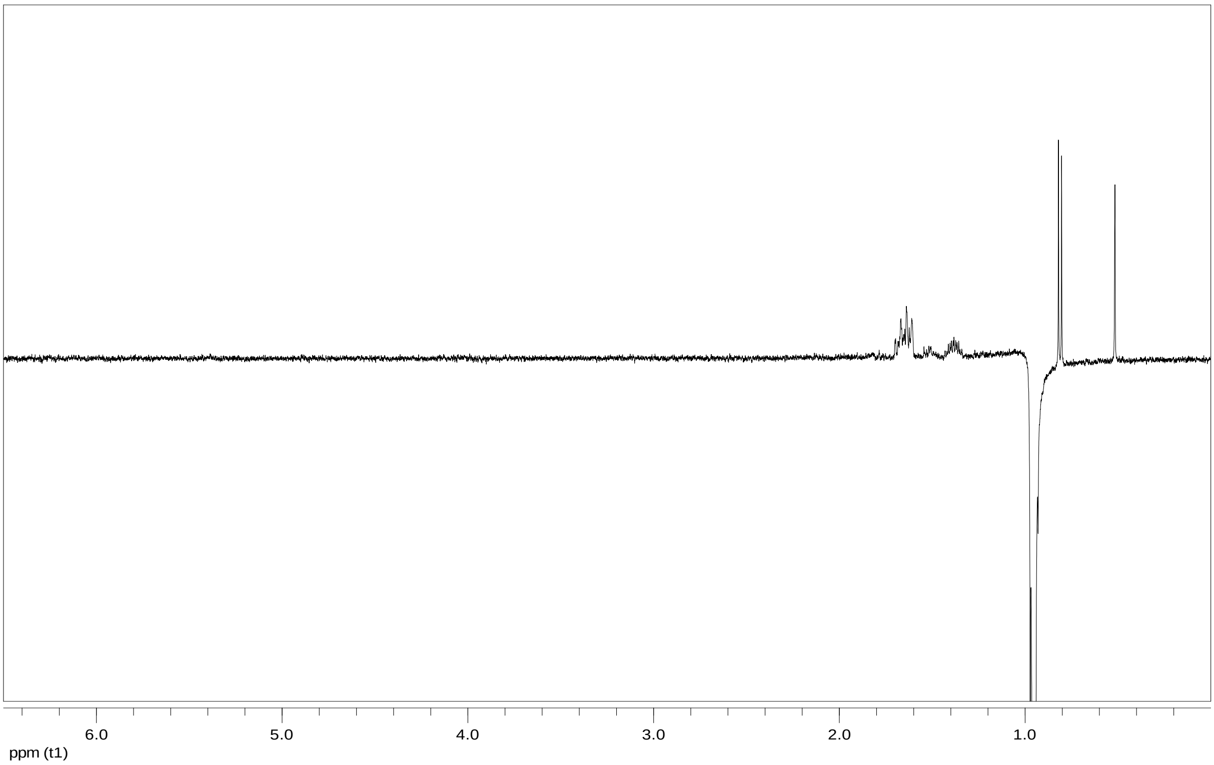

Supplementary Fig. 177. 1D NOE difference spectrum (in $CDCl_3$) of compound **6a** upon irradiation of H_3-12.

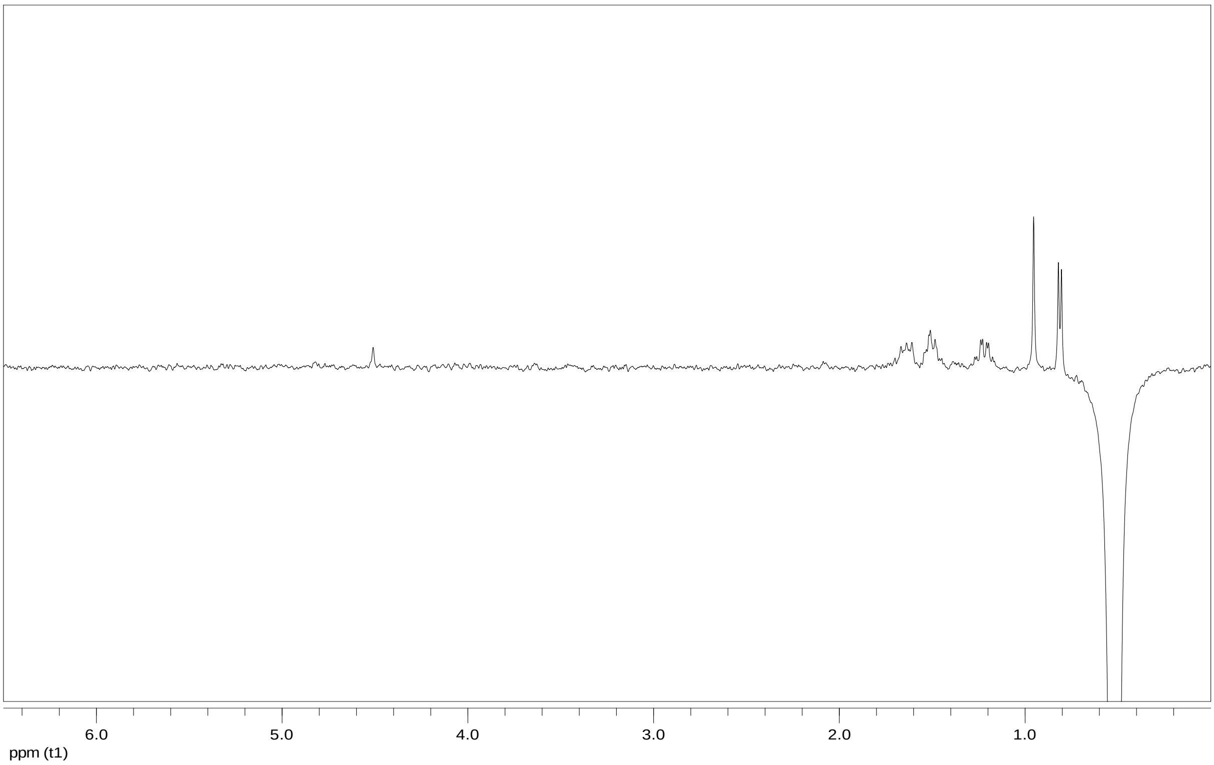

Supplementary Fig. 178. 1D NOE difference spectrum (in $CDCl_3$) of compound **6a** upon irradiation of H_3-13.

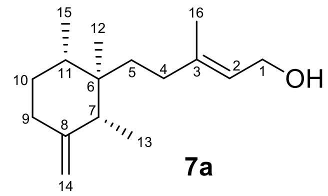

7a

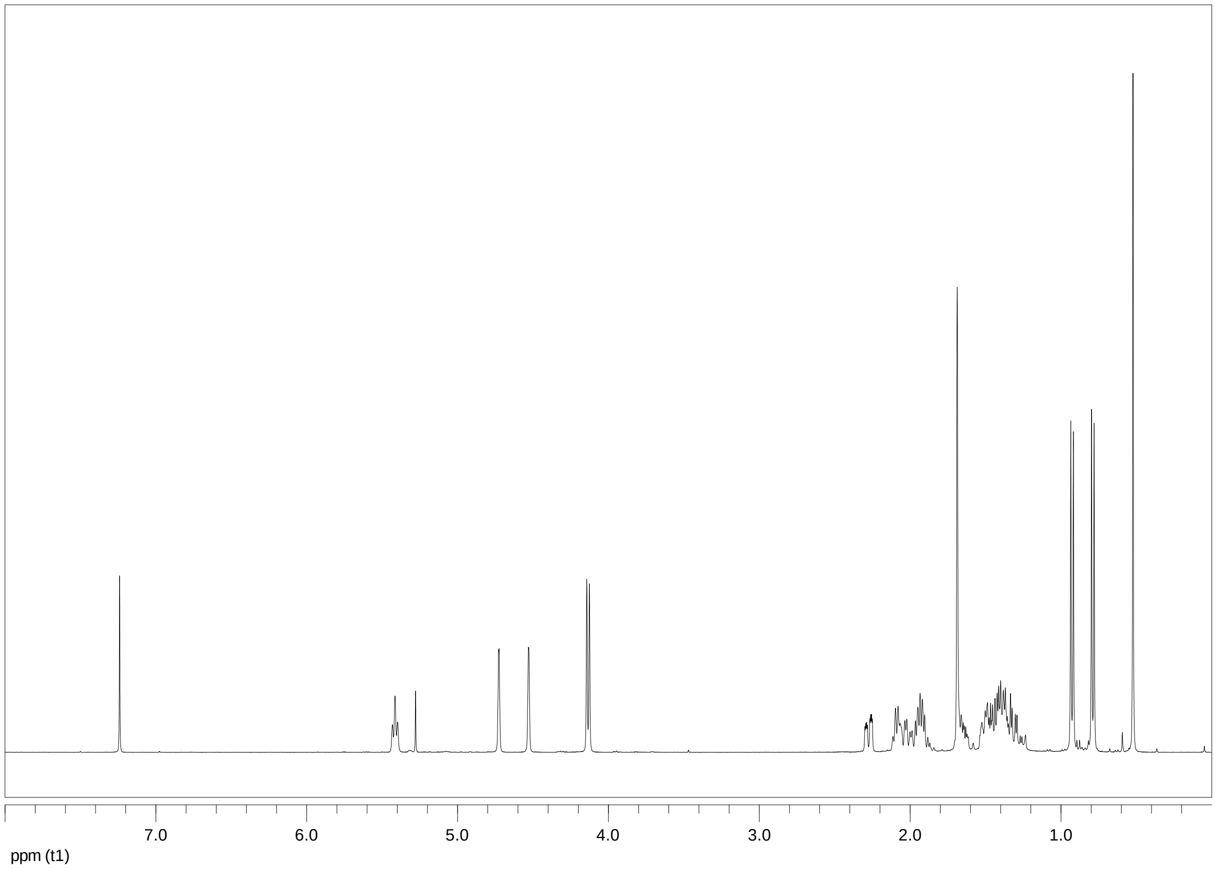

Supplementary Fig. 179. ^{1}H NMR spectrum (in $CDCl_3$) of compound **7a**.

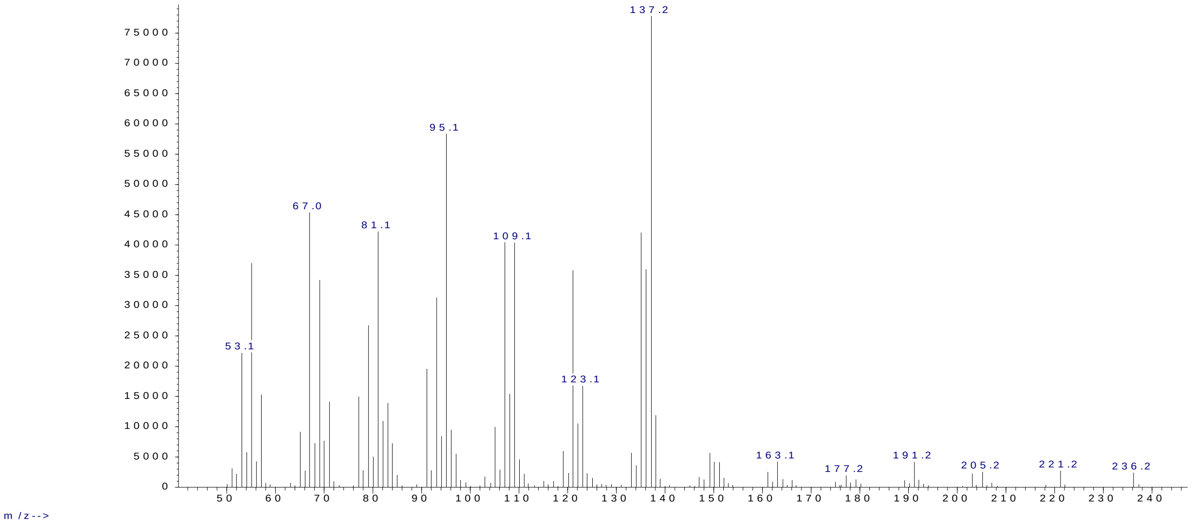

Supplementary Fig. 180. LR-EI-MS spectrum of compound **7a**.

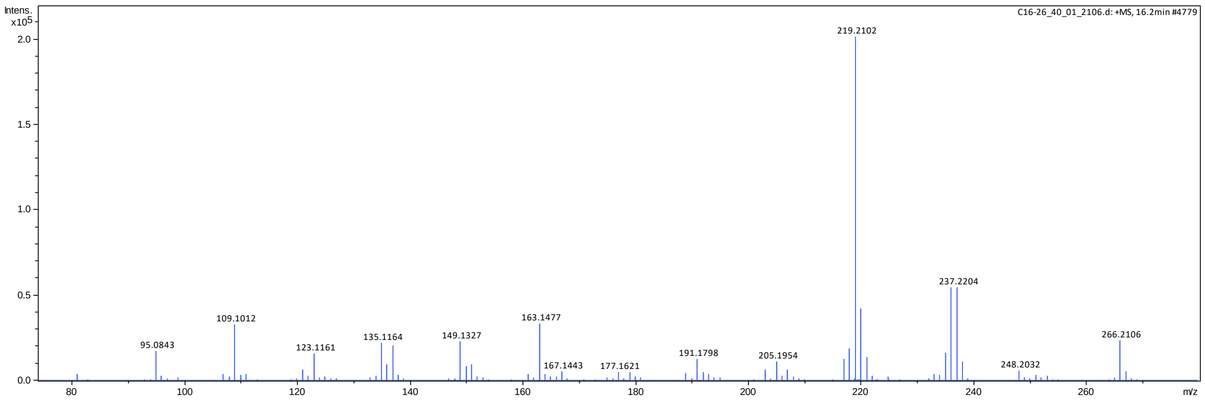

Supplementary Fig. 181. HR-APCI-MS spectrum of compound **7a**.

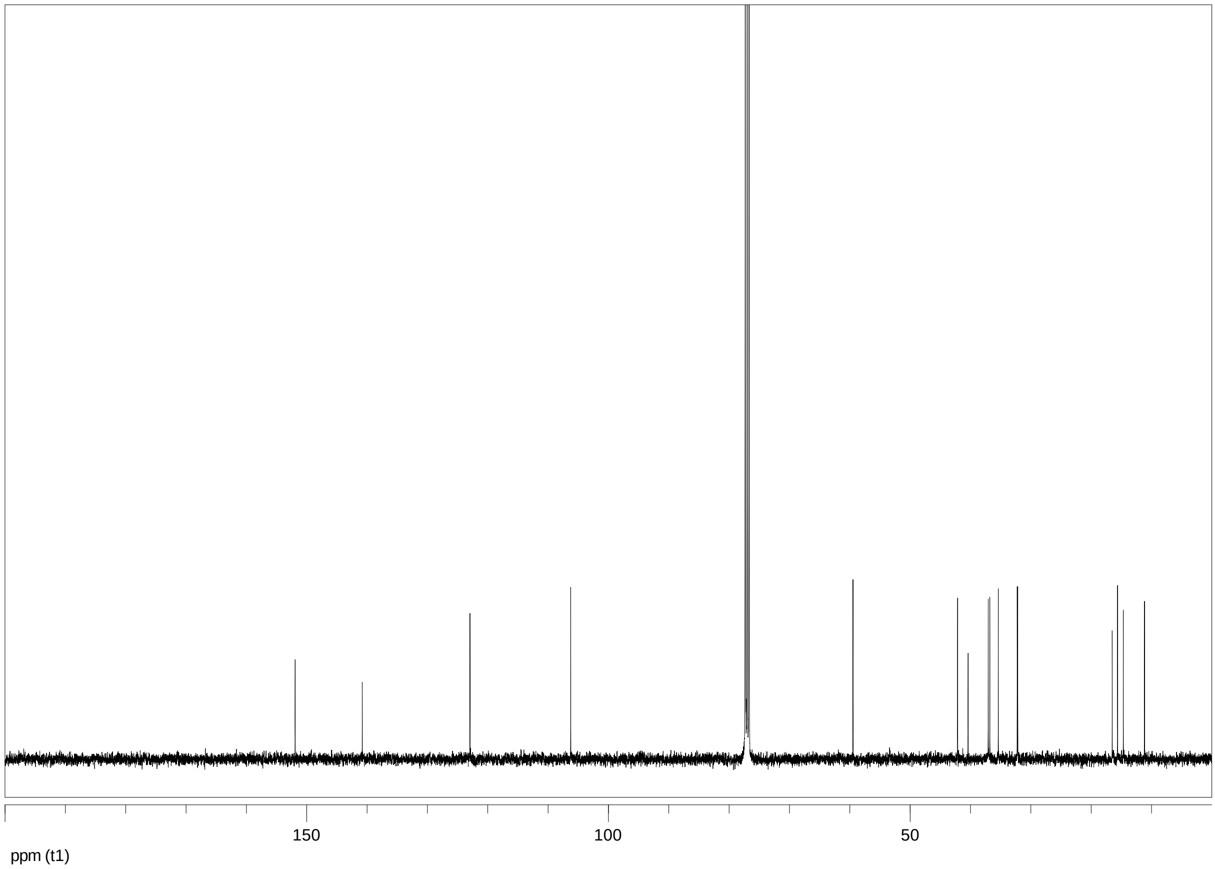

Supplementary Fig. 182. ^{13}C NMR spectrum (in $CDCl_3$) of compound **7a**.

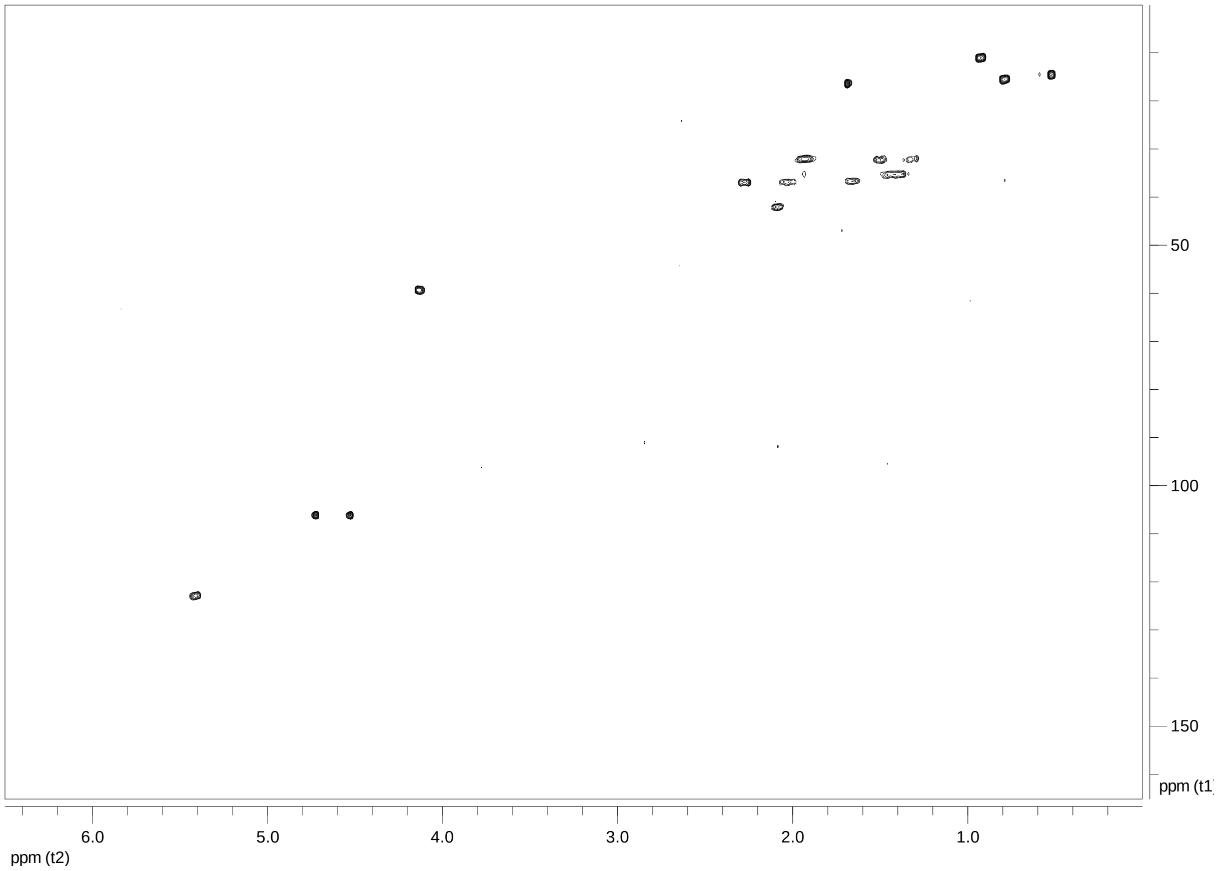

Supplementary Fig. 183. HSQC spectrum (in $CDCl_3$) of compound **7a**.

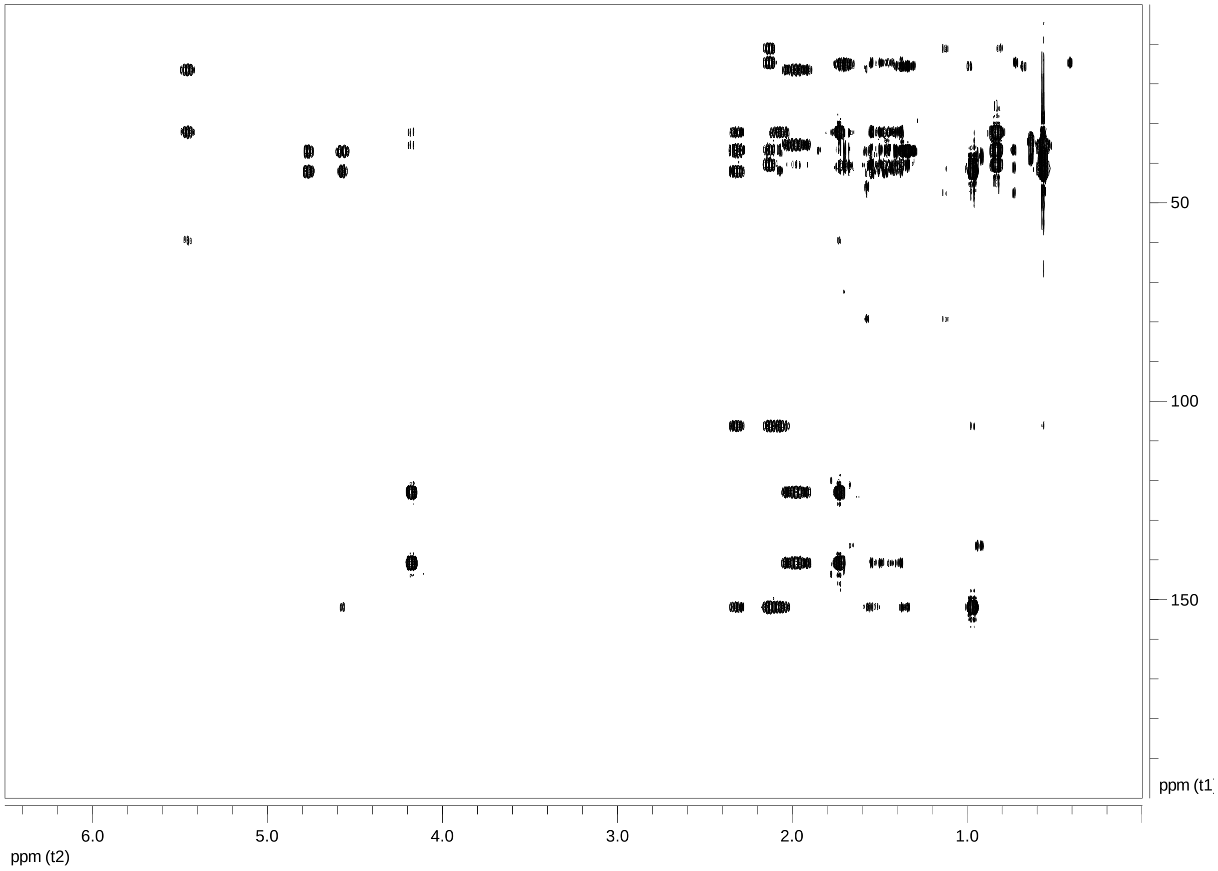

Supplementary Fig. 184. HMBC spectrum (in $CDCl_3$) of compound **7a**.

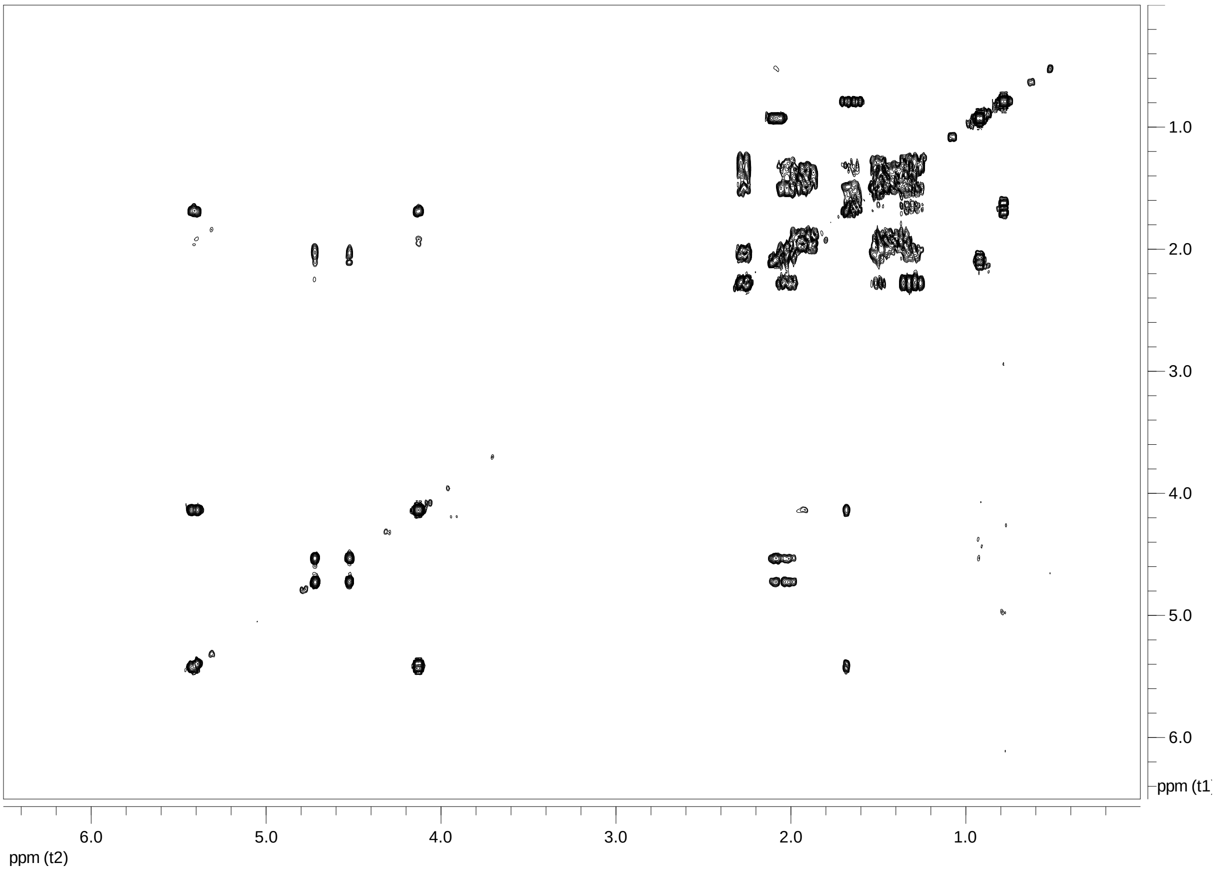

Supplementary Fig. 185. COSY spectrum (in $CDCl_3$) of compound **7a**.

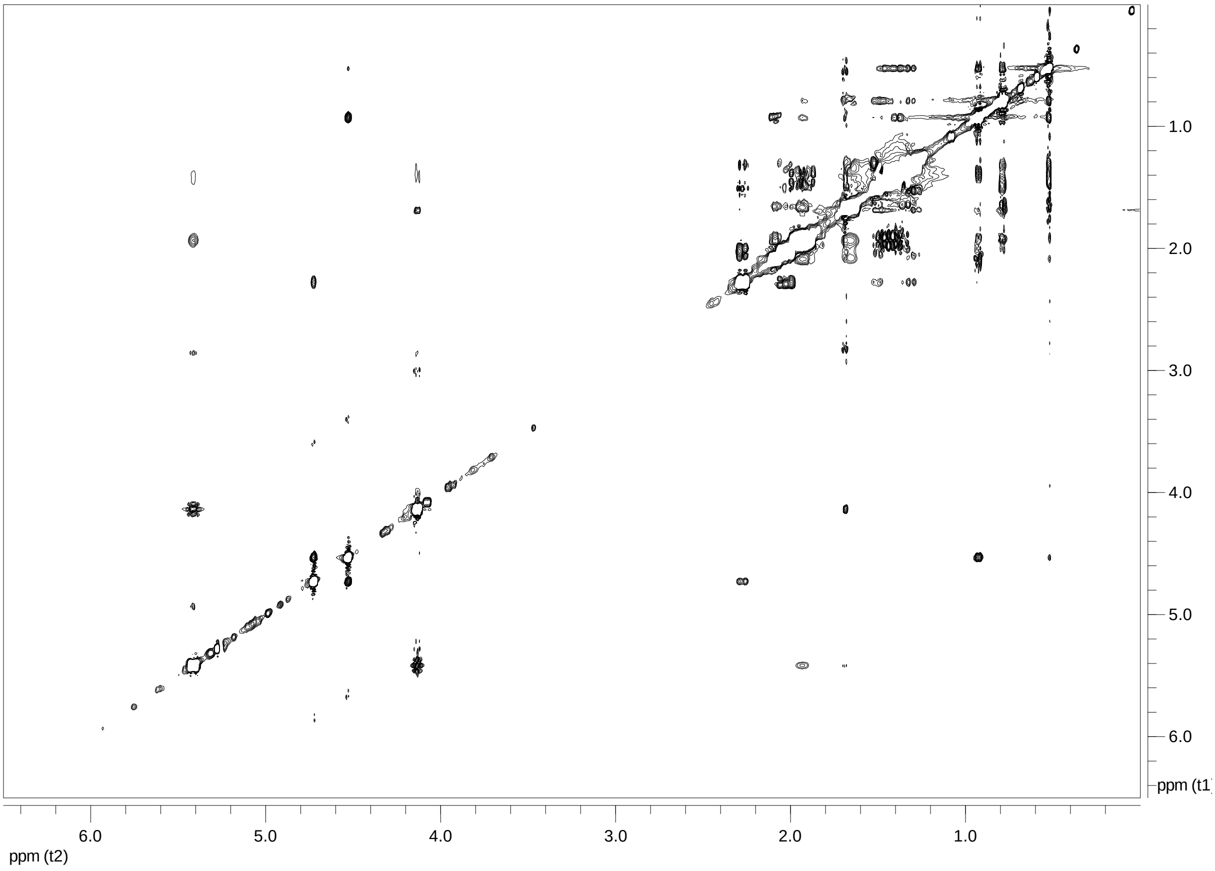

Supplementary Fig. 186. NOESY spectrum (in $CDCl_3$) of compound **7a**.

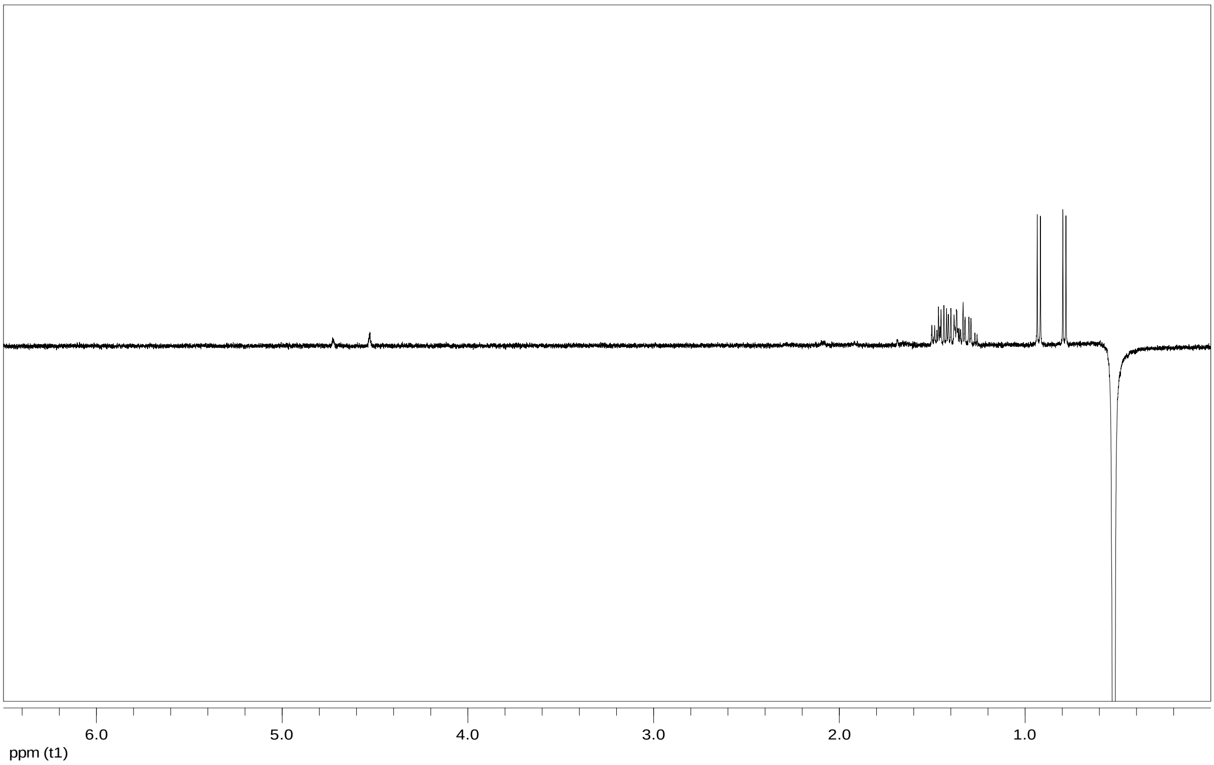

Supplementary Fig. 187. 1D NOE difference spectrum (in $CDCl_3$) of compound **7a** upon irradiation of H_3-12.

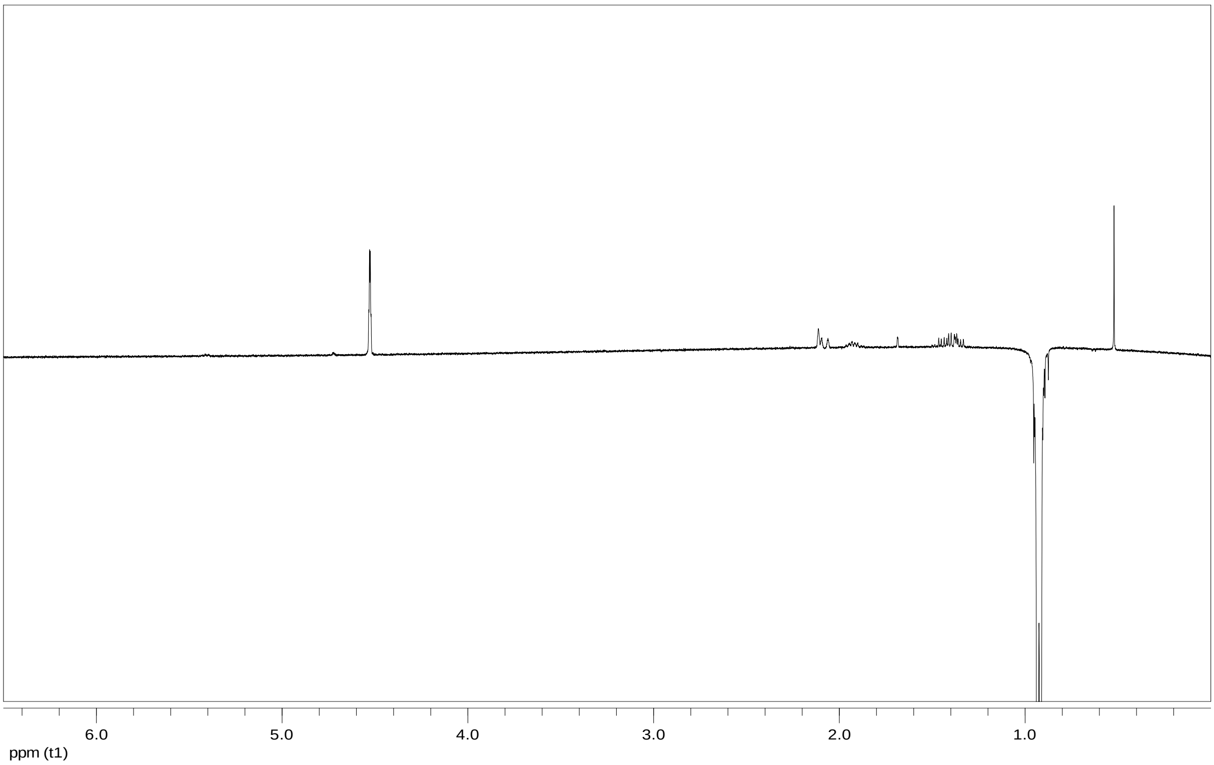

Supplementary Fig. 188. 1D NOE difference spectrum (in $CDCl_3$) of compound **7a** upon irradiation of H_3-13.

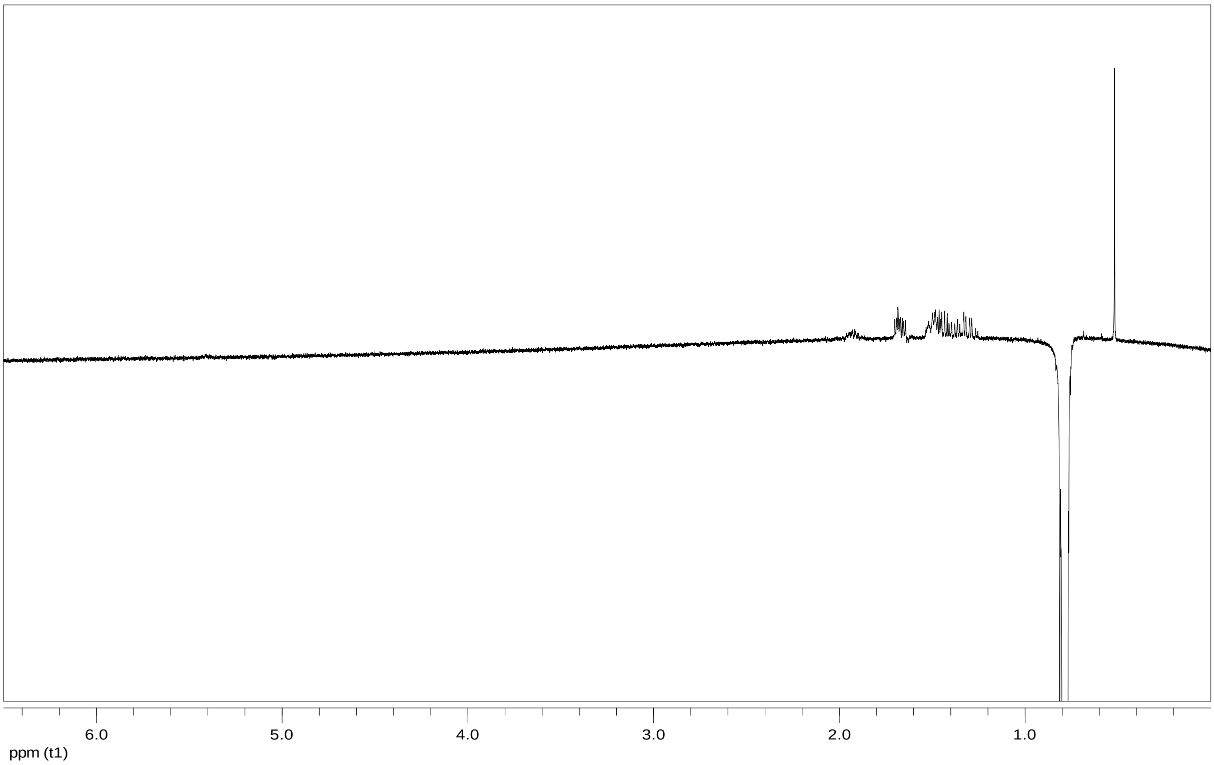

Supplementary Fig. 189. 1D NOE difference spectrum (in $CDCl_3$) of compound **7a** upon irradiation of H_3-15.

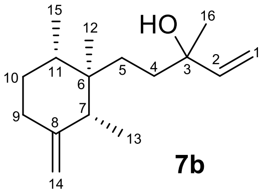

7b

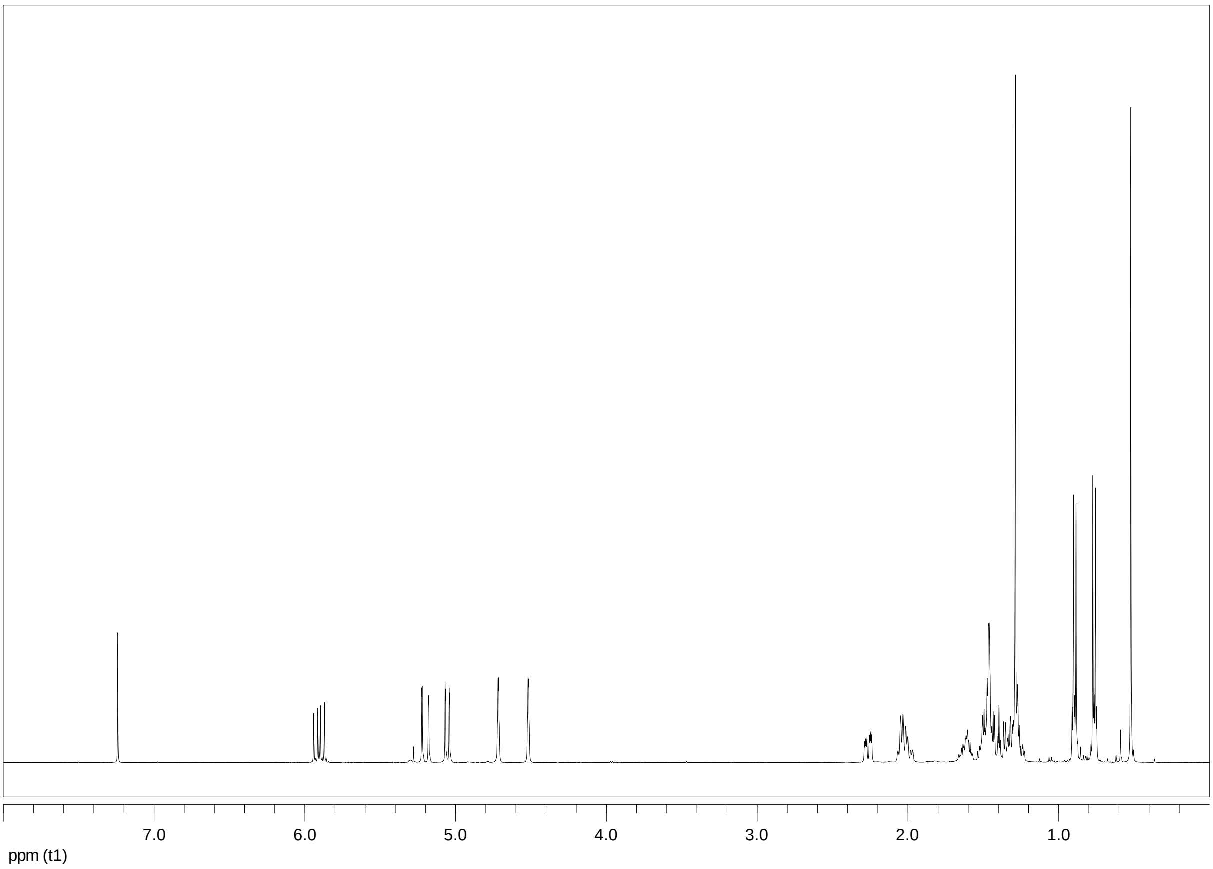

Supplementary Fig. 190. ^{1}H NMR spectrum (in $CDCl_3$) of compound **7b**.

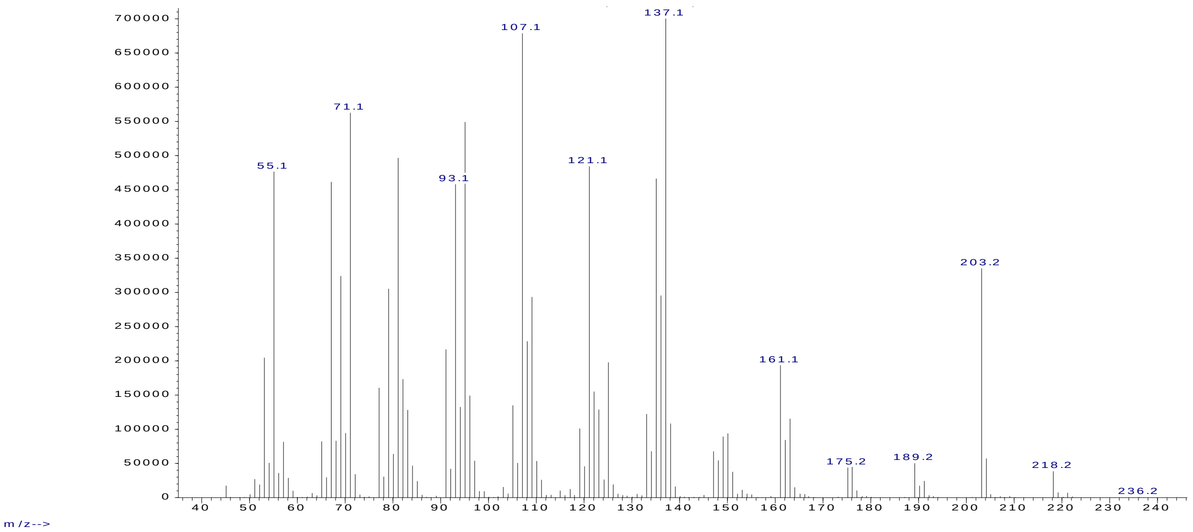

Supplementary Fig. 191. LR-EI-MS spectrum of compound **7b**.

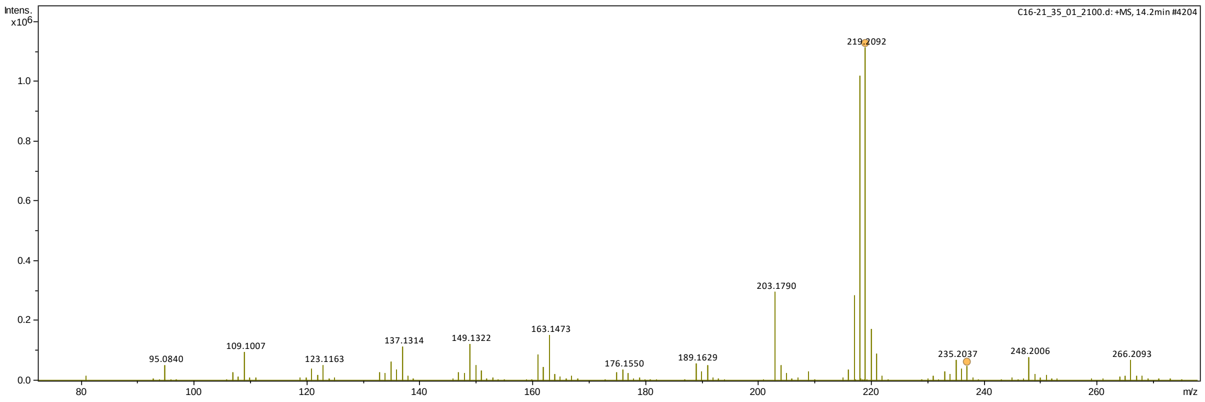

Supplementary Fig. 192. HR-APCI-MS spectrum of compound **7b**.

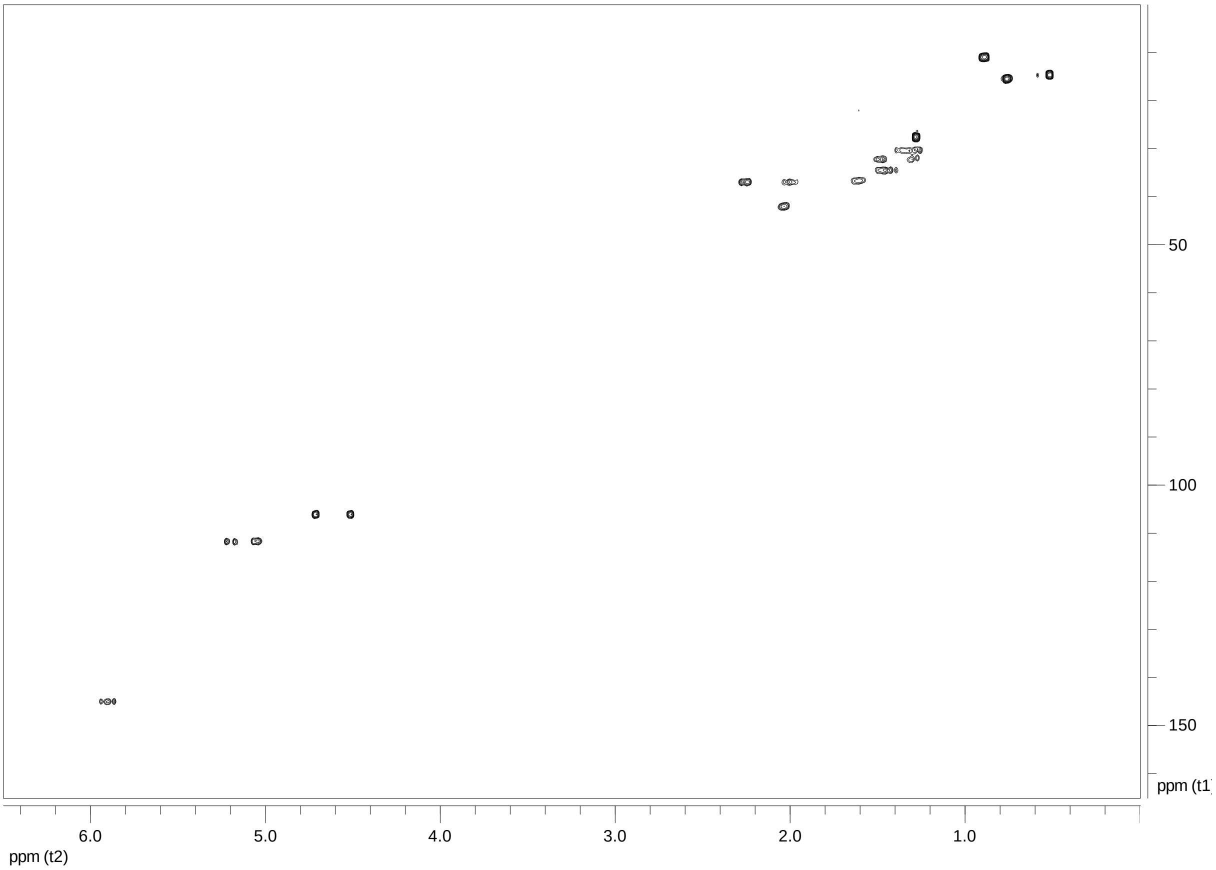

Supplementary Fig. 193. HSQC spectrum (in $CDCl_3$) of compound **7b**.

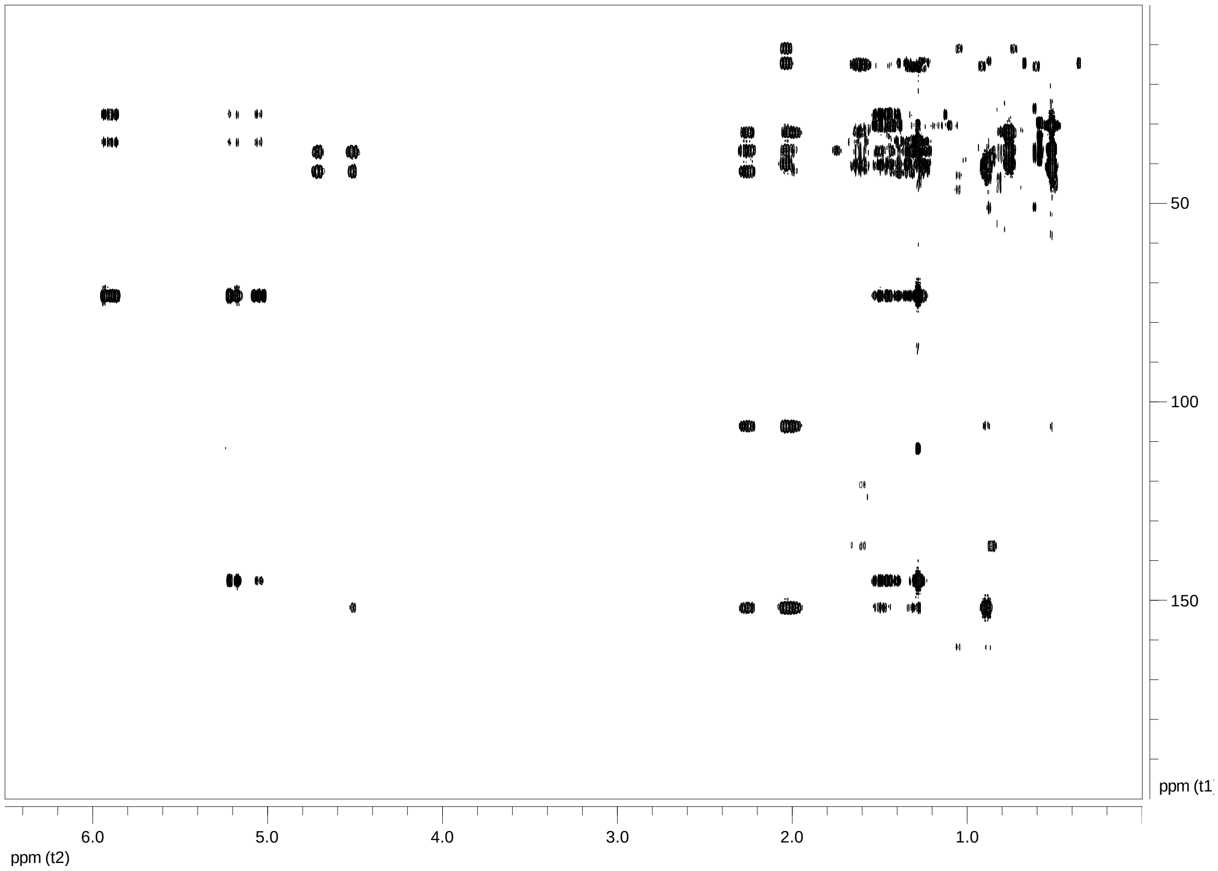

Supplementary Fig. 194. HMBC spectrum (in $CDCl_3$) of compound **7b**.

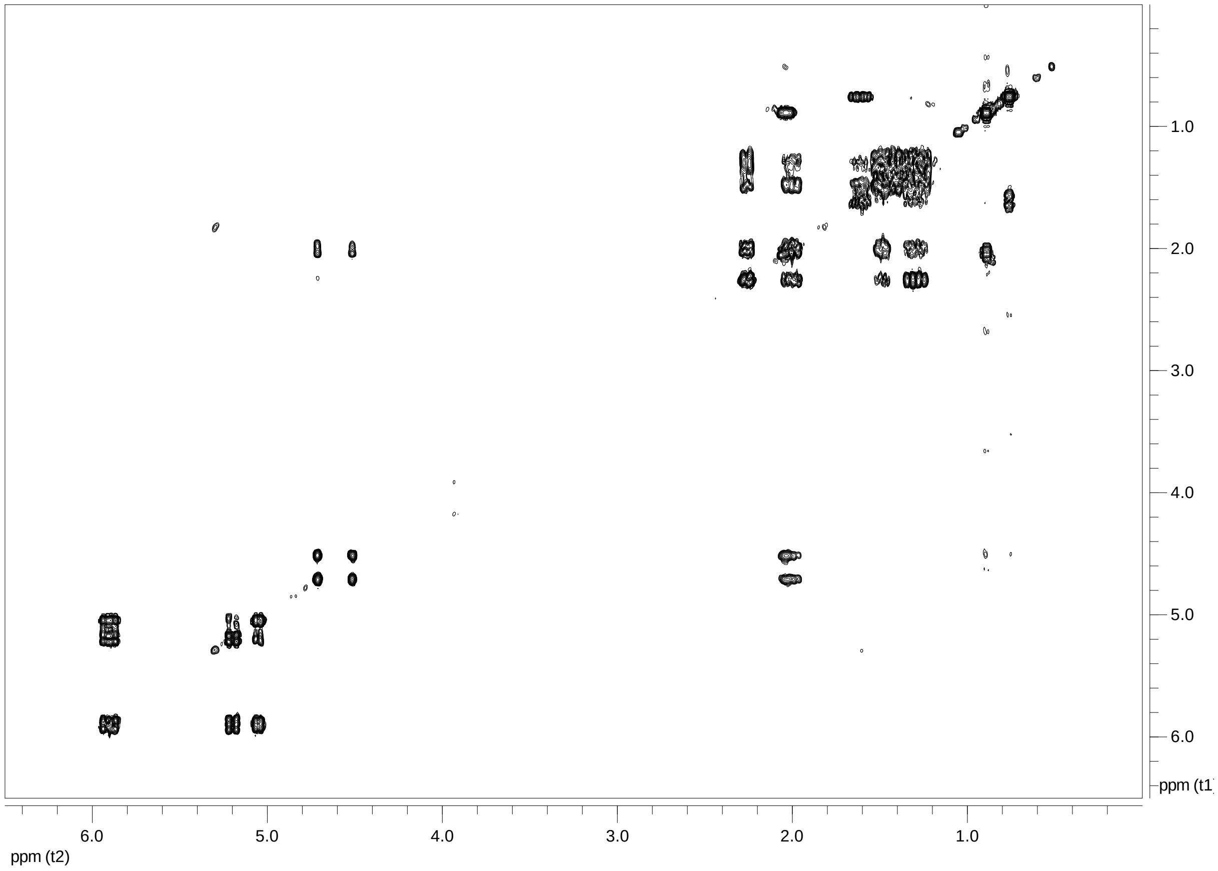

Supplementary Fig. 195. COSY spectrum (in $CDCl_3$) of compound **7b**.

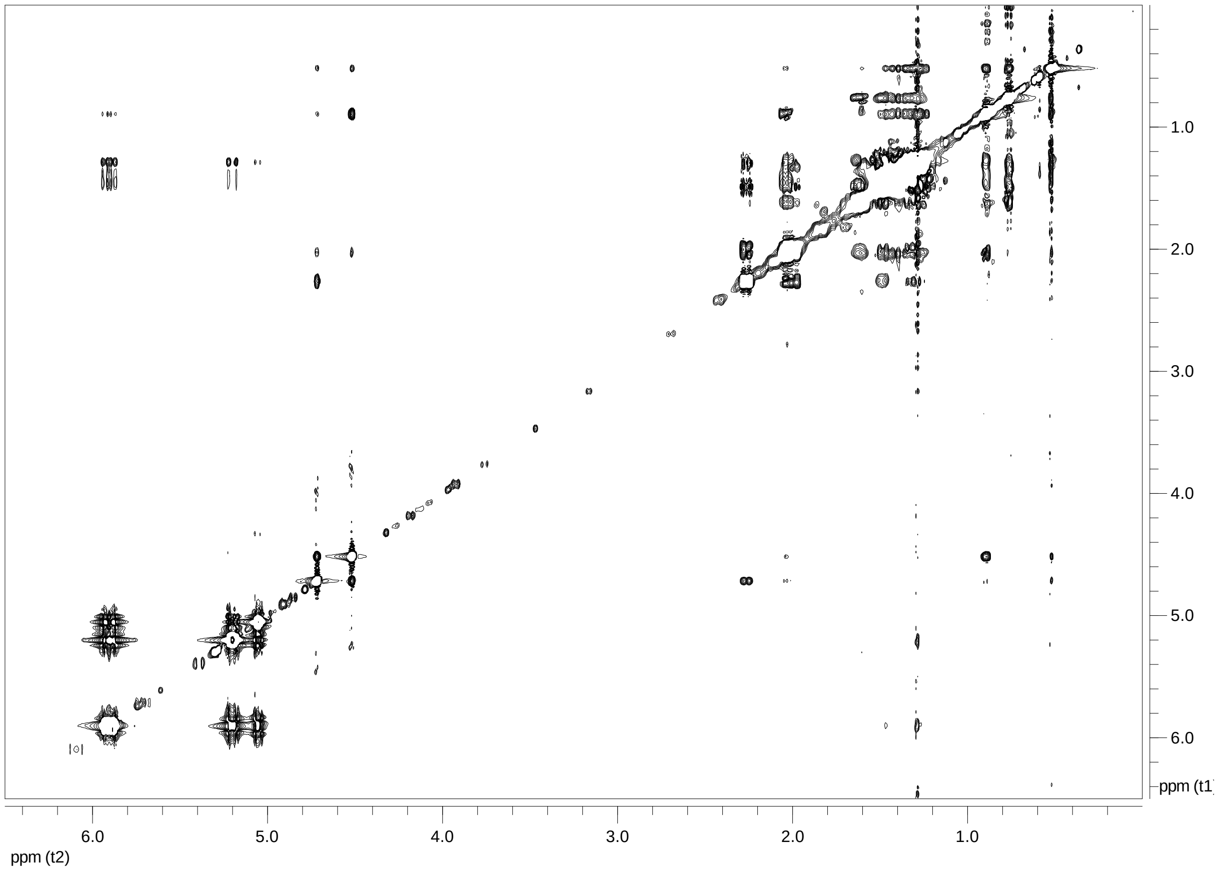

Supplementary Fig. 196. NOESY spectrum (in $CDCl_3$) of compound **7b**.

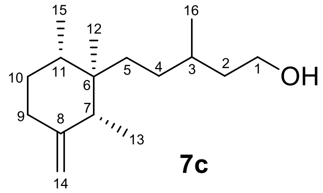

7c

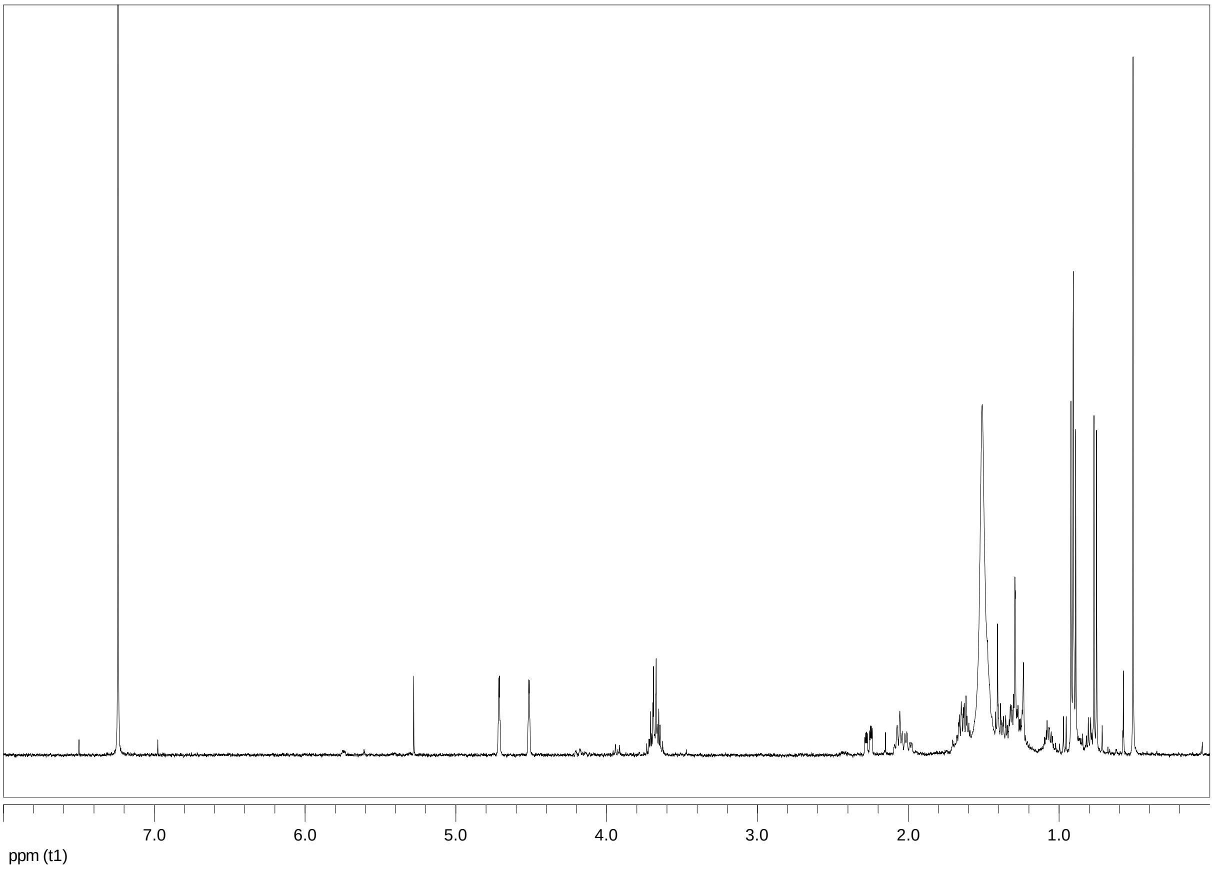

Supplementary Fig. 197. ^{1}H NMR spectrum (in $CDCl_3$) of compound **7c**.

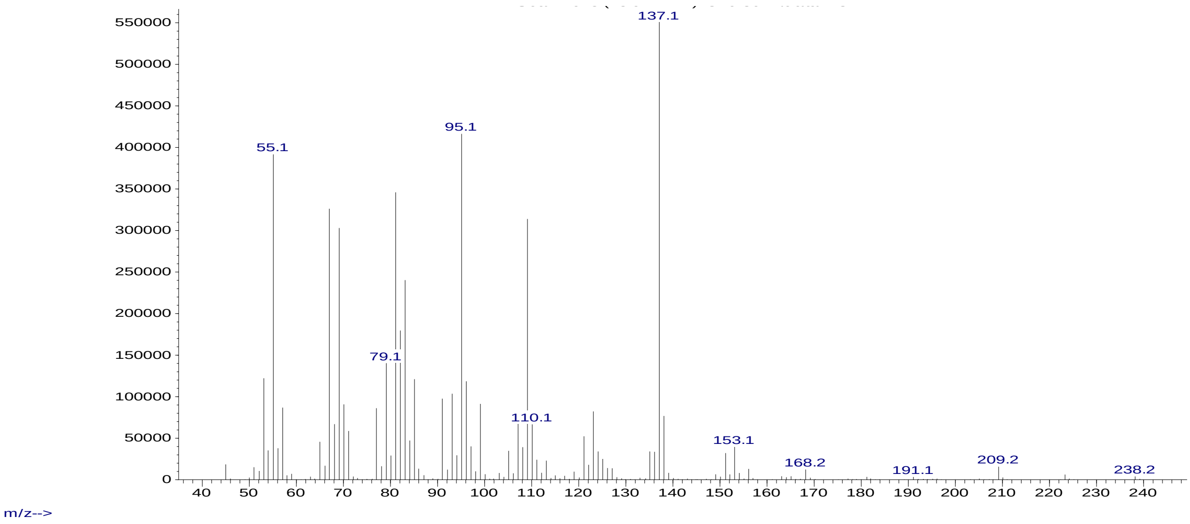

Supplementary Fig. 198. LR-EI-MS spectrum of compound **7c**.

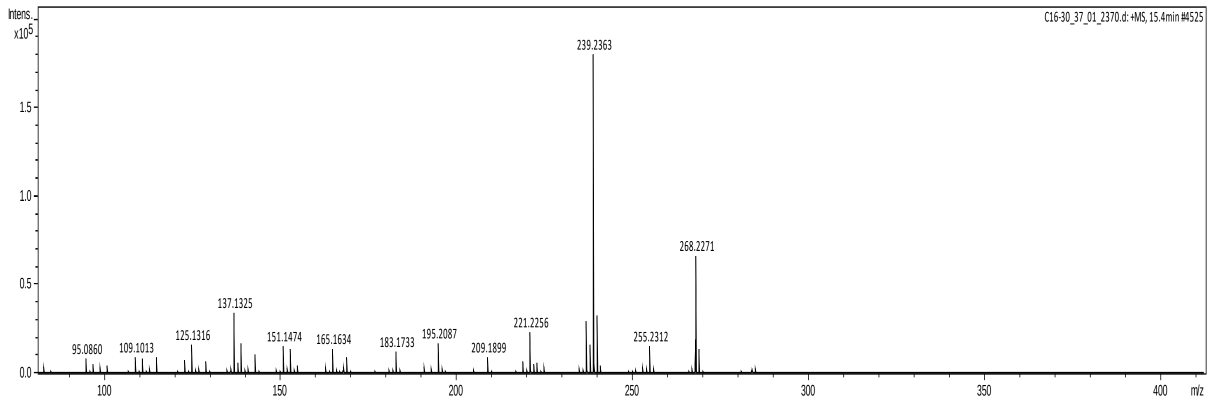

Supplementary Fig. 199. HR-APCI-MS spectrum of compound **7c**.

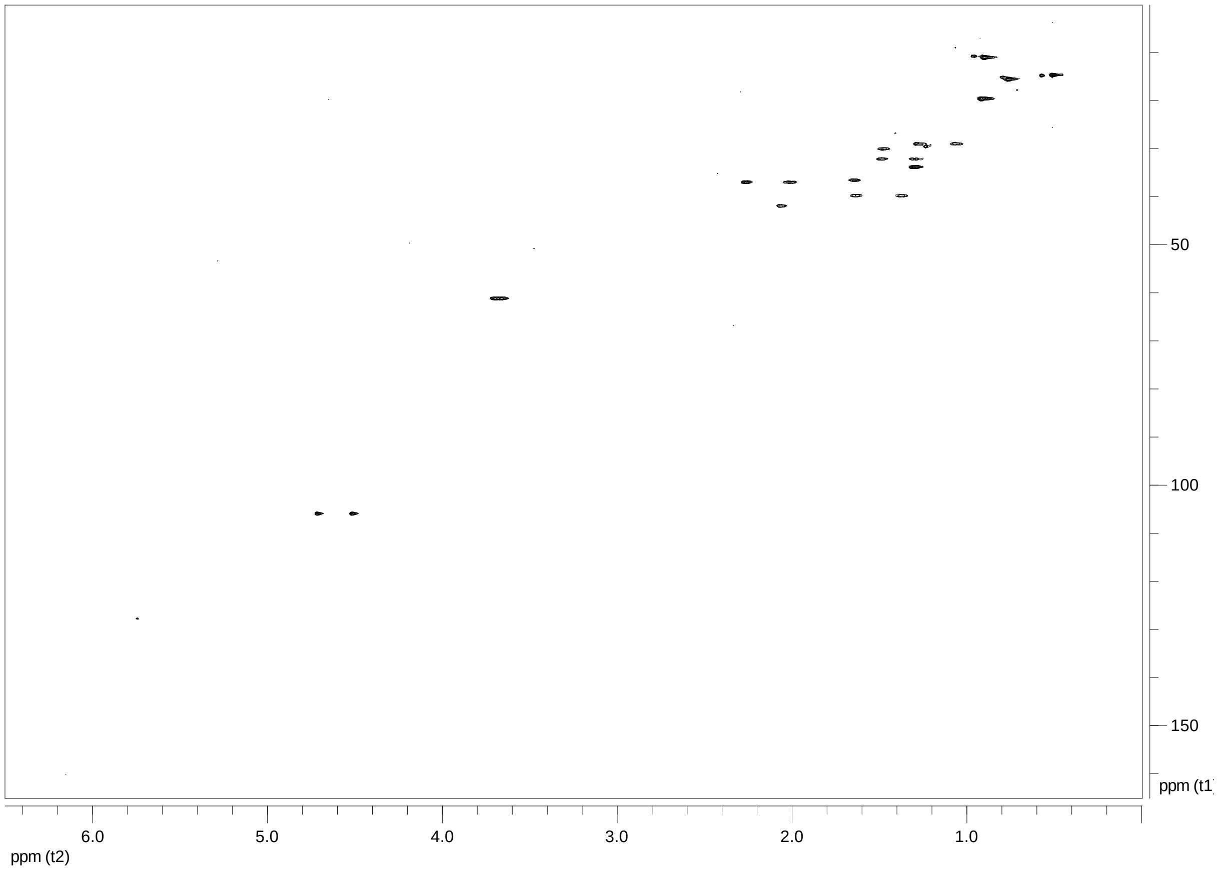

Supplementary Fig. 200. HSQC spectrum (in $CDCl_3$) of compound **7c**.

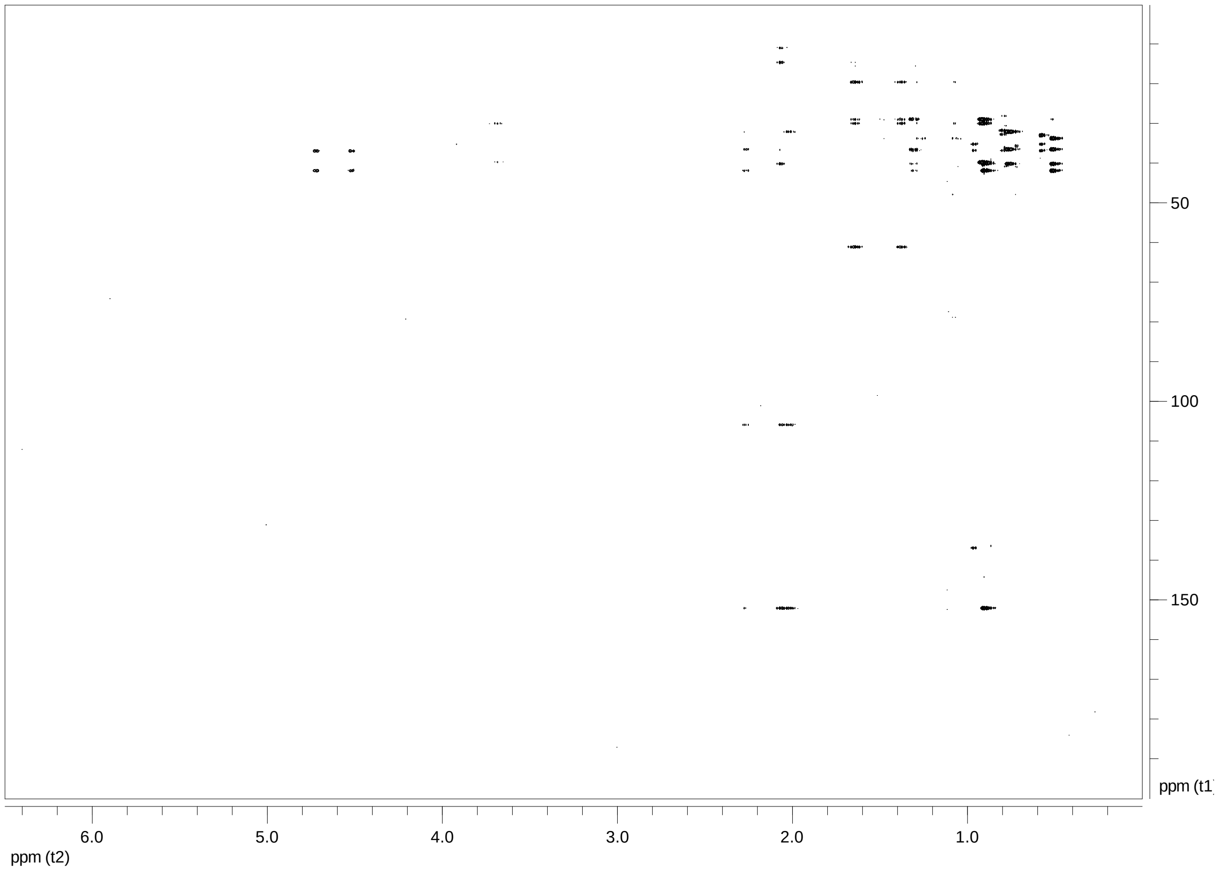

Supplementary Fig. 201. HMBC spectrum (in $CDCl_3$) of compound **7c**.

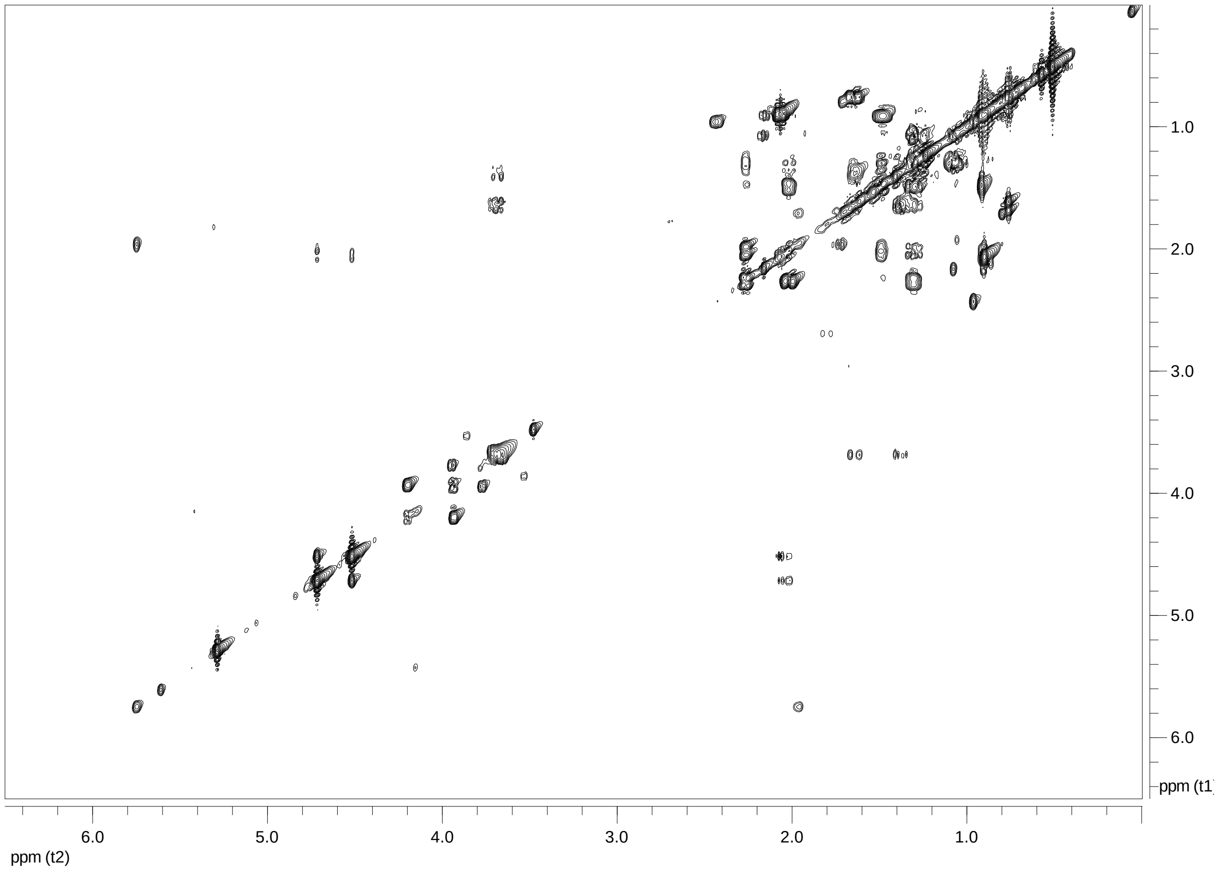

Supplementary Fig. 202. COSY spectrum (in $CDCl_3$) of compound **7c**.

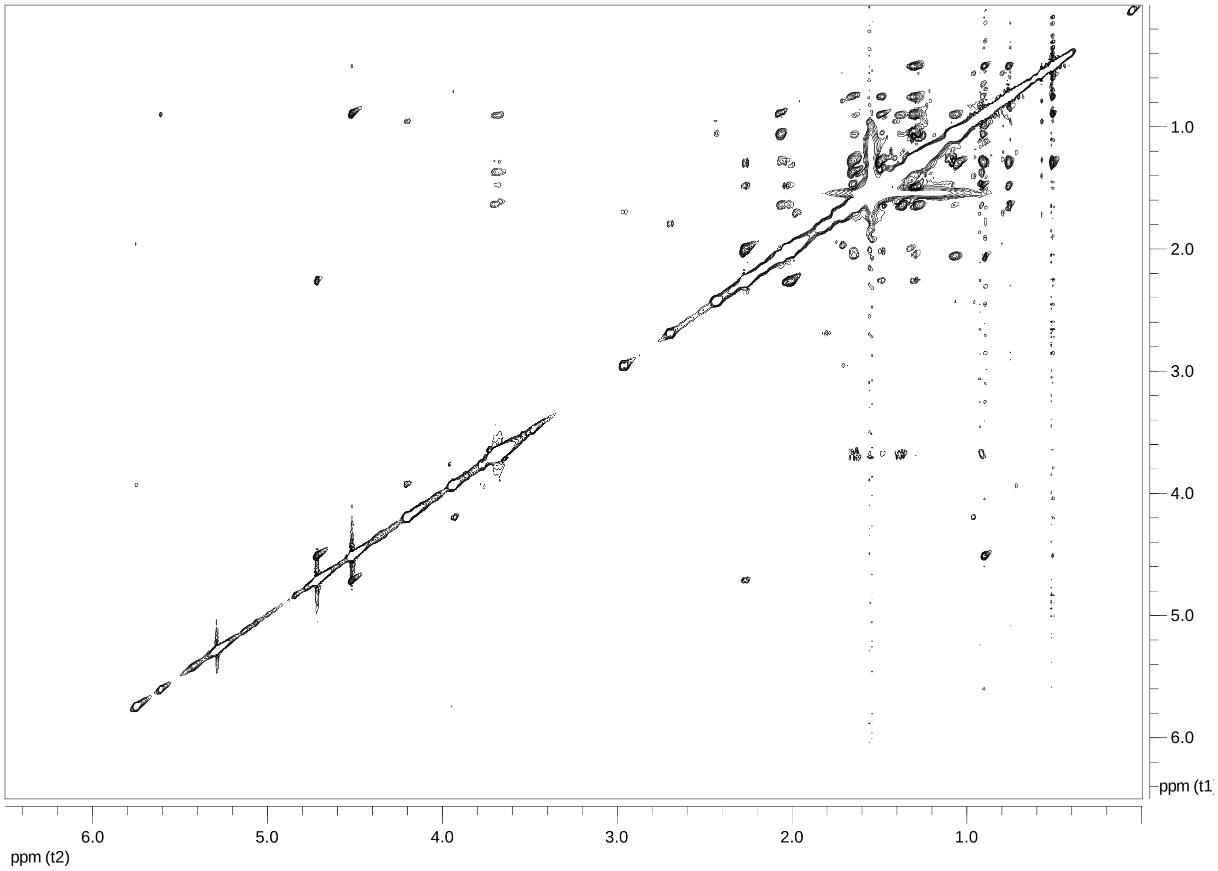

Supplementary Fig. 203. NOESY spectrum (in $CDCl_3$) of compound **7c**.

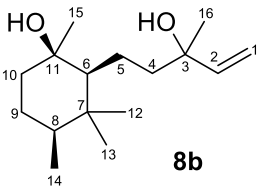

8b

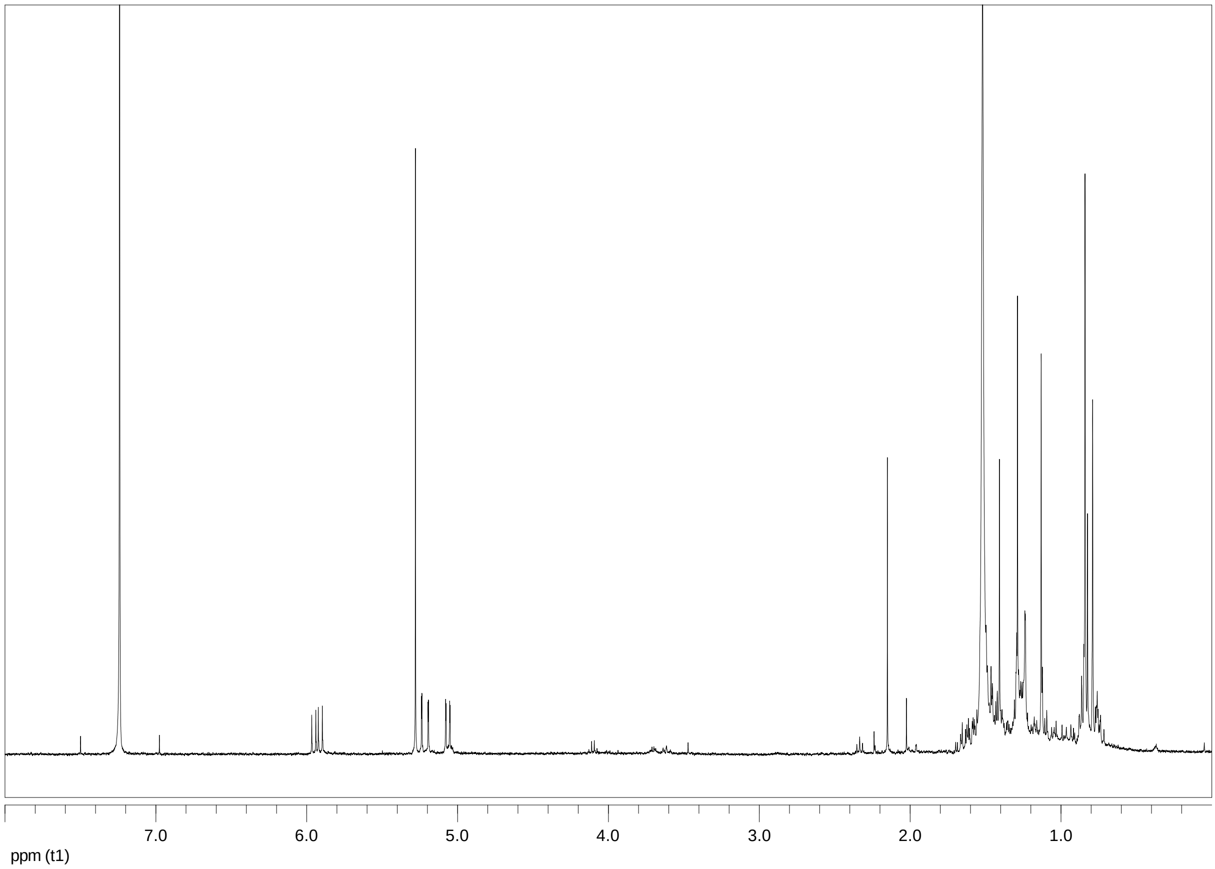

Supplementary Fig. 204. ^{1}H NMR spectrum (in $CDCl_3$) of compound **8b**.

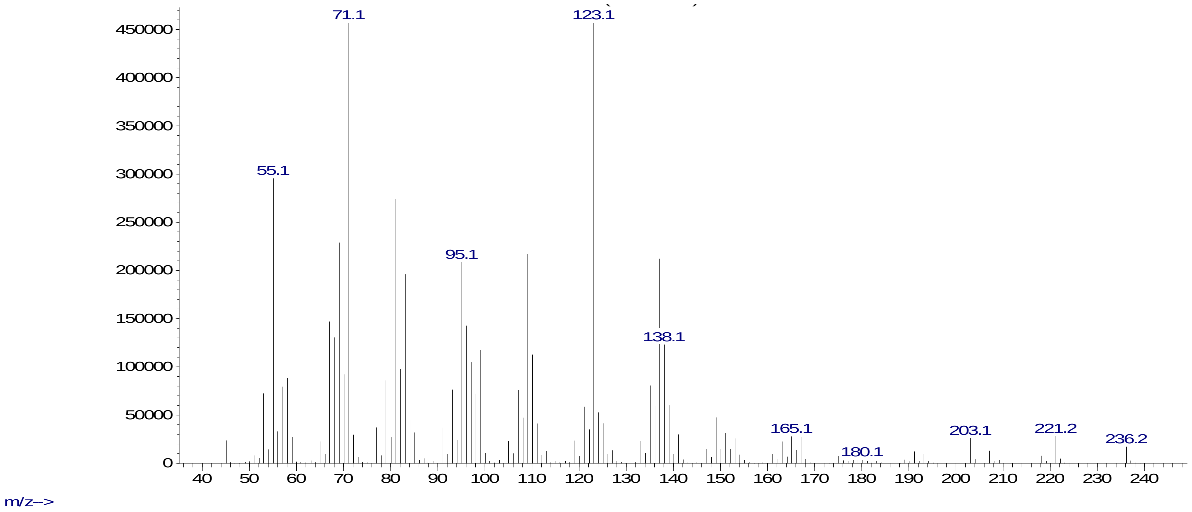

Supplementary Fig. 205. LR-EI-MS spectrum of compound **8b**.

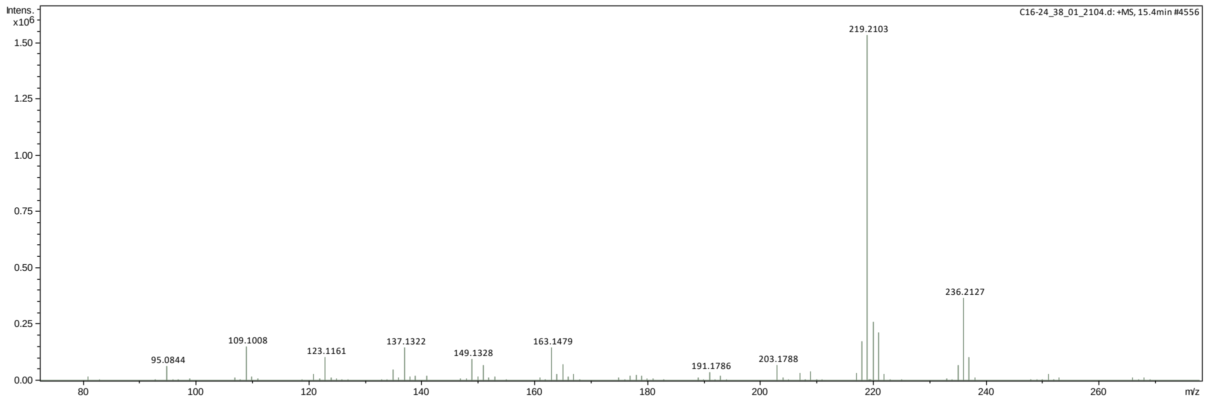

Supplementary Fig. 206. HR-APCI-MS spectrum of compound **8b**.

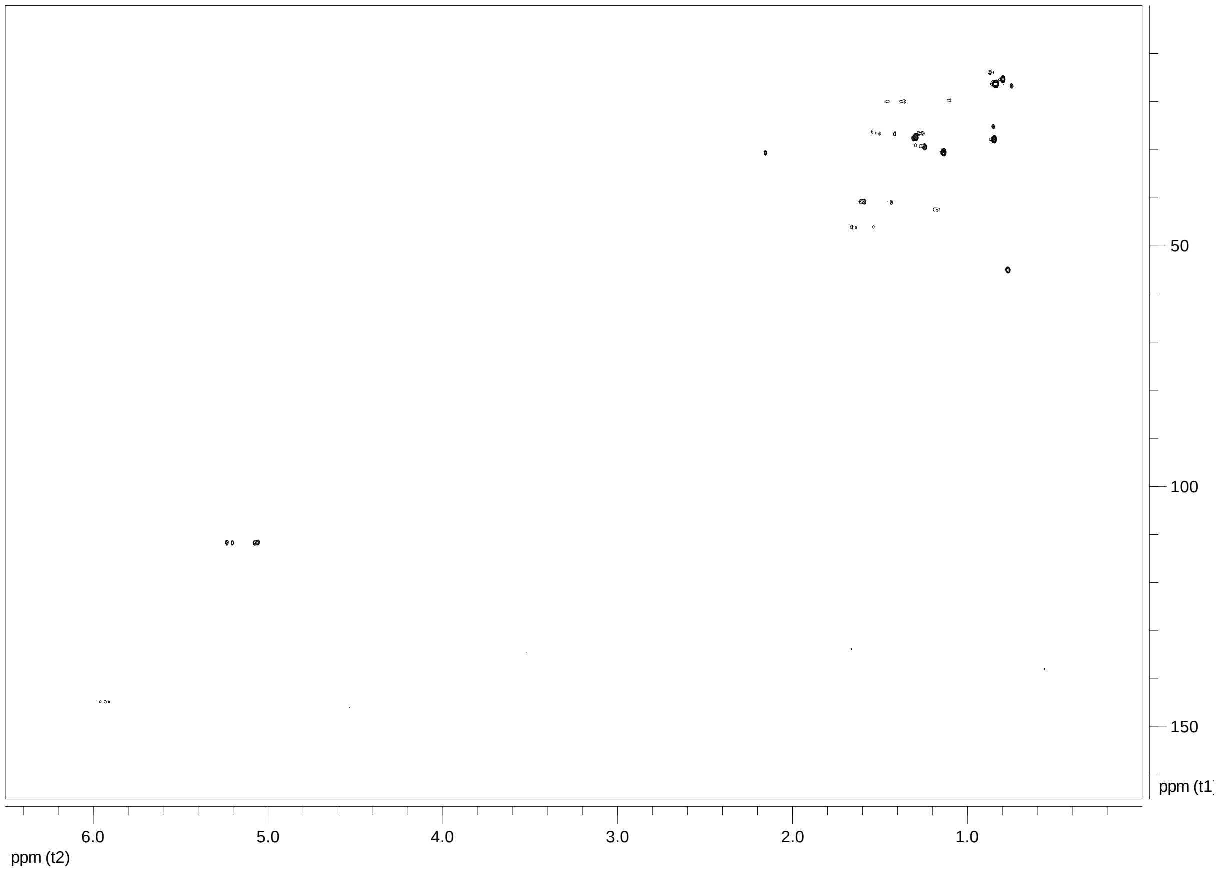

Supplementary Fig. 207. HSQC spectrum (in $CDCl_3$) of compound **8b**.

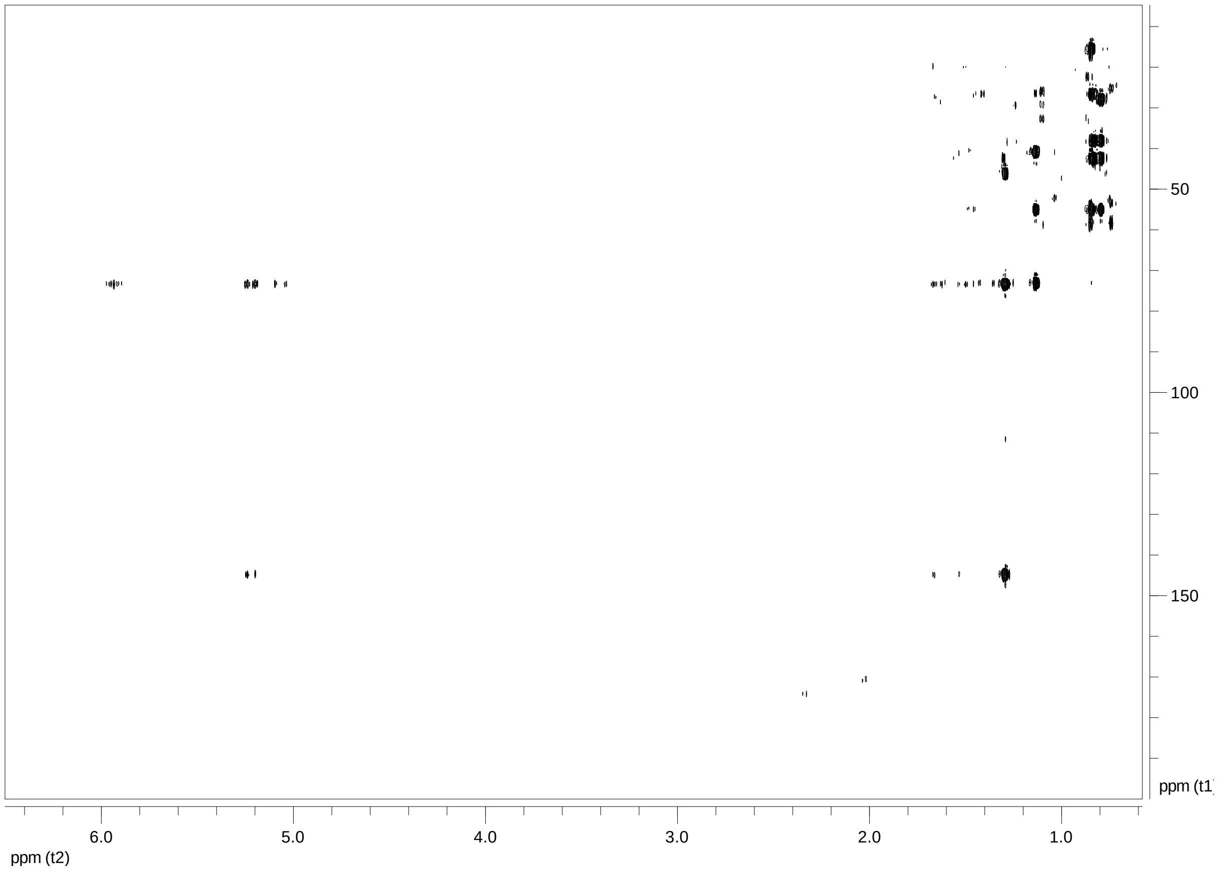

Supplementary Fig. 208. HMBC spectrum (in $CDCl_3$) of compound **8b**.

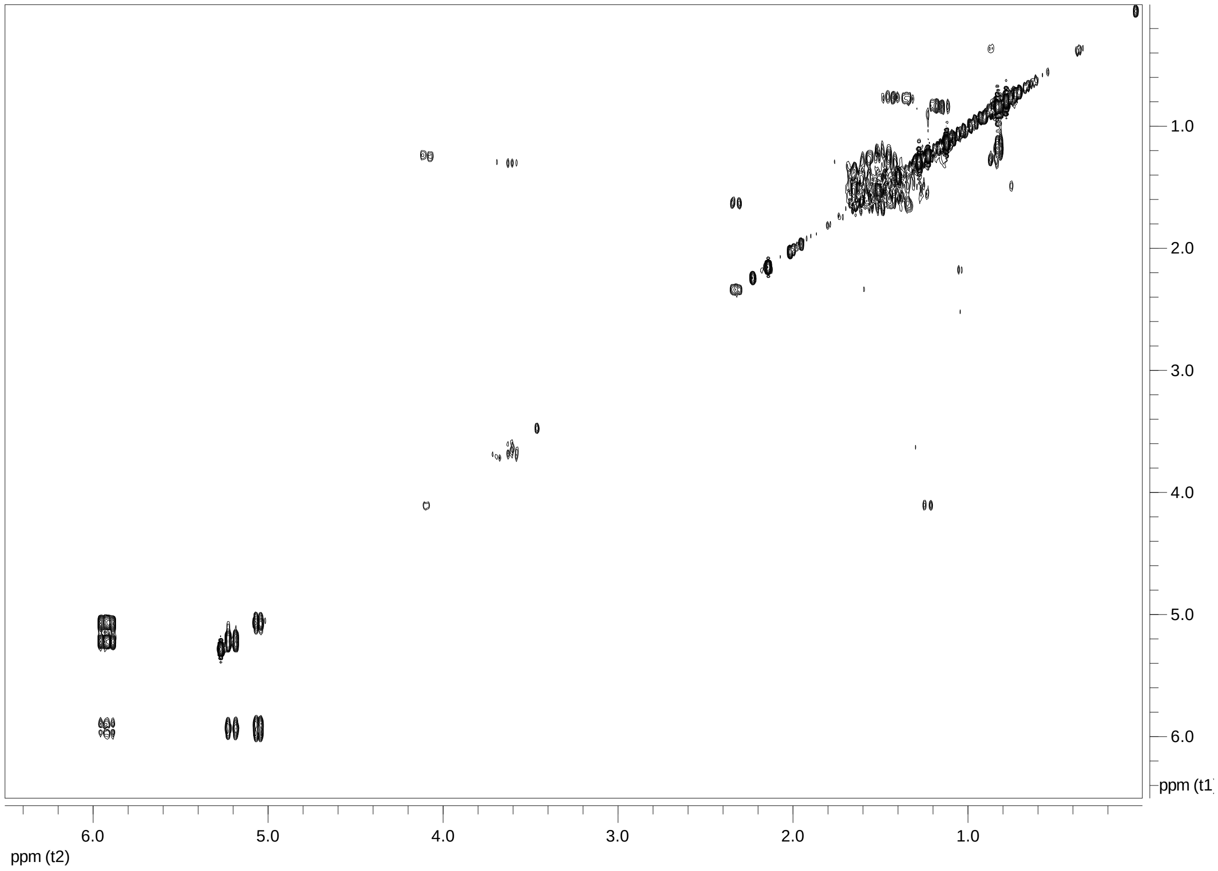

Supplementary Fig. 209. COSY spectrum (in $CDCl_3$) of compound **8b**.

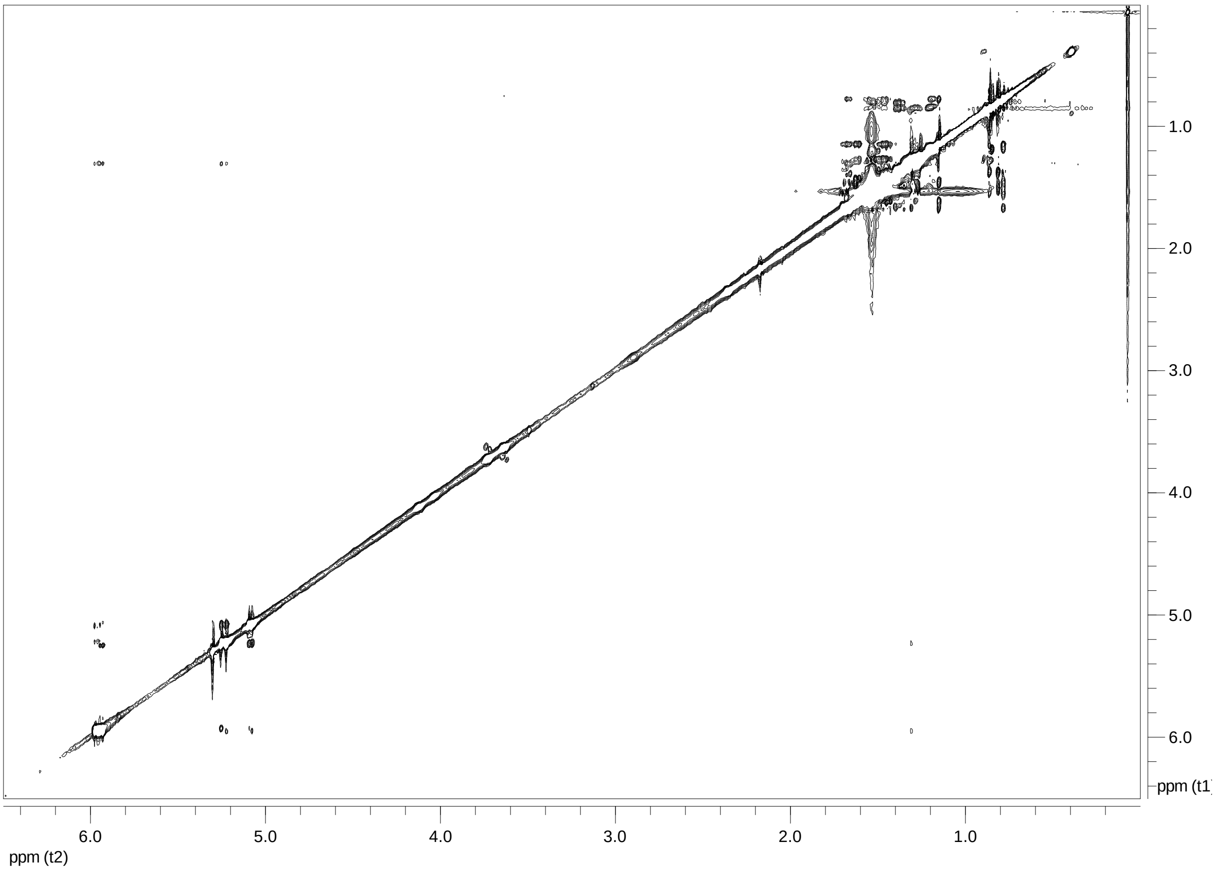

Supplementary Fig. 210. NOESY spectrum (in $CDCl_3$) of compound **8b**.

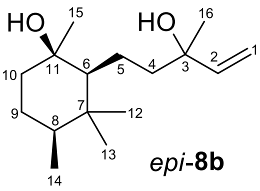

epi-**8b**

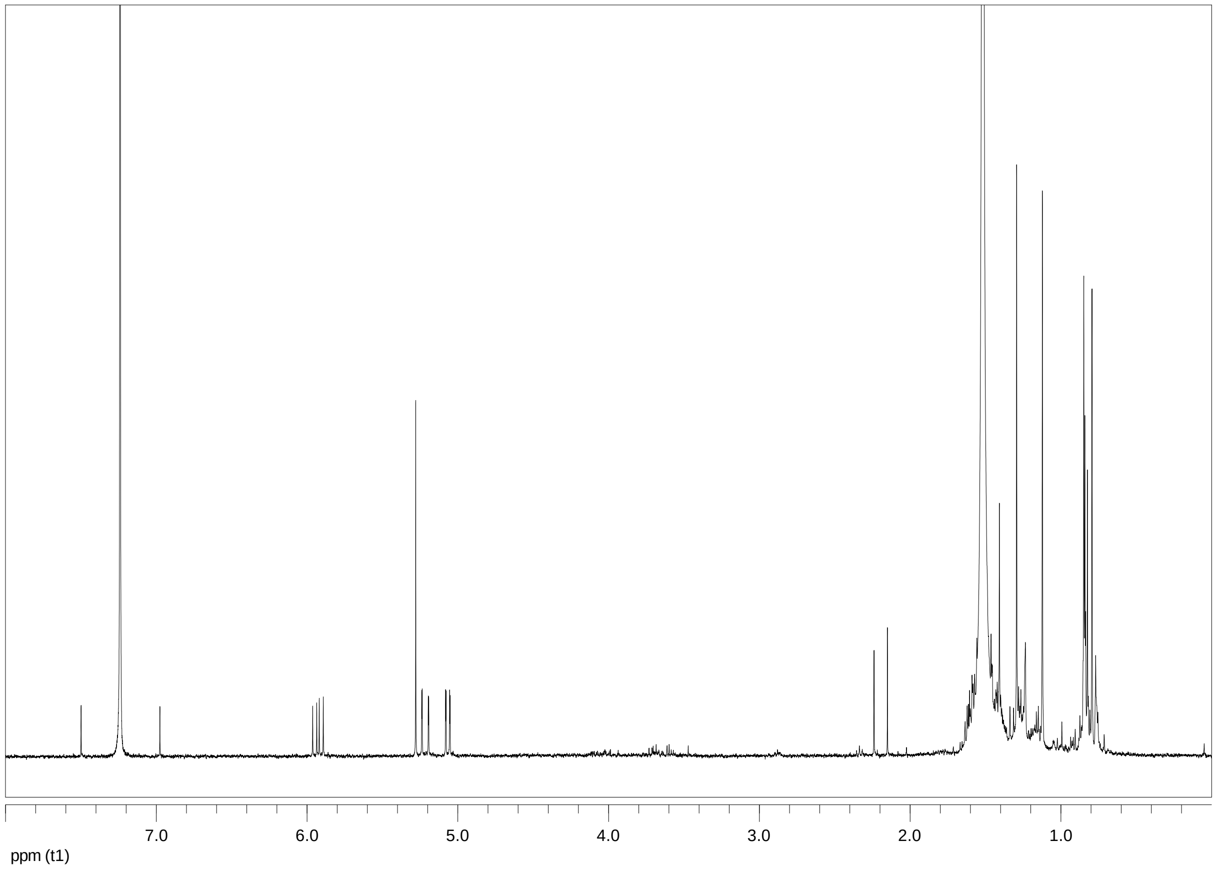

Supplementary Fig. 211. ^{1}H NMR spectrum (in $CDCl_3$) of compound ***epi*-8b**.

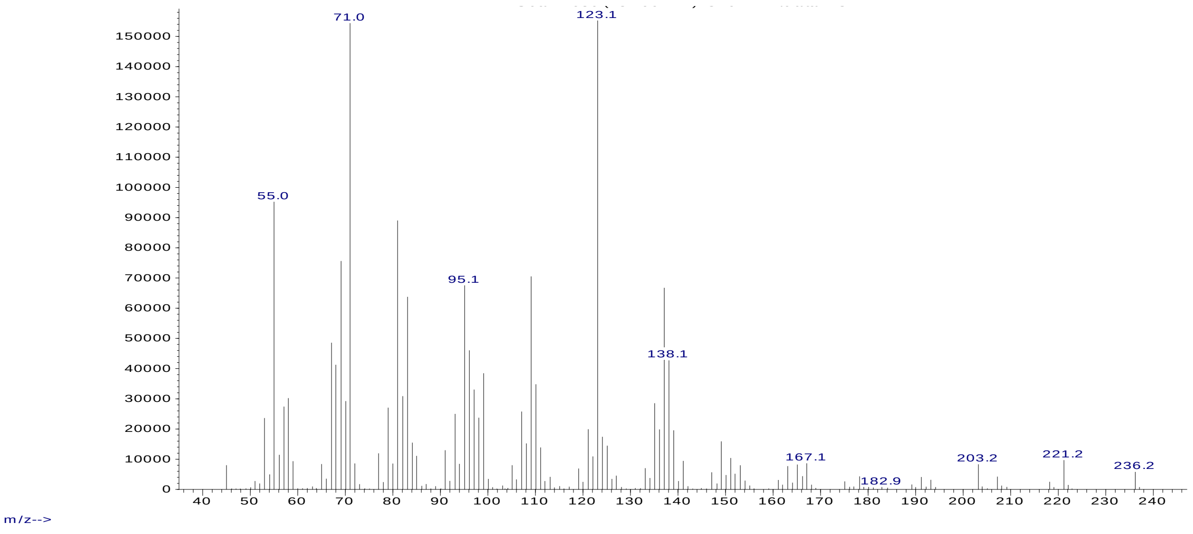

Supplementary Fig. 212. LR-EI-MS spectrum of compound ***epi*-8b**.

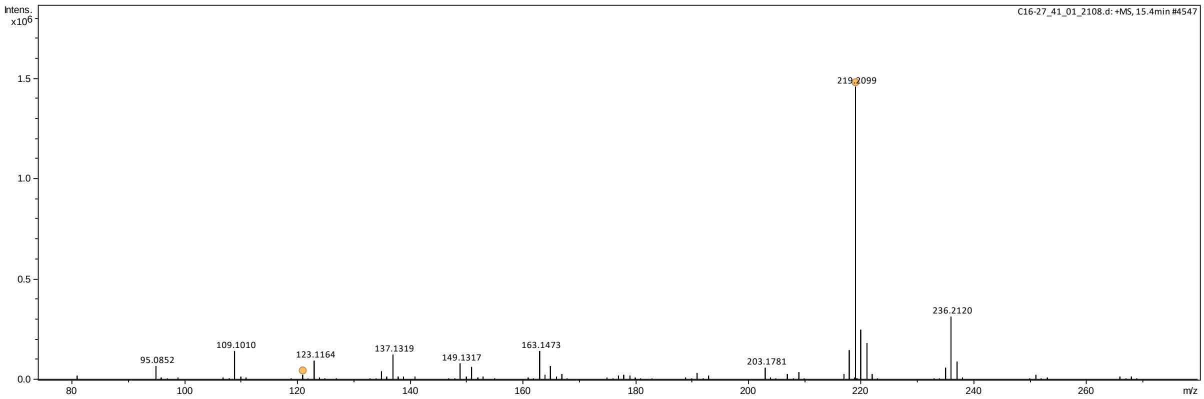

Supplementary Fig. 213. HR-APCI-MS spectrum of compound ***epi*-8b**.

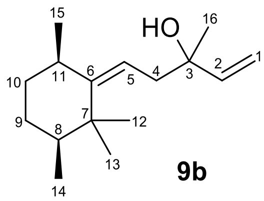

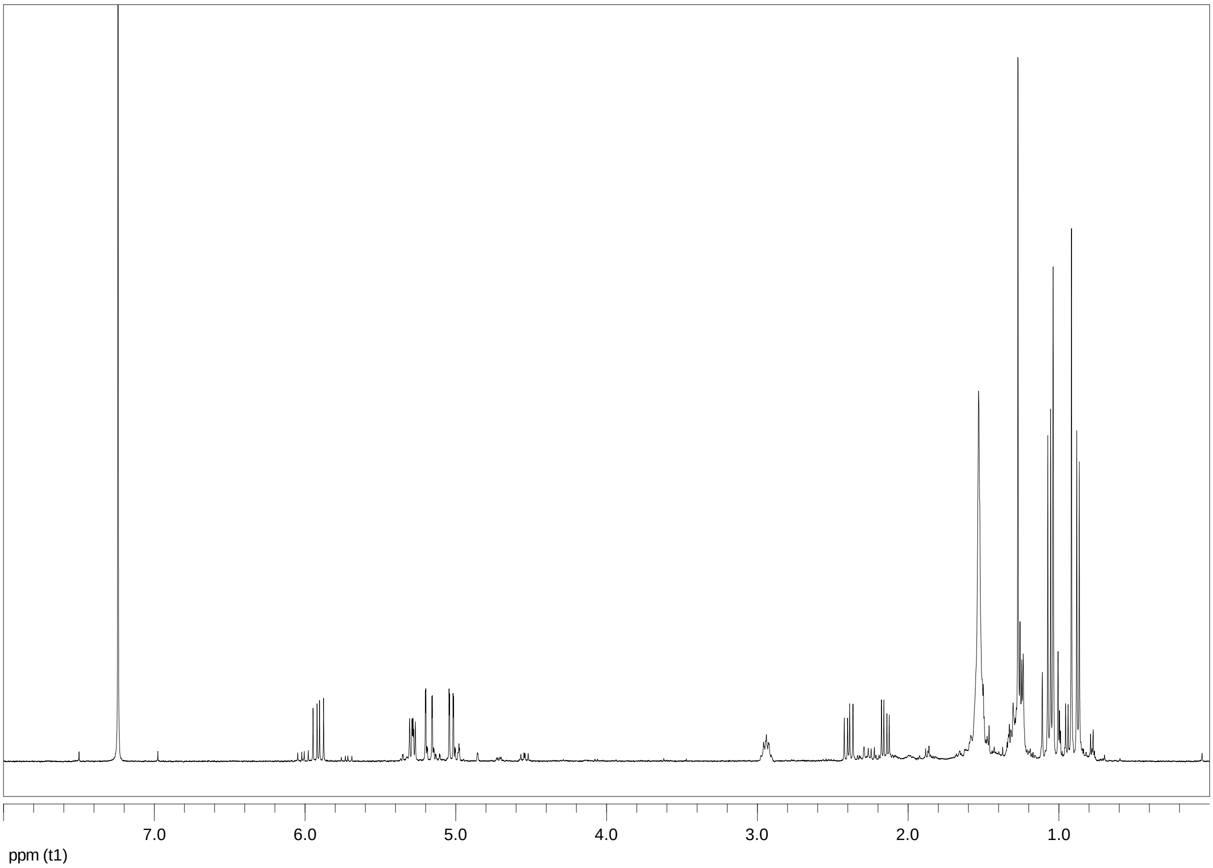

Supplementary Fig. 214. ^{1}H NMR spectrum (in $CDCl_3$) of compound **9b**.

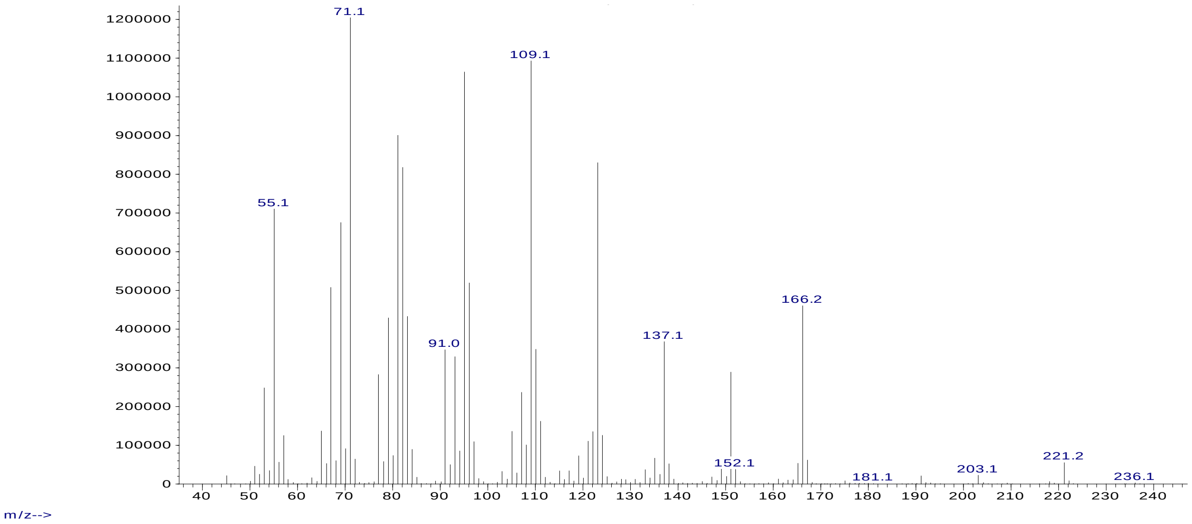

Supplementary Fig. 215. LR-EI-MS spectrum of compound **9b**.

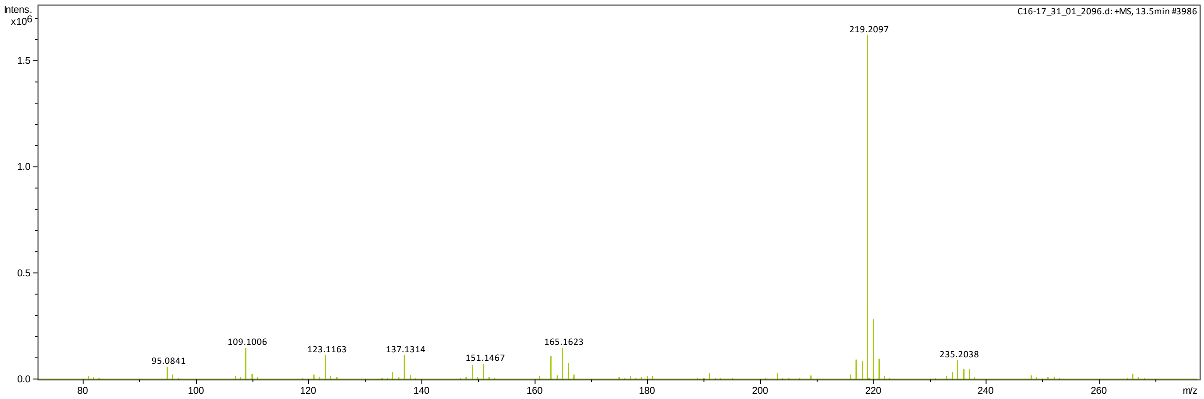

Supplementary Fig. 216. HR-APCI-MS spectrum of compound **9b**.

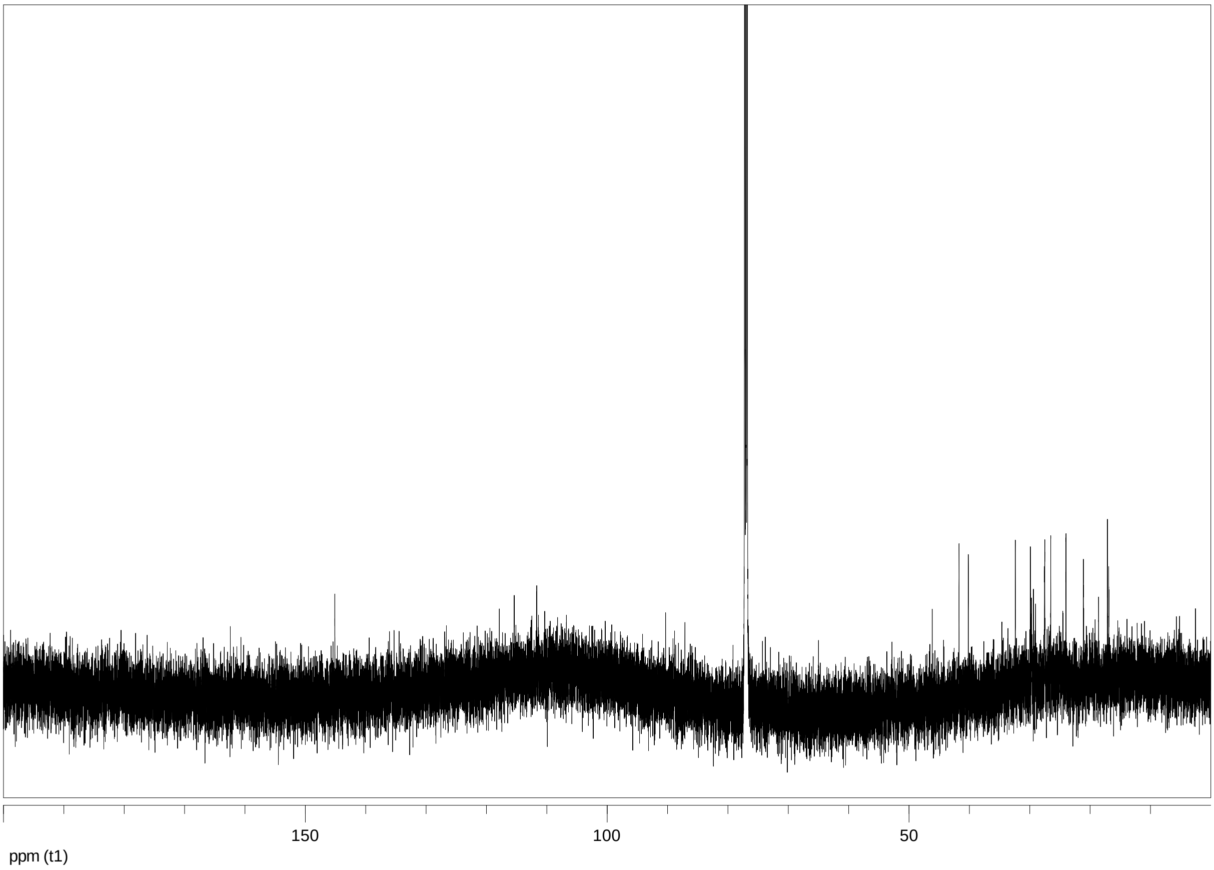

Supplementary Fig. 217. ^{13}C NMR spectrum (in $CDCl_3$) of compound **9b**.

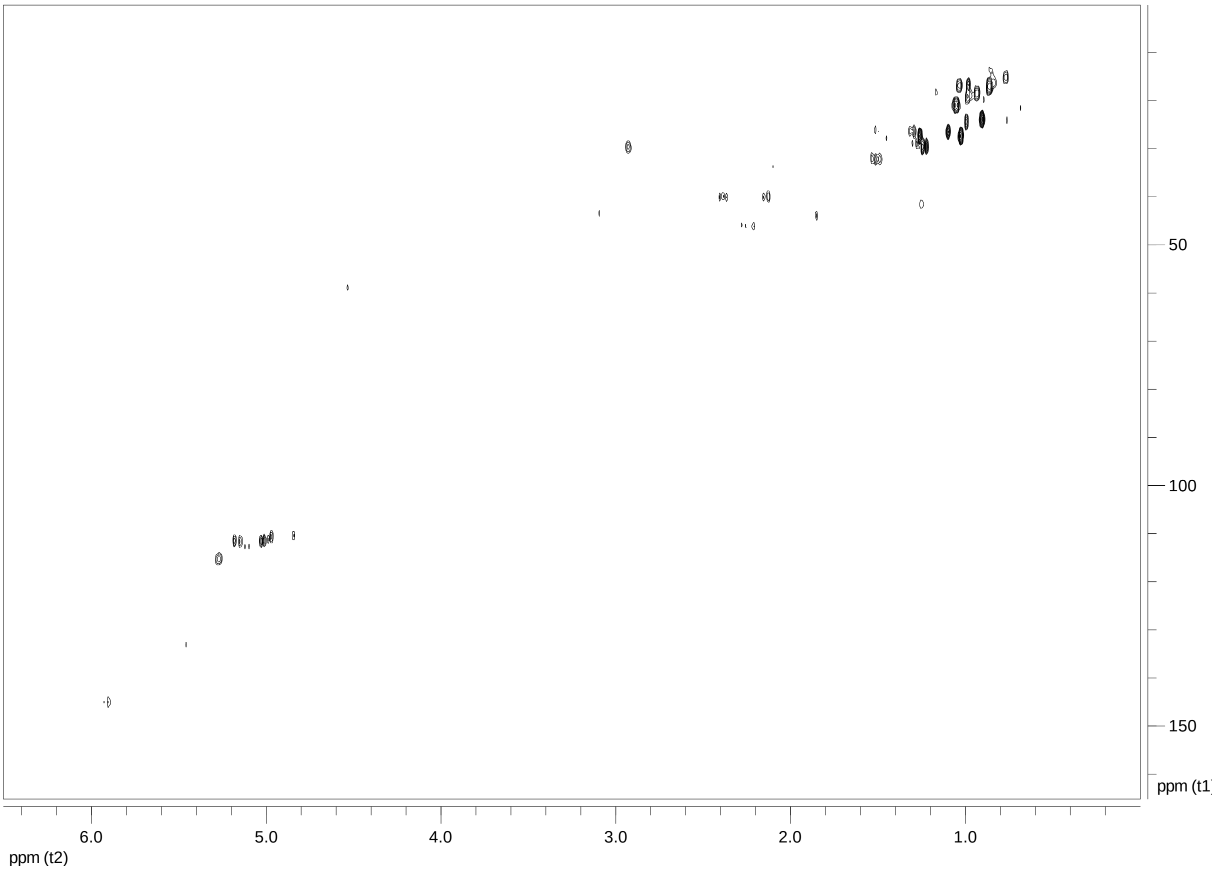

Supplementary Fig. 218. HSQC spectrum (in $CDCl_3$) of compound **9b**.

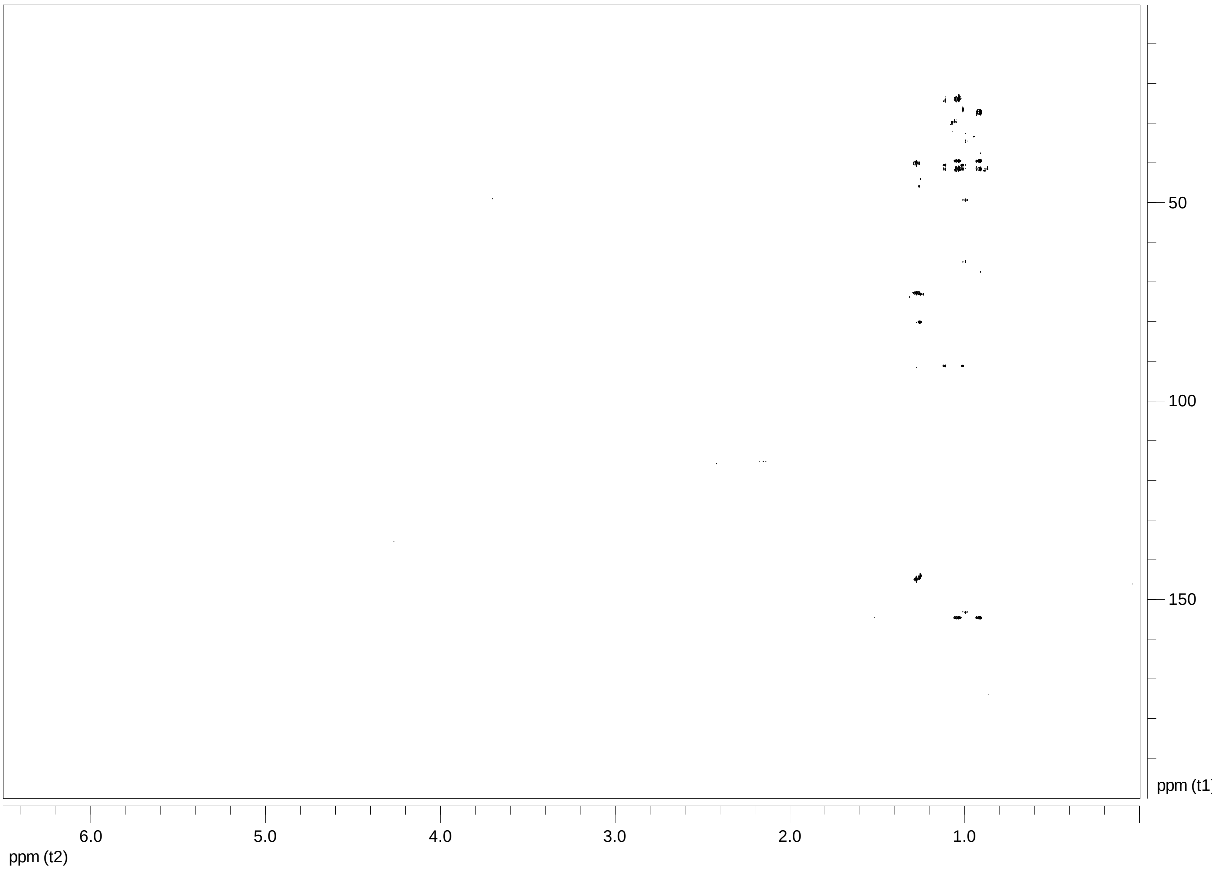

Supplementary Fig. 219. HMBC spectrum (in $CDCl_3$) of compound **9b**.

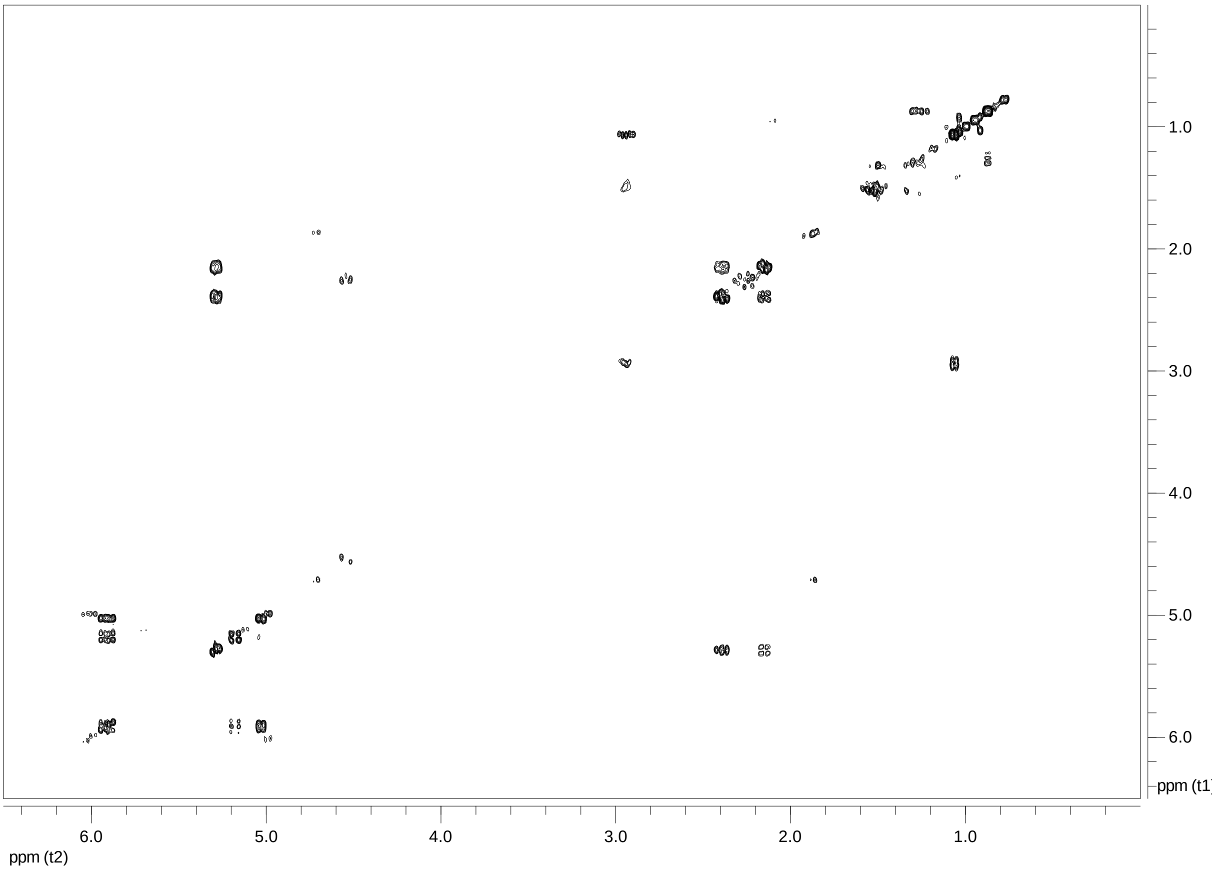

Supplementary Fig. 220. COSY spectrum (in $CDCl_3$) of compound **9b**.

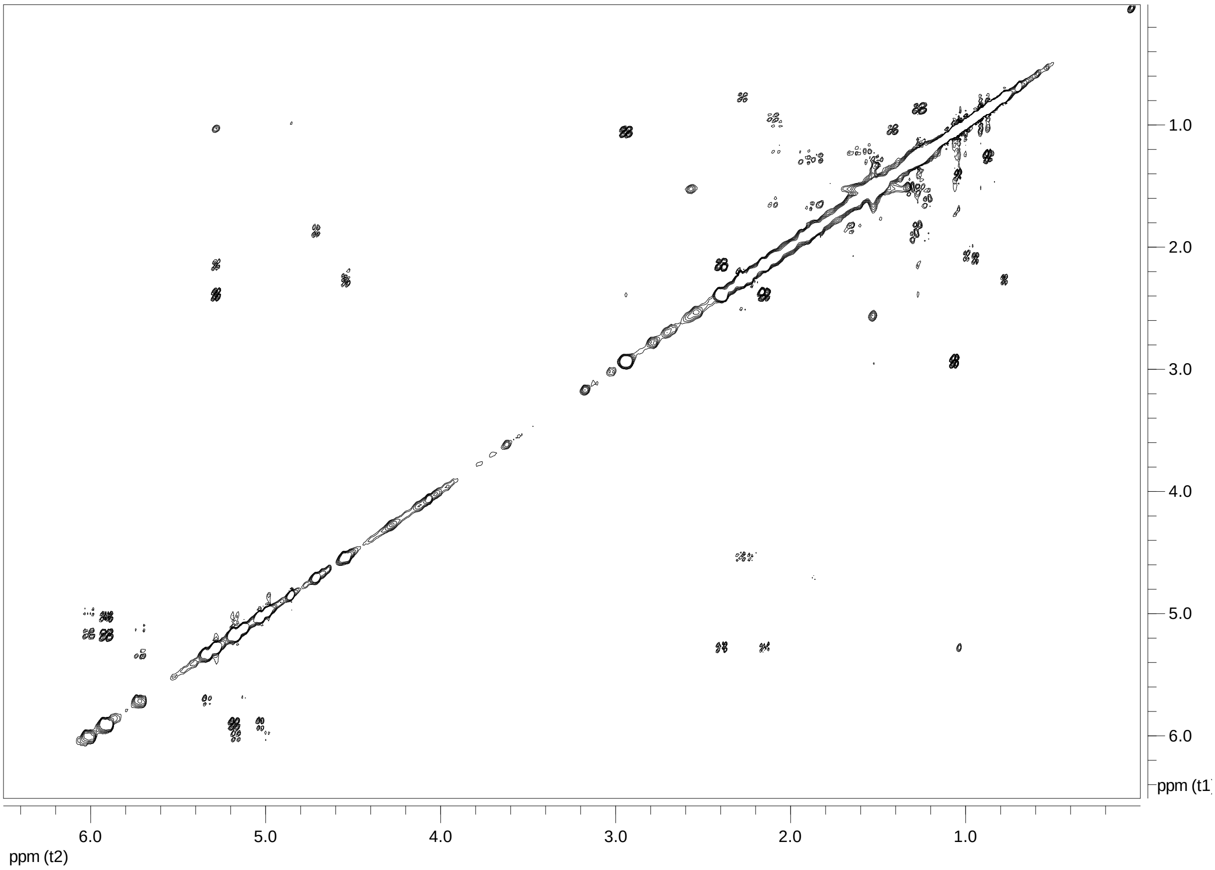

Supplementary Fig. 221. NOESY spectrum (in $CDCl_3$) of compound **9b**.

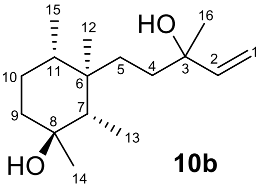

10b

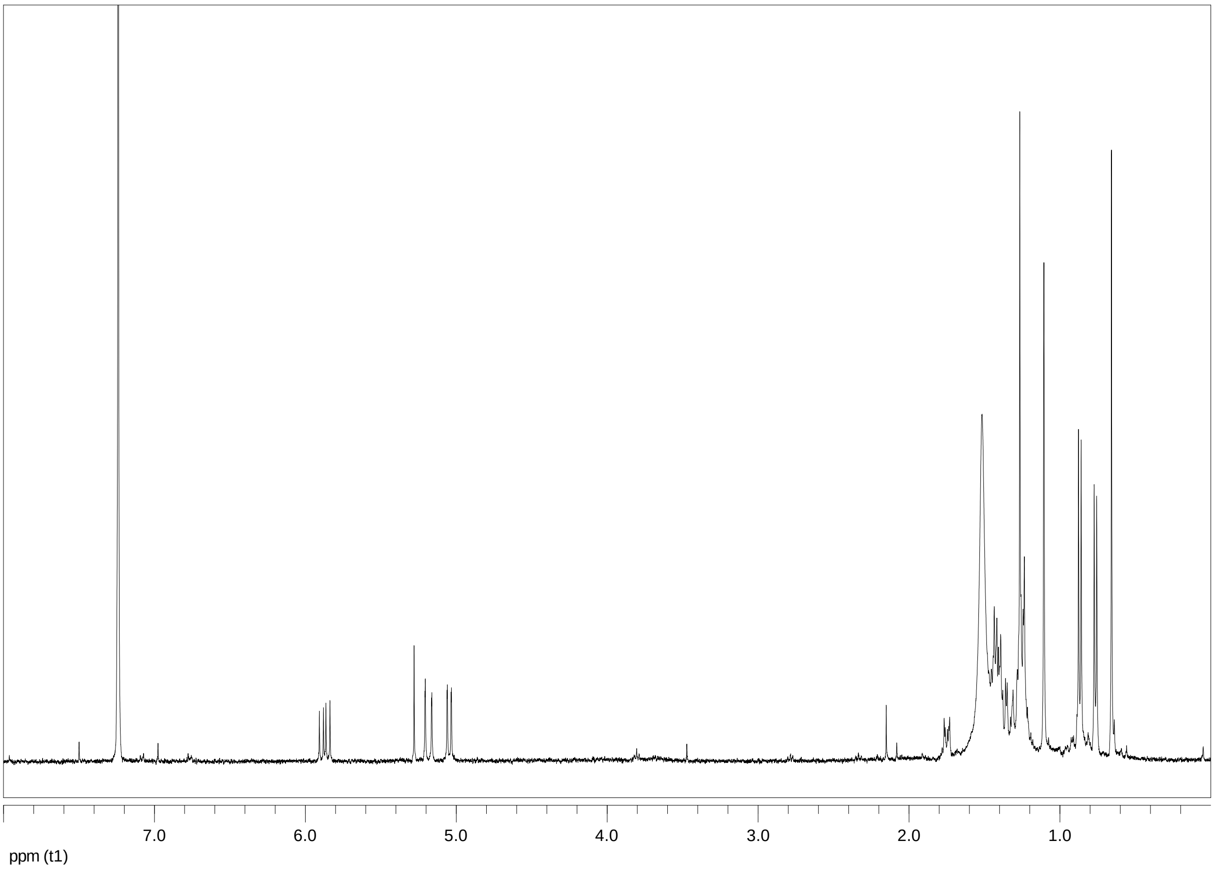

Supplementary Fig. 222. ^{1}H NMR spectrum (in $CDCl_3$) of compound **10b**.

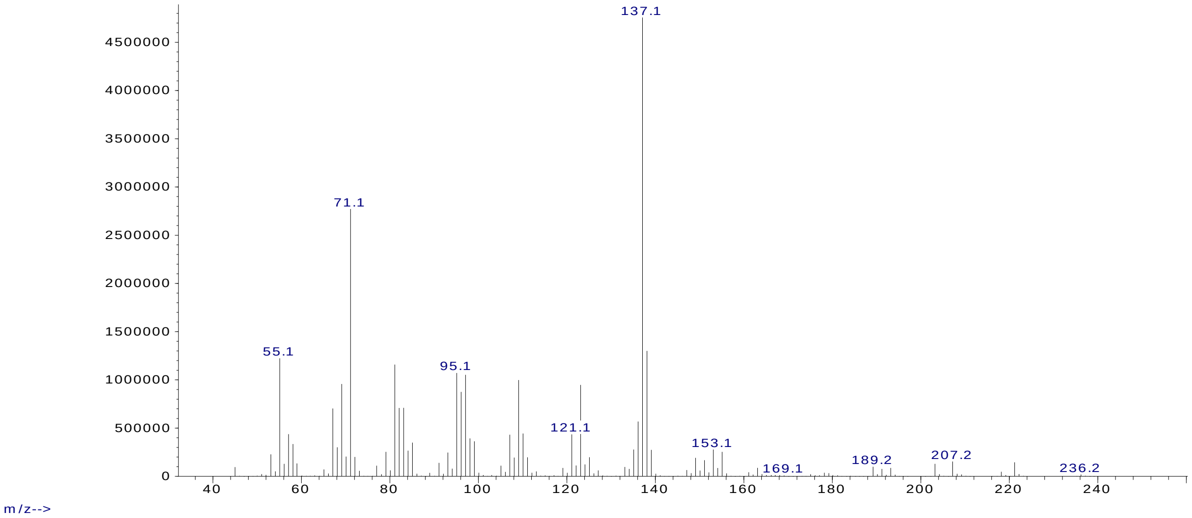

Supplementary Fig. 223. LR-EI-MS spectrum of compound **10b**.

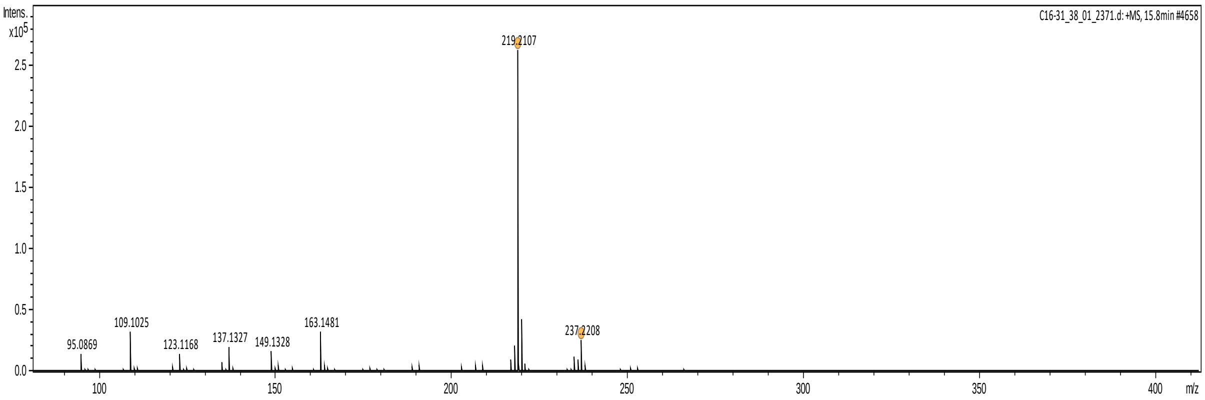

Supplementary Fig. 224. HR-APCI-MS spectrum of compound **10b**.

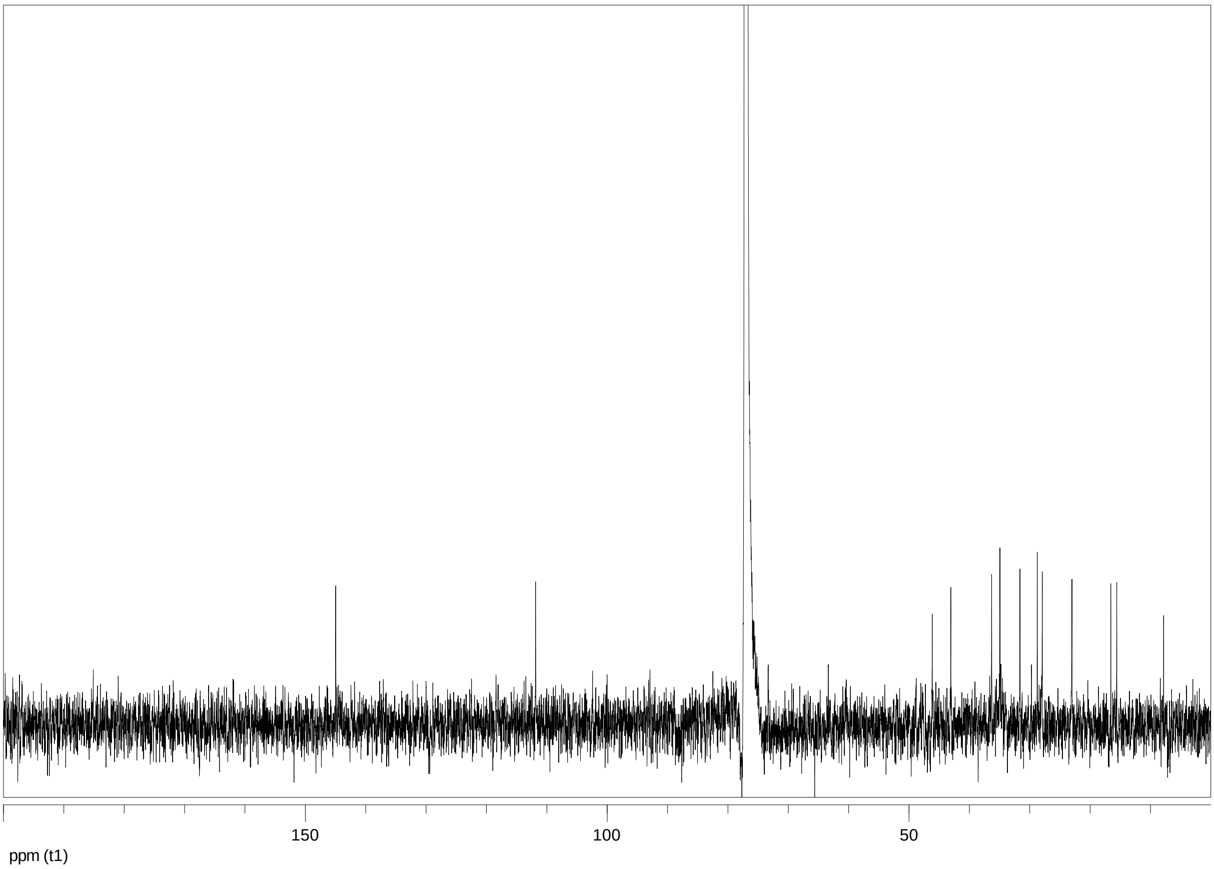

Supplementary Fig. 225. ^{13}C NMR spectrum (in $CDCl_3$) of compound **10b**.

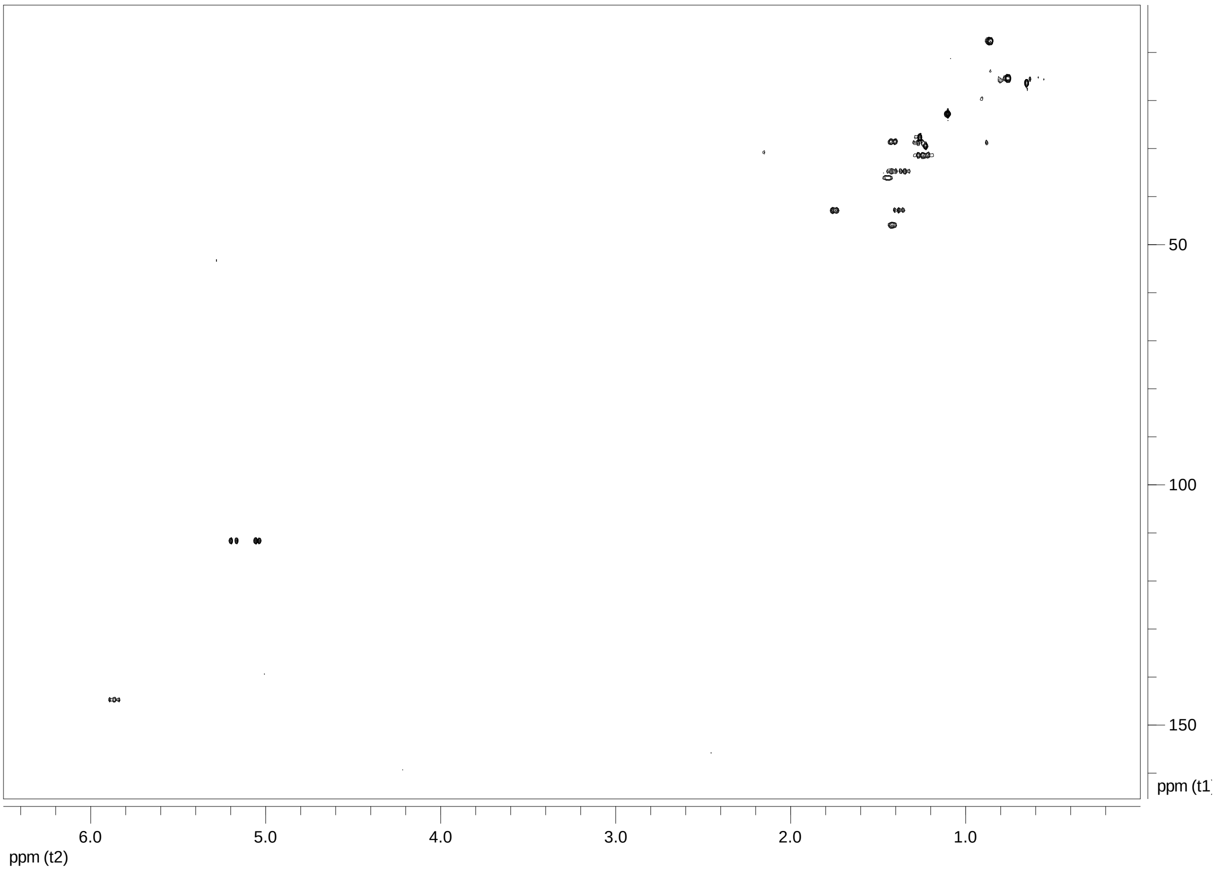

Supplementary Fig. 226. HSQC spectrum (in $CDCl_3$) of compound **10b**.

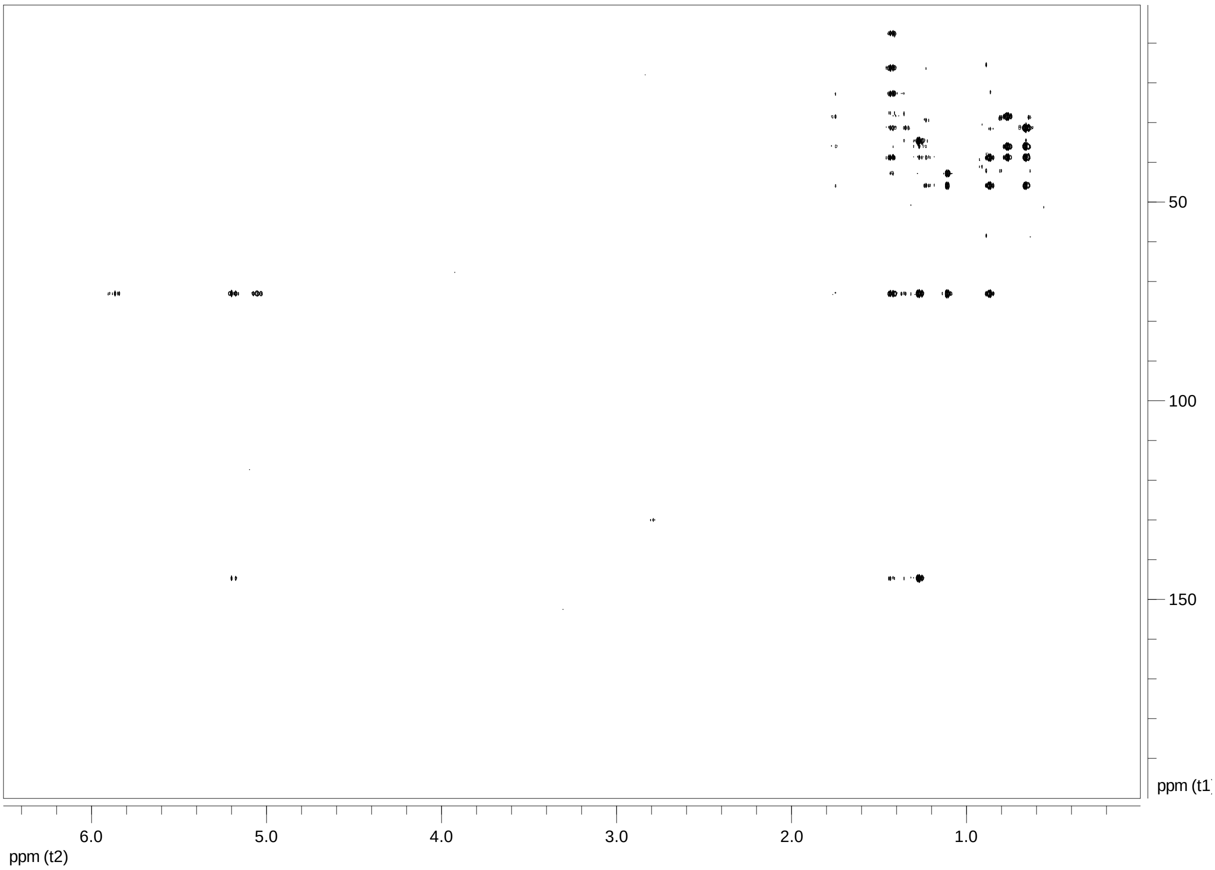

Supplementary Fig. 227. HMBC spectrum (in $CDCl_3$) of compound **10b**.

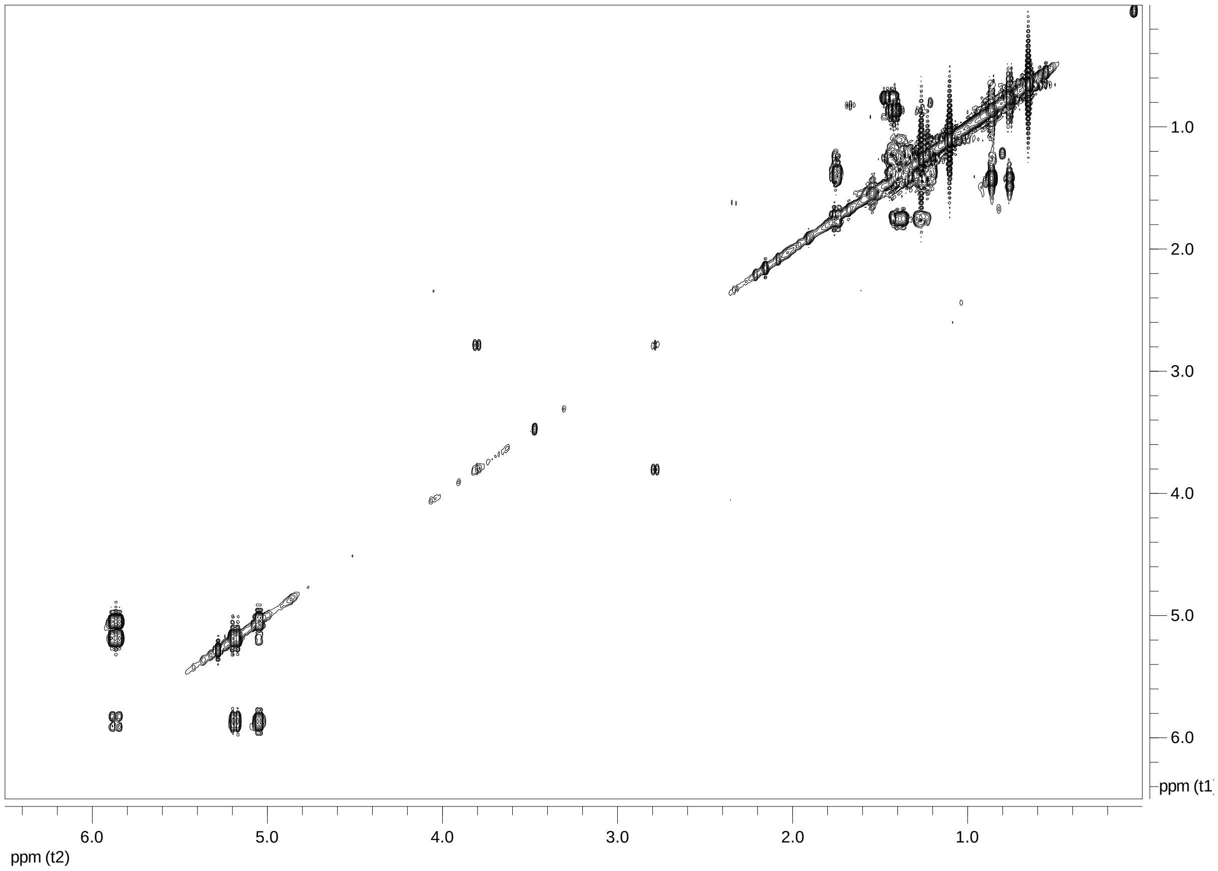

Supplementary Fig. 228. COSY spectrum (in $CDCl_3$) of compound **10b**.

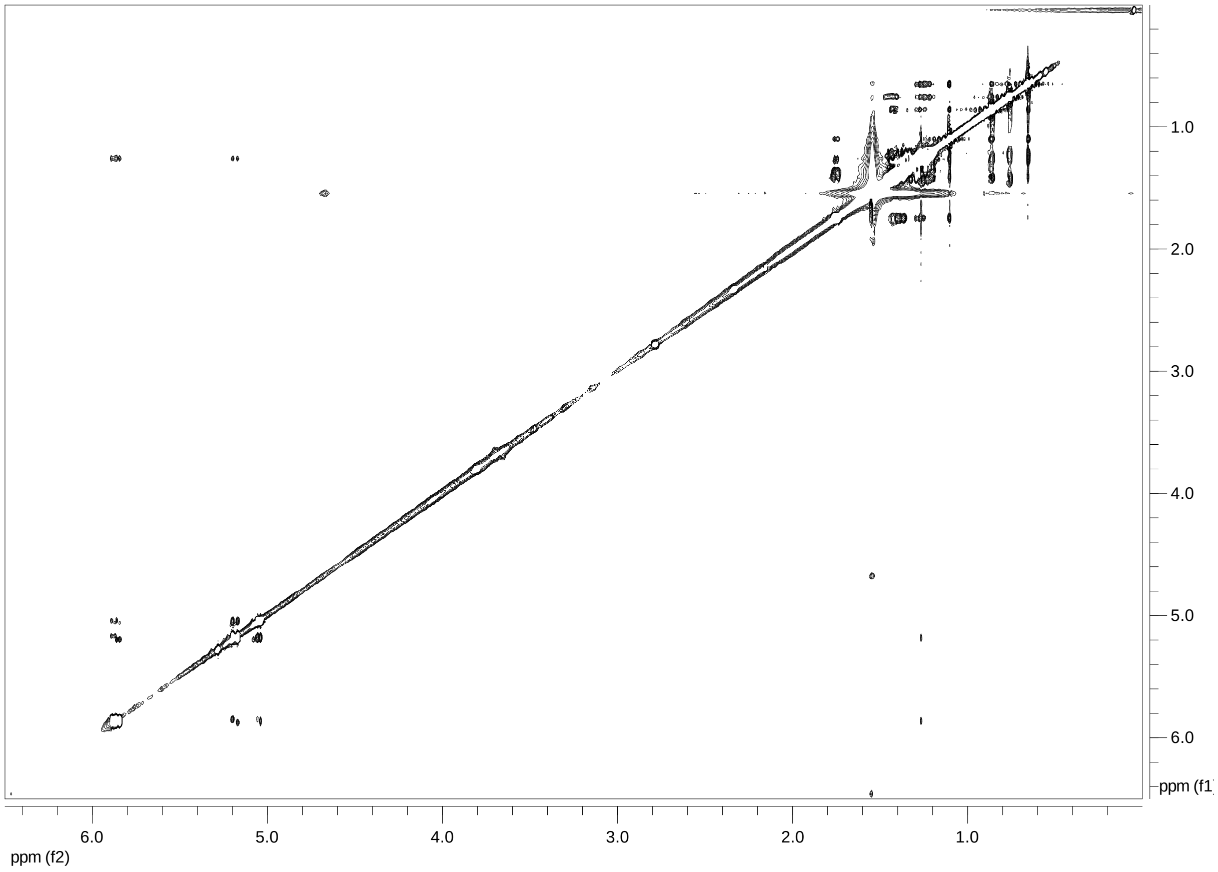

Supplementary Fig. 229. NOESY spectrum (in $CDCl_3$) of compound **10b**.

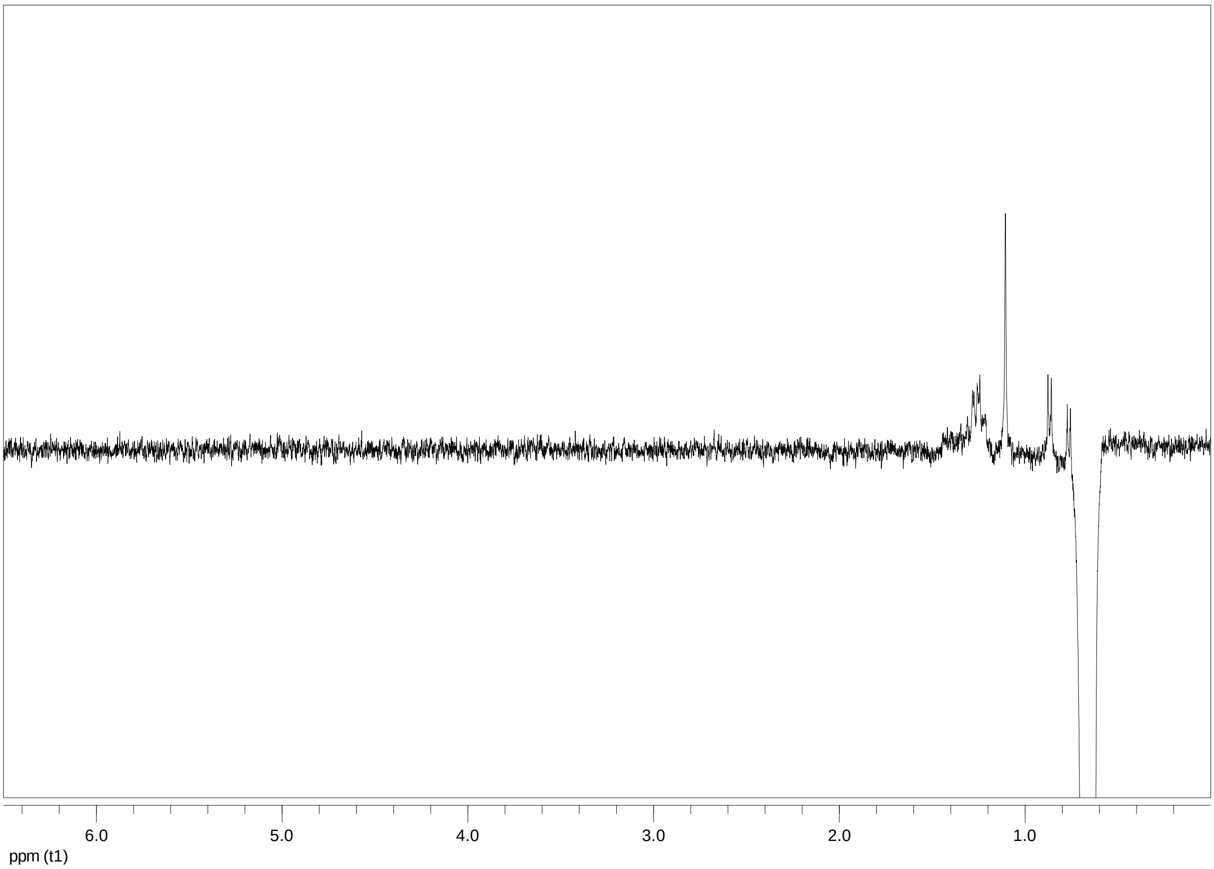

Supplementary Fig. 230. 1D NOE difference spectrum (in $CDCl_3$) of compound **10b** upon irradiation of H_3-12.

Supplementary Notes

Supplementary Note 1. Structure elucidation and compound characterization.

The molecular formulae of the 28 C_{16} compounds isolated in pure form (Supplementary Fig. 1) were indicated by the molecular ion peaks, pseudomolecular ion peaks or fragment ion peaks observed in their LR-EI-MS and HR-APCI-MS spectra (Supplementary Figs. 19, 20, 26, 27, 34, 35, 37, 38, 43, 44, 50, 51, 59, 60, 65, 66, 76, 77, 82, 88, 89, 95, 96, 103, 104, 109, 110, 118, 119, 129, 130, 141, 142, 148, 149, 155, 156, 167, 168, 170, 171, 180, 181, 191, 192, 198, 199, 205, 206, 212, 213, 215, 216, 223, 224). The ^{1}H and ^{13}C chemical shifts of the compounds featuring the same building blocks (**1**–**10**), as well as of the compounds featuring the same side chains (**a**–**d**) were highly conserved regarding the respective parts of the molecules (Supplementary Tables 9–14 and Supplementary Figs. 18, 21, 25, 28, 33, 36, 42, 49, 58, 64, 67, 75, 81, 83, 87, 94, 97, 102, 108, 117, 120, 128, 131, 140, 143, 147, 154, 157, 166, 169, 172, 179, 182, 190, 197, 204, 211, 214, 217, 222, 225). The chemical structures of the isolated C_{16} compounds were determined on the basis of the correlations observed in their HSQC, HMBC and COSY spectra (Supplementary Figs. 16, 22–24, 29–31, 39–41, 45–47, 52–54, 61–63, 68–70, 78–80, 84–86, 90–92, 98–100, 105–107, 111–113, 121–123, 132–134, 144–146, 150–152, 158–160, 173–175, 183–185, 193–195, 200–202, 207–209, 218–220, 226–228), while their relative configurations were assigned on the basis of the cross-peaks and enhancements observed in their NOESY and 1D NOE spectra, respectively (Supplementary Figs. 17, 32, 48, 55–57, 71–74, 93, 101, 114–116, 124–127, 135–139, 153, 161–165, 176–178, 186–189, 196, 203, 210, 221, 229, 230).

Presodorifenol (**1a**): colorless oil; ^{1}H NMR data, see Supplementary Table 9; ^{13}C NMR data, see Supplementary Table 13; LR-EI-MS *m/z* 236 $[M]^{+}$; HR-APCI-MS *m/z* 237.2328 $[M+H]^{+}$ (calcd. for $C_{16}H_{29}O$ 237.2213).

Presodorifelool (**1b**): colorless oil; ^{1}H NMR data, see Supplementary Table 9; ^{13}C NMR data, see Supplementary Table 13; LR-EI-MS *m/z* 236 $[M]^{+}$; HR-APCI-MS *m/z* 237.2196 $[M+H]^{+}$ (calcd. for $C_{16}H_{29}O$ 237.2213).

epi-Presodorifelool (***epi*-1b**): colorless oil; ^{1}H NMR data, see Supplementary Table 9; LR-EI-MS *m/z* 236 $[M]^{+}$; HR-APCI-MS *m/z* 237.2203 $[M+H]^{+}$ (calcd. for $C_{16}H_{29}O$ 237.2213).

2,3-Dihydro-presodorifenol (**1c**): colorless oil; ^{1}H NMR data, see Supplementary Table 9; ^{13}C NMR data, see Supplementary Table 13; LR-EI-MS *m/z* 238 $[M]^{+}$; HR-APCI-MS *m/z* 239.2356 $[M+H]^{+}$ (calcd. for $C_{16}H_{31}O$ 239.2369).

β-Presodorifene (**1d**): colorless oil; ^{1}H NMR data, see Supplementary Table 9; ^{13}C NMR data, see Supplementary Table 13; LR-EI-MS *m/z* 218 $[M]^+$; HR-APCI-MS *m/z* 219.2096 $[M+H]^+$ (calcd. for $C_{16}H_{27}$ 219.2107).

13-Hydroxy-presodorifelool (**13OH-1b**): colorless oil; ^{1}H NMR data, see Supplementary Table 9; ^{13}C NMR data, see Supplementary Table 13; LR-EI-MS *m/z* 252 $[M]^+$; HR-APCI-MS *m/z* 235.2051 $[M-H_2O+H]^+$ (calcd. for $C_{16}H_{27}O$ 235.2056).

Plymuthenol (**2a**): colorless oil; ^{1}H NMR data, see Supplementary Table 10; ^{13}C NMR data, see Supplementary Table 13; LR-EI-MS *m/z* 236 $[M]^+$; HR-APCI-MS *m/z* 237.2198 $[M+H]^+$ (calcd. for $C_{16}H_{29}O$ 237.2213).

Plymuthelool (**2b**): colorless oil; ^{1}H NMR data, see Supplementary Table 10; ^{13}C NMR data, see Supplementary Table 13; LR-EI-MS *m/z* 236 $[M]^+$; HR-APCI-MS *m/z* 219.2103 $[M-H_2O+H]^+$ (calcd. for $C_{16}H_{27}$ 219.2107).

2,3-Dihydro-plymuthenol (**2c**): colorless oil; ^{1}H NMR data, see Supplementary Table 10; ^{13}C NMR data, see Supplementary Table 13; LR-EI-MS *m/z* 238 $[M]^+$; HR-APCI-MS *m/z* 239.2370 $[M+H]^+$ (calcd. for $C_{16}H_{31}O$ 239.2369).

β-Plymuthene (**2d**): colorless oil; ^{1}H NMR data, see Supplementary Table 10; ^{13}C NMR data, see Supplementary Table 13; HR-APCI-MS *m/z* 219.2097 $[M+H]^+$ (calcd. for $C_{16}H_{27}$ 219.2107).

Thorvaldsenol (**3a**): colorless oil; ^{1}H NMR data, see Supplementary Table 10; ^{13}C NMR data, see Supplementary Table 13; LR-EI-MS *m/z* 236 $[M]^+$; HR-APCI-MS *m/z* 219.2093 $[M-H_2O+H]^+$ (calcd. for $C_{16}H_{27}$ 219.2107).

Thorvaldselool (**3b**): colorless oil; ^{1}H NMR data, see Supplementary Table 10; ^{13}C NMR data, see Supplementary Table 13; LR-EI-MS *m/z* 236 $[M]^+$; HR-APCI-MS *m/z* 219.2101 $[M-H_2O+H]^+$ (calcd. for $C_{16}H_{27}$ 219.2107).

2,3-Dihydro-thorvaldsenol (**3c**): colorless oil; ^{1}H NMR data, see Supplementary Table 10; ^{13}C NMR data, see Supplementary Table 13; LR-EI-MS *m/z* 238 $[M]^+$; HR-APCI-MS *m/z* 239.2354 $[M+H]^+$ (calcd. for $C_{16}H_{31}O$ 239.2369).

9-Hydroxy-thorvaldselool (**9OH-3b**): colorless oil; ^{1}H NMR data, see Supplementary Table 10; ^{13}C NMR data, see Supplementary Table 13; LR-EI-MS *m/z* 252 $[M]^+$; HR-APCI-MS *m/z* 217.1944 $[M-2H_2O+H]^+$ (calcd. for $C_{16}H_{25}$ 217.1951).

Weylandtenol (**4a**): colorless oil; ^{1}H NMR data, see Supplementary Table 11; ^{13}C NMR data, see Supplementary Table 14; LR-EI-MS *m/z* 236 $[M]^+$; HR-APCI-MS *m/z* 237.2203 $[M+H]^+$ (calcd. for $C_{16}H_{29}O$ 237.2213).

Weylandtelool (**4b**): colorless oil; ^{1}H NMR data, see Supplementary Table 11; ^{13}C NMR data, see Supplementary Table 14; LR-EI-MS *m/z* 236 $[M]^+$; HR-APCI-MS *m/z* 219.2105 $[M-H_2O+H]^+$ (calcd. for $C_{16}H_{27}$ 219.2107).

2,3-Dihydro-weylandtenol (**4c**): colorless oil; ^{1}H NMR data, see Supplementary Table 11; ^{13}C NMR data, see Supplementary Table 14; LR-EI-MS *m/z* 238 $[M]^+$; HR-APCI-MS *m/z* 239.2352 $[M+H]^+$ (calcd. for $C_{16}H_{31}O$ 239.2369).

Blixenol (**5a**): colorless oil; ^{1}H NMR data, see Supplementary Table 11; ^{13}C NMR data, see Supplementary Table 14; LR-EI-MS *m/z* 236 $[M-H_2O]^+$; HR-APCI-MS *m/z* 237.2202 $[M-H_2O+H]^+$ (calcd. for $C_{16}H_{29}O$ 237.2213).

Blixelool (**5b**): colorless oil; ^{1}H NMR data, see Supplementary Table 11; ^{13}C NMR data, see Supplementary Table 14; LR-EI-MS *m/z* 236 $[M-H_2O]^+$; HR-APCI-MS *m/z* 219.2103 $[M-2H_2O+H]^+$ (calcd. for $C_{16}H_{27}$ 219.2107).

epi-Blixelool (***epi*-5b**): colorless oil; ^{1}H NMR data, see Supplementary Table 11; LR-EI-MS *m/z* 236 $[M-H_2O]^+$; HR-APCI-MS *m/z* 219.2107 $[M-2H_2O+H]^+$ (calcd. for $C_{16}H_{27}$ 219.2107).

Kimlarsenol (**6a**): colorless oil; ^{1}H NMR data, see Supplementary Table 11; ^{13}C NMR data, see Supplementary Table 14; LR-EI-MS *m/z* 236 $[M]^+$; HR-APCI-MS *m/z* 219.2098 $[M-H_2O+H]^+$ (calcd. for $C_{16}H_{27}$ 219.2107).

Serratinol (**7a**): colorless oil; ^{1}H NMR data, see Supplementary Table 12; ^{13}C NMR data, see Supplementary Table 14; LR-EI-MS *m/z* 236 $[M]^+$; HR-APCI-MS *m/z* 237.2204 $[M+H]^+$ (calcd. for $C_{16}H_{29}O$ 237.2213).

Serratilool (**7b**): colorless oil; ^{1}H NMR data, see Supplementary Table 12; ^{13}C NMR data, see Supplementary Table 14; LR-EI-MS *m/z* 236 $[M]^+$; HR-APCI-MS *m/z* 219.2092 $[M-H_2O+H]^+$ (calcd. for $C_{16}H_{27}$ 219.2107).

2,3-Dihydro-serratinol (**7c**): colorless oil; ^{1}H NMR data, see Supplementary Table 12; ^{13}C NMR data, see Supplementary Table 14; LR-EI-MS *m/z* 238 $[M]^+$; HR-APCI-MS *m/z* 239.2363 $[M+H]^+$ (calcd. for $C_{16}H_{31}O$ 239.2369).

Jacobselool (**8b**): colorless oil; ^{1}H NMR data, see Supplementary Table 12; ^{13}C NMR data, see Supplementary Table 14; LR-EI-MS *m/z* 236 $[M-H_2O]^+$; HR-APCI-MS *m/z* 219.2103 $[M-2H_2O+H]^+$ (calcd. for $C_{16}H_{27}$ 219.2107).

epi-Jacobselool (***epi*-8b**): colorless oil; ^{1}H NMR data, see Supplementary Table 12; LR-EI-MS *m/z* 236 $[M-H_2O]^+$; HR-APCI-MS *m/z* 219.2099 $[M-2H_2O+H]^+$ (calcd. for $C_{16}H_{27}$ 219.2107).

Hammersholool (**9b**): colorless oil; ^{1}H NMR data, see Supplementary Table 12; ^{13}C NMR data, see Supplementary Table 14; LR-EI-MS *m/z* 236 $[M]^+$; HR-APCI-MS *m/z* 219.2097 $[M-H_2O+H]^+$ (calcd. for $C_{16}H_{27}$ 219.2107).

Anchelool (**10b**): colorless oil; ^{1}H NMR data, see Supplementary Table 12; ^{13}C NMR data, see Supplementary Table 14; LR-EI-MS *m/z* 236 $[M-H_2O]^+$; HR-APCI-MS *m/z* 237.2208 $[M-H_2O+H]^+$ (calcd. for $C_{16}H_{29}O$ 237.2213).

Supplementary references

1 Steele, C. L., Crock, J., Bohlmann, J. & Croteau, R. Sesquiterpene synthases from grand fir (*Abies grandis*). Comparison of constitutive and wound-induced activities, and cDNA isolation, characterization, and bacterial expression of delta-selinene synthase and gamma-humulene synthase. *J. Biol. Chem.* **273**, 2078-2089 (1998).

2 Ignea, C. *et al.* Improving yeast strains using recyclable integration cassettes, for the production of plant terpenoids. *Microb. Cell Fact.* **10**, 4 (2011).

3 Kampranis, S. C. *et al.* Rational conversion of substrate and product specificity in a salvia monoterpene synthase: structural insights into the evolution of terpene synthase function. *Plant Cell* **19**, 1994-2005 (2007).

4 Ignea, C. *et al.* Positive genetic interactors of HMG2 identify a new set of genetic perturbations for improving sesquiterpene production in *Saccharomyces cerevisiae*. *Microb. Cell Fact.* **11**, 162 (2012).

5 Tsaballa, A. *et al.* Use of the de novo transcriptome analysis of silver-leaf nightshade (*Solanum elaeagnifolium*) to identify gene expression changes associated with wounding and terpene biosynthesis. *BMC Genom.* **16**, 504 (2015).

6 Martin, D. M., Faldt, J. & Bohlmann, J. Functional characterization of nine Norway Spruce TPS genes and evolution of gymnosperm terpene synthases of the TPS-d subfamily. *Plant Physiol.* **135**, 1908-1927 (2004).

7 Schepmann, H. G., Pang, J. & Matsuda, S. P. Cloning and characterization of *Ginkgo biloba* levopimaradiene synthase which catalyzes the first committed step in ginkgolide biosynthesis. *Arch. Biochem. Biophys.* **392**, 263-269 (2001).

8 Ignea, C. *et al.* Reconstructing the chemical diversity of labdane-type diterpene biosynthesis in yeast. *Metab. Eng.* **28**, 91-103 (2015).

9 Sallaud, C. *et al.* Characterization of two genes for the biosynthesis of the labdane diterpene Z-abienol in tobacco (*Nicotiana tabacum*) glandular trichomes. *Plant J.* **72**, 1-17 (2012).

10 Schalk, M. *et al.* Toward a biosynthetic route to sclareol and amber odorants. *J. Am. Chem. Soc.* **134**, 18900-18903 (2012).

11 Dairi, T. *et al.* Eubacterial diterpene cyclase genes essential for production of the isoprenoid antibiotic terpentecin. *J. Bacteriol.* **183**, 6085-6094 (2001).

12 Kiss, H. *et al.* Complete genome sequence of the filamentous gliding predatory bacterium *Herpetosiphon aurantiacus* type strain (114-95(T)). *Stand. Genom. Sci.* **5**, 356-370 (2011).

13 Ro, D. K. *et al.* Microarray expression profiling and functional characterization of AtTPS genes: duplicated *Arabidopsis thaliana* sesquiterpene synthase genes At4g13280 and At4g13300 encode root-specific and wound-inducible (Z)-gamma-bisabolene synthases. *Arch. Biochem. Biophys.* **448**, 104-116 (2006).

14 Cankar, K. *et al.* Valencene oxidase CYP706M1 from Alaska cedar (*Callitropsis nootkatensis*). *FEBS Lett.* **588**, 1001-1007 (2014).

15 Cankar, K. *et al.* A chicory cytochrome P450 mono-oxygenase CYP71AV8 for the oxidation of (+)-valencene. *FEBS Lett.* **585**, 178-182 (2011).

16 Ro, D. K., Arimura, G., Lau, S. Y., Piers, E. & Bohlmann, J. Loblolly pine abietadienol/abietadienal oxidase PtAO (CYP720B1) is a multifunctional, multisubstrate cytochrome P450 monooxygenase. *Proc. Natl. Acad. Sci. U S A* **102**, 8060-8065 (2005).

17 Trikka, F. A. *et al.* Combined metabolome and transcriptome profiling provides new insights into diterpene biosynthesis in *S. pomifera* glandular trichomes. *BMC Genom.* **16**, 935 (2015).

18 Ignea, C. *et al.* Carnosic acid biosynthesis elucidated by a synthetic biology platform. *Proc. Natl. Acad. Sci. U S A* **113**, 3681-3686 (2016).

19 Guo, J. *et al.* Cytochrome P450 promiscuity leads to a bifurcating biosynthetic pathway for tanshinones. *New Phytol.* **210**, 525-534 (2016).

20 Pateraki, I. *et al.* Total biosynthesis of the cyclic AMP booster forskolin from *Coleus forskohlii*. *eLife* **6**, e23001 (2017).

21 Steyer, D. *et al.* Genetic analysis of geraniol metabolism during fermentation. *Food Microbiol.* **33**, 228-234 (2013).

22 Brown, S., Clastre, M., Courdavault, V. & O'Connor, S. E. De novo production of the plant-derived alkaloid strictosidine in yeast. *Proc. Natl. Acad. Sci. U S A* **112**, 3205-3210 (2015).
