# Supplementary material for: Expanding the terpene biosynthetic code with non-canonical 16 carbon atom building blocks
Source: Nat Commun. 2022 Sep 3;13:5188. doi: 10.1038/s41467-022-32921-w (PMC9440906; doi:10.1038/s41467-022-32921-w)
Supplement: Supplementary file 3 — Description of Additional Supplementary Files [file 41467_2022_32921_MOESM3_ESM.pdf]

Description of Additional Supplementary Files

File Name: Supplementary Data 1
Description: List of non-canonical (NC) yeast strains constructed in this study to study production of C16 terpenes. All strains were based on AM109 (Mat a/α, PGAL1-HMG2(K6R):: HOX2, ura3, trp1, his3, PTDH3-HMG2(K6R)X2-::leu2, ERG9/erg9, UBC7/ubc7, SSM4/ssm4) described in Reference 1 below.

File Name: Supplementary Data 2
Description: Mass spectra (EI) of C16 terpenoids identified in this study.

File Name: Supplementary Data 3
Description: List of primers used for SpSodMT mutagenesis.
