# Supplementary material for: Expanding the terpene biosynthetic code with non-canonical 16 carbon atom building blocks
Source: Nat Commun. 2022 Sep 3;13:5188. doi: 10.1038/s41467-022-32921-w (PMC9440906; doi:10.1038/s41467-022-32921-w)
Supplement: Supplementary file 7 — Reporting Summary [file 41467_2022_32921_MOESM7_ESM.pdf]

nature portfolio

Corresponding author(s): Sotirios C. Kampranis

Last updated by author(s): Aug 12, 2022

Reporting Summary

Nature Portfolio wishes to improve the reproducibility of the work that we publish. This form provides structure for consistency and transparency in reporting. For further information on Nature Portfolio policies, see our Editorial Policies and the Editorial Policy Checklist.

Statistics

For all statistical analyses, confirm that the following items are present in the figure legend, table legend, main text, or Methods section.

n/a	Confirmed	
☐	☒	The exact sample size (*n*) for each experimental group/condition, given as a discrete number and unit of measurement
☐	☒	A statement on whether measurements were taken from distinct samples or whether the same sample was measured repeatedly
☒	☐	The statistical test(s) used AND whether they are one- or two-sided<br>*Only common tests should be described solely by name; describe more complex techniques in the Methods section.*
☒	☐	A description of all covariates tested
☒	☐	A description of any assumptions or corrections, such as tests of normality and adjustment for multiple comparisons
☐	☒	A full description of the statistical parameters including central tendency (e.g. means) or other basic estimates (e.g. regression coefficient) AND variation (e.g. standard deviation) or associated estimates of uncertainty (e.g. confidence intervals)
☒	☐	For null hypothesis testing, the test statistic (e.g. *F*, *t*, *r*) with confidence intervals, effect sizes, degrees of freedom and *P* value noted<br>*Give P values as exact values whenever suitable.*
☒	☐	For Bayesian analysis, information on the choice of priors and Markov chain Monte Carlo settings
☒	☐	For hierarchical and complex designs, identification of the appropriate level for tests and full reporting of outcomes
☒	☐	Estimates of effect sizes (e.g. Cohen's *d*, Pearson's *r*), indicating how they were calculated

Our web collection on statistics for biologists contains articles on many of the points above.

Software and code

Policy information about availability of computer code

Data collection	BRUKER DALTONICS MS WORKSTATION V8.2.1 for GC-TQ MS quantification,<br>and GCMS Solution for Shimadzu GC-MS quantification.<br>Compass CDS (Version 3.0.1; Bruker Daltonics), Compass oTOF Control (Version 3.4, Bruker Daltonics) and Hystar (Version 3.2 SR4, Bruker Daltonics) for GC-APCI-QqToF acquisition<br>topspin 4.0.8 for NMR data acquisition. MSD ChemStation F.01.03.2357 for data acquisition.
Data analysis	LabSolutions GCMS solution version 4.20 for mass spectra analysis.<br>PyMOL (v. 2.3) for structural analysis.<br>VegaZZ AMMP edition with CG and MM2 algorithms for energy minimization<br>Chem3D (v. 16.0) from (CSC, Cambridge (USA)) for energy minimization<br>ChemDraw Professional 15.1 for chemical structures, MS Excel for bar charts and graphs, and MS Powerpoint for the preparation of illustrations.<br>topspin 4.0.8, MestReC 4.9.9.9, MSD ChemStation F.01.03.2357 for NMR and MS spectra analyses.<br>GC-APCI-QqToFData analysis was performed using Compass DataAnalysis software (Version 4.3, Bruker Daltonics)<br>HyperChem 8.0.8 for Molecular dynamics and geometry optimization studies.

For manuscripts utilizing custom algorithms or software that are central to the research but not yet described in published literature, software must be made available to editors and reviewers. We strongly encourage code deposition in a community repository (e.g. GitHub). See the Nature Portfolio guidelines for submitting code & software for further information.

Data

Policy information about availability of data

All manuscripts must include a data availability statement. This statement should provide the following information, where applicable:

- Accession codes, unique identifiers, or web links for publicly available datasets
- A description of any restrictions on data availability
- For clinical datasets or third party data, please ensure that the statement adheres to our policy

All data necessary to interpret, verify and extend the research presented in the article are provided within the paper and in the Supplementary Information, Supplementary Data, and Source Data files. Supplementary Information contains: Supplementary Tables 1–14, Supplementary Figs. 1–230, and Supplementary Note with information relevant to the structure elucidation. Supplementary Data includes Supplementary Data files 1, 2, and 3. The source data underlying Fig. 4; Supplementary Tables 2, 4, 6, 7, 8; Supplementary Figs. 3, 6, 8, 9 and 10 are provided as a Source Data file. Structural data for PDB id: 5kok is available from: www.rcsb.org/structure/5KOK, and for FPP from: zinc.docking.org/substances/ZINC000012494625. A reporting summary for this article is available as a Supplementary Information file.

Field-specific reporting

Please select the one below that is the best fit for your research. If you are not sure, read the appropriate sections before making your selection.

[x] Life sciences [ ] Behavioural & social sciences [ ] Ecological, evolutionary & environmental sciences

For a reference copy of the document with all sections, see nature.com/documents/nr-reporting-summary-flat.pdf

Life sciences study design

All studies must disclose on these points even when the disclosure is negative.

Sample size	Predetermination of sample size was not relevant to this study. Based on previous experience with the yeast assays performed, replication consisting of three biological replicates was sufficient to provide statistically reliable data.
Data exclusions	No data exclusion.
Replication	All determinations were repeated in triplicates. All attempts at replication of the experiments using re-establishing of yeast strain and reanalysis of the products have been successful.
Randomization	For each of the constructed yeast strains (each biological replicate), 20-30 colonies from the same plate were randomly selected, pooled together, and analyzed. No further allocation into experimental groups was carried out and control of covariates was not relevant.
Blinding	Blinding was not relevant for data collection because the type of analysis carried out could not be biased by lack of blinding

Reporting for specific materials, systems and methods

We require information from authors about some types of materials, experimental systems and methods used in many studies. Here, indicate whether each material, system or method listed is relevant to your study. If you are not sure if a list item applies to your research, read the appropriate section before selecting a response.

Materials & experimental systems

n/a	Involved in the study	
[x]	[ ]	Antibodies
[x]	[ ]	Eukaryotic cell lines
[x]	[ ]	Palaeontology and archaeology
[x]	[ ]	Animals and other organisms
[x]	[ ]	Human research participants
[x]	[ ]	Clinical data
[x]	[ ]	Dual use research of concern

Methods

n/a	Involved in the study	
[x]	[ ]	ChIP-seq
[x]	[ ]	Flow cytometry
[x]	[ ]	MRI-based neuroimaging
